# *In Silico* Survey of the Mitochondrial Protein Uptake and Maturation Systems in the Brown Alga *Ectocarpus siliculosus*

**DOI:** 10.1371/journal.pone.0019540

**Published:** 2011-05-18

**Authors:** Ludovic Delage, Catherine Leblanc, Pi Nyvall Collén, Bernhard Gschloessl, Marie-Pierre Oudot, Lieven Sterck, Julie Poulain, Jean-Marc Aury, J. Mark Cock

**Affiliations:** 1 Université Pierre et Marie Curie, The Marine Plants and Biomolecules Laboratory, UMR 7139, Station Biologique de Roscoff, Roscoff, France; 2 Centre National de la Recherche Scientifique, Unité Mixte de Recherche 7139, Laboratoire International Associé Dispersal and Adaptation in Marine Species, Station Biologique de Roscoff, Roscoff, France; 3 Department of Botany, University of British Columbia, Vancouver, British Columbia, Canada; 4 VIB Department of Plant Systems Biology, Ghent University, Ghent, Belgium; 5 Commissariat à l'Energie Atomique, Direction des Sciences du Vivant, Institut de Génomique, Génoscope, Evry, France; 6 Centre National de la Recherche Scientifique, UMR 8030, Evry, France; 7 Université d'Evry, Evry, France; University of Melbourne, Australia

## Abstract

The acquisition of mitochondria was a key event in eukaryote evolution. The aim of this study was to identify homologues of the components of the mitochondrial protein import machinery in the brown alga *Ectocarpus* and to use this information to investigate the evolutionary history of this fundamental cellular process. Detailed searches were carried out both for components of the protein import system and for related peptidases. Comparative and phylogenetic analyses were used to investigate the evolution of mitochondrial proteins during eukaryote diversification. Key observations include phylogenetic evidence for very ancient origins for many protein import components (Tim21, Tim50, for example) and indications of differences between the outer membrane receptors that recognize the mitochondrial targeting signals, suggesting replacement, rearrangement and/or emergence of new components across the major eukaryotic lineages. Overall, the mitochondrial protein import components analysed in this study confirmed a high level of conservation during evolution, indicating that most are derived from very ancient, ancestral proteins. Several of the protein import components identified in *Ectocarpus*, such as Tim21, Tim50 and metaxin, have also been found in other stramenopiles and this study suggests an early origin during the evolution of the eukaryotes.

## Introduction

There is now strong evidence that mitochondria are derived from an α-proteobacterium that was enslaved by an ancestral eukaryotic cell during a single event that occurred about 1.5 billion years ago [1], [2]. The endosymbiotic process involved both the transfer of many of the symbiont's genes to the host nuclear genome and substantial gene loss. As a result, present day mitochondrial genomes encode only a small number of proteins. The nucleus-encoded mitochondrial proteins needed to be imported into the organelle. Consequently, one of the key steps for the establishment of functional mitochondria was the acquisition of an efficient protein import system. Once established, this system not only served to import proteins encoded by endosymbiont-derived nuclear genes, but also permitted the targeting of novel proteins to the organelle. The original bacterial endosymbiont is thought to have had a proteome that consisted of about 630 distinct proteins [3] whereas present day mitochondria have been estimated to contain about 1,000 to 1,500 different polypeptides [4], [5].

The mitochondrial protein import machinery is complex because a heterogeneous set of proteins needs to be correctly targeted to several different compartments within the mitochondrion: the matrix, the outer membrane (OM), the inner membrane (IM) and the intermembrane space (IMS). These compartments can be considered to be equivalent to the two cellular membranes and the periplasmic space of bacteria. Over the past ten years, studies using yeast have yielded a great deal of information about the molecular mechanisms of mitochondrial protein import. This process involves the cooperation of five membrane and three IMS complexes [6]. The major components of this machinery are conserved in both opisthokonts (animals and fungi) [7], [8] and plantae [9], [10], [11], indicating a very ancient origin [12]. Five translocase complexes are involved in the import of proteins through the mitochondrial membranes and insertion of proteins into the phospholipid bilayer. The Transporter of the Outer Membrane (TOM) and two Transporter of the Inner Membrane complexes (TIM22 and TIM23) are specialized in importing proteins across the membranes. The outer membrane Sorting and Assembly Machinery (SAM) and the inner membrane insertase (OXA) are involved in the insertion of proteins into the membrane.

The TOM complex is the first channel encountered by all proteins that enter mitochondria. The SAM is also located in the OM, where it mediates correct folding of β-barrel proteins. This complex is related to the bacterial β-barrel assembly machinery (BAM), and hence presumably of bacterial origin [13]. SAM is also known as the complex for Topogenesis of mitochondrial Outer membrane β-Barrel proteins (TOB). Unlike the bacterial OM, the mitochondrial OM contains a large number of proteins with an α-helical transmembrane segment suggesting that the ability to insert this type of protein (in addition to β-barrel proteins) evolved in the mitochondrial system [14]. Indeed, it has been demonstrated that the substrate specificity of SAM is not restricted to β-barrel proteins but also includes α-helical proteins [15].

The two major IM translocases, TIM22 and TIM23, perform similar functions. TIM23 imports matrix soluble or weakly hydrophobic proteins (e.g. proteins containing a single a-helix transmembrane domain), whereas TIM22 mediates the transfer of multi-spanning proteins [16]. Most of the components of TIM22 and TIM23 are probably endosymbiont-derived [17]. The third translocase, OXA, is responsible for the insertion of some polytopic proteins into the IM from the matrix side. Oxa1 is the only component of this machinery identified so far [18].

Two small TIM protein complexes consisting of Tim9 and Tim10 and of Tim8 and Tim13, respectively, are located in the IMS. They serve as guide complexes (chaperones), stabilising hydrophobic protein precursors in the hydrophilic medium of the IMS during their transit to their target membrane [19], [20]. Skp performs a similar function in bacteria but is phylogenetically unrelated to the small Tim proteins [21]. Despite its small size, the IMS is an important compartment that contains proteins involved in key functions such as respiration and programmed cell death. The IMS is equivalent to the periplasmic space of prokaryotes and, as in bacterial cells, cysteine residues occur mainly as cystines. In bacteria, thiol oxidation is carried out by the DsbA-DsbB machinery whereas eukaryotes have independently evolved the Mia40-Erv1 system to carry out the same function in mitochondria [22].

Bacteria possess two distinct systems to export folded proteins through the IM, the SECretion (SEC) and Twin-Arginine Transport (TAT) systems [23]. The predominant route is the SEC pathway, although a subset of periplasmic proteins (including many that bind redox-active cofactors) is transported by the TAT system. This machinery has been conserved in plastids but it is not yet clear whether mitochondria possess a functional TAT system [24].

Mitochondrial protein precursors contain specific targeting sequences that allow them to be directed to the correct subcompartment [25], [26]. Most targeting sequences are short, cleavable, N-terminal, amphipathic helices [9], [27], although many membrane proteins possess alternative targeting information within the mature protein [28]. N-terminal targeting peptides are rich in positive, hydrophobic, and hydroxylated amino acids whilst acidic residues are absent. The removal of these cleavable targeting signals is mediated by a variety of proteases. Mitochondria possess five main proteolytic activities that are involved in the maturation of mitochondrial proteins. The five proteases are located in either the IMS or the matrix and include both soluble and membrane-bound enzymes [29].

In this study we searched the genome of the brown alga *Ectocarpus siliculosus* for genes that are predicted to encode components of mitochondrial protein uptake and maturation systems and the complement of import/maturation proteins in *Ectocarpus* was compared with those of other stramenopiles and species from other eukaryotic supergroups. Previous studies of the evolutionary history of the mitochondrial import machinery have concentrated on opisthokonts, green lineage members and a small number of protists including *Giardia*, *Trichomonas*, trypanosomatids and amoebozoa. The inclusion of a comprehensive analysis of *Ectocarpus* and other stramenopiles (diatoms and oomycetes) in the current picture of mitochondrial protein import has provided several insights into the evolutionary history of this essential machinery.

## Methods

### Identification and annotation of mitochondrial proteins

Mitochondrial proteins encoded by the *Ectocarpus* nuclear genome were identified using several different approaches. First, proteins with N-terminal mitochondrial targeting sequences were identified by applying the HECTAR program [30] to the complete set of proteins encoded by the genome [31]. Additional analyses (Table 1 and S1) were carried out with the mitochondrial localisation predictors listed in Table 2. Note that although most of these programs (including HECTAR) only recognise N-terminal targeting sequences, some, such as MitoPred, Cello and SubLoc, also take into account other features [32]. Second, additional mitochondrial proteins were identified by searching for genes that had been assigned either definition descriptions containing the word mitochondrial or gene ontology annotations with mitochondria as the subcellular localisation during the automatic annotation of genome sequence (which was based on matches to existing sequence databases). Finally, detailed manual searches were carried out for specific mitochondrial proteins involved in protein import and maturation. During the manual functional annotation process, particular attention was paid to the identification of protein motifs, transmembrane domains, shared structural characteristic and key amino acids as a means to validate protein function (Tables 2, S2 and S3, Figs. S1 and S2).

**Table 1:** Comparative analysis of mitochondrial protein import systems in *Ectocarpus* and in other stramenopiles.

		STRAMENOPILES					*ECTOCARPUS* DATA		
Name	Alternative names	Oomycetes *P. infestans*	Pelagophytes *A.anophagefferens*	Blastocystis *B.hominis*	Diatoms *P. tricornutum*	Brown algae *E.siliculosus*	Locus ID (protein size)	PREDICTIONS (HECTAR)	PREDICTIONS (ALL)
**OUTER MEMBRANE**									
**mtOM64**		0	0	0	0	0	NI		
**TOM complex**									
**Tom70**	Mas70, Mom72, Omp1	2	0	1	1	2	Esi0007_0019 (952)Esi0232_0002 (739)	NONO	0/96/9
**Tom40**	Isp42, Mom38	1	1	NI	1	1	Esi0055_0058 (442)	YES	7/9
**Tom22**	Mas17, Mas22, Mom22	1	0	0	1	1	Esi0246_0018 (112)	NO	2/9
**Tom20**	Mas20, Mom19	0	0	0	0	0	NI		
**Tom7**	Mom7, Yok22	NI	NI	NI	1	1	Esi0179_0016 (57)	NO	6/9
**Tom6**	Isp6, Mom8B, OM10	NI	NI	NI	NI	0	NI		
**Tom5**	Mom8A, OM7.5, OM5	NI	NI	NI	NI	0	NI		
**SAM /TOB complex**									
**Mim1**	Tom13	0	0	0	0	0	NI		
**Sam50**	Omp85, Tob55, Tom50	1	1	1	1	1	Esi0503_0006 (451)	NO	1/9
**Sam35**	Fmp20, Tob38, Tom38	0	0	0	0	0	NI		
**Sam37**	Mas37, Pet3027, Tom37	1	0	0	0	0	NI	NO	2/9
**Metaxin**		1	0	0	0	1	Esi0338_0018 (407)		
**INTERMEMBRANE SPACE**									
**MIA/ERV complex**									
**Mia40**	Fmp15, Tim40	1	0	1	1	0	NI		
**Erv1**		1	1	1	1	1	Esi0202_0015 (194)	NO	1/9
**Hot13**		1	0	0	1	1	Esi0046_0129 (101)	NO	0/9
**TIM8/13 Complex**									
**Tim8**		2	NI	1	1	1	Esi0109_0044 (125)	NO	0/9
**Tim13**		1	NI	1	1	1	Esi0243_0012 (82)	NO	0/9
**TIM9/10 Complex**									
**Tim10**	Mrs11	1	NI	1	1	1	Esi0041_0146 (107)	NO	0/9
**Tim9**		1	1	NI	1	1	Esi0075_0052 (88)	NO	0/9
**INNER MEMBRANE**									
**TIM23 complex**									
**Tim50**		1	1	0	1	1	Esi0000_0471 (547)	YES	8/9
**Tim44**	Isp45, Mim44, Mpi1	1	1	0	1	1	Esi0086_0051 (602)	YES	8/9
**Tim23**	Mim23, Mpi3, Mas6	1	NI	1	1	1	Esi0047_0026 (206)	NO	1/9
**Tim21**		1	0	0	1	1	Esi0103_0070 (205)	NO	0/9
**Tim17**	Mim17, Mpi2, Sms1	1	1	1	1	1	Esi0117_0080 (264)	NO	0/9
**Pam17**	Fmp18	0	0	0	0	0	NI		
**Tim16**	Pam16, Mia1	1	0	1	1	1	Esi0237_0006 (155)	NO	6/9
**Tim14**	Pam18	1	1	1	1	1	Esi0159_0032 (209)	YES	8/9
**mtHsp70**	Ssc1, Ens1	1	1	1	1	1	Esi0010_0066 (688)	NO	1/9
**Mge1**	Yge1, hTid	1	1	1	1	1	Esi0000_0394 (281)	NO	2/9
**TIM22 complex**									
**Tim54**		0	0	0	0	0	NI		
**Tim22**		1	1	1	1	1	Esi0063_0018 (192)	NO	2/9
**Tim18**		0	0	0	0	0	NI		
**Tim12**	Mrs5	0	0	0	0	0	NI		
**OXA1, polytopic membrane protein insertion and folding**									
**Oxa1**		1	NI	1	1	1	Esi0028_0040 (411)Esi0025_0161 (552)	NOYES	2/98/9
**TAT system**									
**TatA/B**		NI	NI	NI	1	1	Esi0067_0034 (170)	NO	2/9
**TatC**	Orfx, MttB	1	NI	NI	1	1	mt genome (254)		

The table is based on searches of public databases of protein sequences. ALL, predictions obtained using all the subcellular localisation predictors listed in Materials and Methods. NI, not identified. For a more complete list, including species from across the eukaryotic tree, see Table S1.

**Table 2:** List of the programs used for the *in silico* analysis.

Mitochondrial predictions	Program name	Server link	References
	HECTAR	http://www.sb-roscoff.fr/hectar/	[30]
	TargetP v1.1	http://www.cbs.dtu.dk/services/TargetP/	[101]
	Predotar v1.03	http://urgi.versailles.inra.fr/predotar/predotar.html	[102]
	Pprowler v1.2	http://pprowler.itee.uq.edu.au/pprowler_webapp_1-2/	[103]
	MitoprotII	http://ihg2.helmholtz-muenchen.de/ihg/mitoprot.html	[104]
	MitoPred	http://bioapps.rit.albany.edu/MITOPRED/	[105]
	Psort	http://psort.ims.u-tokyo.ac.jp/form.html	[106]
	PsortII	http://psort.ims.u-tokyo.ac.jp/form2.html	
	iPsort	http://ipsort.hgc.jp/	[107]
	Cello v2.5	http://cello.life.nctu.edu.tw/	[108]
	SubLoc v1.0	http://www.bioinfo.tsinghua.edu.cn/SubLoc/eu_predict.htm	[109]

Domains and motifs predictions	Program name	Server link	References
	InterProScan v4.6	http://www.ebi.ac.uk/Tools/InterProScan/	[110]
	Panther v6.1	http://www.pantherdb.org/tools/hmmScoreForm.jsp	[111]
	Pfam v22.0	http://pfam.sanger.ac.uk/search	[112]
	Scan Prosite	http://www.expasy.ch/tools/scanprosite/	[113]
	HHrep ID	http://toolkit.tuebingen.mpg.de/hhrepid	[114]
	TPRpred	http://toolkit.tuebingen.mpg.de/tprpred	[115]

Transmembrane predictions	Program name	Server link	References
	DAS	http://www.sbc.su.se/~miklos/DAS/	[116]
	TMPred	http://www.ch.embnet.org/software/TMPRED_form.html	[117]
	TMHMM v2.0	http://www.cbs.dtu.dk/services/TMHMM/	[118]

2D and 3D structure predictions	Program name	Server link	References
	PHYRE v0.2	http://www.sbg.bio.ic.ac.uk/phyre/	[119]
	SOPMA	http://npsa-pbil.ibcp.fr/cgi-bin/npsa_automat.pl?page=/NPSA/npsa_sopma.html	[120]

3D structure modelling	Program name	Server link	References
	Swiss-Model	http://swissmodel.expasy.org/	[121]
	ProSa Web Server	https://prosa.services.came.sbg.ac.at/prosa.php	[122], [123]
	FATCAT	http://fatcat.burnham.org/fatcat-cgi/cgi/fatcat.pl?-func=pairwise	[124], [125]

Orthologous groups of proteins	Program name	Server link	References
	KOGnitor	http://www.ncbi.nlm.nih.gov/COG/grace/kognitora.html	[126]

### Phylogenetic analyses

Phylogenetic analyses were performed using Phylogeny.fr [33]. Full-length protein alignments were constructed with MUSCLE and were curated by removal of poorly aligned positions using GBlocks (using the lowest stringency parameters), excepting for the Oxa protein phylogeny where only gap positions were removed in order to retain a sufficiently large data set. The resulting set of aligned amino-acids was used for reconstruction of phylogenetic trees using the maximum likelihood (ML) method implemented in PhyML (v3.0 aLRT). The default substitution model was selected, assuming an estimated proportion of invariant sites and four gamma-distributed rate categories to account for rate heterogeneity across sites. The neighbour-joining method was performed with JTT amino acid substitution matrix using BioNJ. For both the ML and NJ methods, bootstrap analyses of 100 replicates were used to provide confidence estimates for the phylogenetic tree topologies. Finally, Bayesian inference analyses were performed on the Metaxin, Tim21 and Tim50 curated amino-acid alignments using the MrBayes program with default parameters.

### Modelling of three dimensional structures and validation

To estimate whether *Ectocarpus* proteins were structurally similar to known Tom70 orthologues, their sequences were carefully checked using a similar procedure to that employed for the validated model of *Blastocystis* Tom70 [34]. First, *Ectocarpus* proteins were analysed with SOPMA to determine their secondary structure composition. Second, opisthokont and *Blastocystis* Tom70 sequences were aligned with other putative stramenopile sequences that exhibited conservation of the TPR domains (Fig. S3). The alignment of Esi0007_0019 (residues 353–945) and Esi0232_0002 (residues 164–739) with the crystal structure of yeast Tom70 (accession number 2GW1A) was then used to generate a homology model at the SIB modelling server.

The quality of the predicted structural models of the putative *Ectocarpus* Tom70 proteins was assessed using the ProSa Web Server [35], [36]. Finally, the predicted structures of Esi0007_0019 and Esi0232_0002 were aligned with the yeast Tom70 template using FATCAT [37], [38].

## Results and Discussion

### The TOM complex

**Figure 1:**
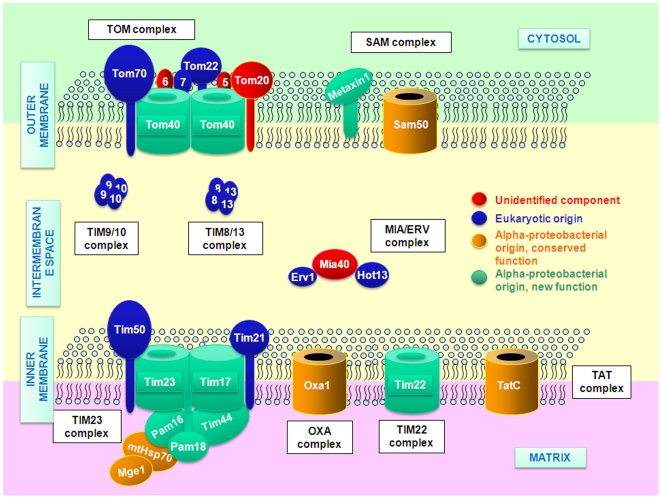
Schematic representation of the predicted mitochondrial protein import machinery in *Ectocarpus*. Proteins of bacterial origin that have conserved their primary function are shown in brown. Proteins which are derived from bacterial ancestors but have evolved a new function in eukaryotes are shown in green. Proteins that have evolved in the eukaryotes are shown in blue. Other proteins that are present in most opisthokonts or plantae but could not be found in *Ectocarpus* have been colored in red.

The TOM complex consists of a central, pore-forming protein, Tom40, which is associated with the small Tom7 and possibly other accompanying proteins such as Tom5, Tom6 (Fig. 1 and Table 1 and S1). In yeast, several receptors (Tom70, Tom20 and Tom22) mediate the recognition of targeting sequences or internal information in the proteins being imported [34]. Tom70 is responsible for the translocation of presequence-less proteins with multiple membrane-spanning domains, which are subsequently directed to the OM or IM [35]. Tom20 and Tom22 carry out similar functions, preferentially importing polypeptides with N-terminal targeting sequences, but do not share sequence similarity [36], [37].

All opisthokont and plant genomes examined so far encode Tom40, Tom22 and Tom7. These three proteins represent the minimum machinery necessary to import proteins from the cytosol into mitochondria and they were all acquired early in mitochondrial evolution [38]. Homologues of Tom40 and Tom7 were identified in *Ectocarpus* (Fig. S1A,B). Esi0246_0018 resembles the short Tom22 found in plants, lacking the acidic N-terminal cytosolic domain typical of the opisthokont proteins (Fig. S1C) [38].

No clear homologue of either the opisthokont-type or the plant-type Tom20 receptor was found in the *Ectocarpus* genome or in other stramenopile genomes. In stramenopiles, it is possible that mitochondrial presequences are recognised by Tom22 rather than Tom20. Tom22 has been reported to share the common signal recognition pathway with Tom20 [34] and both proteins exhibit a chaperone-like activity, stabilising unfolded preprotein substrates [39]. Also, it is important to note that, although the acidic domain of opisthokont Tom22 has been shown to be involved in the recognition of presequences [40], an abundance of negative charges in this cytosolic domain is not essential for binding or import of mitochondrial precursor proteins, suggesting that other features of the domain are involved [41]. Hence, stramenopile Tom22 proteins could recognise mitochondrial presequences, despite the absence of an acidic domain. Finally, there is evidence, for Tom20, that presequence binding is mediated mainly by hydrophobic rather than ionic interactions [42]. Nevertheless, it cannot be excluded that a highly divergent Tom20 receptor or a novel protein with the same role is present in stramenopiles.

**Figure 2:**
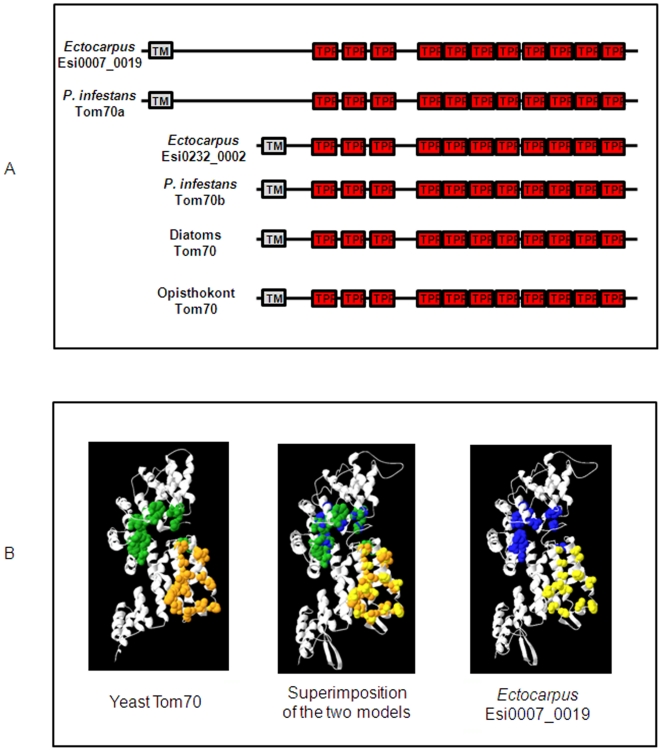
Description of the Tom70 homologues found in stramenopiles. **(A) Comparison of the different domain structures found in Tom70 homologues in *Ectocarpus*, oomycetes and diatoms.** Transmembrane and TPR domains regions are shaded in grey and red respectively. **(B) Model of the *Ectocarpus* Esi0007_0019 protein obtained using the yeast Tom70 crystal structure (pdb accession: 2GW1) as a template.** Residues implicated in substrate binding are coloured in green and blue for yeast and *Ectocarpus,* respectively. Residues involved in dimerisation are coloured in orange and yellow for yeast and *Ectocarpus,* respectively. The Esi0007_0019 model was generated in alignment mode on the SIB website and the pdb file was compared to yeast Tom70 using pdbViewer software. The superimposition of the two models (middle panel) shows the degree of conservation of key amino acids between the two proteins.

Previous studies have indicated that Tom70 is present in opisthokonts but absent from the plantae and many protozoan lineages [43]. But recently, a functional Tom70 gene has been characterised in the stramenopile *Blastocytis sp.* and homologues are present in other stramenopile genomes [44] suggesting that Tom70 did not arise within the opisthokont lineage as previously proposed by Chan *et al.* [43]. Interestingly, *Ectocarpus* possesses a gene (Esi0232_0002) that is homologous with the *Blastocystis sp.* sequence, plus a second Tom70-like gene, Esi0007_0019, both of which share several characteristics with Tom70 including strong sequence similarities with opisthokont Tom70 proteins. Like Tom70, both proteins are predicted to possess eleven distinct TPR domains and an N-terminal transmembrane segment (Figs. 2A and S3A). Moreover, Esi0007_0019 and Esi0232_0002 could be superimposed on the crystal structure model of yeast Tom70 (Figs. 2B and S3B). A high proportion of α-helical structures were predicted for both the *Ectocarpus* proteins (54.8% for Esi0007_0019 and 59.8% for Esi0232_0002). This was comparable with the composition of the yeast (68.2%) and *Blastocystis* (49.9%) proteins. Neither Esi0007_0019 nor Esi0232_0002 were predicted to contain significant amounts of β-sheet. Their Z-scores calculated using the ProSa Web Server (−4.31 for Esi0007_0019 and −4.96 for Esi0232_0002) were in the range of X-ray crystal structures of a comparable size. Using FATCAT, the average reported root mean square deviation values of Esi0007_0019 (1.04 for 478 equivalent positions) and Esi0232_0002 (1.15 for 475 equivalent positions) were reported to be significantly similar with a P value of 0.00e+00 (respective raw scores of 1218.11 and 1245.72). Structure pairs with a probability of <0.05 are significantly similar. The resulting modelled structures exhibited good conservation of the overall α-helical content and several of the potentially key residues for substrate binding and dimerisation identified by Wu and Sha [49] appeared to be conserved. These residues are indicated in the alignment of opisthokont and stramenopile Tom70 sequences shown in Fig. S3A. Note that in many cases it is not the exact amino acid but rather the class of the amino acid (hydrophobic, acidic, basic, etc.) that is conserved. This was to be expected, given the level of conservation of these key residues within the opisthokont and stramenopile groups. Several of the variants at key residues in the *E. siliculosus* proteins (in the TPR9 and TPR10 domains, for example) were also observed in the *Blastocystis* and oomycete Tom70 proteins suggesting a possible modification of preprotein binding interactions in stramenopiles. Only oomycetes seem to have homologues of the Tom70-like Esi0007_0019, suggesting that this protein was lost from diatoms and *Blastocystis*. No Tom70-like proteins were identified in *Aureococcus anophagefferens* (pelagophyceae). The function of these proteins in mitochondrial protein import in *Ectocarpus* will need to be confirmed experimentally.

In plantae, a mitochondrial OM protein similar to chloroplast Toc64 called mtOM64 is thought to carry out a similar role to Tom70 in protein import, although it is not part of the TOM complex [45], [46], [47]. No homologue of mtOM64 was identified in stramenopiles.

### The SAM complex

Sam50, the major SAM subunit, forming the central channel of the complex [48], is derived from the bacterial protein Omp85/BamA [49]. Compared with Omp85/BamA, Sam50 has lost four of the five repeats of the polypeptide transport (POTRA) domains located on the IMS side of the membrane and an N-terminal sequence, but nonetheless carries out the same function as the bacterial protein. Sam50 is accompanied by the peripheral proteins Sam35 and Sam37 [50], [51]. Together they constitute the minimal functional SAM complex in yeast. Animal Metaxin1 and Metaxin2 are distantly related orthologues of Sam37 and Sam35 respectively [17]. Although they are involved in mitochondrial protein import, Metaxins are not part of mammalian SAM complexes [52].

**Figure 3:**
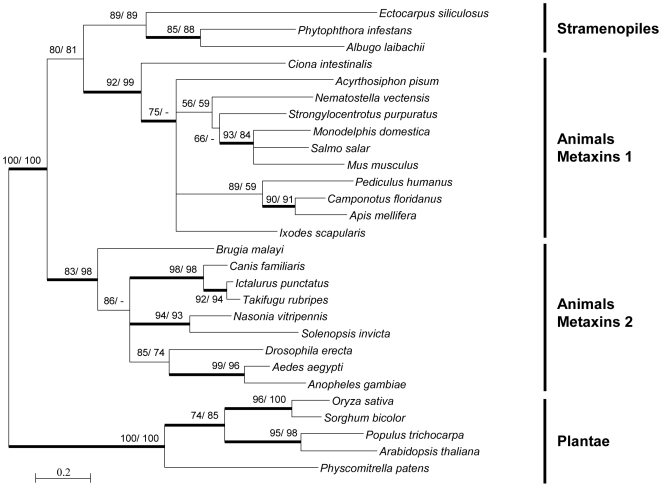
Maximum likelihood tree based on Metaxin protein alignments. A PHYML tree was constructed as described in the materials and methods section, with 108 amino acid sites after curation of the alignment. Bootstrap values above 50 are indicated (100 replicates) for PHYML (first value) and BioNJ (second value) analyses and the thick branches correspond to posterior probabilities of >0.9 with the Bayesian method.

The *Ectocarpus* genome was found to encode homologues of only two SAM proteins, Sam50 (Esi0503_0006) and Metaxin (Esi0338_0018). Comparison of the secondary structures of the yeast Sam50 protein and Esi0503_0006 indicated that they share a conserved α-helix in the N-terminus and a major β-strand structure (Table S2). Moreover, phylogenetic analyses of Sam50 proteins from a broad range of eukaryotes, using the α-proteobacterial Omp85 as outgroup, indicated that, *Ectocarpus* Sam50 is most closely related to the diatom and oomycete homologues and that stramenopile Sam50 proteins seem to cluster independently of well-supported clades formed by the green lineage Sam50 proteins and those of the opisthokont lineage (Fig. S4A). Surprisingly, the phylogenetic analysis of Metaxin proteins showed that the *Ectocarpus* homologue is more closely related to animal Metaxin1 than to animal Metaxin2 and green plant Metaxins, which form two well-supported, independent clusters (Fig. 3). Oomycetes and diatoms also possess a single Metaxin, which is again most similar to animal Metaxin1. In contrast with opisthokonts, which possess two Metaxins, only one of these proteins seems to be required in green plants and stramenopiles. It will be important to identify additional putative metaxin proteins in other stramenopile species and in other eukaryotic lineages in order to determine whether the distribution of these proteins is the result of vertical gene inheritance and successive lost, or due to ancient lateral gene transfer.

### Small TIM Machinery

In many organisms, the IMS contains two small TIM complexes, both of which help direct precursor proteins to their correct destination. In yeast, Tim9 and Tim10 form the major functional complex, which chaperones hydrophobic proteins directed to the SAM or the TIM22 machineries, whereas the non-essential complex formed by Tim8 and Tim13 transports preproteins to the TIM23 complex. The Tim8-Tim13 complex is also involved in the earliest step of Tom40 assembly [20].

The *Ectocarpus* genome encodes homologues of four small Tim proteins (Table 1 and S1) corresponding putatively to Tim8, Tim9, Tim10 and Tim13 on the basis of a motif search carried out using the HMMPanther database (Table S2). All have a double α-helix structure and a twin CX3C motif, except Tim8, which has only one cysteine in each motif (Fig. S1H). Tim9 and Tim10 have a short, internal targeting signal that directs them to the IMS [53]. We identified a similar internal targeting signal in the *Ectocarpus* Tim9 homologue (Fig. S1H).

In fungi, another small Tim protein, Tim12, is associated with the TIM22 complex [54]. This protein, which is anchored to the IM at its C-terminal end, plays an important role in the insertion of polytopic proteins into the phospholipid bilayer. Like animals and plants, *Ectocarpus* does not possess Tim12. In animals, variant forms of Tim9, Tim8, and Tim13 were found instead of Tim12 [55] but no similar sequences could be identified in *Ectocarpus*.

### The MIA-ERV Machinery

Mitochondrial Intermembrane space import and Assembly protein 40 (Mia40) is a receptor protein involved in the import of small cysteine-containing proteins. Mia40 and its partners the FAD-dependent sulfhydryl oxidase Essential for Respiration and Vegetative growth 1 (Erv1) and the zinc finger protein Hot13 regulate the redox states of these proteins. Electrons are delivered to Mia40 by the TOM machinery and then transferred to Erv1. In yeast, Hot13 interacts with Mia40 to remove a zinc atom and thereby facilitates efficient reoxidation by Erv1 [56].

We found Mia40 candidates in diatoms and oomycetes using a low stringency search for the CXXC signature and the twin CX9C motifs of the *D. discoideum* sequence (Fig. S1E) but we failed to identify an *Ectocarpus* homologue.

Erv1 proteins play crucial roles in the biogenesis of cytosolic or mitochondrial Fe/S proteins. The mammalian orthologue is Alr (Augmenter of Liver Regeneration) and Erv1/Alr seem to be ubiquitously distributed across the eukaryote tree with the exception of some protozoans [57]. This enzyme possesses a YPCXXC consensus sequence, which is implicated in the primary redox-reaction [58] and two additional cysteine pairs that are essential for correct function [59]. It has recently been suggested that Erv1 could have originated from marine bacteria via horizontal gene transfer [22] but the *Psychroflexus torquis* Erv1/Alr sequence (ZP_01257237) on which this suggestion was based has since been removed from the NCBI database because it was a contamination. Consequently, the current data suggest that Erv1 was a novel, eukaryotic invention.

The *Ectocarpus* genome encodes two proteins that are highly similar to Erv1/Alr: the first is presumably the mitochondrial sulfhydryl oxidase (Esi0202_0015, Fig. S1F) whereas a second gene (Esi0052_0149) is encoded by an inserted viral genome similar to EsV-1 [60] and its role, if any, remains to be specified.

Curiously, *Ectocarpus* possesses homologues of Hot13 (Fig. S1G) and Erv1, despite the absence of Mia40. Also, like most organisms that apparently lack Mia40 (*Encephalitozoon cuniculi*, *Cryptosporidium parvum*, *Plasmodium falciparum*, *Giardia lamblia*, *Trichomonas vaginalis*), *Ectocarpus* possesses many of the proteins that have been shown to be Mia40 substrates in yeast [22]. As sequence similarity is very low (even between stramenopiles), it is possible that an *Ectocarpus Mia40* homologue exists but that we were not able to identify it. Alternatively, the putative *Ectocarpus* Erv1 and Hot13 proteins could interact with an, as yet, unidentified protein that carries out the same function as Mia40. Note also that some Erv1-like proteins are known to function in the absence of a partner protein [61].

### The TIM23 complex

TIM23 is the largest of the mitochondrial protein import complexes, composed of nine different subunits in yeast [6]. The most important components are the embedded proteins Tim23 and Tim17 and the major receptor for N-terminal targeting peptide recognition, Tim50. Additional proteins include Tim44, Tim16/Pam16, Tim14/Pam18, mtHsp70 and Mge1, all of which are located on the matrix side of the complex. Complete translocation of precursors into the matrix requires the heat shock protein mtHsp70, which is recruited to the translocase by Tim44 and binds to incoming precursors in an ATP-dependent manner. Binding is regulated by the nucleotide exchange factor Mge1 and the J-complex, which consists of Tim14 and Tim16 [62]. Tim21 and Pam17 do not seem to be essential for TIM23 to function; they modulate the activity of the translocase by acting as antagonists [63].

**Figure 4:**
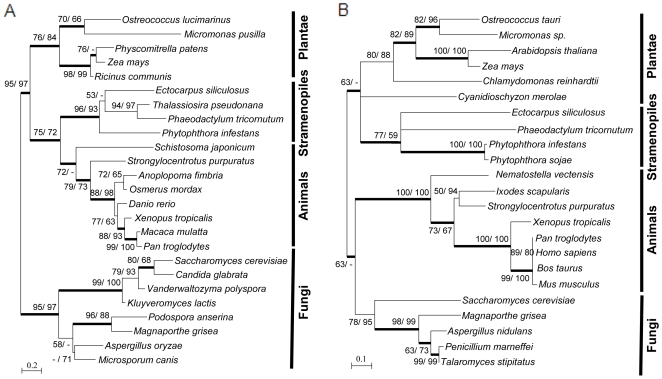
Maximum likelihood trees based on (A) Tim21 and (B) Tim50 protein alignments. PHYML trees were constructed as described in the materials and methods section, with 66 and 149 amino acid sites for Tim21 and Tim50, respectively, after curation of the alignment. Bootstrap values above 50 are indicated (100 replicates) for PHYML (first value) and BioNJ (second value) analyses and the thick branches correspond to posterior probabilities of >0.9 with the Bayesian method.

There are single homologues of *Tim23*, *Tim17*, *Tim44*, *Tim16*/*Pam16*, *Tim14*/*Pam18*, *mtHsp70* and *Mge1* in *Ectocarpus*. All the corresponding proteins appear to be ubiquitous in opisthokonts and green plants (Table 1 and S1). Tim21 and Pam17 were found in opisthokonts but were reported to be absent from *Arabidopsis*, *Physcomitrella* and *Chlamydomonas* [11]. A recent study of plant import systems described the presence of Tim21 homologues [69] and we have identified *Tim21* homologues in *Ectocarpus*, diatoms and oomycetes. Phylogenetic analysis indicated that these proteins group together in a well-supported monophyletic clade, as a sister group to animal Tim21-like proteins (Fig. 4A). On average, Tim21-like proteins shared between 26 and 35% amino-acid identity within each well-defined cluster, but there was a low level of overall sequence conservation. The stramenopile Tim21 proteins were most similar to their opisthokont homologues (15% amino-acid identity). These results suggest that, like their stramenopile counterparts, the newly identified plant Tim21-like proteins may actually be derived Tim21 proteins. It will be necessary, however, to demonstrate biochemically that the latter are components of the TIM23 complex to confirm this hypothesis.

The TIM23 complex imports proteins to both the matrix and the IM and both types of substrate are recognized by Tim50 [64]. Interaction between the IMS domains of Tim50 and Tim23 is important for the transfer of preproteins between the TOM and TIM23 complexes. Tim50 is composed of a single transmembrane region located N-terminal to a large soluble domain exposed to the IMS. The origin of Tim50 is unclear but it shares sequence homology with a family of nuclear LIM interactor-interacting factor-like (NLI-IF) phosphatases, most of which are targeted to the nucleus [65]. Esi0000_0471, the *Ectocarpus* Tim50 homologue, is predicted to be imported into mitochondria and it shares strong similarity with all Tim50 proteins in its C-terminal part. Esi0000_0471 is predicted to possess an N-terminal α-helical transmembrane domain. As with the Tim21 proteins, the Tim50 proteins grouped into four relatively well-supported clusters in phylogenetic analyses (corresponding to green lineage, stramenopile, animal and fungal proteins) but with low resolution as far as deep branching was concerned (Fig. 4B). This phylogenetic analysis indicated that these proteins were acquired early and have evolved independently in each of the major eukaryotic lineages

### The TIM22 complex

TIM22 is responsible for the insertion of carrier proteins in a membrane potential-dependent manner. Tim22 is a member of the Tim17/Tim22/Tim23 family and is the main component of the TIM22 complex. Tim17, Tim23 and Tim22 are all composed of four α-helical segments and it has been suggested that they have evolved from the bacterial LivH protein [10]. In yeast, Tim22 is associated with at least three partners: Tim18, Tim54 and the small Tim12. No counterparts of these proteins have been found in other eukaryotic lineages suggesting that they arose recently in fungi. *Ectocarpus* possesses a single Tim22 gene (*Esi0063_0018*). Two additional genes (*Esi0019_0065* and *Esi0046_0104*) were assigned to the Tim17/Tim22/Tim23 family but they could not be precisely attributed to one sub-family. Their role remains to be elucidated.

Like plants and animals, it is possible that accompanying proteins analogous to fungal Tim54, Tim18 or Tim12 are present in the stramenopile TIM22 complex but at present, there is no biochemical evidence to support this.

### The OXA complex

Oxa1 mediates the insertion of mitochondrial-encoded subunits of respiratory complexes and several nuclear-encoded proteins into the IM from the matrix side. Oxa1 is part of the YidC/Oxa/Alb3 family, which includes proteins that are specialised in the integration of polytopic proteins into membranes in bacteria, mitochondria and chloroplasts, respectively. The members of this large family are derived from an ancestral, bacterial YidC protein [66]. The primary sequences of the members of this family are not well conserved but they share common structural features including five transmembrane segments flanked by N- and C-terminal extensions of variable length [67]. In addition to Oxa1, mitochondria often contain a second member of this family called Oxa2 or Cox18. In yeast, Cox18 is implicated in the insertion of Cox2 into the membrane [68]. Unlike Cox18, Oxa1 has a long C-terminal extension containing a ribosome binding domain capable of interacting with the mitochondrial protein translation machinery. This domain mediates cotranslational insertion of nascent polypeptides [69]. Establishment of the Oxa1 and Oxa2 subfamilies appears to have occurred following an ancient duplication, because most eukaryotes, including plants and opisthokonts possess two Oxa genes [70]. The family has further expanded in other lineages, such as protists, resulting in a rich diversity of these proteins [66]. Three members of the Oxa sub-families were identified in the *Ectocarpus* genome (Table 1 and S1). Our phylogenetic analysis of these proteins (based on the study by Zhang *et al.* [66]) indicated that Esi0028_0040 is probably an Oxa1 homologue, whereas Esi0170_0057 groups with the Oxa2 proteins from oomycetes (Fig. S4B). Alignment of Esi0170_0057 with Oxa1 and Oxa2 proteins showed that it lacks a C-terminal extension supporting its classification as an Oxa2 orthologue (Fig. S5). Esi0025_0161 did not cluster within the Oxa1 and Oxa2 clades and it was therefore not possible to classify it into one of these sub-families. Of the three genes, only *Esi0025_0161* lacked EST support.

### Do mitochondria have a functional TAT system?

The TAT system is a protein-targeting pathway that is found in many bacteria. TatA and TatC are the minimal requirements for a functional pathway [71]. TatB is phylogenetically related to TatA but performs a distinct role in the TAT translocation system [72]. TatC is a polytopic membrane protein with several transmembrane helices. This protein is involved in both the recognition and translocation of proteins containing the “twin-arginine” signal peptide (S/TRRxFLK) consensus sequence [73].

*TatC* homologues have been found in both the mitochondrial and chloroplast genomes of many green plants, algae and protists, including stramenopiles (Organelle Genome Database, http://gobase.bcm.umontreal.ca) [24], [74]. One animal mitochondrial genome has also been shown to contain a homologue [75]. *TatA* and *TatB* have been found in the nuclear genomes of several eukaryotes and phylogenetic analysis has identified a third, intermediate class (called *TatA/B* here) in land plants, green algae and cyanobacteria [71]. The proteins encoded by these nuclear genes are predicted to be targeted to either plastids or mitochondria. Taken together, these data suggest that TAT systems may be present in both the plastids and mitochondria of wide range of photosynthetic eukaryotes. However, although there is now convincing evidence for a functional TAT system in plastids [76], this remains to be demonstrated experimentally for mitochondria. Recently, *TatC* gene expression has been reported in tobacco suggesting that this gene plays a role in mitochondria [77]. Notably, it has been proposed that a TatC pathway might be responsible either for the correct functioning of FeS-containing protein complexes in the IM [78] or for the import and/or export of folded proteins from the matrix into the IMS [24].

*TatC* homologues were found in both the plastid and mitochondrial genomes of *Ectocarpus* [79]. However, the nuclear genome only contains one *TatA/B* homologue (*Esi0067_0034*). The presence of a single, nuclear *TatA/B* gene does not rule out the existence of a mitochondrial TAT system provided that the protein is targeted to both the plastids and the mitochondria. MitoprotII and SubLoc predict that Esi0067_0034 has a mitochondrial destination. There is now clear evidence that many proteins can be targeted to both mitochondria and chloroplasts in plants [80] and to both mitochondria and the endoplasmic reticulum in yeast [81]. *In silico* and biochemical studies suggest that the plastids of land plants have two distinct but closely related TAT complexes. In some instances, TatB may be absent, in which case TatA alone may be associated with TatC [82]. It is therefore possible for a functional TAT system to consist of TatC plus a single protein of the TatA/TatB family. Taken together, therefore, analysis of the genome indicated that *Ectocarpus* might possess a mitochondrial TAT system consisting of a TatA/B and a TatC. However, we cannot rule out the possibility that TatC only plays a role in the recognition of twin-arginine proteins in the mitochondrion, in which case it would not necessarily function as a component of a TAT system.

### Inner membrane peptidases

**Figure 5:**
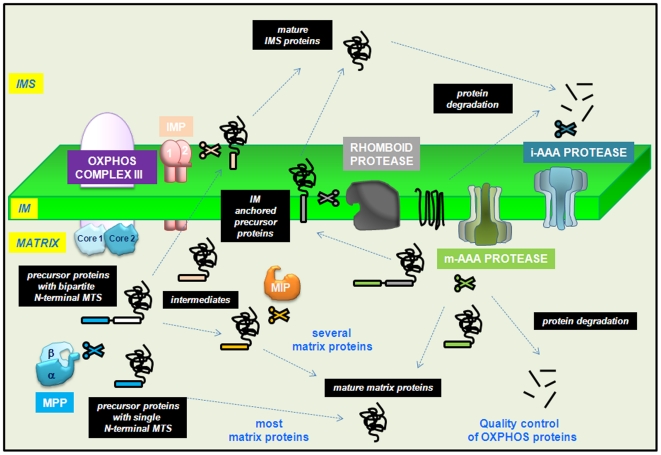
Schematic representation of stramenopile mitochondrial peptidases. Three of the five mitochondrial peptidases implicated in protein maturation after import are located on the inner membrane. The IMP and Rhomboid proteases release mature proteins into the IMS after cleavage of a transmembrane segment (represented respectively in pink and grey) whereas the m-AAA protease cuts precursors in the matrix side (green). m-AAA protease and its counterpart on the IMS side of the inner membrane are also involved in the general degradation pathway of mitochondrial proteins. The two main enzymes that produce mature mitochondrial proteins are the matrix-located MPP and MIP. They can act sequentially or independently to generate functional proteins. The peptides recognized by MPP and MIP are colored in blue and orange, respectively. In *Ectocarpus*, non functional paralogues of β-MPP and α-MPP (core1 and core2 subunits) were identified in the genome suggesting their presence in the complex III of respiratory chain (OXPHOS). Inner membrane (IM); intermembrane space (IMS).

Mitochondrial peptidase complexes play important roles both in the removal of N-terminal targeting peptides and in the turnover of mitochondial proteins. Of the five major classes of mitochondrial peptidase complex, three are located on the IM (Fig. 5).

The Inner Membrane Peptidase complex (IMP) is derived from the α-proteobacterial leader peptidase LepB [83]. Bacterial IMP is monomeric but mitochondrial IMP is a heterodimer of Imp1 and Imp2 proteins. Imp1 and Imp2 are relatively similar, possessing the same catalytic S and K residues [84]. Imp1 is more similar to LepB than Imp2. Each subunit is anchored to the IM with its N-terminal extremity and the C-terminal catalytic domains are located in the IMS [85]. Recently, it has been proposed that the motifs RX5P and NX5S, found in the catalytic domains of Imp1 and Imp2 respectively, are discriminatory for these two proteins in most species [84].

Two Imp1 homologues and one Imp2 homologue were identified in *Ectocarpus* (Table 3). All have the conserved catalytic residues and the discriminatory motif RX5P is present in Esi0007_01117 and Esi0009_0118 while an imperfect NX5p motif was found in Esi0324_0009. Moreover, analysis of an alignment that included plant and stramenopile sequences indicated that new and more restrictive consensuses (GDNX7RX5P and EGDX8NX5(S/P)) could be defined for Imp1 and Imp2, respectively. IMP substrates have been well studied in opisthokonts (Table 4). Homologues of four of the six proteins that have shown to be Imp1 substrates in other species were identified in the *Ectocarpus* nuclear (*Mcr1, GPDM* and *AIF*) and mitochondrial (*cox2*) genomes, as well as a homologue of the Imp2 substrate *cyt c1*. Further analysis will be required to determine whether these proteins are substrates of IMP in *Ectocarpus*, but their presence suggests that there is a need for functional IMP complexes in this brown alga.

**Table 3:** *Ectocarpus* mitochondrial proteases.

Subunit name	Locus ID	Phylogenetic origin	Structural features	mt Pred.	Known substrates
**Inner Membrane Peptidase (IMP)**					
Imp1	Esi0007_0117	α-proteobacteria, leader peptidase (LepB)	caltalytic S/K, RX_5_P	6/9	Mcr1, cyt b2, GPDM/Gut2, Cox2, DIABLO/Smac, AIF*
Imp1	Esi0009_0118	α-proteobacteria, leader peptidase (LepB)	caltalytic S/K, RX_5_P	8/9	
Imp2	Esi0324_0009	α-proteobacteria, leader peptidase (LepB)	caltalytic S/K, NX_5_S	2/9	cyt c1
**Inner Membrane AAA Protease**					
m-AAA Protease	Esi0035_0150	α-proteobacteria, metalloprotease (FtsH)	ATP-binding, HEXXH	8/9	Ccp1, Mrpl32
i-AAA Protease	Esi0028_0127	α-proteobacteria, metalloprotease (FtsH)	ATP-binding, HEXXH	9/9	
**Mitochondrial Rhomboid Protease**					
(Pcp1/Rbd1)-like	Esi0140_0003	prokaryote, rhomboid protease	caltalytic N/S/H	0/9	Mgm1/Opa1, Ccp1, HtrA2/Omi, Pink1
(AtRBL12/PARL/Rho-7)-like	Esi0044_0125	prokaryote, rhomboid protease	caltalytic N/S/H	0/9	
(PARL/Rho-7)-like	Esi0140_0044	prokaryote, rhomboid protease	caltalytic N/S/H	1/9	
**Mitochondrial Processing Peptidase (MPP)**					
MPP alpha subunit	Esi0268_0010	α-proteobacteria, metalloprotease (RPP)	GGGGSFSAGGPGKGM(Y/F)SRLY	9/9	Most matrix proteins [29]
MPP beta subunit	Esi0098_0070	α-proteobacteria, metalloprotease (RPP)	HXXEH(X)_76_E	9/9	
**Mitochondrial Intermediate Peptidase (MIP)**					
MIP	Esi0033_0093	prokaryote, oligopeptidase A (OpdA)	FHEXGH(X)_2_H(X)_12_G(X)_5_D(X)_2_EXPS(X)_3_E	8/9	Several matrix proteins [29]

Substrate short name	Locus ID	Substrate full name	Publication
Mcr1	Esi0179_0058	NADH-cytochrome b5 reductase	[127]
cyt b2	NI	cytochrome b2	[128]
GPDM/Gut2	Esi0117_0019	FAD-dependent glycerol-3-phosphate dehydrogenase	[88]
Cox2	mt encoded	subunit II of cytochrome c oxidase	[129]
DIABLO/Smac	NI	caspase activator	[84]
AIF	Esi0098_0001	apoptosis-inducing factor	[130]
cyt c1	Esi0003_0099	cytochrome c1	[131]
Ccp1	Esi0095_0043	cytochrome c peroxidase	[88], [92]
Mrpl32	NI	mitochondrial ribosomal protein L32	[89]
Mgm1/Opa1	Esi0024_0159	dynamin-related GTPase	[92], [132]
HtrA2/Omi	Esi0509_0002	serine protease	[133]
Pink1	NI	serine/threonine protein kinase	[133]

Conserved residues in structural features and substrates found in *E.siliculosus* genome are underlined. Substrate information is detailed in Table 4. mt Pred, fraction of the subcellular localisation predictor programs (see Table 2) that predict a mitochondrial location.

**Table 4:**
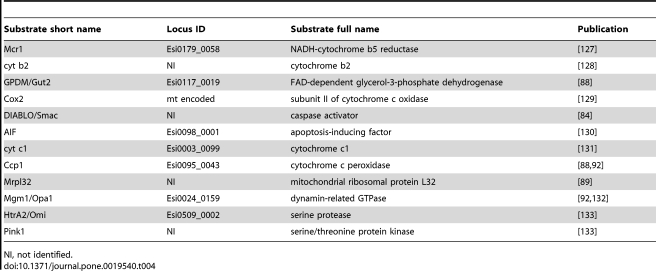
Putative substrates of the *Ectocarpus* mitochondrial proteases.

NI, not identified.

m-AAA protease, a member of the AAA superfamily (ATPases Associated with diverse cellular Activities), is principally involved in the proteolysis of membrane proteins and in the maintenance of mitochondrial activities. It is anchored in the IM with the catalytic domain in the matrix. A similar enzyme, i-AAA protease, is required for degrading polypeptides between the mitochondrial membranes. Both enzymes are involved in the turnover of unincorporated or mistranslated mitochondrial translation products, notably in the quality control of several OXPHOS proteins [86]. Phylogenetic analysis has revealed three main types of enzyme, represented by *S. cerevisiae* Yme1, human Spg7/paraplegin and *S. cerevisiae* Yta10/Afg3 and Yta12/Rca1, respectively [87]. Yme1 is an i-AAA protease, whereas the other three are m-AAA proteases. Bacteria generally contain only one AAA protease, the FtsH metalloprotease. The m-AAA and i-AAA proteases probably evolved from an ancestral α-proteobacterial FtsH gene via a duplication mechanism that created a novel and specific pathway for processing of a subset of matrix proteins including Ccp1 and MrpL32 [88], [89]. The *Ectocarpus* genome encodes both i-AAA and m-AAA proteases (Table 3), the latter being more similar to paraplegin than to yeast m-AAA proteases. Both *Ectocarpus* proteins have the ATP binding and the HEXXH metal binding domains required for activity [90].

Rhomboid proteases are a small, highly conserved protein family within the superfamily of serine proteases. Generally rhomboid proteases function in intercellular communication but recently some of them have been shown to act within the lipid bilayer of the mitochondrial IM [91]. These integral membrane proteins are intramembrane cleaving proteases, cutting substrates at membrane-spanning segments. Two rhomboid proteases, Pcp1/Rbd1 and Rbd2, have been isolated from the IM of yeast mitochondria [92]. Mitochondrial rhomboid proteases have also been found in other eukaryotes including human (PARL), *Drosophila* (Rho-7) and *A. thaliana* (AtRBL12). Based on sequence similarity, there appear to be ten rhomboid-like proteases in *Ectocarpus.* Esi0140_0003 is similar to Pcp1/Rbd1. No clear homologue of Rbd2 was detected. Esi0044_0125 shares similarity with the human, *Drosophila* and *Arabidopsis* sequences. A third sequence Esi0140_0044 may also be related to these proteins. The catalytic residues of rhomboid proteases (an Asn-Ser-His triad [91]) are conserved in Pcp1/Rbd1, PARL, Rho-7 and AtRBL12. Esi0140_0003 and Esi0140_0044 possess all three residues but Esi0044_0125 contains only the serine. Three substrates of Pcp1 have been described and two of these, Mgm1 and Ccp1, are predicted to be present in *Ectocarpus*. Both are expected to reside in the IMS sub-compartment. Note, however, that in humans Opa1 (the homologue of Mgm1) is not processed by PARL (the homologue of Pcp1) and therefore experimental evidence will be required to confirm that these proteins are actually Pcp1 substrates in *Ectocarpus*.

### Matrix peptidases

The mitochondrial processing peptidase (MPP) is the principal protease that cleaves N-terminal targeting sequences of nuclear encoded proteins during their transport into the organelle [93]. MPP is a heterodimeric enzyme made up of α-MPP and β-MPP subunits. In mammals and yeast, the enzyme is localized in the matrix whilst in land plants MPP is a component of complex III of the respiratory chain, replacing the Core1 and Core2 subunits. An intermediate situation is found in some fungi, like *Neurospora crassa*, which have a fully active, soluble α/β-MPP complex in the matrix and a partially active Core2/β-MPP complex associated with complex III [94], [95]. MPP subunits and Core subunits have probably evolved from the bacterial metallopeptidase RPP [17]. All known β-MPP subunits possess an inverted zinc binding motif HXXEH(X)76E, which is required for zinc binding and catalytic activity of MPP. This sequence is less conserved in Core and a-MPP proteins. Both the α and β components of MPP are normally inactive in the monomeric state, but it has been recently demonstrated that β-MPP orthologues in some protozoans are functional as monomers [96], [97]. This observation suggests that α-MPP and β-MPP are derived from an ancestral protein that was active as a monomer.

Four MPP-homologous genes were identified in *Ectocarpus,* corresponding to two putative α-MPP/Core2-type (Esi0268_0010 and Esi0111_001) and two putative β-MPP/Core1-type (Esi0098_0070 and Esi0011_0084) proteins. Alignment of their sequences showed that only Esi0098_0070 possessed the inverted zinc-binding consensus HXXEH(X)76E suggesting that Esi0011_0084 is a Core1 subunit of complex III without enzymatic activity (Fig. S6A). Similarly, Esi0268_0010 possesses the essential glycine-rich region and probably corresponds to the α-MPP subunit whilst Esi0111_0016, which does not share this feature, would probably correspond to Core2 of the respiratory chain complex III.

In contrast to the situation in *Ectocarpus*, we only found two or three MPP genes in other stramenopile genomes. For example, as has been previously described for land plants, *P. tricornutum* possesses putative α-MPP and β-MPP subunits but no Core proteins (Fig. S6B). *T. pseudonana* and *Phytophthora* species appear to have α-MPP and β-MPP plus an additional α-MPP/Core2-type subunit, which is probably inactive. A recent proteomic analysis of *Chlamydomonas reinhardtii* reported the existence of a Core2 protein in addition to the soluble α-MPP and β-MPP [98]. This resembles more closely the situation described for *N. crassa* than that described for land plants. The situation in *Ectocarpus* is similar to that described for opisthokonts, except that *Ectocarpus* may also have a Core2 subunit. Note that β-MPP is probably able to associate with Core2 as well as with α-MPP.

Mitochondrial Intermediate Peptidase (MIP) is a thiol-dependent metalloprotease, which has the ability to cleave intermediate-size proteins following cleavage by MPP. MIP proteins are related to bacterial zinc metalloproteases. Many proteins that are targeted to the mitochondrial matrix or to the IM are processed in two sequential steps, first by MPP and then by MIP. After MPP cleavage at R+1, a typical processing octapeptide-carrying intermediate is generated and subsequently recognized by MIP, leading to production of the mature protein [99]. The *Ectocarpus* gene *Esi0033_0093* is predicted to encode an MIP protein.

### Conclusion

Despite the large evolutionary distances that separate the stramenopiles from well-studied organisms such as yeast or *Arabidopsis*, most of the core components of mitochondrial protein import systems appear to be encoded by the *Ectocarpus* genome. On the other hand, in many instances we failed to find genes encoding accessory proteins (small Toms, Tim12 or Pam17 for example) and this is consistent with previous observations suggesting that these proteins often arose later in evolution within individual eukaryotic lineages to increase the efficiency of the import systems [12]. Components that are known to be different between opisthokonts and plantae, such as TOM complex receptors, were also variable in *Ectocarpus*. However, a candidate counterpart for Tom70, plus a closely related Tom70-like protein, were identified in this brown alga, concurring with the recent finding of Tsaousis *et al.* [44] that the Tom70 receptor is not found only in opisthokonts. Additional potential Tom70 homologues were identified in recent genomic data for other stramenopiles, suggesting that the presequence-less import pathway mediated by Tom70 could be more ancient than previously supposed, being acquired before the separation of opisthokonts and stramenopiles. In contrast, no Tom20 counterpart was identified in *Ectocarpus*. Even if Tom22 is also able to bind N-terminal presequences and has been proposed to be the ancestral receptor for the presequence-mediated mitochondrial import, Tom20 is the major protein involved in this pathway in opisthokonts and plantae. This component, if it exists in brown algae, remains to be discovered.

Other interesting observations included the absence of Mia40, despite the apparent presence of other components of the MIA/ERV system, and the identification of stramenopile Tim21-like proteins related to opisthokont Tim21, suggesting that this protein might be more broadly distributed across the eukaryotic tree that previously thought.

All the known mitochondrial peptidases appear to be present in *Ectocarpus,* including the IMP, m-AAA and rhomboid proteases, MPP and MIP. Interestingly, different combinations of MPP and Core subunits were predicted to be present in diverse stramenopiles, as has been reported for opisthokonts and plantae. This indicates that incorporation of MPP into the respiratory chain could have occurred several times during the evolution of eukaryotes.

Previous studies focusing on the plantae [69] and opisthokont [7], [8] lineages, and on other protist groups [106] have indicated that mitochondrial protein import complexes consist of very ancient and conserved components together with a variety of variable accompanying proteins depending on the lineage. The analysis presented here indicates that a similar situation exists in the stramenopiles, with individual protein uptake complexes exhibiting more or less complexity depending on the subgroup analysed.

## Supporting Information

Figure S1 **Alignments of conserved regions of predicted** ***Ectocarpus*** **mitochondrial proteins with the corresponding regions from their eukaryotic homologues. (A) Alignment of Tom7 proteins from diverse eukaryotes.** EctsiTom7 (Esi0179_0016, *Ectocarpus siliculosus*), ChlreTom7 (XP_001690575, *Chlamydomonas reinhardtii*), ArathTom7-1 (NP_568593, *Arabidopsis thaliana*), ApimeTom7 (NP_001155870, *Apis mellifera*), VitviTom7 (XP_002269243, *Vitis vinifera*), RiccoTom7 (XP_002523617, *Ricinus communis*), SacceTom7 (CAA95944, *Saccharomyces cerevisiae*), NeucrTom7 (AAK18812, *Neurospora crassa*), HomsaTom7 (NP_061932, *Homo sapiens*), SoltuTom7 (O82067, *Solanum tuberosum*), PhatrTom7 (sequence obtained from [38], *Phaeodactylum tricornutum*). Transmembrane sequence and Tom7 motif are shaded in grey and dark grey respectively (this alignment representation is based on Fig. 2, Maćasev *et al.* [38]). **(B) Alignment of Tom40 proteins from diverse eukaryotes.** EctsiTom40 (Esi0055_0058, *Ectocarpus siliculosus*), PhatrTom40 (XP_002182279, *Phaeodactylum tricornutum*), ThapsTom40 (XP_002293745, *Thalassiosira pseudonana*), PhysoTom40 (Physo1_1_108992, *Phytophthora sojae*), PhyraTom40 (Phyra1_1_72372, *Phytophthora ramorum*), PhyinTom40 (EEY60531, *Phytophthora infestans*), MicpuTom40 (EEH60685, *Micromonas pusilla*), ChlreTom40 (XP_001702575, *Chlamydomonas reinhardtii*), ArathTom40-1 (NP_188634, *Arabidopsis thaliana*), ArathTom40-2 (NP_175457, *Arabidopsis thaliana*), PhypaTom40 (XP_001783489, *Physcomitrella patens*), OstluTom40 (XP_001417025, *Ostreococcus lucimarinus*), SacceTom40 (EDN64139, *Saccharomyces cerevisiae*), ToxgoTom40 (EEE23864, *Toxoplasma gondii*), PermaTom40 (EER18361, *Perkinsus marinus*), PlafaTom40 (CAG24986, *Plasmodium falciparum*), HydmaTom40 (XP_002159190, *Hydra magnipapillata*), NeucrTom40 (XP_961545, *Neurospora crassa*), HomsaTom40 (O96008, *Homo sapiens*), DicdiTom40 (EAL68763, *Dictyostelium discoideum*), CyameTom40 (AP006488 DNA, *Cyanidioschyzon merolae*), CioinTom40 (XP_002131405, *Ciona intestinalis*). Two predicted β-strands are indicated in yellow as shown in Fig. 1 of Maćasev et al. [38]. The PoxGxxφxφ consensus has been described in Kutik *et al.* [100] as an important C-terminal motif for recognition by β-barrel proteins assembly machinery (where Po is a polar amino acid, φ****a hydrophobic residue and x any amino acid). **(C) Alignment of Tom22 proteins from diverse eukaryotes.** EctsiTom22 (Esi0246_0018, *Ectocarpus siliculosus*), PhatrTom22 (XP_002179609, *Phaeodactylum tricornutum*), PhyinTom22 (EEY62448, *Phytophthora infestans*), OstluTom22 (XP_001416904, *Ostreococcus lucimarinus*), MicpuTom22 (EEH59409, *Micromonas pusilla*), SacceTom22 (CAA96013, *Saccharomyces cerevisiae*), NeucrTom22 (XP_956695, *Neurospora crassa*), HomsaTom22 (BAB16408, *Homo sapiens*), DromeTom22 (NP_523910, *Drosophila melanogaster*), ArathTom22-1 (NP_563699, *Arabidopsis thaliana*), ArathTom22-2 (NP_199210, *Arabidopsis thaliana*). Transmembrane region (grey and dark grey) exhibits a conserved WxxxT(T/S)xxxxxxP as observed in [38]. Acidic residues (D/E) are shown in red and basic residues (K/R) are in light blue. The cytosolic region is larger in opisthokonts with a patch of acidic amino acids. This additional domain is currently restricted to opisthokonts (not found in plantae, stramenopiles and other protists). **(D) Alignment of Mia40 proteins from diverse eukaryotes.** Stramenopiles are represented by *Thalassiosira pseudonana* (Thaps, XP_002292670), *Phytophthora ramorum* (Phyra, Phyra1_1_82670), *Phytophthora sojae* (Physo, Physo1_1_133729), *Phaeodactylum tricornutum* (Phatr, XP_002182915). Plantae sequences include *Ostreococcus lucimarinus* (Ostlu, XP_001419132) and *Micromonas pusilla* (Micpu, EEH56979), a red alga *Cyanidioschyzon merolae* (Cyame, AP006492) and a land plant *Arabidopsis thaliana* (Arath, NP_680211) and *Physcomitrella patens* (Phypa, XP_001765902). The opisthokont sequences are from *Homo sapiens* (Homsa, 2K3J) and Sacce (*Saccharomyces cerevisiae*). Amoeboza are represented by *Dictyostelium discoideum* (Dicdi, XP_643501). Mia40 was not found in the *Ectocarpus* genome. A new CXXC consensus (colored black) than previously described CPC signature is present in Mia40 homologues and this sequence is followed by a twin CX_9_C motif (colored black). **(E) Alignment of Erv1 proteins from diverse eukaryotes.** SacceErv1 (NP_011543, *Saccharomyces cerevisiae*), HomsaALR (CAB87993, *Homo sapiens*), EctsiErv1 (Esi0202_0015, *Ectocarpus siliculosus*), ThapsErv1 (XP_002291504, *Thalassiosira pseudonana*), PhatrErv1 (XP_002185840, *Phaeodactylum tricornutum*), PhyinErv1 (EEY69279, *Phytophthora infestans*), PhysoErv1 (Physo1_1_129835 estExt, *Phytophthora sojae*), PhyraErv1 (Phyra1_1_93536 C, *Phytophthora ramorum*), ArathErv1 (NP_564557, *Arabidopsis thaliana*), ChlreErv1 (EDP03768, *Chlamydomonas reinhardtii*), CyameErv1 (AP006502 DNA, *Cyanidioschyzon merolae*). Cys residues are shaded in brown for Stramenopiles, in green for plantae and in blue for opisthokonts. YPCXXC and CX16X motifs are conserved in all Erv1 proteins and are important for FAD binding [59]. Specific twin Cys motifs are present in SacceErv1 (CRSC), HomsaALR (CRAC AND CX10C), in ArathErv1 (CEQKSC) and in Stramenopiles (CX4C). **(F) Alignment of Hot13 proteins from diverse eukaryotes.** In panel 1, EctsiHot13 (Esi0046_012, *Ectocarpus siliculosus*), PhysoHot13 (Physo1_1_123261 estExt, *Phytophthora sojae*), PhyraHot13 (Phyra1_1_49641, *Phytophthora ramorum*), PhyinHot13 (EEY65160, *Phytophthora infestans*), CyameHot13 (AP006495 DNA, *Cyanidioschyzon merolae*), SacceHot13 (AAS56591, *Saccharomyces cerevisiae*), NeucrHot13 (Neurospora crassa, EAA28094). Conserved Cys and His of Zinc-ion coordination are shaded in black as referred to panel 2. **(G) Conserved motifs in the** ***Ectocarpus*** **small Tim proteins.** Part a. Alignment of *E. siliculosus* small Tim sequences showing the twin CX3C motif (conserved cysteines are shaded in black and conserved residues with consensus determinedin [55] are in blue). Part b. Example of the presence of an internal sequence signal for import into mitochondria in *E. siliculosus* Tim9 as observed in different eukaryotic species (Ectsi: *Ectocarpus siliculosus*, Sacce: *Saccharomyces cerevisiae*, Neucr: *Neurospora crassa*, Orysa: *Oryza sativa*, Homsa: *Homo sapiens*, Drome: *Drosophila melanogaster*). **(H) Partial alignment of Tim44 proteins. (I) Partial alignment of Tim23 proteins. (J) Partial alignment of Tim17 proteins. (K) Partial alignment of Tim16 proteins. (L) Partial alignment of Tim14 proteins. (M) Partial alignment of mtHsp70 proteins. (N) Partial alignment of Mge1 proteins. (O) Partial alignment of Tim22 proteins.** For (H) to (O) accession numbers and corresponding names of species are listed in Table S3. Black and grey coloured residues correspond to the amino acid identities (>80% and >60% respectively).
(PDF)

Figure S2 **Alignments of complete, predicted** ***Ectocarpus*** **mitochondrial proteins with their eukaryotic homologues. (A) Metaxin, (B) Tim21, (C) Tim50, (D) Sam50, (E) YidC, Oxa1, Cox18/Oxa2, Alb3 proteins.** These alignments were used for the phylogenetic analyses. Accession numbers and corresponding names of species are presented in Table S3. Black and grey coloured residues correspond to the amino acid identities (>80% and >60% respectively).
(PDF)

Figure S3 **Alignment of Tom70 proteins showing the presence of a single transmembrane region and multi-TPR domains.** TPR regions have been superimposed on the *Saccharomyces cerevisiae* model and these domains were validated by bioinformatic analysis using the HHrep ID and TPRpred tools. Transmembrane regions were predicted by DAS and TMpred. Residues shaded in green (opisthokonts) or blue (stramenopiles) represent amino acids implicated in substrate binding and red-coloured (opisthokonts) or yellow-coloured (stramenopiles) positions correspond to residues involved in dimerisation according to [34], [49]. Esi0007_0019 and Esi0232_0002 are the *Ectocarpus siliculosus* proteins and the sequences from other organisms correspond to *Blastocystis sp.* (Blasp), *Phaeodactylum tricornutum* (Phatr), *Phytophthora infestans* (Phyin), *Pichia pastoris* (Picpa), *Saccharomyces cerevisiae* (Sacce), *Naumovia castellii* (Nauca), *Neurospora crassa* (Neucr), *Aspergillus nidulans* (Aspni), *Trichoplax adhaerens* (Triad), *Nematostella vectensis* (Nemve), *Danio rerio* (Danre), *Homo sapiens* (Homsa) and *Xenopus laevis* (Xenla).
(PDF)

Figure S4 **Maximum likelihood trees based on alignments of (A) Sam50 and (B) Oxa protein families.** PHYML trees were constructed as described in the materials and methods section, with 113 and 88 amino acid sites for Sam50 and Oxa trees, respectively. Bootstrap values (100 replicates) above 50 are indicated for Maximum likelihood (first value) and Neighbour-Joining (second value) analyses.
(PDF)

Figure S5 **Alignment of the C-terminal regions of Oxa1 family proteins.** C-terminal domains characteristic of Oxa1 and Oxa2 are boxed.
(PDF)

Figure S6 **MPP proteins. (A) Alignment of MPP and core subunit motifs from diverse eukaryotes.** Esi0111_0016, Esi0268_0010, Esi0011_0084 and Esi0098_0070 are the *Ectocarpus* sequences. The sequences from other organisms are from *Homo sapiens* (Homsa), Saccharomyces cerevisiae (Sacce), *Neurospora crassa* (Neucr), *Chlamydomonas reinhardtii* (Chlre), *Solanum tuberosum* (Soltu). alphaMPP and betaMPP indicate functional MPP subunits (shown in negative type). Core2 and core1 represent the corresponding non-functional MPP homologues inserted into the respiratory chain. The highlighted residues correspond to the key amino acids of alphaMPP and betaMPP activities. **(B) Schematic representations of mitochondrial MPP complexes from diverse eukaryotes.** Panels 1, 2 and 3 reflect the evolution of MPP proteins as suggested by [94]. See text for details.
(PDF)

Table S1 **Comparative analysis of mitochondrial protein import systems across the eukaryotic tree.** The table is based on searches of public databases of protein sequences. ALL, predictions obtained using all the subcellular localisation predictors listed in Materials and Methods. NI, not identified.
(PDF)

Table S2 **Summary of the results of manual searches for components of mitochondrial protein import systems.** Relevant hits are in bold characters.
(PDF)

Table S3 **Mitochondrial protein import components across the eukaryotic tree.** These accessions have been used in amino acid alignments and phylogenetic analyses. Note that the table includes both proteins identified in previous published studies and proteins found in this study by manual searches.
(PDF)
