# Supplementary material for: In Silico Survey of the Mitochondrial Protein Uptake and Maturation Systems in the Brown Alga Ectocarpus siliculosus
Source: PLoS One. 2011 May 18;6(5):e19540. doi: 10.1371/journal.pone.0019540 (PMC3097184; doi:10.1371/journal.pone.0019540)
Supplement: Figure S1 — Alignments of conserved regions of predicted Ectocarpus mitochondrial proteins with the corresponding regions from their eukaryotic homologues. (A) Alignment of Tom7 proteins from diverse eukaryotes. EctsiTom7 (Esi0179_0016, Ectocarpus siliculosus), ChlreTom7 (XP_001690575, Chlamydomonas reinhardtii), ArathTom7-1 (NP_568593, Arabidopsis thaliana), ApimeTom7 (NP_001155870, Apis mellifera), VitviTom7 (XP_002269243, Vitis vinifera), RiccoTom7 (XP_002523617, Ricinus communis), SacceTom7 (CAA95944, Saccharomyces cerevisiae), NeucrTom7 (AAK18812, Neurospora crassa), HomsaTom7 (NP_061932, Homo sapiens), SoltuTom7 (O82067, Solanum tuberosum), PhatrTom7 (sequence obtained from [38], Phaeodactylum tricornutum). Transmembrane sequence and Tom7 motif are shaded in grey and dark grey respectively (this alignment representation is based on Fig. 2, Maćasev et al. [38]). (B) Alignment of Tom40 proteins from diverse eukaryotes. EctsiTom40 (Esi0055_0058, Ectocarpus siliculosus), PhatrTom40 (XP_002182279, Phaeodactylum tricornutum), ThapsTom40 (XP_002293745, Thalassiosira pseudonana), PhysoTom40 (Physo1_1_108992, Phytophthora sojae), PhyraTom40 (Phyra1_1_72372, Phytophthora ramorum), PhyinTom40 (EEY60531, Phytophthora infestans), MicpuTom40 (EEH60685, Micromonas pusilla), ChlreTom40 (XP_001702575, Chlamydomonas reinhardtii), ArathTom40-1 (NP_188634, Arabidopsis thaliana), ArathTom40-2 (NP_175457, Arabidopsis thaliana), PhypaTom40 (XP_001783489, Physcomitrella patens), OstluTom40 (XP_001417025, Ostreococcus lucimarinus), SacceTom40 (EDN64139, Saccharomyces cerevisiae), ToxgoTom40 (EEE23864, Toxoplasma gondii), PermaTom40 (EER18361, Perkinsus marinus), PlafaTom40 (CAG24986, Plasmodium falciparum), HydmaTom40 (XP_002159190, Hydra magnipapillata), NeucrTom40 (XP_961545, Neurospora crassa), HomsaTom40 (O96008, Homo sapiens), DicdiTom40 (EAL68763, Dictyostelium discoideum), CyameTom40 (AP006488 DNA, Cyanidioschyzon merolae), CioinTom40 (XP_002131405, Ciona intestinalis). Two predicted β-str [file pone.0019540.s001.pdf]

(a)

Tom7

```
CLUSTAL FORMAT: MUSCLE (3.7) multiple sequence alignment

                                        CYTOSOL (out)                              transmembrane      IMS (in)

EctsiTom7        ---------MGEKIMRRRPRRKSAPLLPK-----------VLAVAKPIMRWGIMPAVLLMGM-RSE----PNPTLLEV--LYPL---  Stramenopiles
PhatrTom7        -MAVPRRRQSGGLVKFGKLSSRILSMLDV---------NYIFAKTRTLLLYGFAPAVVLIGL-RTE----PRPSPWDLVNIWE----  Stramenopiles
ChlreTom7        ------MNAITGPNGSNDLAKKLEQAVDV-----------IVTYGKPIVHWGFIPAVILVGMLTTK----PRPTLGQL--LWLG---  Plantae
ArathTom7-1      MESTISLKVNKGKGKGSKGASSSDDKSKF--DVVKEWTNWSLKKAKVVTHYGFIPLVIFVGM-NSD----PKPHLFQL--LSPV---  Plantae
SoltuTom7-1      -----MLKPKGKNTKKAAAADEDDGAVAVVGKFVKEWGTWTAKKAKVITHYGFIPLVIIIGM-NSE----PKPSLSQL--LSPV---  Plantae
VitviTom7        MASRVSVKGKGKSIKGSKDGSEGHSTAKC----LKDWSNWALKKAKVITHYGFIPMVIIIGM-NSE----PKPQLYQL--LSPV---  Plantae
RiccoTom7-1      MASRVSLKSKGKSSGGAKGSKAMEEKSTI--QCLKEWSTWTLKKAKVITHYGFIPLVVIIGM-NSE----PKPQLYQL--LTPV---  Plantae
SacceTom7        ----------MSFLPSFILSDESKERISK-----------ILTLTHNVAHYGWIPFVLYLGWAHTS----NRPNFLNL--LSPLPSV  Opisthokonts
NeucrTom7        ---------------MFALSEESKERIGK-----------LIDISRVVVHYGYLPLILYLGYTRSV----PRPSIIRL--LSPLS--  Opisthokonts
ApimeTom7        ----------------MAMCPRTKQKVAI-----------ILDVSKAIFHWGFIPAILFLGFRKGADPGMPQLSIINL--LWQ----  Opisthokonts
HomsaTom7        ---------------MVKLSKEAKQRLQQ-----------LFKGSQFAIRWGFIPLVIYLGFKRGADPGMPEPTVLSL--LWG----  Opisthokonts
                                                              .    :*  * :: :*                :  :
```

(b)

Tom40

```
CLUSTAL FORMAT: MUSCLE (3.7) multiple sequence alignment

EctsiTom40      PQFPSGH------------YQGTLTVV---------GESE-T---MVRASMDAD-----CNVSMDAHAPLGMPGL-------------AGKLTVHQGKN------------DVIQGTALY
PhatrTom40      SMLPDGRTST---------YSFVTQVA----------DEA-G---FMMARWDPGQASVNGRVHRALLGGLAMGKL-------------QIGVSAE-GQN------------DTLLGEVDF
ThapsTom40      SMLPDGRTSS---------YGFTTQMN----------DEE-G---FCMARVDPERGTMDGRIHKALFGGVAMAKL-------------QVGTSSE-GSN------------DQLLGEIDL
PhyinTom40      PMIPGGL------------YQFGANVVMGDP-----MDPS-T---FLMSKITPD-GYLDARWNQKLSDNWKM-RV-------------KAQLKNE-EHG------------SQALADFDY
PhysoTom40      PMIPGGL------------YQFGANVVMGDP-----MDPS-T---FLMSKITPD-GYLDARWNQRLSGNWKM-RV-------------KSQLKNE-EHG------------SQALADFDY
PhyraTom40      PMIPGGL------------YQFGANVVMGDP-----MDPS-T---FLMSKITPD-GYLDARWNQRLSAKWKM-RV-------------KSQLKNE-EHG------------SQALADFDY
CyameTom40      QTTPG--------------YALSVNYI----------TNT-L---IAVSRMDMT-GRFNGRLFYTHTPRLIS-KLLVSRQPAEDPAAAAAMLAAA-TGGAAIQPPPPTSATTTVMYDLDY
ChlreTom40      MELQTGGRQ--IIKTPVGTYEFGATTV---------SESQNL---VLMGRLASD-GRLSGRIKYDVANWLGF-KV-------------QAQLANA-ANQ------------SQVMLDTDV
MicpuTom40      IEVPAQGNQ--IIKIPNATYEFGANVV----------DQK-Y---MLVGRALTD-GRLSGRLKYDFNDAISA----------------KIQTSKE-PGF------------SQVMMDLDY
OstluTom40      VEVPAQGQ---IVKLPTSTYEFGANVV----------DQD-Y---MLVGRILTD-GRVSGRVKYDLSDWLSL----------------KLQSTKE-PGF------------SQVMFDADF
ArathTom_2      TEVPNPTPSSEIIKIPTANYDFGAGFI----------DPK-L---YLIGRITTD-GRLNARAKFDLTDNFSV-KA-------------NALLTDE-EDK------------SQGHLVIDY
ArathTom_1      TEVPAQSPET-TIKIPTAHYEFGANYY----------DPK-L---LLIGRVMTD-GRLNARLKADLTDKLVV-KA-------------NALITNE-EHM------------SQAMFNFDY
PhypaTom40      VEVPSQGAQ--IIKVPAAHYEFGANLI----------DQRMM---MLIGRILTD-GRMSARIKYDFTDRFSV-KV-------------NAQLTNE-PHF------------SQGMFHFDY
CioinTom40      EGSS---------------YRFGSTYVGTKQ-----PSPT-EAYPVMIGEMSNE-GNLQAQFIHQVTSRFKA----------------KCIAQTLGSKL------------QSVQVGGDV
HydmaTom40      PQLTS--------------YHFGATYVGGSQ-----SNPA-EATPVILGDIDNS-GSLSAQIIHQFNKRIKG----------------KCVVQTQQKEF------------AMVQADVDY
HomsaTom40      IGESN--------------YHFGVTYVGTKQ-----LSPT-EAFPVLVGDMDNS-GSLNAQVIHQLGPGLRS----------------KMAIQTQQSKF------------VNWQVDGEY
SacceTom40      QALPK--------------YAFSALFA----------NDN-L---FAQGNIDND-LSVSGRLNYGWDKKNIS----------------KVNLQISDGQP------------TMCQLEQDY
NeucrTom40      RLNP---------------YAFAALYG--------------TNQIFAQGNLDNE-GALSTRFNYRWGDRTIT----------------KTQFSIG-GGQ------------DMAQFEHEH
DicdiTom40      PNENSRTPK----------YSNQNVYS---------KDST-----LLYGKIDSE-RRLFGRFDQGFFNNSIRVSL-------------TNMMDKSFKNN------------LAGELEFKL
PlafaTom40      TLREVGYL-----------YQFGANFT---N-----LDNS-L---LMISRINID-GSVNGRFCKKINNNIDC-KL-------------NFNTYAKNDTR------------NMYEMSLEV
ToxgoTom40      QSKEGGCS-----------YSFGPTLVIGEPDEAAQQEGQ-MPNFFGMARMNSD-GFLQARFIKAISKTFDI-KFNS-----------NSSISEDAKDK------------SMYEVSFDK
PermaTom40      SLSPNQYL-----------YQFGPTYA---------TDDGRT---VMVGRLGMD-GGVNARLMKKLFKGTDL-KI-------------NANSNLHDPQR------------NMYEFTVDQ
                                   *                            .            .
```

```
EctsiTom40      HGDTCSGQISLGT------G----PTEISYNQAVTPHLSMGGQGQFSS----AQQAVGLLYGFKY--NTPSWA--------------VLGRLIG----------GGANVVTAQYLRRVVP
PhatrTom40      GGHSWMGNLRYGS---MG-G--GVVLGCNYMQALTPALHMGGDGMYVG----ANGSFQTNYALKY--TM-PALTGEEDLPTTTPAKPATGMETAGSSTLCAQFKPGQGMASLDYKRVVTP
ThapsTom40      GAQTWTANLKYGS---MG-G--GNVFGCNYFQSVTPRLAMGGEGMYIG----ANGNMMSSYTAKY--SFNPSN-------------SGDGD-------------ERQGMLSLNYKRAITP
PhyinTom40      TGEDFTWNMKVSN----G-P----LLGVSYLQSVTQNLALGGEAYYHG----KHRKVISAYAGKW--TDRDWV--------------GMATY------------GAMGTLQLAYLRKVGH
PhysoTom40      VGEDFTWNMKISN----G-P----LLGVSYLQSVTQNLALGGEAYYHG----KHRKVISAYAGKW--SERDWV--------------GMATY------------GAMGTFQLAYLRKVGH
PhyraTom40      AGDDFTWNMKVSN----G-P----LFGVSYLQSVTQNLALGGEAYYHG----KHRKVISAYAGKW--TDRDWV--------------GMATY------------GAMGTLQLAYLRKVGH
CyameTom40      RGSDYSANLKLGT----G-G----VISLAYMQSIVPSFSMGGEGFFQL----RNKFSALTAAARF--LTTDGI--------------FSATV------------ASFGPVVASYVHRVNP
ChlreTom40      KGGDWNAQLKLGA----P-G----FVGLNYFQSLTPKLSAGGEFFYLP----SNPKSGVGLALRH--QGDKHV--------------ATCQV------------ATTGLMNMQYTHKVTE
MicpuTom40      KGLDCQAQLKLGN----G-Q----FYGVNYLQSVSDALALGGEGFYLG----GQRKSGVGFAARY--NDDKTV--------------ATGQV------------ATTGLVSLTYTTKVSE
OstluTom40      KGLDYQAQLKYGS----G-K----FYGVNYLQSVTPTLSLGGEGFYLG----TQGKSGVGFAARY--ADDKTV--------------ATGQI------------ATTGLLSLTYCTKVSE
ArathTom_2      KGSDYRTQLQLGN----N-S----VYAANYIQHVTPHLSLGGEAFWLG----QQLMSGVGYAARY--ETDKTV--------------ASGQI------------ASTGVAVMNYVHKVSE
ArathTom_1      MGSDYRAQLQLGQ----S-A----LIGATYIQSVTNHLSLGGEIFWAG----VPRKSGIGYAARY--ETDKMV--------------ASGQV------------ASTGAVVMNYVQKISD
PhypaTom40      RGKDYQTQLQLGN----N-A----FYGFNYIQSVTPTVALGGEVFWLG----HQRKSGIGFAGRY--NTDKVV--------------ATGQF------------ASTGIVSLTYVQRVSD
CioinTom40      VFNDSTLSVVCADPDLLN-G--TGMLIVHYLQAITPKLSIGSELLYQR--GAARQQAIASIAGRY--KTENWQ--------------AAGTI-------------AAGGMHASFYRKANE
HydmaTom40      RGDDFTTTCTLGNIDILN-E--SGIIVAHYLQRVTDNIDIGTELLYHY--GQGQQSAILSLAGRY--SADKWV--------------AAAQI-------------NAGGWHASYYRKGND
HomsaTom40      RGSDFTAAVTLGNPDVLV-G--SGILVAHYLQSITPCLALGGELVYHR--RPGEEGTVMSLAGKY--TLNNWL--------------ATVTL-------------GQAGMHATYYHKASD
SacceTom40      QASDFSVNVKTLNPSFSEKGEFTGVAVASFLQSVTPQLALGLETLYSRTDGSAPGDAGVSYLTRYVSKKQDWI--------------FSGQL------------QANGALIASLWRKVAP
NeucrTom40      LGDDFSASLKAINPSFLD-GGLTGIFVGDYLQAVTPRLGLGLQAVWQRQGLTQGPDTAISYFARY--KAGDWV--------------ASAQL------------QAQGALNTSFWKKLTD
DicdiTom40      PFMNFCLKADTEN----------QRGFSFLTSISKKLAIGYENSYLF--NHSQSIKQIQFFVNN--PLSTWS----------------LVV------------GNTAQIGSSYVYRQK-
PlafaTom40      NKPLYTYNFKSIW----Q-G--AWIFNTSYTQLLTKKLQAGVDLTYIA----SNCASIGSFGLRY--NHKNNV-LTMQIVRQPNFKSPEFML------------NQTHLYKIQYAKKISD
ToxgoTom40      MGSDWAANLKLAW----Q-G--TWILNGLFSQVITPKLQLGGELTWVA----ATGISMGSVGARY--GFNENNTVTCQIGVGPDFSSPMGFA------------NDVYSTKAQYVRKVTD
PermaTom40      QGEDWAASAKLAY----Q-G--TWISNVSFAQEVTNNLSLGGELTHVA----VNGVSIGGLGARY-----VWGPNYLSFALGRQPDFKQGPQ------------ANVHSAKMQYVRKVSD
                                                             :   :   .  * :                     .

EctsiTom40      GRVTLGAEYQ---------A---------QLGAGSQMMVGAEFQLKQSK--MSASVDSNG KVDSTLELKCGTFPTLPVTLTISSSLDHSEDKQTFGLALTCGQ-----------------
PhatrTom40      NRVTLASSLEF--------S---------PLSLESQLLVAAEFKLSRST--LNLCVDGSG HLQSVLEAKLGMGQGSP-TINFSADMDHAKQQMRFGYGIKIEG-----------------
ThapsTom40      SRVNIGASLEC--------S---------PATLESQVMVGAEFNLTRSK--MNVCVDGTG RIQSTLEAKLGREQGSP-SLNFAAELDHGKSLMRFGYGLNIG------------------
PhyinTom40      -RIRYGSELVY--------N---------HASGEAQTTCGVEMDLGQTR--FVSSVDSTF RVATSIESRVLPNF----VLTLSAEGFPLKDDFKFGYGAQLSF-----------------
PhysoTom40      -RIRYGSELVY--------N---------HASGEAQTTCGVEMDLGQTR--FVSSVDSTF RVATSIESRVLPNF----ALTLSAEGFPLKDDFKFGYGAQLSF-----------------
PhyraTom40      -RIRYGSELVY--------N---------HASGEAQTTCGVEMDLGQTR--FVSSVDSTF RVATSIESRVLPNF----VLTLSAEGFPLKDDFKFGYGAQLSF-----------------
CyameTom40      -RVSLAAELFW--------D***-------------------------------------------------------------------------------------------------
ChlreTom40      -KVTLAADFMW--------H---------LLSRDVTATVGYDVVLRQCR--IRGKADTNG VITTLLEERFSPGI----NFVLSAEMDHWQSNYKFGFGIVAGE-----------------
MicpuTom40      -KAMLASDFMW--------N---------WNARQAQASVGYDYILRQSR--LRGRIDNTG VVSAFLEERLNAGF----TLTFSAEIDHANKNHKFGFGMTVGE-----------------
OstluTom40      -KAMLASDFLW--------N---------WNQRSAQASVGYDYILRQCR--IRGRIDNHG VVSTYLEERLNVGL----NLILSAEVDHFNKNHKFGFGMTVGE-----------------
ArathTom_2      -KLSFATDFIY--------N---------YLSRDVTASVGYDLITRQSR--LRGKVDSNG VVAAYLEEQLPIGL----RFLLSAEVDHVKKDYKFGFGVNAF------------------
ArathTom_1      -KVSLATDFMY--------N---------YFSRDVTASVGYDYMLRQAR--VRGKIDSNG VASALLEERLSMGL----NFLLSAELDHKKKDYKFGFGLTVG------------------
PhypaTom40      -KVSLASDFLY--------N---------WNSKEATTSVGYDYLLRQCR--LRGRLDTNG CIAAYLEERLNLGV----NFLLSAEIDHWKKDYKFGFGMTVGE-----------------
CioinTom40      -NVQVGVELEA--------S---------LKNKESVTTFAYQMDLPKMNLLFKGMLTSEW TIGSALEKRLQPLPI---TLNLTGTYNIKKDKVAVGIGAVLG------------------
HydmaTom40      -NVQVGVDYEY--------N---------SRMQDSNVSLGYQIDIPKANVTFRGMVDTNW AVGGLFEKKLYPLPF---SFILSGKLDHLKNQSRFGFGLQIG------------------
HomsaTom40      -QLQVGVEFEA--------S---------TRMQDTSVSFGYQLDLPKANLLFKGSVDSNW IVGATLEKKLPPLPL---TLALGAFLNHRKNKFQCGFGLTIG------------------
SacceTom40      -NVEAGIETTLQAGMVPITDPLMGTPIGIQPTVEGSTTIGAKYEYRQSV--YRGTLDSNG KVACFLERKVLPTL----SVLFCGEIDHFKNDTKIGCGLQFETAGNQELLMLQQGLDADG
NeucrTom40      -RVQAGVDMTL--------SVAPSQSMMGGLTKEGITTFGAKYDFRMST--FRAQIDSKG KLSCLLEKRLGAAPV---TLTFAADVDHVTQQAKLGMSVSIEASDVDLQEQQEGAQSLNI
DicdiTom40      -NLHIGTDLVM--------G------ISQEGKFMSEYSFGVRYAFQQSL--VKFRADSHG SIFGSYDQMINSFT----KLNLSGSLNYFAQDYKFGLGLTFQK-----------------
PlafaTom40      -RLSLGTELEI--------T---------PQTKESAMRLGWDYSFRHAK--VQGSIDTSG KISVFTQDYSGFG--------VSGYIDYLNNDYKFGFMMHISPSQEQPQ------TAA--
ToxgoTom40      -RLSMGTELEF--------T---------HPDMSSAMRVGWQYLFRQAR--VQGLVDTAG RVSMFAQDYNGFG--------LSGMIDYWHGDYKFGFQMNVVPPPPQAE----QPPPM--
PermaTom40      -RLAIGSEFEY--------S---------QPEQESQLRLGYEYTFRHAR--VQGLLDTCG KVSCFVQDFMGFG--------ISGMVDFVKGDYRFGFMMHMVPQPEDAAAAGGAPTSA--
                 .   . .                                                                                  PoxGxxΦxΦ
```

(c)

Tom22

```
CLUSTAL FORMAT: MUSCLE (3.7) multiple sequence alignment

                                                                                         CYTOSOL (out)                                   transmembrane
EctsiTom22      ----------------------------------------------MAK--KLTFNDEKRDNGPGVLQ--TLVVTGRSLGVA-----GFGWAQWAAKKTGRTGWVLLTTAVVTLVPLVFE
PhatrTom22      --------------------------------------------MTKEP--KSSGGLKGLLQRTGKFFYTTGVYARDKGSTA---------FKWGYQVGGQVAFAVATTSMVMLMPLLFE
PhyinTom22      --------------------------------MSAIGALRELEKDKKGP--KNAGIKELFLNLPSRIRQSGPVKGGINFAVK------------SWKTSGRWLWVLSTTLLVTLVPLSIE
ArathTom22_1    ----------------------------------------------MAP--KKIGAGKGDSSILAKISNYDIVSQGRRAACD-----AVYVSKKLLKSTGKAAWIAGTTFLILAVPLILE
ArathTom22_2    ---------------------------------------------MAAKRIGAGKSGGGDPNILARISNSEIVSQGRRAAGD-----AVEVSKKLLRSTGKAAWIAGTTFLILVVPLIIE
OstluTom22      ---------------------------------------------MEIT--PDYGAEEAKSAKPVRLGWADASRLTRKLAVH----------------TGKAAWTFGTSFLVLIVPLIVQ
MicpuTom22      ---------------------------------------------MEIT--P--APDATCNSQMSSLG--DSARVGKKLIWH----------------TGKAAWVFGTSFLLLVVPLIVQ
SacceTom22      MVELTEIKDDVVQLDEPQFSRNQAIVEEKASATNNDVVDDEDDSDSDFE--DEFDENETLLDRIVALK--DIVPPGKRQTISNFFGFTSSFVRNAFTKSGNLAWTLTTTALLLGVPLSLS
NeucrTom22      -------------MVQLTEVEDEHFQQPQVGPEEDDEDFTDTDSEISVD--SDYESQETFTDRLYALR--DMVSPTTRGWFYHKYSTTTNFVKSTLSFAGRAAWAVSVSGLLIGVPFAIA
HomsaTom22      ----------------MAAAVAAAGAGEPQSPDELLPKGDAEKPEEELE--EDDDEELDETLSERLWGLTEMFPERVRSAAGATFDLSLFVAQKMYRFSRAALWIGTTSFMILVLPVVFE
DromeTom22      ----------MDSDPEIEFIEKDSGMSSLGGSKDETPERRAVAATSNDPQRENYDDEPDETASERFWGLTEMFPEPVRNAVGAVSSATVKSVKGFYSFSCNASWIFFTSAVILFAPVIFE
                                                                                                        :   .: ::   *. .

                          IMS (in)
EctsiTom22      ITREAQLIEQEKI-----HINALLAEGKTRQEIAQMGLYSAL--DPNVMGPEAS--    stramenopiles
PhatrTom22      IAREGQMLESERA-----QVKDYKGRGYSDRQLQELGFSEAALHTPSVASLNKK--    stramenopiles
PhyinTom22      MLREEQTNEVVK---------ELVSKGFSYNQIQGMGYMVSQPQSTLSTPAEGATQ    stramenopiles
ArathTom22_1    LEQDHRLGEIDF------EQASLLG-TP-----------------PVGAML-----    Plantae
ArathTom22_2    MDREAQINEIEL------QQASLLGAPPS----------------PMQRGL-----    Plantae
OstluTom22      LHREEQLIELEK------EQLGVLN---------------------QPTGLQAE--    Plantae
MicpuTom22      LHREEQMAAAEQ------EQLGVLNLGSS-----------SLMTNAVPVQ------    Plantae
SacceTom22      ILAEQQLIEMEKTFDLQSDANNILAQGEK---------------DAAATAN-----    Opisthokonts
NeucrTom22      FAEDQNYAAMEQEARMRELGSDVLTAGGEGQAGTAEKTLAAIGGEGARPAL-----    Opisthokonts
HomsaTom22      TEK-LQMEQQQQLQ----QRQILLGPNTG---------LSGGMPGALPSLPGKI--    Opisthokonts
DromeTom22      TER-AQMEELHKSQ----QKQVLLGPGSA-----------MGPGGPSPSLPLIR--    Opisthokonts
                     .
```

(d)

Mia40

```
ThapsMia40      SIETVDR----------EDCPLC-KKFGSGP-CGKVFQQWLSCTDANPGKDESGEPLHLTKCAIFAEKLAVCIDTN
PhyraMia40      DATTGECLAPK------AECGFC-KFMKAGP-CGREFSAWEACLDRCKKSGDD----FIDTCGPQTLALRDCVDAN
PhysoMia40      DTTAGECLAPK------AECGFC-KFMKAGP-CGKEFTAWEACLDRCKKSGDD----FIEKCGPQTLALRDCVDAN
PhatrMia40      -----------------DSCPLC-RQFRQGP-CAAPFNSWYACTERANAASQD----HVAVCSESFAAFHACVASN
DicdiMia40      -----------------ADCPPCIRRMANSQYCGDELVKSYLCFQESNKNGTS-----VESCAGSFNKLKDCMVKY
OstluMia40      -----------------LAC-PCVADLRQSS-CGTSFDEALTCFMLAKDEEKG------KKCVEEFVSLHACMVKN
MicpuMia40      DAIKAA-----------MEC-PCVADLKNSA-CGEAFAGALGCFMSADAEERG------SKCVKEFVAMHACMVEN
ArathMia40      GEDDNENESLEAKAQRALDC-PCIADLRNGS-CGSQFSEAFLCFLKSTAEEKG------SDCVNPFVALQSCINAN
PhypaMia40      -----------------LKC-DCVAELRDGP-CGSQFKEAFYCFLTSTAEEQG------SDCTKSFIAMQTCMTAN
CyameMia40      -----------------LNC-PCVESLKEGS-CGGAFIAAYRCFLESEAEPRG------SDCYEVFQRMQDCMLAH
HomsaMia40      N----------------WNC-PCLGGMASGP-CGEQFKSAFSCFHYSTEEIKG------SDCVDQFRAMQECMQKY
SacceMia40      EEVQHEGAYNPDTGEINWDC-PCLGGMAHGP-CGEEFKSAFSCFVYSEAEPKG------IDCVEKFQHMQDCFRKY
                ------------------CXXC---------C---X9----C------------------C---X9----C----
```

(e)

Erv1

CLUSTAL FORMAT: MUSCLE (3.7) multiple sequence alignment

```
EctsiErv1       --------------MPSSRRRSKDPADCEDPACADMADLLRKGRALAAKNKQKAASNTATDGKAAGSQAQPSSSAAETDEHAA-------------SSSRNDGCPLDKGELGAATWGLIH
PhyraErv1       ------------------MVAAKSDPNCVEPACADKMDFFKSSMG-------KRVTTPAMQPK---------------------------------------DCPLDRQELGNATWGLLH
PhyinErv1       ------------------MVATKSDPNCVEPACADKMDFFKSSMG-------KKTKKTATQPKPAT------------------------------------DCPLDRQELGNATWGLLH
PhysoErv1       ------------------MVATKSDPNCVEPACADKMDFFKSSMG-------KKATKPAAQPKPAT------------------------------------DCPLDRQELGNATWGLLH
ThapsErv1       ----------------------------------------------------------------------------------------------------***CPPTRDEIGVSTWSLLH
PhatrErv1       -----------------------------------------------------------------------------------------------------***PPSSASLGNSSWTLLH
CyameErv1       -----------------------------------------------------------------------------------------------------***PPTRAELGRAGWTLLH
ChlreErv1       -----MPNAAAAVSEAAATPAARKLSDCKSRACS---------------------------------------------------------------------CPPDTWELGRATWTFLH
ArathErv1       ---------MGEKPWQPLLQSFEKLSNCVQTHLSNFIGIKNTPPSSQSTIQNPIISLDSSPPIATNSSSLQKLPLKDKSTG-----------------------PVTKEDLGRATWTFLH
HomsaALR        ----------MAAPGERGRFHGGNLFFLPGGARSEMMDDLATDAGPGRGAERRGRLGLDASPGADLRFSCRRGRLPEAACRACVDFKTWMRTQQKRDTKFREDCPPDREELGRHSWAVLH
SacceErv1       MKAIDKMTDNPPQEGLSGRKIIYDEDGKPCRSCNTLLDFQYVTGKISNGLKNLSSNGKLAGTGALTGEASELMPGSRTYRKVD---------------------PPDVEQLGRSSWTLLH
                                                                                                                        *    .:*   * .:*

EctsiErv1       TTAAHYPEKPSKETQDQARALVTGLAGLYPCTYCRKDFREEVRKLPPDVSSRVALSLWACQQHNLVNEKIGKPTFGCT-LPALDERWKKGKPSCW--EGGAEGV---------------
PhyraErv1       SMGIYYPDKPSAEYQAKTKTFIEALALMYPCVHCADDFQKEIAKSPPCVESRTTFSMWLCEQHNIVNRKIHKPVFECT-MEKLEERWRKGKPSCWGEDGDEDSAKESLG----------
PhyinErv1       SMGIYYPDKPSPEYQAKAKTFIEALALMYPCVHCAEDFQKEVAISPPRVESRTTFSMWLCEQHNIVNRKIHKPVFECT-MEKLEERWRKGKPACWGEDGDEDSAQDSLH----------
PhysoErv1       SMGIYYPDKPSPEYQAKAKTFIEALALMYPCVHCADDFQKEIAKSPPRVESRTTFSMWLCEQHNIVNRKIHKPLFECT-MEKLEERWRKGKPSCWGEDGDEESAQDALG----------
ThapsErv1       SMAAWYPNQPSSQDEQFMSDFMKALARFYPCTWCASDFQRNIELSPPKTETREDLCIWICEQHNIVNEKLGKPLFQCT-MDKLDERWKK---------SSDPKCQK-------------
PhatrErv1       TMAAWYPDKPTTEDRSYITGFMNALARFYPCPWCAKDFRHNIEEKPVQTSSREALCTWLCEQHNIVNQKLGKPQYACD-IQTLDERWRK---------SSKDACQSGSH----------
CyameErv1       SIAANYPEVATPEMQTHARQFIASFAALYPCPTCREHFQGYVRTHPPALESREQFVKWCCRAHNAVNLRLGKPTIPCTDLQLLDKRWRDCH-------CDEQEPQNMIRAARRLIESRR
ChlreErv1       SVAAGYPESPSERQQGLMRGMVEGLAEFYPCEVCREHLREQVAARPPQVGSARELNMWLCGLHNEVNEMLGKPLFDCA---RVGERWRE---------GPADGSCD-------------
ArathErv1       TLAAQYPEKPTRQQKKDVKELMTILSRMYPCRECADHFKEILRSNPAQAGSQEEFSQWLCHVHNTVNRSLGKLVFPCE---RVDARWGKL--------ECEQKSCDLHGTSMDF-----
HomsaALR        TLAAYYPDLPTPEQQQDMAQFIHLFSKFYPCEECAEDLRKRLCRNHPDTRTRACFTQWLCHLHNEVNRKLGKPDFDCS---KVDERWRD---------GWKDGSCD-------------
SacceErv1       SVAASYPAQPTDQQKGEMKQFLNIFSHIYPCNWCAKDFEKYIRENAPQVESREELGRWMCEAHNKVNKKLRKPKFDCN---FWEKRWKD---------GWDE-----------------
                : .  **  .:         ::  :: :***  * . :   :         :   :  * *   ** **  : *    *        ** .
                                            YPCXXC                          ______CX16C______
```

*** : incomplete sequences

(f)

Hot13

Panel 1

CLUSTAL FORMAT: MUSCLE (3.7) multiple sequence alignment

```
EctsiHot13      --------------------MLVPGQVSIQFPCCKRWYECPECHDERENHPQRI-----S TKNVAFACKTCRKIFVKDLSIYGTEDSA--CPHCETVFV----------LPAVTPEAKV-
PhyinHot13      -----------------MCKHIPNAQVSFQTPCCNRWFDCGECHFELSDHRQQP------ AAEMAFVCKQCRNPFRKDLTAFDEEDES--CPHCGNGLV----------QPVADLRA---
PhyraHot13      -----------------MCKHIPNAQVSFRAPCCNRWFDCSECHFELSDHRQQA------ ADEMAFVCKQCRNPFRKDLTAFDEEDEN--CPHCGNELI----------QSA--------
PhysoHot13      -----------------MCKHIPNAQVSFRAPCCSRWFDCSECHFELSDHRQQA------ AAEMAFVCKQCRNPFRKDLTAFDEEDES--CPHCGNMLV---------------------
CyameHot13      ----------------------------***PCCQVYYTCRLCHDEVNDHVMDR------ YAVERILCMRC----------LTEQAPAEVCSSCGERFXXXXXXXXNVSEPCTTFRIHVK
SacceHot13      MIETAIYGKTVDDQSRCVHWHLPKDVIAIRFKCCDKYYACFECHQELSSHPLEKYDLLDD ANKHLIICGVCRHEM--TFAEYYDYNSNLICPNCRSPFN---------------PGCKLH
NeucrHot13      -----------------MCKHVLNAQVSIRSPCCRKWFDCPECHAESESHPLKK------ TLEMTFACKKCKKCFRKDATEFEDSDEY--CPHCDNHYI----------IEAVTPKAALR
                                 **  :: *  ** * ..*                : *  *                   *. *

EctsiHot13      -----AQLALSILAAEVSAEVSKF-----------------
PhyinHot13      -----ATPAEANHRNELVVERAMP-----------------
PhyraHot13      -----------------------------------------
PhysoHot13      -----------------------------------------
CyameHot13      SVYHTSDLSLKHCHSRFL-----------------------
SacceHot13      YHLYFQNPPPAMC----------------------------
NeucrHot13      VEGEDARMDARMLKDERVKQTKKTMNQISIFDPDVDADKLG
```

Panel 2

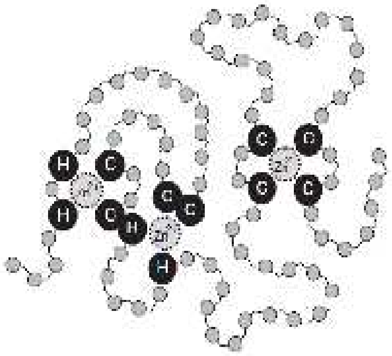

Model of zinc-ion coordination by conserved Cys and His residues within SacceHot13
(Mesecke et al., 2008)

(g)

Small Tims

Panel 1

```
EctsiTim9        -------MTPVQQQDFLQH-------------------------------------LESQ
EctsiTim13       ---MDGQASAEQQQAIIQQ-------------------------------------LQEQ
EctsiTim8        MSWFRGKKEPEPESTPEPTFSSDTSAFEGSTNFASGPPSRGGGGATSAGMGDLQAAMQIE
EctsiTim10       MSWF-GNSEPEPPSGPSPM-------------------------------------DLAK


EctsiTim9        QRKDSL-AMYNNLVFRCFDECSKSFRSKR-LDDGETKCINVCAEKFIKLTSRVALRFQDI
EctsiTim13       VQAKALQELMTQMTDQCFNRCAKTSSGDR-INSSEQGCLAMCMDRYMDT-------MGLV
EctsiTim8        QQKAQMQAIVSRLTDLAFTKCIQKPSSS--LSSSEQSCINATVLKYFDTSEFV---LGRL
EctsiTim10       QEVDMYSDLFTKMSGLCFKKCVVKMHGESDLNVGEMSCVDRCVSKYMEAQEKVGVILKRA
                                 CFxxC                CxxxC

EctsiTim9        QQQKAKDEAA-------GVQR----
EctsiTim13       NKAMVAKANR---------------
EctsiTim8        MKSQQGGGDI---------------
EctsiTim10       EESLAAQQGGVGGPAAGGVPGVPGR
```

Panel 2

```
SacceTim9        NLVERCFTD
NeucrTim9        NLVDNCFTA
OrysaTim9        SLVERCFTD
HomsaTim9        KLTETCFLD
DromeTim9        KLSETCFTD
EctsiTim9        NLVFRCFDE
                 .*. .**.
```

(h)

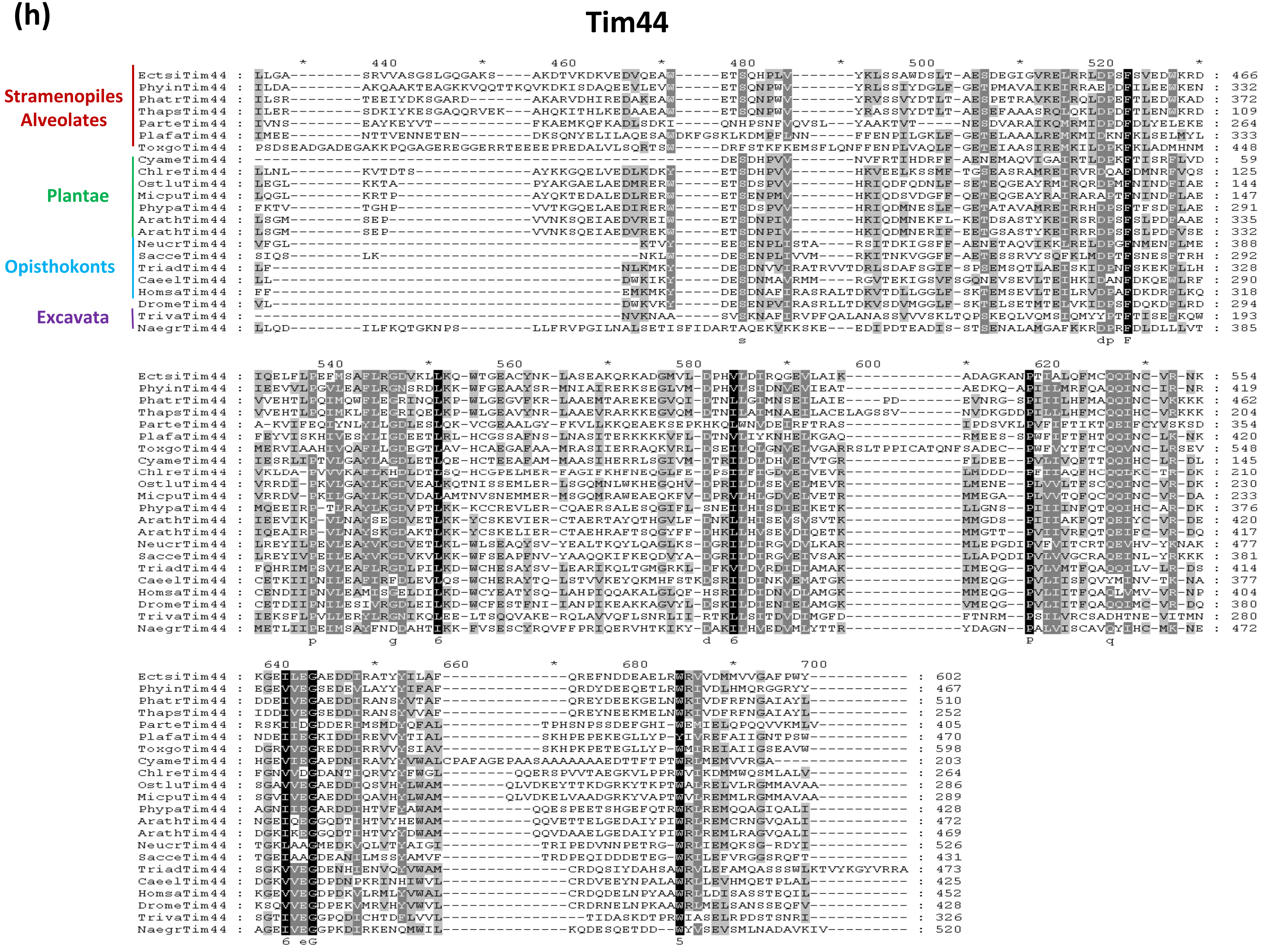
Tim44
Stramenopiles
Alveolates
Plantae
Opisthokonts
Excavata
EctsiTim44
PhyinTim44
PhatrTim44
ThapsTim44
ParteTim44
PlafaTim44
ToxgoTim44
CyameTim44
ChlreTim44
OstluTim44
MicpuTim44
PhypaTim44
ArathTim44
ArathTim44
NeucrTim44
SacceTim44
TriadTim44
CaeelTim44
HomsaTim44
DromeTim44
TrivaTim44
NaegrTim44

(i)

Tim23

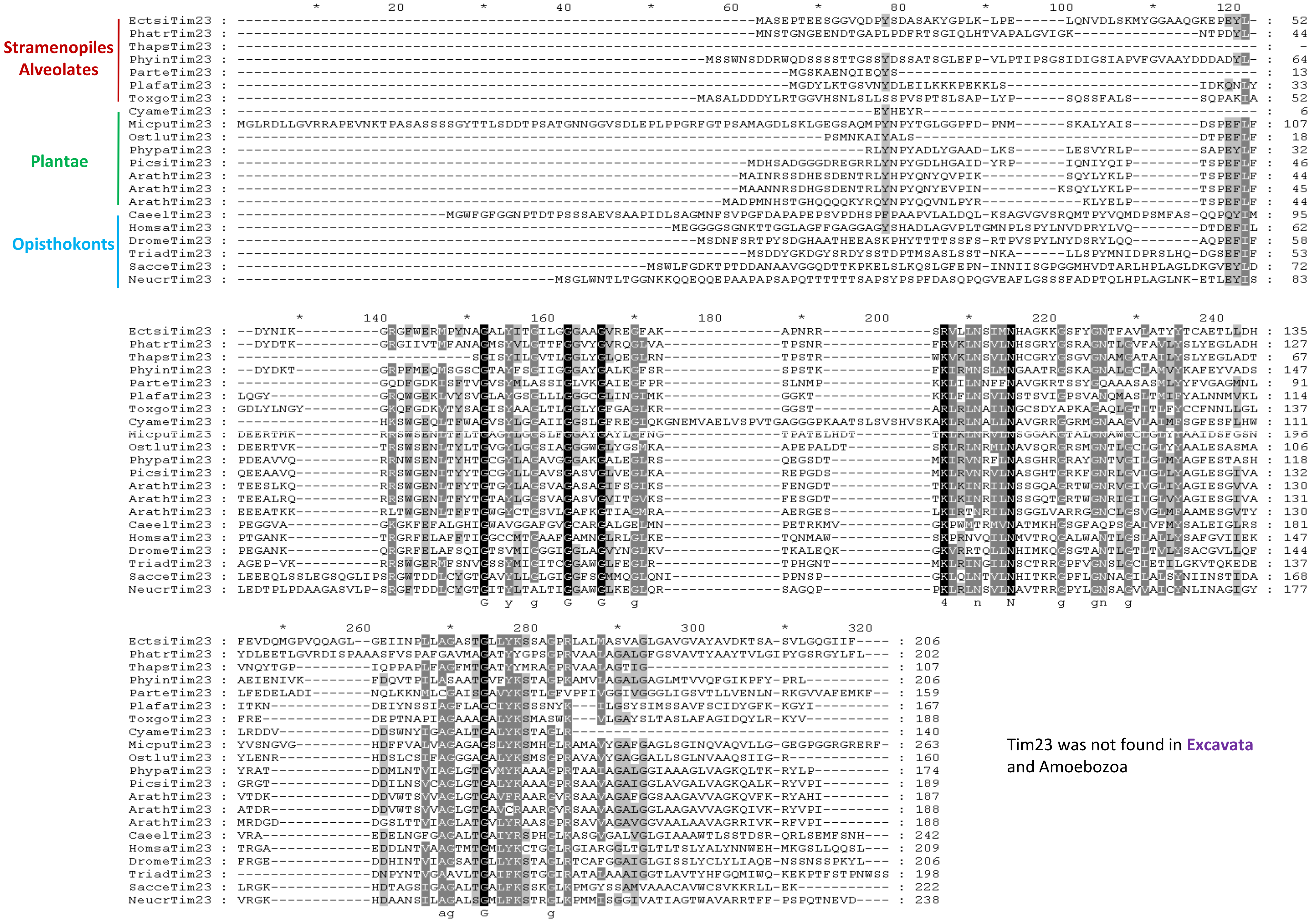

Tim23 was not found in Excavata and Amoebozoa

(j)

Tim17

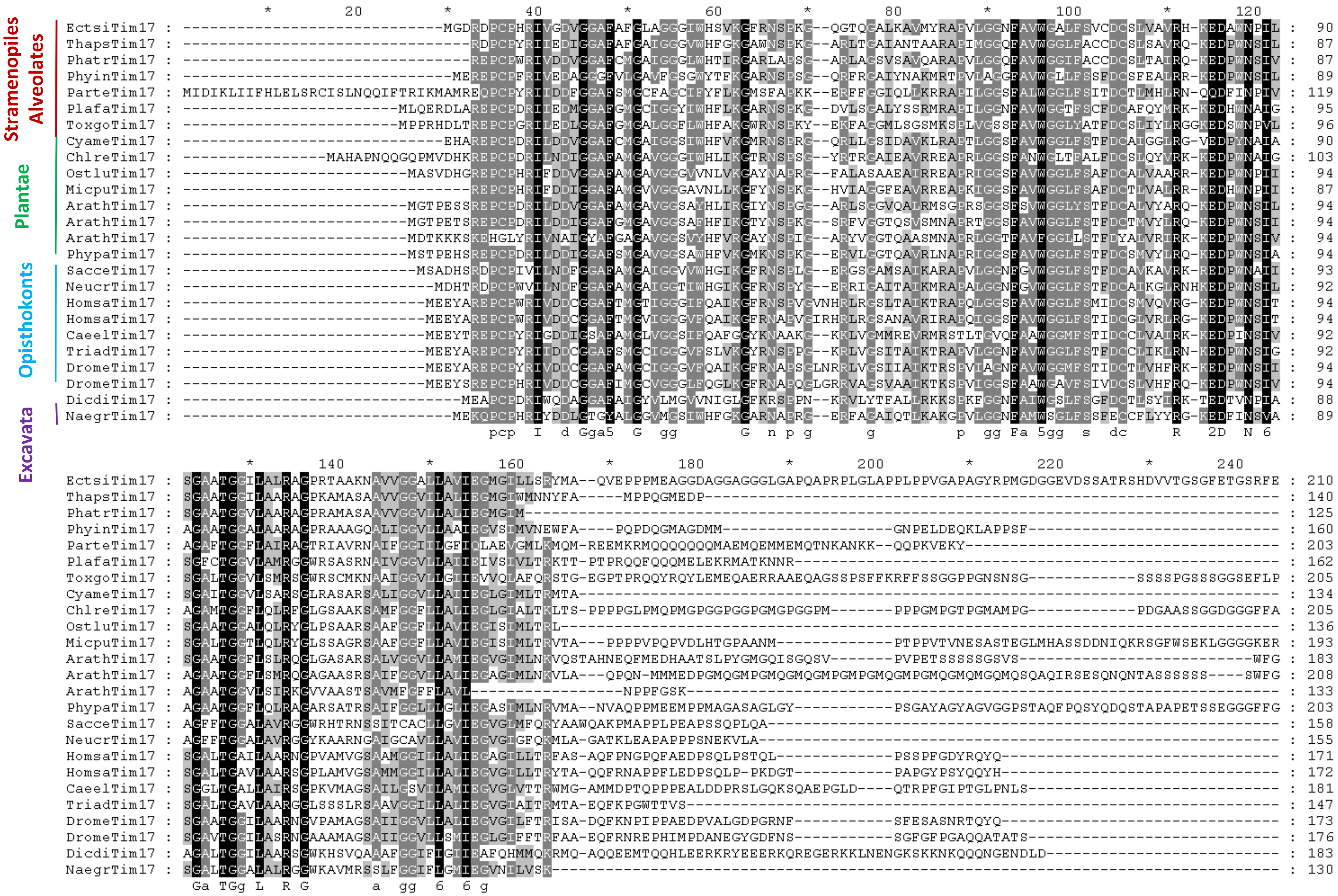

Stramenopiles
Alveolates
Plantae
Opisthokonts
Excavata
EctsiTim17
ThapsTim17
PhatrTim17
PhyinTim17
ParteTim17
PlafaTim17
ToxgoTim17
CyameTim17
ChlreTim17
OstluTim17
MicpuTim17
ArathTim17
ArathTim17
ArathTim17
PhypaTim17
SacceTim17
NeucrTim17
HomsaTim17
HomsaTim17
CaeelTim17
TriadTim17
DromeTim17
DromeTim17
DicdiTim17
NaegrTim17

(k)

Tim16

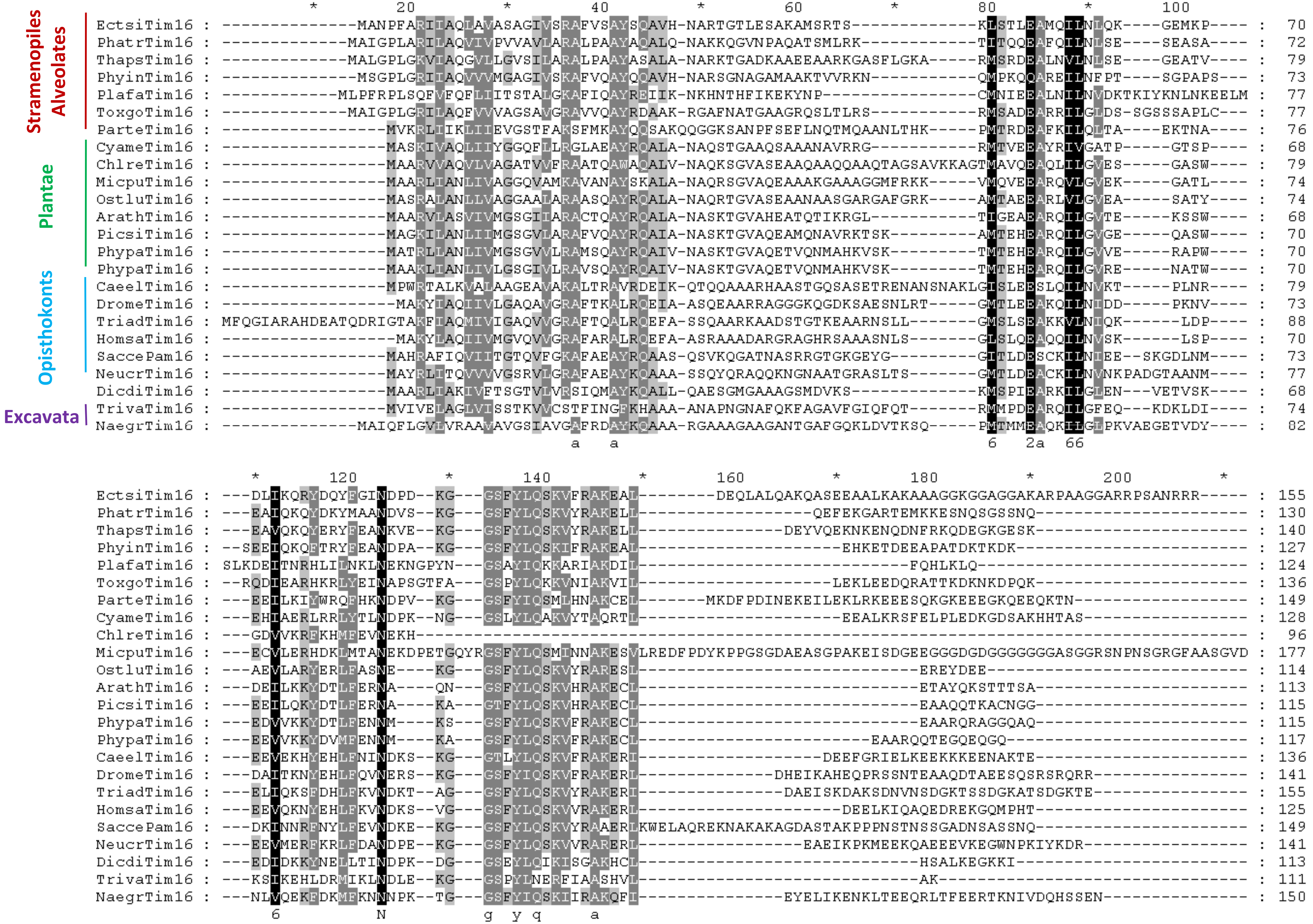

Stramenopiles
Alveolates
Plantae
Opisthokonts
Excavata
* 20 * 40 * 60 * 80 * 100
EctsiTim16 : --------------MANPFARIIAQLAVASAGIVSRAFVSAYSQAVH-NARTGTLESAKAMSRTS-------------KLSTLEAMQIINLQK----GEMKP---- : 70
PhatrTim16 : -------------MAIGPLARILAQVIVPVVAVLARALPAAYAQALQ-NAKKQGVNPAQATSMLRK------------TITQQEAFQIINLSE----SEASA---- : 72
ThapsTim16 : -------------MALGPLGKVIAQGVLLGVSILARALPAAYASALA-NARKTGADKAAEEAARKGASFLGKA-----RMSRDEALNVLNLSE----GEATV---- : 79
PhyinTim16 : --------------MSGPLGRIIAQVVVMGAGIVSKAFVQAYQQAVH-NARSGNAGAMAAKTVVRKN-----------QMPKQQAREIINFPT----SGPAPS--- : 73
PlafaTim16 : ------------MLPFRPLSQFVFQFLIITSTALGKAFIQAYREIIK-NKHNTHFIKEKYNP----------------CMNIEEALNIINVDKTKIYKNLNKEELM : 77
ToxgoTim16 : -------------MAIGPLGRILAQFVVVAGSAVGRAVVQAYRDAAK-RGAFNATGAAGRQSLTLRS-----------RMSADEARRILGLDS-SGSSSAPLC--- : 77
ParteTim16 : -----------------MVKRLIIKLIIEVGSTFAKSFMKAYQQSAKQQGGKSANPFSEFLNQTMQAANLTHK-----PMTRDEAFKILQLTA----EKTNA---- : 76
CyameTim16 : -----------------MASKIVAQLIIYGGQFLLRGLAEAYRQALA-NAQSTGAAQSAAANAVRRG-----------RMTVEEAYRIVGATP-----GTSP---- : 68
ChlreTim16 : -----------------MAARVVAQVLVAGATVVFRAATQAWAQALV-NAQKSGVASEAAQAAQQAAQTAGSAVKKAGTMAVQEAQLILGVES-----GASW---- : 79
MicpuTim16 : -----------------MAARLIANLIVAGGQVAMKAVANAYSKALA-NAQRSGVAQEAAAKGAAAGGMFRKK-----VMQVEEARQVLGVEK-----GATL---- : 74
OstluTim16 : -----------------MASRALANLLVAGGAALARAASQAYRQALA-NAQRTGVASEAANAASGARGAFGRK-----AMTAEEARLVLGVEA-----SATY---- : 74
ArathTim16 : -----------------MAARVLASVIVMGSGIIARACTQAYRQALA-NASKTGVAHEATQTIKRGL-----------TIGEAEARQILGVTE-----KSSW---- : 68
PicsiTim16 : -----------------MAGKILANLIIMGSGVLARAFVQAYRQAIA-NASKTGVAQEAMQNAVRKTSK---------AMTEHEARQILGVGE-----QASW---- : 70
PhypaTim16 : -----------------MATRLLANLIVMGSGVVLRAMSQAYRQAIV-NASKTGVAQETVQNMAHKVSK---------TMTEHEARQILGVVE-----RAPW---- : 70
PhypaTim16 : -----------------MAAKLIANLIVLGSGIVLRAVSQAYRQAIV-NASKTGVAQETVQNMAHKVSK---------TMTEHEARQILGVRE-----NATW---- : 70
CaeelTim16 : -----------------MPWRTALKVALAAGEAVAKALTRAVRDEIK-QTQQAAARHAASTGQSASETRENANSNAKLGISLEESLQILNVKT-----PLNR---- : 79
DromeTim16 : ------------------MAKYIAQIIVLGAQAVGRAFTKALRQEIA-ASQEAARRAGGGKQGDKSAESNLRT-----GMTLEEAKQILNIDD-----PKNV---- : 73
TriadTim16 : MFQGIARAHDEATQDRIGTAKFIAQMIVIGAQVVGRAFTQALRQEFA-SSQAARKAADSTGTKEAARNSLL-------GMSLSEAKKVLNIQK------LDP---- : 88
HomsaTim16 : ------------------MAKYLAQIIVMGVQVVGRAFARALRQEFA-ASRAAADARGRAGHRSAAASNLS-------GLSLQEAQQILNVSK------LSP---- : 70
SaccePam16 : -----------------MAHRAFIQVIITGTQVFGKAFAEAYRQAAS-QSVKQGATNASRRGTGKGEYG---------GITLDESCKIILNIEE--SKGDLNM---- : 73
NeucrTim16 : -----------------MAYRLITQVVVVGSRVLGRAFAEAYKQAAA-SSQYQRAQQKNGNAATGRASLTS-------GMTLDEACKILNVNKPADGTAANM---- : 77
DicdiTim16 : -----------------MAARLIAKIVFTSGTVLVRSIQMAYKQALL-QAESGMGAAAGSMDVKS-------------KMSPIEARKILGLEN---VETVSK---- : 68
TrivaTim16 : -----------------MVIVELAGLVISSTKVVCSTFINGFKHAAA-ANAPNGNAFQKFAGAVFGIQFQT-------RMMPDEARQILGFEQ---KDKLDI---- : 74
NaegrTim16 : --------------MAIQFLGVLVRAAVAVGSIAVGAFRDAYKQAAA-RGAAAGAAGANTGAFGQKLDVTKSQ-----PMTMMEAQKILGLPKVAEGETVDY---- : 82
a a 6 2a 66
* 120 * 140 * 160 * 180 * 200 *
EctsiTim16 : ---DLIKQRYDQYFGINDPD--KG---GSFYLQSKVFRAKEAL--------DEQLALQAKQASEEAALKAKAAAGGKGGAGGAKARPAAGGARRPSANRRR----- : 155
PhatrTim16 : ---EAIQKQYDKYMAANDVS--KG---GSFYLQSKVYRAKELL------------------QEFEKGARTEMKKESNQSGSSNQ---------------------- : 130
ThapsTim16 : ---EAVQKQYERYFEANKVE--KG---GSFYLQSKVYRAKELL---------------DEYVQEKNKENQDNFRKQDEGKGESK---------------------- : 140
PhyinTim16 : --SEEIQKQFTRYFEANDPA--KG---GSFYLQSKIFRAKEAL---------------------EHKETDEEAPATDKTKDK------------------------ : 127
PlafaTim16 : SLKDEITNRHLILNKLNEKNGPYN---GSAYIQKKARIAKDIL--------------------------FQHLKLQ---------------------------- : 124
ToxgoTim16 : --RQDIEARHKRLYEINAPSGTFA---GSPYLQKKVNIAKVIL---------------------LEKLEEDQRATTKDKNKDPQK--------------------- : 136
ParteTim16 : ---EEILKIYWRQFHKNDPV--KG---GSFYIQSMLHNAKCEL-------MKDFPDINEKEILEKLRKEEESQKGKEEEGKQEEQKTN------------------ : 149
CyameTim16 : ---EHIAERLRRLYTLNDPK--NG---GSLYLQAKVYTAQRTL---------------------EEALKRSFELPLEDKGDSAKHHTAS----------------- : 128
ChlreTim16 : ---GDVVKRFKHMFEVNEKH-- : 96
MicpuTim16 : ---ECVLERHDKLMTANEKDPETGQYRGSFYLQSMINNAKESVLREDFPDYKPPGSGDAEASGPAKEISDGEEGGGDGDGGGGGGGASGGRSNPNSGRGFAASGVD : 177
OstluTim16 : ---AEVLARYERLFASNE----KG---GSFYLQSKVYRARESL----------------------------EREYDEE-------------------------- : 114
ArathTim16 : ---DEILKKYDTLFERNA----QN---GSFYLQSKVHRAKECL----------------------------ETAYQKSTTTSA---------------------- : 113
PicsiTim16 : ---EEILQKYDTLFERNA----KA---GTFYLQSKVHRAKECL----------------------------EAAQQTKACNGG--------------------- : 115
PhypaTim16 : ---EDVVKKYDTLFENNM----KS---GSFYLQSKVFRAKECL----------------------------EAARQRAGGQAQ--------------------- : 115
PhypaTim16 : ---EEVVKKYDVMFENNM----KA---GSFYLQSKVFRAKECL------------------------EAARQQTEGQEQGQ----------------------- : 117
CaeelTim16 : ---EEVEKHYEHLFNINDKS--KG---GTLYLQSKVFRAKERI--------------------DEEFGRIELKEEKKKEENAKTE--------------------- : 136
DromeTim16 : ---DAITKNYEHLFQVNERS--KG---GSFYIQSKVFRAKERL---------------DHEIKAHEQPRSSNTEAAQDTAEESQSRSRQRR--------------- : 141
TriadTim16 : ---ELIQKSFDHLFKVNDKT--AG---GSFYLQSKVYRAKERI---------------DAEISKDAKSDNVNSDGKTSSDGKATSDGKTE---------------- : 155
HomsaTim16 : ---EEVQKNYEHLFKVNDKS--VG---GSFYLQSKVVRAKERL---------------------DEELKIQAQEDREKGQMPHT---------------------- : 125
SaccePam16 : ---DKINNRFNYLFEVNDKE--KG---GSFYLQSKVYRAAERLKWELAQREKNAKAKAGDASTAKPPPNSTNSSGADNSASSNQ---------------------- : 149
NeucrTim16 : ---EEVMERFKRLFDANDPE--KG---GSFYLQSKVVRARERL-----------------EAEIKPKMEEKQAEEEVKEGWNPKIYKDR----------------- : 141
DicdiTim16 : ---EDIDKKYNELLTINDPK--DG---GSEYLQIKISGAKHCL----------------------------HSALKEGKKI------------------------- : 113
TrivaTim16 : ---KSIKEHLDRMIKLNDLE--KG---GSPYLNERFIAASHVL----------------------------AK-------------------------------- : 111
NaegrTim16 : ---NLVQEKFDKMFKNNNPK--TG---GSFYIQSKIIRAKQFI---------------EYELIKENKLTEEQRLTFEERTKNIVDQHSSEN--------------- : 150
6 N g y q a

(I)

Tim14

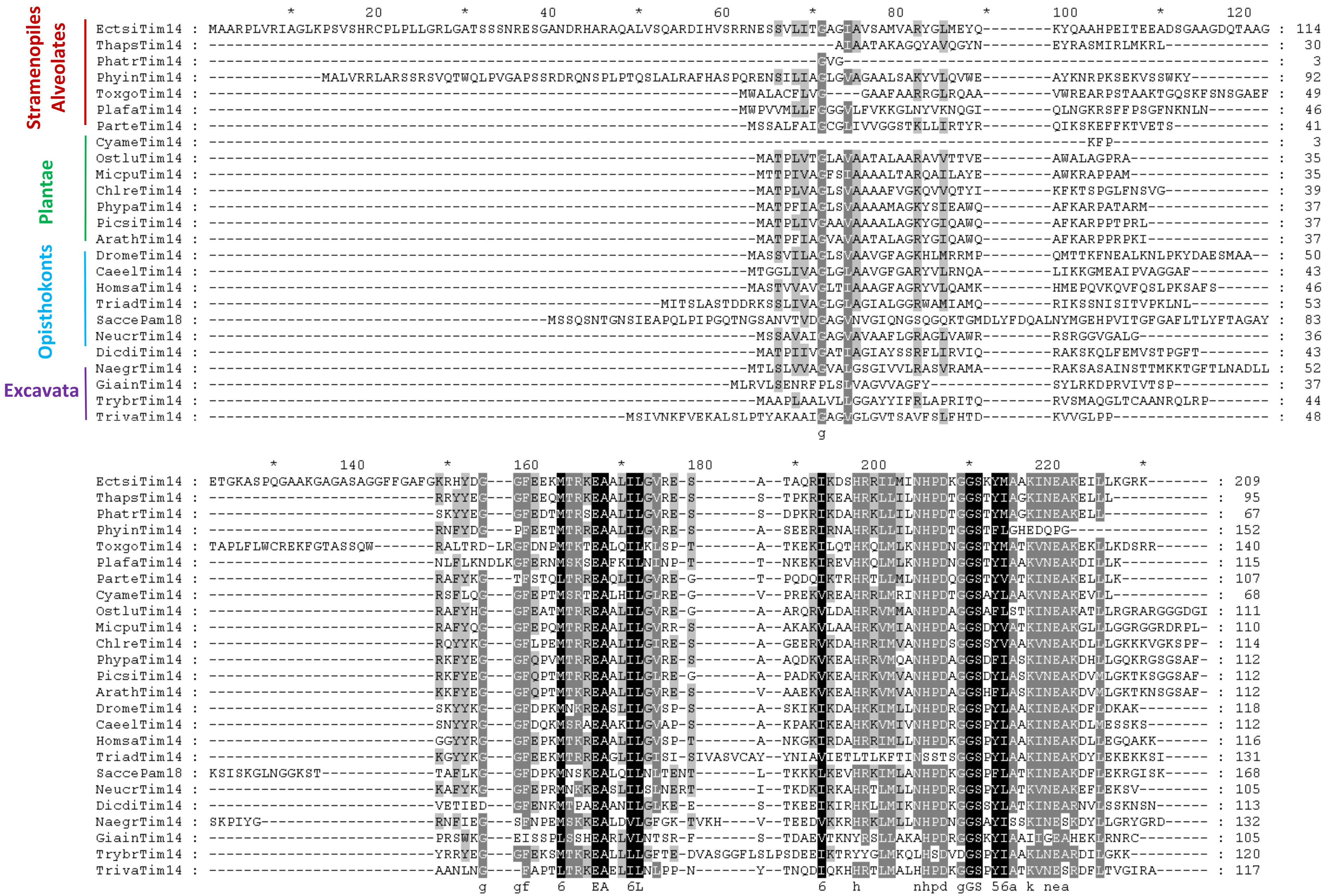
Stramenopiles
Alveolates
Plantae
Opisthokonts
Excavata
EctsiTim14
ThapsTim14
PhatrTim14
PhyinTim14
ToxgoTim14
PlafaTim14
ParteTim14
CyameTim14
OstluTim14
MicpuTim14
ChlreTim14
PhypaTim14
PicsiTim14
ArathTim14
DromeTim14
CaeelTim14
HomsaTim14
TriadTim14
SaccePam18
NeucrTim14
DicdiTim14
NaegrTim14
GiainTim14
TrybrTim14
TrivaTim14

(m)

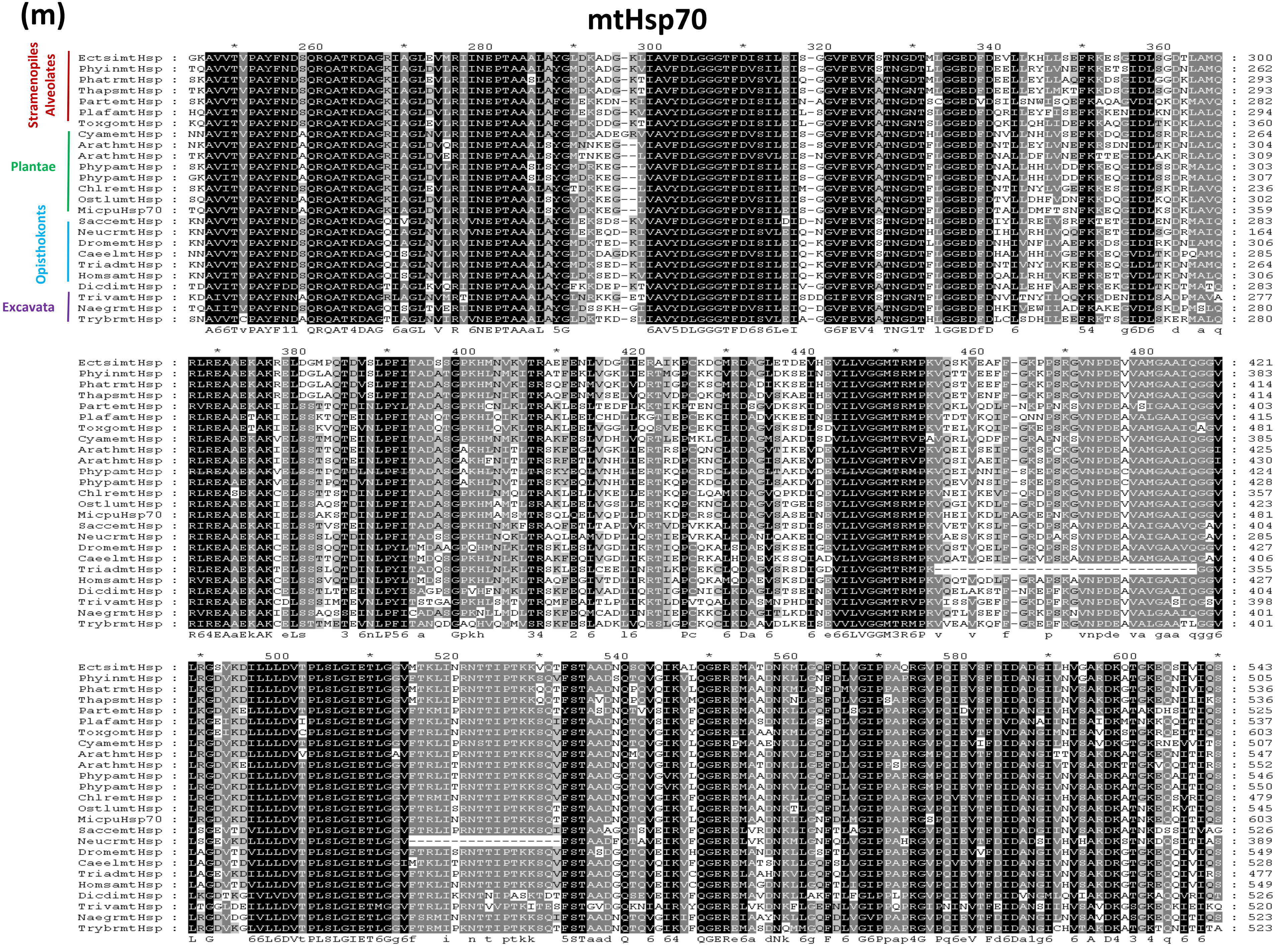
mtHsp70
Stramenopiles
Alveolates
Plantae
Opisthokonts
Excavata
EctsimtHsp
PhyinmtHsp
PhatrmtHsp
ThapsmtHsp
PartemtHsp
PlafamtHsp
ToxgomtHsp
CyamemtHsp
ArathmtHsp
PhypamtHsp
ChlremtHsp
OstlumtHsp
MicpuHsp70
SaccemtHsp
NeucrmtHsp
DromemtHsp
CaeelmtHsp
TriadmtHsp
HomsamtHsp
DicdimtHsp
TrivamtHsp
NaegrmtHsp
TrybrmtHsp

(n)

Mge1

Stramenopiles Alveolates: EctsiMge1_, PhatrMge1_, ThapsMge1_, PhyinMge1_, ParteMge1_, PlafaMge1_, ToxgoMge1_
Plantae: ChlreMge1_, OstluMge1_, MicpuMge1_, ArathMge1-, ArathMge1-, PhypaMge1-, PhypaMge1-, PicsiMge1_
Opisthokonts: SacceMge1_, NeucrMge1_, TriadMge1_, HomsaMge1_, DromeRoe1_, CaeelMge1_
Excavata: NaegrMge1_, TrivaMge1_, TrybrMge1_

```
                   *      140        *      160        *      180        *      200        *      220        *      240
EctsiMge1_ : GEG----------------------------------ED--EDEDQEGLAEKLAKTEERLMDTKEKALYLAAEMENVRSIAKKDAESARLYAVQKFAKQLLDVADNLERAIASAKEAEGEGG : 165
PhatrMge1_ : ----------------------------------------------------EEQLEAQVQQLKDQLLRSLAEQENTRSIAKRDVENGKLYAIKSFAKSLLDVSDNLTRAMEAVPEDARVDQ :  70
ThapsMge1_ : -------------------------------------------------TNKLADLEKQVKDLKDNLLRSLAEQENTRRIAKRDVDQARSFAISSFAKSLLDTSDNLSRALDAVPEELRHDH :  92
PhyinMge1_ : ------------------------------------------------LQKQVEELTTQNKDMNDRLLRALADAENVRRISRVDVNNAREFAISKFAKALLDVSDNLKRAHESIDVATLQPE : 129
ParteMge1_ : FYS-----------------KQIEKLETQIKDHKEKIHD--QIKLINQLEASNKDHNTKIKELRDALKAEIEESELSSKRVLKEKEQLKVFAISNFAKELLDVQDNLERAIASTTDKPEN-- : 192
PlafaMge1_ : SPT-----EGKKKKNSLNESEEANEKKEDINYEDFNKID--LINEIKKTKRDMEEKMVDNKVLKEKYLSVLAENENLRNRYMKEIETSKLYCISNFAKSLLDVADNLSLAIKNINEESLKTN : 219
ToxgoMge1_ : GGASVERMEDEEGSGERRKRSPTESADREQPENEEGEMETALEDKYRQCVEEVESLKKKNRELQDKALRAFADMENARMRHQKEMASLKDYAVSDFAKAMLEVADAMAYATNSLKEAVQTDA : 244
CyameMge1_ : --------------------------------------D--AQERVQRLEGLLQEKEQEIATLRDRAIRLLAEMENVRMIARRDVEAARKYSISSFAKELLDVADNLNRALAAVPRQQLEVS :  82
ChlreMge1_ : GEP--------------------------------SAQE--LMSQLKAKEDHATKLTQQVETLTDSLKRTLAEMENLRARTAREVDVSKKFAIQGFVKSLLDVPDNLERAASVVPSEALKED : 170
OstluMge1_ : GED-----------------------------------------EVSRLRGELEEKDAKVADLTDRILRTMAEMENLRERTRRQAEDAKKFAIQGFCKDLLDVADNLDRAIATVTVDDDEND : 151
MicpuMge1_ : ----------------------------------------------------------QVKDLNDKLLRTLADMENLRERTRRQAENAEKFAIQGFCKDLLDVADNLGRAAATVDADAIAAE :  64
ArathMge1- : TDA-------------------------EIDFDDLSRDD--LVKLVSEKEDLLKVQQKDIMEMKDKFLRTYAEQQNLMDRTNRNAESAKKFAVQNFATSLLDVADNLERASSVVKESFSKID : 199
ArathMge1- : SKDSVTDSAKRKRKGAKGAASSSSESDSESDDDELSADD--LVKLVAEKEELLSEKEEEIKQLKDKVLRTYAEMENVMDRTRRDAENTKKYAVQNFAKSLLDVADNLGRASSVVKESFSKLD : 224
PhypaMge1- : EGT-------------------SESVQHAERSNDIKADD--LARMVAERDALLQEKDKTIKELQDKVLRGYAEVENVMARARREAESTRKFALQGFAKGLLDVADNLGRATGAVPENLRKLD : 208
PhypaMge1- : -------------------------------------------------------------------------MENLIDRTRREAESTRKYSIQDFAQSLLDVADNLGRALETVRKSVSADD :  49
PicsiMge1_ : EIVEPVSQNQEPKKAAQRKESDSESEISSDEEKELSIED--LIRLVQEKELLLKEKHKEIKVMQEKVLRSYAEVENVMDRARREAESTKKFAIQSFAKSLLDVADNLSRASSVVKESFSKID : 218
SacceMge1_ : ---------------------------------------------IKKLESQLSAKTKEASELKDRLLRSVADFRNLQQVTKKDIQKAKDFALQKFAKDLLESVDNFGHALNAFKEEDLQKS : 143
NeucrMge1_ : -------------------------------------------DEVAALKKQLEAKDAEAREWKDKCLRTVADFRNLQERTARDVKQAKDFAIQKFAKDLVESVDNFERALSVVPQDKLKSE : 149
TriadMge1_ : ---------------------------------------------------NIAQIQEELRETRDKYLRTLAEIENMRERTVRQINDAKMYAIQNFSKDIIAVADILEKATESVPQQEIASA : 105
HomsaMge1_ : TEK--------------------------------------------TLLEEKVKLEEQLKETVEKYKRALADTENLRQRSQKLVEEAKLYGIQAFCKDLLEVADVLEKATQCVPKEEIKDD : 133
DromeRoe1_ : --------------------------------------------EVEKLTKELAAAKEQNAELMDKYKRSLADSENMRNRLNKQISDAKIFGIQSFCKDLLEVADTLGHATQAVPKDKLSGN : 131
CaeelMge1_ : G-------------------------------------------AFDVLLKEYDDLQAESLDFKDKYQRSLAETENVRRRGIKQTDDAKVFAIQSFCKDLLEVSDILDIAVKSVKPEDLESG : 152
DicdiMge1_ : -----------------------------------------------SLEETIEKLKEELEETKKQLLYTAADRENVRRFAKEDNEKAKKFGIQSFTKELLEVVDQLEMATNLFPKEKLDEN : 128
NaegrMge1_ : QQT--------------------ENTQKTQQQTESKPAV--DDARVKELEKEIENLKNSNAKYDDQLKRAVAEMANVRRIAKNDVDNAKKFALQSFSKNLLDVVDNLEAGLKHLIEEDVSQI : 180
TrivaMge1_ : -------------------------------------------------KPTIQELEAQIKDIRNRNLFLLAEVENARRRFARLEVEMETYAVSKLAKDLLPVADNMGRIINSGAKQN---- : 111
TrybrMge1_ : AYA--------------------------------------------KLEKELSDAKERIAELKKEVLYRAADAENARRIGSEDVTKAKAYGITSFGKDMLDVVDTLERGLEAITKLPQAEV : 134
                                                                            a   n                 5 6  f k 66 v D    a

                 *      260        *      280        *      300        *      320        *      340        *      360
EctsiMge1_ : GDSSH-------------------DVLLQGVEMTSNELTKVFRSQGLEKYGEVKD-KFDPHLHDAMFEFV--NPAQEP-----------------GTLGQVLKCGYTLHGRVIRAAQVGTVK : 248
PhatrMge1_ : QESNH-----------------VLHNLYEGIAMTERGLLKAFESNGLVKFGQAGE-AFDPNRHEALYEYV--DPDKEP-----------------GTVGQVVKDGFLLNKRVLRPAEVGIVK : 155
ThapsMge1_ : ENHPV------------------LANLYEGISMTDEGLTKAFAKNGLKKFGVPGE-KFDPNKHEALFEYP--DPNGEA-----------------GNIGQVMKVGFMLNDRVVRPAEVGVVK : 176
PhyinMge1_ : KQLDA------------------IEMLHEGVVMTEQQLQKVFREFKINQVGAVGD-KFDPNVHDALFEYE--DATKEA-----------------GSIGQLMKTGYLLNERVIRPAQVGVVK : 213
ParteMge1_ : ------------------------NPLLEGVVMTHSILEKVYKKFGVQKMNVIGQ-KFDPNFHESLFQVE--DPEKEP-----------------GTICYVAQEGYAIGERVLRPAKVGVVK : 270
PlafaMge1_ : EE---------------------INNIYKGIEMTETILHNIFNKYGIDKYNPINE-KFNPQLHEAIFEIN--DSTKEK-----------------GTVATVIQHGYKIKDRILRYF------ : 294
ToxgoMge1_ : LIGPE--ENGDL---DVATLKARLQQIYDGVKLTENLLHKTLDRFGVEQYNPEGE-KFNPALHEALFELE--HPEKAK-----------------GEVAQVIQRGYKIKERVLRAAKVGVSK : 341
CyameMge1_ : SSSNSTGAGGAVSAAESAKLASLLTALYEGVSATERELQRTLQKFGIERYGEIGE-PFNPEVHQAVFEAP--APVADSATSEQSGAAKPSPSLPDGAILHVLKAGYRIHDRVLRPAEVGVVK : 201
ChlreMge1_ : GGVPP------------EKLRNLLAGLLEGVRATESILHKVLKQNGVERYDAAGQ-PFDPNLHNALFDIP--DPTKEN-----------------NTIAVVTKKGYKLNDRVIRPAEVGVVR : 260
OstluMge1_ : VEKVK----------------TKLKSFHEGVVMTEKTLLSAFKKHGVTKFNPEGE-EFDANSHMALFNVP--IPEGSDAKA--------------GTVAAVTKTGYSLHERVIRAAEVGVYQ : 240
MicpuMge1_ : SDGEK--------------LKKMLTSLHEGVLMVEKQLGSTFGKHGVEKYDPTGE-DFDPNAHMALFNVP--DAEKTA-----------------GTIASVTKAGYKLHDRVIRPAEVGV-- : 150
ArathMge1- : TSKDL------------AGATPLLKNLLEGVEMTEKQLAEVFRKAGLVKEDPLNE-PFNPNRHNAVFQVP--DASKPK-----------------GTIAHVLKSGYSLYDRVIRPAEVGVTC : 289
ArathMge1- : TSEDS------------AGAAPLLKTLLEGVEMTEKQLAEVFKKFGMEKYDPINE-PFDPNRHNAVFQVP--DASKPE-----------------GTVAHVLKSGYTLYDRVIRPAEVGVTQ : 314
PhypaMge1- : STLED-----------SSGAAKVLITLLQGVEMTEKQLQQVFRQNGLEKFESEGK-EFDPNYHSAMFELE--DETKTP-----------------GTVAIVTKVGYLLHDRVIRPAEVGVIK : 299
PhypaMge1- : AEINA----------------KLLVSLLEGVEMTDKQLMKVFEKHGLTRFNPEGI-VFDPNEHQAVFEVE--DANKTP-----------------GTVAVVLKTGYKLHDRVIRPAVVGVVK : 135
PicsiMge1_ : PSKDS------------SGAAPLLKTLLEGVAMTEKQLSDVLKKHGVERFDPLNE-QFDPNMHMAVFQVQ--DASKQT-----------------GSVAVVLKPGYTLHDRVIRPAEVGVVE : 308
SacceMge1_ : KE---------------------ISDLYTGVRMTRDVFENTLRKHGIEKLDPLGE-PFDPNKHEATFELP--QPDKEP-----------------GTVFHVQQLGFTLNDRVIRPAKVGIVK : 224
NeucrMge1_ : EQSEH---------------LKDLVNLYEGLKMTESILLSTLKKHGLERIEPEGE-VFNPNEHEATFMAP--MPDKEH-----------------NVVFHVQQKGFKLNGRVLRPAQVGVVK : 236
TriadMge1_ : AANQH------------------FKSLYEGLKLTESQLQKVFSAHGLRKIYPINE-KFDPNFHEALFQVE--NGEKPD-----------------GSIAQVSKAGYLLHGRTLRPAMVGVTK : 189
HomsaMge1_ : NPH--------------------LKNLYEGLVMTEVQIQKVFTKHGLLKLNPVGA-KFDPYEHEALFHTP--VEGKEP-----------------GTVALVSKVGYKLHGRTLRPALVGVVK : 215
DromeRoe1_ : AD---------------------LKNLYEGLTMTRASLLQVFKRHGLEPLDPINQ-KFDPNQHEALFQKE--DKTVEP-----------------NTVVEVTKLGYKLHERCIRPALVGVSK : 212
CaeelMge1_ : GKA--------------------LKDLFEGVSMTRTVMAKTFAKHGLVTVDPTNE-KFDPNLHEAVFQIPSANAKQPV-----------------GHIEVCTKIGYSLKERPIRPAQVGVVS : 236
DicdiMge1_ : KE---------------------LKDLHEGVKMTEQLFLKIMGNQGLQRFNPIGE-KFDFNNHHAIFELN--DPTKEN-----------------NTVGHVVKQGYKLHDRLVRPAMVGVNK : 209
NaegrMge1_ : VKLAQNNPECSE---EMRKKANALFTSIEGVKRTENVLLKVLERNGVTKMEVAEKTPFDPNFHEAMMKVP--PSEKTPH----------------NTVAMVLKSGWILNERVLRPAQVIVAI : 281
TrivaMge1_ : -----------------------VKDVIEAVKLVDAEFHNIFKRFKIEKIVSKGQ-KFDPQYHDAIQMID--TRGSAPS----------------GTIIDCTTEGYKIDKRLLRAAKVIVAK : 191
TrybrMge1_ : EGHKT------------------LSSIHTGIKLSLKLLLNNLAKHGIEKLDVAVGAKFDPNFHDALLKVPP-TAEAPP-----------------GHISTVLKTGYKIQDRVLRASQVGVAS : 220
                                        g6                   g6        F1p   H a                          g 6      G5 6   R 6R a vg
```

(o)

Tim22

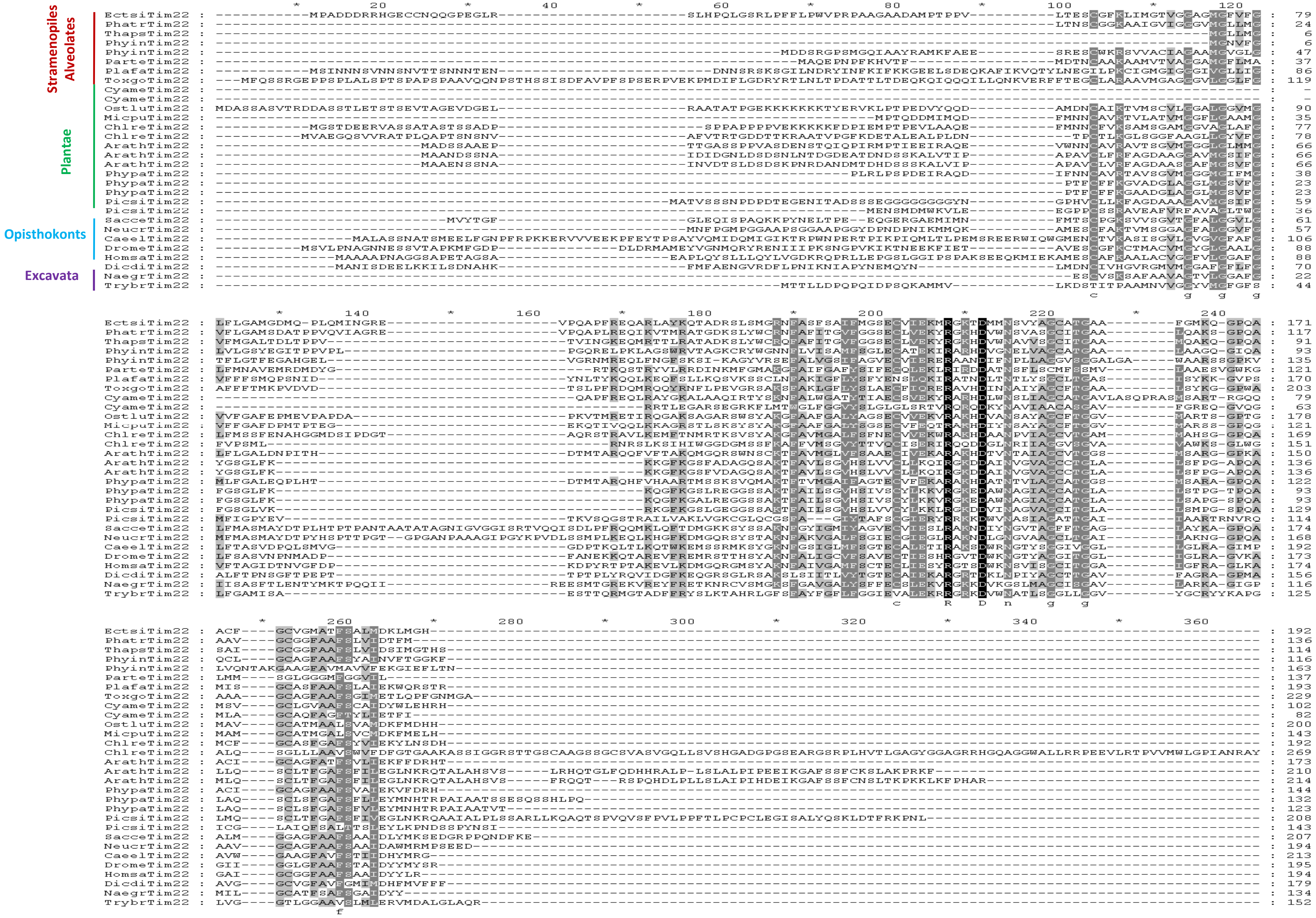

Stramenopiles
Alveolates
Plantae
Opisthokonts
Excavata
