# Supplementary material for: In Silico Survey of the Mitochondrial Protein Uptake and Maturation Systems in the Brown Alga Ectocarpus siliculosus
Source: PLoS One. 2011 May 18;6(5):e19540. doi: 10.1371/journal.pone.0019540 (PMC3097184; doi:10.1371/journal.pone.0019540)
Supplement: Figure S2 — Alignments of complete, predicted Ectocarpus mitochondrial proteins with their eukaryotic homologues. (A) Metaxin, (B) Tim21, (C) Tim50, (D) Sam50, (E) YidC, Oxa1, Cox18/Oxa2, Alb3 proteins. These alignments were used for the phylogenetic analyses. Accession numbers and corresponding names of species are presented in Table S3. Black and grey coloured residues correspond to the amino acid identities (>80% and >60% respectively). (PDF) [file pone.0019540.s002.pdf]

(a) Metaxin amino-acid alignment (p. 1-2)

```
                            *        20         *        40         *        60         *        80         *       100         *       120         *       140
Physcomitr : -----MMDMVSSTSSAWAESSSGSADLVLVTRPP---AFGL----------PTA-------------CPACLPAYLYLRLAGARFDVHVTAVEPDSE------DLPSVEYGENVGFASENGGVVEFLREEKIVDLDAGLNE
Oryza_sati : -----MASAAAAAAAEWEEAERKVLVARKA-------AFGL----------PTA-------------CPTCLPVLLYLRMCNVPFDIHVDSSFPDAD------HIPYVEFGECVAFNNEKGGVIEYLKEEKIVDLNSKHPS
Sorghum_bi : ----MASAAAAAAAAEWEAAARKVLVARKP-------CFGL----------PTA-------------CPTCLPVFLYLRMCNVPFDIHVDTNFPDAD------HIPYVEFGDCVAFNNEKGGVIEYLKDEKIVDLNSNFPS
Populus_tr : -------MQESQERAEYTLVARKP-------------SFGL----------PTG-------------CPICLPVYIHLKFASFPFRLDFNNTFPDSD------QIPYIESGTYVAFNDENGGLIERLREDGIVDLDAAFCS
Arabidopsi : -------MEGDQETNVYTLVARKP-------------SFDL----------PTA-------------CPNCLPAYIYLKLAQLPFELAFNSTFPDSD------ELPYFESDTYVAYNNEDGGVIEKLKKDGIVNLDSQLQS
Ectocarpus : -MASEEHGDAVPVRTSWNPLRALR-------------SWGRIEEQPDLEQPGPVITHGSALDVMVVTQFRPAWEIQAHLRFVRLPYRVE-NSSYMGSAATG---LYPALTDGQF-VL--RSEDAAGHIASR-RSDVDVGLTE
Phytophtho : MFPEDRFHPLPGPADRLHDDLIEATELESTPKTSR--ELGS----------ALVLHQ-----------FLPAWDVQAYLRVAGIQLHVH-NSKYPTYEATG---ELPQLSDGNF-LL-PK-EEIIMHLQTF-HKDIDEFLTD
Albugo_lai : MFPTNRFTSLLPSAPSLKQILRKENDEDLEFVPKTSKDYGS----------RIVVHQ-----------FWPAYNVIAYMRMANLNFHVH-NSRYPRYEVSG---ELPQLHDGNY-LL-SK-SDINLHLQTF-HTDIDVHLTA
Canis_fami : --------------MMWSGNRRVRVQKAAE-------PWPE----------NATLYQQLKGEQILLSDNAASLAVQAFLQMCNLPIKVVCRANAEYMSPSG---KVPFIHVGNQ-VV-SELGPIVQFVKAK-GHSLSDGLDE
Ictalurus_ : -------MSLAAEAFVSQIAAAE--------------PWPE----------NATLYQPLREDQILLSDCASSLAVQAYLRMLGLPVRVRCRANAEYMSPSG---KVPFIHVGNQ-VV-SELGPIMQFTRAK-GHSLSDSLDD
Takifugu_r : -------MSLAAEAFVSQIAAAE--------------PWPE----------SATLYQPLKEDQVLLSDCASSLAVQTYLRMCGLPVEVMYRANAEYMSPSG---KIPFIHVGNQ-VV-SELGPIVQFTKAK-GHSLSDGLDD
Nasonia_vi : -------MQNSTLLDPWNVELGASILLVAAQE-----PWPQ----------PIHLYQPYEVEQILLPDNANCLAVQAYLKMCNLDFTIEPRWNAEYMSPSA---KVPFIKCGAF-VI-PDYDNIVSFISSK-GAS LSEGLSE
Solenopsis : -------MPHVLLGDSIAVELEAQE------------PWPQ----------PITLYQPYEVEQILLPDNANCLAVQAFLKMCQLDFQIEPRKNAEFMSPSG---RVPFIKCGTK-LI-SEFDGIVAHIGSK-GISLSDHLDP
Brugia_mal : -------MTGSYFMTDLVADSLSSEIDV---------DWS-----------GAVLYTPYYT-QALLYEYADCVAVQTLLKMANLPVRLEERPNAEFMSPTG---KVPFLKLQSF-LV-SEFLPVVDFLAKR-NVKLSAGLTD
Drosophila : -------MKSQYLNQLHTAEKLVAE------------PWPE----------DATLYQPYEAEQILLPENASCLAVKAYLKMCNLPFGIRSCANAEHMSPGGRMTKLPFIRAGAF-IF-AEFEPIVNFVEQK-HLAIGNWQDE
Aedes_aegy : -------MSAITSEYLKNETIASQ-------------PWPQ----------QAHLYQPYEEEQILLAEHASCLAVRTYLTMLNLPFVVDQRANAEFMSPGGKRTKLPVLRVENY-TY-AEFEHILSFVELK-GLSLTKQLTP
Anopheles_ : -------MASTIDNFYQKTATAAMK------------DWPR----------DAVLYQPYEEEQILLAENASCLAVRTYLKMLNLPVALEQRANAEFMSPGGKRTKLPVLRVENF-IY-AEFDHIVTFLEKNFNKSLSAPLTP
Ciona_inte : MSSPKKSTDGKMEVKCWGE------------------GWGL----------PSL-------------DPDCLSVIAYANLAKAPIEV--VPALPKDTITG---SLPELQIDEF-IY-PRAFTIIGIFRRE-GYNADYSLSQ
Acyrthosip : -----MFKPHQLEVESWKG------------------DWGL----------PSI-------------DYKCLEVLAFARFTNAPINIY-HSNNPYRSVTG---HLPVVSYNKR-LL-MDRDSLIDCLEKQ-NLTPDFGLTS
Pediculus_ : ----------MELEVWGG-------------------DWGL----------PSI-------------DLDCLQILVYCKLSGAPVQLK-VKNNPLTT------SLPTFRHGQV-VL-YKLQDVVSYLQSK-NFYADHILSS
Camponotus : -----MDNAEILQLDIWKG------------------DWGL----------PSV-------------DIDCLQVLAYAKFSGIPLKVN-LTSNPFKTPNG---RLPLLRAGLN-TL-DTVKDILPFFRAK-HYNSDYTLTD
Apis_melli : --------MDMFELSIWKG------------------DWGL----------PSV-------------DTECLQVLVYAKFNGIPLKIN-SAGNPFTTPNG---SLPVLKTRNG-TF-NAVKDIIEFFRKE-RYNTEYELSR
Strongyloc : -------MAAMMEMDCWKG------------------DWGL----------PSV-------------DRDCLKVMAYAQFAGAPIKVH-RDRRLWRNISA---HYPVFYSDGR-TL-KSADSIIEHLKQN-QFDADSELND
Ixodes_sca : -----------MELNIWKG------------------DWGL----------PSI-------------DTACLEALAYAKFSGAPLIIN-EVRRPWFA------TLPIMRHGPSTRI-TKFEDMVAHFRKQ-NYSADIQLTA
Nematostel : -------MADVLELFSWPG------------------DFGL----------PSV-------------DIPCLAVLSYVKFAGCPLKVN-RSNKFWKSPTW---EFPVMKSGEE-VL-TSPYKIMDHLRQK-NFNADYQLTA
Mus_muscul : -------MAAPMELSCWGG------------------GWGL----------PSV-------------HSESLVVLAYAKFSGAPLKIN-IIDNTWRGSRG---DVPILTTEDS-IV-SKPAKILNFLRKQ-KYNADCELSA
Salmo_sala : --------MATSELYCWEG------------------DWGL----------PSV-------------DTECLIILAYAKFAGAPLKLH-KIGNPWRSPTG---SLPALKTSENGSL-SRPSDIIIHLRKQ-KYNADFDLSA
Monodelphi : -------MAAPMELYCWAG------------------GWGL----------PSV-------------DLESLIVLTYARFTGAPLKVH-KITNPWKSPSG---TLPALRTPDASVI-SQPHKIITHLRKQ-KYNADYDLSA

                          *       160         *       180         *       200         *       220         *       240         *       260         *       280
Physcomitr : RERAELETCKAMMQSWVADASAYEVWM---RDNSRQCKTVYFSELPWGLVQALDWKQRLAVMQRLEITPEN-----TMTRTEELYKKASNAYSALSILLSDQ--K---YFFNDRPTSLDALVLGHLLFHLRVP-LEVS-TLK
Oryza_sati : VSYSDVLSTKAMVMTWLSDALQYELWL---ASDGSIPHDIYFSDLSWPIGKILYWKKTREVKQQLGITKLN-----AAEKEEEIYRKASAAYDALSTRLGDQ--I---FLFDNSPTDVDALFLGHALFVLNV--LPDTSVLR
Sorghum_bi : VSSTTVQETKAMVSTWLADALLYELWV---ASDGSIANDIYFSDLAWPIGKILHWKKTRHVKQQLGITKLN-----AAEKEEEIYRKASAAYDSLSIRLGDE--V---FLFDNSPTDVDALFLGHALFVLSV--LPDTSVLR
Populus_tr : L--PEWISMKAMVCTWLAEAVMYELWV---GSDGTSARAIYYSDLPWLIGKALFMKQVYVVKQRFGITKEN-----AERKEAEIYKRAKIAYGALSTTLGDQ--T---FLF-ERPSSLDAYLLGHVLFTLQA--LPESSVLR
Arabidopsi : L--SDYLSLKALIVSWLEEALTYEIWV---GTEGISTSKIYYSDLPWVISKVLFYKQTYLAKNRLGITKEN-----AEQREKQIYKRASEAYEALSTRLGEQ--K---FLFEDRPSSLDAFLLSHILFIIQA--LPVTSVLR
Ectocarpus : AEKVEAQALAMLVREGLQPLLRVMRYMGDEGEVRQTVHPPMKKALSWPLSWWSPAAEGRRSKRESAVR-GL-----DRLSKAELIGRAKEMYAALDLRLGNSKEA---FFFGSRPTSVDAVVFGHLAEAWTIA------VLL
Phytophtho : QQRSESYAYRSMLSEKLQRVMLYCRWV-DSATYREVTRPHMKRHIPFPLSLFLPKKMHLDTMEQLRLY--------GISTKEQAYVIARDCYTALNAKLESAGTP---FFFGDQPSALDVAVFGHIVD----A-MGNT-QLV
Albugo_lai : EEKADLFAFRSIVTEKLAYVLLYCRWV-DDVEYTEVTLPQLRNVIPFPLNRILPKMMRNAALREAQAH--------GICSREKAYMLARDCYASLNAKVSENSSR---YSFGYHPTSLDAEIVGHVID----G-LANT-QLR
Canis_fami : VQKAEMKAYMELVNNMLLTAELYLQWC-DEATVGEITHARYGSPYPWPLNHILAYQKQWEVKRKMKAI-GW-----GNKTLDQVLEDVDQCCQALSQRLGTQ--P---YFFNKQPTELDALVFGHLYTILTTQ-LTND-ELS
Ictalurus_ : VQRAEMKAYMELVNNMLLTAELYIQWC-YQNTAAEITRPRYSSPYSWPLNHILAYQKQWEVRRKMNAI-GW-----AGKSLEQVYEDVSQCCQALSQRLGTQ--P---FFFNKQPTELDALVFGHLFTILTTR-LTTD-ELS
Takifugu_r : VQRAEMKAYMELVNNMLLTAELYIQWC-DDATATGITRPRYSSPYSWPLSSFLAYQKQWEVRRKMNAI-GW-----GGKTLEQVYEDVNQCCQALSQRLGTQ--P---FFFNKQPTELDALVFGHLFTILTTR-LTST-ELA
Nasonia_vi : EDKVNLRAYQSLVNNVLHNAELYVCWC-HEDTYNSITKNRHGSVYPWPLNHLLNWQKRNQITKRLNVL-GY-----ATKTLKEIFDDVEKCCEALSERLEDK--M---YFFGDRPNELDALVFGHIFSIITTP-LTKDNTLA
Solenopsis : AGKVDMRAYQSLVNNVFVNAELYICWV-DPAILDGVTKQRHGSVYPWPLNYYLNWQKRREVIKKLSVL-GW-----YNKSLDEVFDDVKKCCIALSERLADE--E---FFFGKDPTEVDALMYGHVHTLIAFP-FSSNRDVA
Brugia_mal : LERGDMHAHMALFDDILKNVEMYVMWM-DKRNYSQVTKCRYGSVYLFPLNLILPRVKWREVNNYLTAV-DW-----KSKSQECVMDLADRCFKSMSSKLDHN--E---YFFGDSPTELDALAFGHFYTILTTE-LPNM-ELK
Drosophila : DEKADMRTYVSLVENIFTMAELYISFK-NERVYKEVTAPRNGVVFPWPLNHMQNYGKRRNALRLLKVY-QW-----DDLDIDSVIDNVAKCCETLEYKLKES--PETPFFYGDQPCELDALAFGHLFSILTTN-LPNM-ALA
Aedes_aegy : DQKDDMRAHLCLVEQIFTNAEQYISWV-DPEVLHKVTRQRNGCVYPFPLNHIQNWRKQMAVRRQLGVA-DF-----LNITLDEVVEKVEKLCTSLSMQLGDK--K---YFYGDEPTELDALVFGHLFSIFTMT-LPNN-VLA
Anopheles_ : EEKDQMRSTNCLVEHLFTTAEQYVSWI-DPEVRNTVTKKRNGCVFPFPLNHVQNWRKESAVRRQLRMA-DY-----LHEGIDTIMGEVDHLCQDLSSRLGDK--R---YFFGDSPTELDALVFGHLYSIFTMK-LPNN-VLA
Ciona_inte : SENADTLAFLSYIEQKLKPAILYSLWI-DVRNFNKVTRPAYGKACGFPWSLWYPSRLVKSYTNQLWLSKGGESFTCIKEVEKVIYKDAHDCLNVLESRMSST--D---YFFGDFPTTIDAVLYGHLAVLLHAP-LVST-ELQ
Acyrthosip : LQLAEEHAAISMIDAQLEPALLFAWWM-DESNCLNFTKPWYRNALKFPFNWYYPNVYEREAKHKIYTLYNHLDS--DNDIMMEIYAEAIKCMNVLESRLGNN--F---YFFGSHPTLLDAVAYSYLGPLLKAP-LTGN-RLQ
Pediculus_ : RNISEIQAYIELLKEKLYPGLQFVWWV-DNKNCVNLTKSWFAKKLMFPLNFYYPGHYEAQAKNLITALYGSLEEN-LAAIETQVYSDAEKCLTLLSNRLGES--K---YFFGNQATSLDAIIYSYLAPLLRAP-FPNP-TLQ
Camponotus : KQCADVLAYDALLKEKLYPALQFIWWI-DKKNLDELIRPWYCKALPFPFKFYYPGKFERQAQALMQSLYSMEDN--IDVIENEVYSEAQKCLTLLSMRLGDG--D---FFFGQQPSTIDAIVYSYLALLLKAP-LPNP-VLQ
Apis_melli : KQCAKVMAYGTMLKEKLFPALQFIWWI-DEKNVNELIRPWYCKALPFPFNFYYPGKFERQAHTMFVTLYPLEDN--ITAIENKVYSEAQKCLTLLSTSLGDS--Q---YFLGQKPTILDAIVYSYLAPLLKAP-LPNP-ALQ
Strongyloc : QQRADILAYSSLLEEKLLPALQYTWWV-DAKNYTEFSRPWFAKTMHFPFNYFIPGQLQRTAESSLEVRRGGLHLL-DGELSQNVMKDARYCLNMLSERLGEK--E---FFFGESPTSLDALVFSYLAPLVRVP-FPSN-TLQ
Ixodes_sca : QQSSDVRAYTAMLRQKLKPALLYLWWV-DAKNYVELTRPWYARALAFPLNYVLPGQMQRDAMAALQCKLELLDLE-GKQAETALLKEAQECLTTLSQRLGKD--T---FFFGKRPTSLDAVVFAHLAPLLKAP-FPNA-ALQ
Nematostel : KQGADTLAFIALIEDKLLPAMHYTLWV-DSKNYVEFTRPMYARKLPLPLNFFVPGRIANQKKLRIGHKLDPEEEEANGELENMLFKEAIECLTHLSVLLGDK--D---FFFGESPTTLDAVAFAHLALIWRAPSLPNN-KLA
Mus_muscul : KQGADTLAYIALLEEKLLPAVLHTFWV-ENENYFTVTKPWFASRIPFPLSLILPGRMSRGALNRILLTRGEPPLYHVQEVEAQIYRDARECLNLLSNRLGTS--Q---FFFGDTPSTLDAYVFGFLAPLYKVS-FPKV-HLQ
Salmo_sala : KEGADTLAFISLLEEKLMPALVYTRWI-DPKNYVEVTRRWHAEHAPFPLNFFLPGSIQRQQLQRLRLVRGDEELTAGEEVEKELYRDAMECMNLISQRLGSN--K---FFFGDSPSSLDAFVFGYLVPILKMK-LPNG-RLQ
Monodelphi : RQGADTLAFMSLLEEKLLPVLIHTFWV-DAKNYVELTRKWYAEAMPFPLNLFLPGRMQRKHLERLQLLCGERRPEEEEEVEKELYREARECLTLLSQRLGSQ--K---FFFGDSPASLDAFVFSYVALLLQAK-LPNG-KLQ
```

```
                 *        300        *        320        *        340        *        360        *        380        *        400        *        420
Physcomitr : EAILKY-QNLVDYAEHWSKYLLG-----EEGNSIDSSFRPKTPHSSHASPPRQGPKERDNP-------------EKERREKDIFFKKRAKYF---------------------VIAQVTAVLMYVVFAGYGVDDDGE-----
Oryza_sati : SCLQKY-DNLVNFTKHLKVQLLE-----------------ADSDSSATGLGSTDPSSSSTP-------------RKRASSGRMQ-----------------------------PVRGACSFAQHADGLGPQIFSASD-----
Sorghum_bi : GTLQKH-DNLVNFAEHHKVQLLE--TSSSSSSGLGSSPSPSSSTPRRRPSADQGYKPKPRA-------------KKERTEEEKKFRRRAKYF---------------------LATQLVAVLVFLSLMGGVDSSELDD----
Populus_tr : LALSEH-GNLIRYAEKLKSEYLE----------GGSSSSVPQFHSEASSTSTRRSKTKKQP-------------KREKTEEEKTFRRRAKYF---------------------LVTQLVAVLVFLSVMGGYDFSEVEVDD--
Arabidopsi : CKLLEH-SNLVRYAEKLKSEFLE----------ASSSSPSPPLHSFPSSFPRKSSKPKSKP-------------KVEKTEEEKKFKKRARFF---------------------LAAQFLAVVIYVSVMGGGSSD--------
Ectocarpus : DLLPAF-DNLSRLFRHVCDNYFR--------------PGSFPPPSSGGEGVAESKKSGSED-------------RLRDAMLRADYVNSHNAFNQLAGCALCSEVPYIEDPYPPRPIANAGIPPPVLAGDLPGVEGAQGTAAA
Phytophtho : STVHQHAPLLITLAERIRDAYFGAPGEQPSSLSEAAMYSENQPNYFTTSLDSAFMKKVSPPLQVAFLKPYRSLDWSRRELASEVAQKKRAKEAEERTQDEEHEEVFQKGSRNVVIGAIAALVLYGVATLPITVVDGDESDED
Albugo_lai : DVLFEFAPSLIDVAKTVRAQYFS--RDPEDYVGCLKAYTENEDNYFVKQTPRHFLDPVSFPVDKALLTPYQSIDWRKREMIEEIVEEKIKSSSDRLKDPSQDSVGIEGKRNL-VIGAVVTFALYAISCLPIEFASNDSPE--
Canis_fami : EKVKNY-SNLLAFCRRIEQHYFE--------------------------DRGKGSSSIRSS---------------------------------------------------------------------------------
Ictalurus_ : EKIKSF-SNLLSFCRRIEQAYFE--------------------------SQDRDSQSGALD-------------YSKGPSLM------------------------------------------------------------
Takifugu_r : ERIKSH-SNLLSFCRRIEQTYFE-----------------------------------------------------DKSS--------------------------------------------------------------
Nasonia_vi : NIVRNH-PTLIKHCRNIEDRYFR------------------------------------------------------RDEE-------------------------------------------------------------
Solenopsis : AIIRQF-PKLIEHMFRVKLYYIS----------------SGTRNEIAEEFEIIESSSKATL-------------WDDFRS---------------------------------HPESLPPLSEHSFTLSIN-----------
Brugia_mal : NCLRRY-SNLTEFCQRIDKKYFV-------------------------------------P-------------GTEAKK--------------------------------------------------------------
Drosophila : QTVQKY-KHLVEFCRFVDEKYFQ---------------------------------TRCLP-------------N-------------------------------------------------------------------
Aedes_aegy : VTINQF-KNLTKFCKNIEEKYFK---------------------------------------------------KVAGGR--------------------------------------------------------------
Anopheles_ : LTIHKY-SNLNQFCKNIDETYFS-----------------------------------------------------VKGK--------------------------------------------------------------
Ciona_inte : NHLNSC-DKLRAFCARMS--LFC---------------------PSSSSLPTQVKSNSEDQ-------------IINRDKIIS------------------------------IAVAVTAMLVYALTSGVLTARRSKLATK-
Acyrthosip : SHLKTC-KNLCIWIDRITREYFK-------------IDWQAHKCNEKKKLEETKQQMNVTP-------------WYKRDETKNVLI---------------------------GIVTVITMVVYSFKIGLISVS--------
Pediculus_ : NHLKAC-NNLVSFVIRISQKYFP--------------AISEEFEKTQKENIQKQQTNEEFP-------------NKKRNQFIA------------------------------GVIAVLAMATYALSLGIVQTSKYVDTIE-
Camponotus : NHLRNC-TNLVKYVSRISQRYFE------------YEYQTYEKDKAEKNAEKLGEDECEFP-------------NKRRNQIFA------------------------------AFVAAMAMAGYALSTGIVEVSAKDDETSD
Apis_melli : NHLKAC-TNLVTFISRISEKYFA--------------NECCEYKAKENAQNIRKYSQNEFP-------------NKRRNQLLA------------------------------GLFTTLIMATYIFSTGILEM---------
Strongyloc : VHCKAC-ENLVMFCSRILQRYFP--------------QDPQEPQTAPKESQATSEEVFDDP-------------HKTRNQILS------------------------------VLFAATAMVGYALFSGMVQFEIVDDDEMD
Ixodes_sca : NHLKAC-ENLAAFVGRILQQYFP-----------PASADSEKGDRPGGGSPGSSDTDFNFS-------------WLNTTLS--------------------------------VAFAAGAMLIYAVTSGLIQIEFQEA----
Nematostel : NYLKGY-DNLYNFCGRILQRYFP----------------PDPEDPQQYKPSPTHPSLDEDP-------------YQKRKMWLS------------------------------VGIAVVAMVTYAVLSGLVQIEYKKDEKQE
Mus_muscul : KHLKQL-CNLCRFCDDILDSYFR--------------PGPGGVSPAGQEMVDANLQKLTQL-------------VNKESNLIEKMDGNLR-----------------------QSPQLLPRKLPTLKLTPAEEESNSLQLLS
Salmo_sala : QHLKSL-DNLSHFCSNILALYFP--------------SEGREGISRKVSSQPEGGDADNEP-------------GKRRKQLLS------------------------------VLVALGAMLSYAFLTGIVAIQHVQQEALD
Monodelphi : AHVRGL-GNLCAYCTHILSLYFP----------------WDGAEAPPPRPALSASETDEEP-------------YRRRGQILS------------------------------VLAGLAAMVGYALLSGIVSIQRAAPARPS

               *        440        *        460        *
Physcomitr : ----------------DDGGDDDD----------------------
Oryza_sati : ----------------------------------------------
Sorghum_bi : ----------------DDGLDYED----------------------
Populus_tr : ----------------DEGFSYD-----------------------
Arabidopsi : ----------------ELEYEDEDD---------------------
Ectocarpus : GSTTAAAAAAEAAAGKATAANESLPVKYTVISIAAFMVLSNILSRG
Phytophtho : ----------------EDEYDEDDE---------------------
Albugo_lai : ----------------DPSLSRSAD---------------------
Canis_fami : ----------------------------------------------
Ictalurus_ : ----------------------------------------------
Takifugu_r : ----------------------------------------------
Nasonia_vi : ----------------------------------------------
Solenopsis : ----------------------------------------------
Brugia_mal : ----------------------------------------------
Drosophila : ----------------------------------------------
Aedes_aegy : ----------------------------------------------
Anopheles_ : ----------------------------------------------
Ciona_inte : ----------------LKQLNEFDEFEQMD----------------
Acyrthosip : ----------------DTSDEEDED---------------------
Pediculus_ : ----------------DEGIDDDDD---------------------
Camponotus : MST-------------EYILDDEDAE--------------------
Apis_melli : ----------------------------------------------
Strongyloc : ----------------DHGMMDDEGDDYQAQE--------------
Ixodes_sca : ----------------EKEAQDED----------------------
Nematostel : TGDDELLPDVTSFQYQDERDDE------------------------
Mus_muscul : P---------------------------------------------
Salmo_sala : HRSPGLRSL-------SSHGEEEDGEEEG-----------------
Monodelphi : GNR-------------ALGIAEEEEEE-------------------
```

(b) Tim21 amino-acid alignment (p. 3-5)

```
                       *        20         *        40         *        60         *        80         *       100         *       120         *       140
Phytophtho : ---------------------------------------------------------------------------MLRFLSAPAIR--------------------------------------------------------
Esil_TIM21 : ----------------------------------------------------------------------------------------------------------------------------------------------
Thalassios : ------------------------------------------------------------------------------MSIRSAFI-----------------------RPVVSFTKKGASNASSSRGGGIPLLAMMSTYSK
Phaeodacty : ----------------------------------------------------------------------MIRCAANRLLLHQGRL-----------------------GLLQHS---------------------------
Physcomitr : -------------------------------------------MEERSSGSVPCYRAVAGAALVLPPSRQENREAMAGVLVRKARMRFASGAARTWDAALRQQKPRLLADQADLEVTGYCYFSSSGVSSSQGFLKRIMRPRS
Zea_mays_g : -----------------------------------------------------------------------MASRVTRLLLHRRLT-----------------------TAAEASARRAP----------------QAPCAR
Ricinus_co : ---------------------------------------------------MEHFRRSAMSLRSRCSDLFECQNGLSSVLERKLIK-----------------------RVTDVGLATFSSSATSERAAVNNSTKKLPRDFK
Ostreococc : ----------------------------------------------------------------------------------------------------------------------------------------------
Micromonas : ----------------------------------------------------------------------------------------------------------------------------------------------
Schistosom : -------------------------------------------------------------------------MAILSAKLNVVKI--------------------------------------------------------
Strongyloc : MVDTGMSAQRKRRAQRMANRCQRLALGPVSDHGNEILQAIEIVKAILVKAKQEAQAYPGGDFPEESKDQQIDSSAISTFIPKQSYTLEPEKSAHQNKSVDEEVIQMLQ-PKVSCQTLKLDDLLTFDMGWTVKSINTLQEIGR
Macaca_mul : --------------------------------------------------------------------------MICTFLRAIQYT-----------------------EKLHRSSAKRLL---------------LPHIVL
Pan_troglo : --------------------------------------------------------------------------MICTFLRAVQYT-----------------------EKLHRSSAKRLL---------------LPYIVL
Xenopus_Si : --------------------------------------------------------------------------MMLPRCLRRAVL-----------------------CSSRALGSPRSL---------------TVPCYR
Danio_reri : ---------------------------------------------------------------------------MSSLVLRIGRT-----------------------TNCVQHILKRVHVR-------------LQQPRS
Anoplopoma : --------------------------------------------------------------------------MAYTHILRALLH-----------------------RNLQQTCKLTQVS--------------LLIHTH
Osmerus_mo : --------------------------------------------------------------------------MIYSFVLKSMHR------------------------NLQQAVWIYSP---------------LQICKR
Vanderwalt : --------------------------------------------------------------------------MINLVLKRSPKF--------------------------------------------------------
Kluyveromy : --------------------------------------------------------------------------MFCQFISRPAQS--------------------------------------------------------
Saccharomy : -----------------------------------------------------------------------MSSSLPRSLLRLGHR--------------------------------------------------------
Candida_gl : ----------------------------------------------------------------------------------------------------------------------------------------------
Podospora  : ------------------------------------------------------------------------------MMIKSHLT-----------------------PVLRLR---------------------TLPSTS
Magnaporth : -------------------------------------------------------------------------------------------------------------MKLQTAAAANII---------------IPRALA
Aspergillu : --------------------------------------------------------------------------MSAYLLPRPSLH-------------------------------------------------------R
Microsporu : -----------------------------------------------------------------------MNSSIQLHSLRSGPL--------------------------------------------------FVRLVR

                     *       160         *       180         *       200         *       220         *       240         *       260         *       280
Phytophtho : ---------------------------------AAQKSTLQQRSRAVQV---------------VARR--------------------------------------------------------------------------
Esil_TIM21 : ----------------------------------------------------------------MMSK--------------------------------------------------------------------------
Thalassios : QSTDVAYRLAT----------------------PLKARSLAAQLFIRGGTSAMRSPINHAHRSNTLRA--------------------------------------------------------------------------
Phaeodacty : ---------------------------------KASTSLLRWNQLRWQP---------------------------------------------------------------------------------------------
Physcomitr : QIVPLNYRTGGVDSIQQVDVNFIGYRNVLLNKMSRFSTCTTSQMLHQLPKKEHVASLLPYSTRGIASQ--------------------------------------------------------------------------
Zea_mays_g : AAVSKDVVKAE----------------------ASSLKNSRWYITRSNTSGPLTTRYECRKVFPCSVR--------------------------------------------------------------------------
Ricinus_co : LGYVRRPVGEANHGPKDI---------------LCANGNYDKQCIAGLGSQFQLVKLLGGSTTNSWFA--------------------------------------------------------------------------
Ostreococc : --------------------------------------MSRDRAYATRR---------------GTKS--------------------------------------------------------------------------
Micromonas : ------------------------------------------RASARAA---------------------------------------------------------------------------------------------
Schistosom : ---------------------------------PISAGILRLQFYPSIK----------SSVSSISQQ--------------------------------------------------------------------------
Strongyloc : GSYGRVVIARHRSTGVPLAIKEPSWSDDFASPGEMVEELKATRTRATKEAMIQQLLSGSPYFPKFWGTLDLGNELCQAVEFVGCKKTGTGYPLHEPPSLSMCNVVKIAEDIVKGMMEFHDHGLLHNDLKNDNVLLEKRGKRY
Macaca_mul : NKACLKTEPSLRWGL------------------QYQKKTVRPRCILGVT------------QKTIWTQ--------------------------------------------------------------------------
Pan_troglo : NKACLKTEPSLRCGL------------------QYQKKTVRPRCILGVT------------QKTIWTQ--------------------------------------------------------------------------
Xenopus_Si : NLSCL--------------------------------LLSQQRALAVPT-------------SRFCAE--------------------------------------------------------------------------
Danio_reri : SLLTLDPVFQ-----------------------TNHCPCSKWAFYHTRS--------------LYTGV--------------------------------------------------------------------------
Anoplopoma : SVSTFLRRVE-----------------------APSWGLPVLSCFIQAQ-----------SQRGFSLD--------------------------------------------------------------------------
Osmerus_mo : TQSNFILQRR-----------------------GNFASTSTPFFHGIEGHPSILGSVLCTQKRNIYFH--------------------------------------------------------------------------
Vanderwalt : ---------------------------------GTSLKVIAKPIYSTALQRSLI----------------------------------------------------------------------------------------
Kluyveromy : ---------------------------------LSGKFSSKTLCLPLIA---------------------------------------------------------------------------------------------
Saccharomy : -----------------------------------KPLFPRYNTFVNSS---------------VITH--------------------------------------------------------------------------
Candida_gl : -------------------------------------MVLRSRPLASI----------------------------------------------------------------------------------------------
Podospora  : PVVNL--------------------------------RALQPRHYATHH------------------Q--------------------------------------------------------------------------
Magnaporth : SQ-------------------------------QIRPLIASRRCYATN----------------------------------------------------------------------------------------------
Aspergillu : ASSALLLRP------------------------GFPRTVELTRCYATHSDL-------------------------------------------------------------------------------------------
Microsporu : NSGR-----------------------------PVSLSLTRYNHYATQS---------------------------------------------------------------------------------------------
```

```
                         *       300         *       320         *       340         *       360         *       380         *       400         *       420
Phytophtho : ------------------------------------------------------------------------------------PAASTRSFTSTRSRLNADKAT-----PEGQKAPDALV---------------------
Esil_TIM21 : ------------------------------------------------------------------------------------ASRKGDDSKGEQQQQQKQQGEE----QQYEDADYEPE---------------------
Thalassios : ------------------------------------------------------------------------------------TSSSTNSSGGQKDKASSEKTTGT---EGEEPMSQEIV---------------------
Phaeodacty : ------------------------------------------------------------------------------------SMLRTLSSDASQKGGNEKKK------SEGEDTTSEIV---------------------
Physcomitr : ------------------------------------------------------------------------------------SSPLLRQAQNESKGLSKQPEE-----DPFDSITDKIP---------------------
Zea_mays_g : ------------------------------------------------------------------------------------PSASYSTQASDQKGKQEGKDLSNVE-HPFDDITYNIP---------------------
Ricinus_co : ------------------------------------------------------------------------------------RSFASKASKKTGEATSETRKEVSTVEDPFDAPTYNIP---------------------
Ostreococc : ------------------------------------------------------------------------------------SSSADEAASGTRGDLASTPESG----RYDEITDKWIP---------------------
Micromonas : ------------------------------------------------------------------------------------AAASSESAKSARADVATTRGG-----TYDEITDKWIP---------------------
Schistosom : ------------------------------------------------------------------------------------WPVCSRRSFSSSSNSSVTPSE-----GRGNLPSTSVT---------------------
Strongyloc : NAVIIDYGMASSITAPPKMVGVTKEVKMAYVHGGEADYITPEVVLDEQPTSIASDVFSVGRIFSDMGEIFGGHRFPVVRELSKLGAQCMADNPDHRPSLDTIKSQITRLQRPSRWRRVKRWVAKKLHLRRKLSVIEGYLDSK
Macaca_mul : ------------------------------------------------------------------------------------GPSPRKAKEGSSKQVSVHR-------SQREGTAVPTS---------------------
Pan_troglo : ------------------------------------------------------------------------------------GPSPRKAKEDSSKQVSVHR-------SQRGGTAVPTS---------------------
Xenopus_Si : ------------------------------------------------------------------------------------RPISTGSALLQVKDKRVSVQS-----RGDGASPQTAS---------------------
Danio_reri : ------------------------------------------------------------------------------------YLCSRKDEQKDTDQLSVSR-------SPHTPSRGSAT---------------------
Anoplopoma : ------------------------------------------------------------------------------------SQARNQSSPEERKSVSR---------YQSGAPKPSAA---------------------
Osmerus_mo : ------------------------------------------------------------------------------------TSLRNKIKPEDTESGQLSTS------RLGTAPNLSAA---------------------
Vanderwalt : ------------------------------------------------------------------------------------LSTTNKTYYSTFHSNANSK-------KGNQDKKIPLW---------------------
Kluyveromy : ------------------------------------------------------------------------------------SPVRIKHTVQGYSTFNAQQNAG----NKQQKKKIPLW---------------------
Saccharomy : ------------------------------------------------------------------------------------TSLLRTRLYSNGTGATSGKKD-----DKTRNKPKPLW---------------------
Candida_gl : ------------------------------------------------------------------------------------PRVQFYSSKGKSKGKTSGE-------KKPEHKKIPVW---------------------
Podospora  : ------------------------------------------------------------------------------------TSSSKSEPEIKRRSVTPFNDTG-----SVPWSSLSIL---------------------
Magnaporth : ------------------------------------------------------------------------------------TSANDSNATQRRRAVTPFNDD-----GRVPWTELSGG---------------------
Aspergillu : ------------------------------------------------------------------------------------GGGSGPSSTSKRRNVTVLSDD-----GRYEWGELTGR---------------------
Microsporu : ------------------------------------------------------------------------------------SLNSSKSAQPTRKSITVTSDD-----GTIKWGQLSRG---------------------

                       *       440         *       460         *       480         *       500         *       520         *       540         *       560
Phytophtho : --------------------------------LTPYEKVTATATGGFWLGLIGLGAVGVYFVARELLPNRMSPNGLFSESLDFLSENT------DVTSRLGLP--IHGYGYDHGGHREGRRNRIEHVNLQA-KDGTPRLRIK
Esil_TIM21 : --------------------------------PEEKISIVKTVAGVCVL---GLVVAALSATITELWPSHMAPQSIMSHAHDVFQADP------DTANHFGTP--LKGYGRDNNRHREGRRNFVEHVDHEDPIDKSKRTRVR
Thalassios : --------------------------------LTPGEKVVVGTRLFFWAGAFAFASVCAYYIGKELIPTKMSPNNVFDKATSIIRENA------EVKRRFGEA--FKTYGRDHGGHREGRRNFIEHTEYVSPDDGTKRTRVR
Phaeodacty : --------------------------------LTPGQKVVEASRLTMWGGIAVFAACCAYYIGRELFPTKMSPNRVFDRSFAAIRADS------EVQRRYGDN--LKAYGRDHGGHREGRRNFIEHTEYTDAEDGSKRTRVR
Physcomitr : --------------------------------QKPVTAVEGASYSVVILAGLAVFGFAAYAVLKQLIFEP-KEYKIFGISLARVQNDH------QVMVRIGSP--ITGYGQ-ESRNRAARQR-ISNRIHVD-EDGVEHVEVM
Zea_mays_g : --------------------------------EKPVTFTEGASYSLVILAGLGIAGLAGYAVFKELIFEP-KEYKIFGKALARIQSDS------QVTSRIGHP--ITGYGQ-ETRNRAARQR-IPNKIWTD-EDGVEHVEVN
Ricinus_co : --------------------------------EKPVTFTEGASYSIIILAGLGVAAAAGYAVFKELIFEP-KEYKIFNKALKRIQDDA------QVRVRIGSP--ITGYGQ-ESRNRAARQR-IPNKIYTD-EDGVEHVLVN
Ostreococc : --------------------------------EKPVSVVEGASYGAVGLIGLGIAAGAVWYGASELLVTP-REQKVFNAAMDKLHDDV------RVTVALGSP--MTGYGS-ESRSRSARHR-IAHRVVID-ERGRERLRVQ
Micromonas : --------------------------------EKPVTAVESVSYGAVALVGVGVAVGALWYGIGELLFAP-PAQAAFDEALLLLERDP------RIAVRVGTP--MTSYGN-EGRSRRGRQQ-LAHATEVD-GHGNEWIIAQ
Schistosom : --------------------------------KTVKQVTKDVGYFAIVLGGIVLTGSILYTIIQELFSKK-SPNGVFDDAFKICKTDN------RVLDVFGSA--IKAHGD---PNSRGRRRHVAHDSWYD-DKGRLHMAMK
Strongyloc : MLNELSCRGAYYVFSSLSSKLLQRCTTCSTRSSAVAQTSKDASYAGIIVLGVGITGLMFFTLGKELFSSD-SPNSIYTKAYKVCKKNV------EIQDALGTP--IKGYGE---MTRRKRRRHVSHLEYS--QDGVSFMRMK
Macaca_mul : --------------------------------QKVKEAGRDFTYLIVVLFGISITGGLFYTIFKELFSSS-SPSKIYGRALEKCRSHP------EVIAVFGEP--VKGYGE---VTRRGRRQHVSFTEYV--KDELKHTRVK
Pan_troglo : --------------------------------QKVKEAGRDFTYLIVVLFGISITGGLFYTIFKELFSSS-SPSKIYGRALEKCRSHP------EVIGVFGES--VKGYGE---VTRRGRRQHVRFTEYV--KDGLKHTCVK
Xenopus_Si : --------------------------------HKVKEAGRDFTYFIVVLIGIGVTGGLFYVVFEELFSSS-SPSKIYGEALEKCRSHP------EVIGAFGEP--IKGYGE---TTRRGRRQHVSHMEYV--KDGIKCMRLK
Danio_reri : --------------------------------QKVKQAGKDFTYLIVVLIGLGVTGGLLYVVFQELFSSS-SPSKIYGKAFERCRSHP------EVIGAFGEP--IKGFGE---TSRRGRRQQVSHLEYM--KDGLKYMRLK
Anoplopoma : --------------------------------QKVKEAGRDFTYLIVVLIGLGVTGGLLYVVFQELFSSS-SPNKVYGKAFNKVKLDP------EVIGAFGEP--IKCYGE---TTRRGRRQQVSHQEYL--KDGLKHMRLK
Osmerus_mo : --------------------------------QKVKEAGKDFTYLIVVLIGLGVTGGLLYVVFQELFSTS-SPSKIYGKAFDKTRLNP------EVIGAFGEP--IKCYGE---TTRRGRRQQVSHAEFM--KDGLKHMRLK
Vanderwalt : --------------------------------TRIKSFSTFTASGALVIGATGISTVVIYLILTELFSPS-GDTQLFNRAVTLVEKDEIVRTLLQCDDTDNKKERLKAYGELYADDKWTRNRPIASNKRID-KNGKEHYLMR
Kluyveromy : --------------------------------PRIRAFTTFTFSGILVIGATGLAGVVIYLIGAELFSPS-GDTQIFNRAVSKVENDEVARRLLKCNDTETSTERLKAYGEILTDDRWTRNRPISSTRRID-KEGKEHYLMR
Saccharomy : --------------------------------PQVKSASTFTFSGILVIGAVGISAIVIYLILSELFSPS-GDTQLFNRAVSMVEKNKDIRSLLQCDDGITGKERLKAYGELITNDKWTRNRPIVSTKKLD-KEGRTHHYMR
Candida_gl : --------------------------------HKLKALTSFTASSLLVVGGVGVSGVVLYLILSELFSPS-GDTMLFNRAVTLVEDNEEVRKLLQCNDSSFKKERLKAYGELVTNDRWTRNRPIVSTQKID-KYGKCHHFMR
Podospora  : --------------------------------EKGARASQQTFNFGLVILGLTLTSGVLYVLYTEVFSPS-SRTAYFNRAVDRIKADP------RLIGLLGDGKKIEAFGE-ETGNKWRRARPIASSEVTD-RNGVQHMYIN
Magnaporth : --------------------------------EKTSRAVQQTFNFGMVLVGMALT----------VFSPD-SKVAYYNRAVDRIRKDP------RCLELLGESKSITAFGE-ETSNKWRRARPIASTLRKD-SQGNEHMMIH
Aspergillu : --------------------------------EKVARATQQSFNFVIILAGAALTGGVFYLLYSEVFSPN-SRTWQYEKAVERILDDS------RCTDILGDRREIKAYGE-STSNKWARNRPIAATIEKD-RLGREHLRMN
Microsporu : --------------------------------EKAARATQQSVNFLVILAGAVMTCGVFTFLYLDVFAPD-SKTNQFNRAVSRVKDSE------ECIALLGDSNKIRAYGE-TSWNKWTRNRPIATTIEKD-RKGNEHMRMN
```

```
              *       580         *       600         *       620         *       640         *
Phytophtho : YNIKG--PSGHAYVFAEVNKGMKKNEY--VYLIVQVT----KT--GELLKIIDNRQI-----LSAETKEEQDALRQLLGK----
Esil_TIM21 : FNVEG--PHGHGMAYAEVSNKMESGEW--VYLCVQDL----QT--GHVITLHDNRAL-LMAQAQAGSDEEKNAFRKMLGQ----
Thalassios : FNLEG--QYGNAFVFAEVSKDMPSGEF--VYLLVQDK----RN--GQVITVVDNRSALLAKRMAGGSQEGADVFANLLGGGKK-
Phaeodacty : YNLEG--RFGTAFVFAEVSASMPSGEF--VYILVQDK----SN--GRVHTVVDNRAALTAARLASGSKEAQSAMSQLLGGGGQK
Physcomitr : FYIRG--PQGAGKVYSKMFKDKEDKQWKYTDLIVEIT----SP--TPTRLTLESYMPAVPLPMRAS------------------
Zea_mays_g : FLIRG--PHGAGKVYSEMFKDTDR-TWKFTYLVVDIV----SPPHAKTQLMLESYLPA--------------------------
Ricinus_co : FYIRG--PHGAGKVSAEMFKDKVDKEWKYTYLIVQFL----QP--NQSQLILESYMPAPAPVAA--------------------
Ostreococc : FHARG--ARGSAVVHAEAGLDESTGKWEFQYIIVDVQGA--NP--TRIHVVSPQAQPRRLVAL---------------------
Micromonas : GAVRG--PQGRGAVHLKARKDKESGEWVFAYVAVDV------------------------------------------------
Schistosom : FYIKG--NLASGIVHLEVVEN-ESKEFDYRYLIVETE----GGFSKKQVVLRPLSEAVNAN-----------------------
Strongyloc : FYLEG--PERKATVHLEMKKN-ESGRYEYRYLFVELD-G--YP--KRTIVLEDNR-----------------------------
Macaca_mul : FYIEGSEPGKQGTVYAEVKENRESGEYDFRYIFVEIE-S--YP--RRTIIIEDNRSQDD-------------------------
Pan_troglo : FYIEGSEPGKQGTVYAQVKEVWES--WDVGSEVLSAVLFKFWK--GK-------------------------------------
Xenopus_Si : FYIEGLEPRKQGTVHTEVKENPESGKYEFQYIFVEID-T--YP--RRTIVIEDNRRQS--------------------------
Danio_reri : FYIEGSEPGLRGTVHSESKENPETGKYEFRYIFVEID-T--YP--RRTIVIEDNR-----------------------------
Anoplopoma : FYIEGSEPGRKGTVHSESKENPETGKYEFRYIFVEVD-T--YP--RRTIIVEDNR-----------------------------
Osmerus_mo : FYIEGEEPGRKGTVHSESKENPETGKYEFRYIFVEVD-T--YP--RRTIVVEDNR-----------------------------
Vanderwalt : FHVES--KQKMGLVHIEAVESEKRYQPDFVSVYIDIP----GE--KRYYLIKPKINR---------IVRPKGFLGINWGPRKQ-
Kluyveromy : FHVES--KQKMGIVHVEAKESDVNYQPDFVSMYLDIP----GE--KRHYLIKPKLSI----------VKPKGFLGVNWGPRKD-
Saccharomy : FHVES--KKKIALVHLEAKESKQNYQPDFINMYVDVP----GE--KRYYLIKPKLHP---------VSNSKGFLGIRWGPRKD-
Candida_gl : FHLES--KKKLGLVHVEAVDSEQNYKPNFVSMYIDIP----GE--KRFYIIRPKLRQ---------VVRPKSIFGFPWGSRKE-
Podospora  : FNLKG--PKGTGTAFVHLFKPVGGKQWEYRYFYVDVK----GQ--QRIYLENAEAAATLAAKERSKTD-GIKFLGIRW------
Magnaporth : FNLEG--SKGRGMANMHLIKRVGAEDFEYRYFYLDVV----GH--QRIYLENSEATG------RDGSG-KKTIFGIRFS-----
Aspergillu : FHVEG--PRNQGVVHVHMIKPLDKNEWEYQLLALDVK----GH--SRVILEQAREKP--------GVGQALKIFGIQWR-----
Microsporu : FHVSG--PLNDGVVLVHLVKLAGQHDFEYHLLALDVK----GH--RRIYLENADAAK------KAAAKAGSTIFGIQWR-----
```

(c) Tim50 amino-acid alignment (p. 5-8)

```
                      *        20         *        40         *        60         *        80         *       100         *       120         *       140
Saccharomy : ------------------------------------------------------------------------MLSILRNSVRLNSRALRVVPSAANTLTSVQASRRLLTSYSSFLQKE------------------------
Cyanidiosc : ---------------------------------------------------------------------------------------MLVDRYAGLRALR------------------------------------------
Esil_TIM50 : -----------------------------------MLLGRVGTLLPSCSGRVATRRFLAKKAAGGGGGGKKGSGTPKKGPAAIKARGAMPVSKAGKQGSKTGKTGGAAAREAAATTTSTAKAAAAEAAASVKPPPVG-----
Arabidopsi : ---------------------------------------------------------------------------------------MASIVLRSRLLPRLAKLRSRNLRCFSA----------------------------
Zea_mays_g : ----------------------------------------------------------------------------------------------------------------------------------------------
Phaeodacty : ----------------------------------------------------------------------------------------------------------------------------------------------
Phytophtho : -------------------------------------------------------------------------------------MMLKLVLRAQRGMRAFAGIGATRK---------------------------------
Phytophtho : ---------------------------------------------------------------------------------------MMLRPVLRNAQR-------------------------------------------
Magnaporth : -----------------------------------MLSRLAIRTLRAPALQGLGRTTAARQTTSLLAARTWTRG--------------MAKNNKPAPTPSQQTSK-SNAPSEEQASSPAPKQSENATK--------------
Aspergillu : -----------------------------------MLRRAILPLTRPSGLVSAPRLSALPVSHS----RCYAKGSKPKTPYKLPESVKSSKPEQP-AKPSQQEQYAAEQAEFETTSDPQANTANTTSQASSS----------
Penicilliu : -----------------------------------MLSRSVLPLARARPVPSALRSPLTASPYP----RWYAQGNKPRTPYVLPN--QKAKPAQSKATPSQKNTS-TKSQPTQQKSKPTVVGTTSKAKAAASASSGASTNAS
Talaromyce : -----------------------------------MLSRSVLPLARARPVPSTLRS------YP----RWYAQNNKPRTPYVLPN--QKPKPAQPNPARSQSNTSTTNNQSTQPKTKPTVVGASSGAKATESVPPDAASSKT
Ostreococc : -----------------------------------------------------------------------------------------------XXTW-------------------------------------------
Micromonas : ---------------------------------------------------------------------------------------MFTRVAARALA--------------------------------------------
Chlamydomo : MVLPVRAKKIVLASVVVVTVLTLVNYHQIVHFAETALPHRLPEPFTHNHIFYEPSGGWLVGPAAPCPQRWIPGIWVSKSQRQHTGGVLPLTEDAQQEVFQAMNPPGCGGAKFLVHRVEPQGFAAAIHELGVALQLALDSGRV
Ixodes_sca : ---------------------------------------------------------------------------------------MAAVGRGGMCVWHGLRPKICS----------------------------------
Xenopus_(S : -------------------------------------------------------------------------------------MAASLVPVCGRSVRLLRVL--------------------------------------
Pan_troglo : MASALSLGNKCDPFLRCVLCRGGGALQGPRGRGPDDFESQLSPPGPARRLVRGKRACGNPPDAFGLSRASVHQPLPRVSIGCSSGPGRAKRERVGGAAWRQRKMA-------------------------------------
Homo_sapie : -------------------------------------------------------------------------------------------------AWRQRKMA-------------------------------------
Bos_taurus : --------------------------------------------------------------------------------------------------WRQRKMA-------------------------------------
Mus_muscul : ----------------------------------------------------------------------------------------------------------------------------------------------
Nematostel : ----------------------------------------------------------------------------------------------------------------------------------------------
Strongyloc : ----------------------------------------------------------------------------------------------------------------------------------------------
```

```
                    *       160         *       180         *       200         *       220         *       240         *       260         *       280
Saccharomy : ------------------------------------TKDDKPKSILTDDMLFKAGVDVDEKGQ--------------------GKNEETSGEGGE-----------------------------------------------
Cyanidiosc : ------------------------------------PIGSALLRPGRVLRCLTSGNGGRAAGE--------------------AGSKPTSGPIHP-----------------------------------------------
Esil_TIM50 : ------------------------------------GGGGFTKSEMLAARKARKASVTAKTAA--------------------AAATAKAAAPAA-----------------------------------------------
Arabidopsi : ------------------------------------EASSTNSTSRYSGVTSTQSMFSDFPPP--------------------NQPPPPPPPQVE-----------------------------------------------
Zea_mays_g : --------------------------------------MSRVAGSRLLPRISALSFCTASPAT--------------------AAASSSSSTAAV-----------------------------------------------
Phaeodacty : ----------------------------------------------------------------------------------------------------------------------------------------------
Phytophtho : ------------------------------------LMLAAEASAPSSVAEASSIFAKVGRDI--------------------PLKVVPRAERRA-----------------------------------------------
Phytophtho : ------------------------------------GAALGTTRRLMLQAEASSVLAKVGRDI--------------------PLKVVPRAERRA-----------------------------------------------
Magnaporth : --------PEASDAQPEQ--------VPFNLPDLTKGLPSTFEYEASGADKASKMALEGVASA--------------------GG---------R-----------------------------------------------
Aspergillu : --------PESSPSQSEQDA------PQRPLPDLTQGIPSTLAAELEARSKKSGSGTLNLTED--------------------PSRFEEDYSD-D-----------------------------------------------
Penicilliu : E--TPKSEPDVSPEQPEFETVAQAEKTPQPLPDLTQGIPSTLGAELDAARKGRKTTSLNLTED--------------------PS--QDDYEDEG-----------------------------------------------
Talaromyce : EPKKPLSEPDVAPEQPEFDTAAQPEKPQKPLPDLTQGIPSTLGAELEAARKGRKTTALNLTED--------------------PS--QHDYEEED-----------------------------------------------
Ostreococc : ------------------------------------IPTRALASEAEGEAAKTGTLSDRLGTV--------------------ALVVGTLAGSVV-----------------------------------------------
Micromonas : ------------------------------------GTLLGSGARVAQLSRGTSMGGETAPAT--------------------AAAIARRSLQRR-----------------------------------------------
Chlamydomo : LVEAPGSPHAEGVPECGALRNTLDSCYLLPFSSCRPSEADIIAALNTTQSQASGSAPQGVTVPREPRIIFTNTAMVEAARHRAPKRFEERLQQTAVDHQKHRYWWRAQAAAYLLRPNMGTLANLGKRRMQELRGPEPSPGCI
Ixodes_sca : ------------------------------------AGVPRLGPVRFTSTSGGGG----------------------------------AAAPTG-----------------------------------------------
Xenopus_(S : ------------------------------------GQRTELRYRAFSSEQGAEGGLT-------------------------GAVLQEKLQRRE-----------------------------------------------
Pan_troglo : ------------------------------------ASAAVFSRLRSGLRLGSRGLCTRLATP--------------------PRRAPDQAAEIG-----------------------------------------------
Homo_sapie : ------------------------------------ASAAVFSRLRSGLRLGSRGLCTRLATP--------------------PRRAPDQAAEIG-----------------------------------------------
Bos_taurus : ------------------------------------ASAAVFLRLRSGLRQGARGLCARLATP--------------------PPRAPDQAAEIG-----------------------------------------------
Mus_muscul : ----------------------------------------------------------------------------------------------------------------------------------------------
Nematostel : ----------------------------------------------------------------------------------------------------------------------------------------------
Strongyloc : -------------------------------------------------------------------------------------MPFPSGCDGE-----------------------------------------------

                  *       300         *       320         *       340         *       360         *       380         *       400         *       420
Saccharomy : ---------DKNEPSSKSEKSRRKRQTS------------------------------------------------------------------------------------------------------------------
Cyanidiosc : ---------EEGAAAASDGAAESKRPQH------------------------------------------------------------------------------------------------------------------
Esil_TIM50 : ---------GAGAGRTGGAGAAASAAGA------------------------------------------------------------------------------------------------------------------
Arabidopsi : ---------AAAAAATG-------KERK------------------------------------------------------------------------------------------------------------------
Zea_mays_g : ---------AAAAATAGDPSSQPPQTAR------------------------------------------------------------------------------------------------------------------
Phaeodacty : ----------------------------------------------------------------------------------------------------------------------------------------------
Phytophtho : ---------ARKAAKHSADAATGHKVVR------------------------------------------------------------------------------------------------------------------
Phytophtho : ---------ARKAAKHSADAAEHKVVRT------------------------------------------------------------------------------------------------------------------
Magnaporth : ---------GKGELPASAYVSSSDRRRQ------------------------------------------------------------------------------------------------------------------
Aspergillu : ---------GRGDIPKGGYESSLDRKRA------------------------------------------------------------------------------------------------------------------
Penicilliu : ---------AGGGRSSDAYESSLDRRRA------------------------------------------------------------------------------------------------------------------
Talaromyce : ---------GGGGRSSDAYESSLDRRRA------------------------------------------------------------------------------------------------------------------
Ostreococc : ---------GASTYASTTEELRREVEAN------------------------------------------------------------------------------------------------------------------
Micromonas : ---------GLSTESASKAGAEEQGVMA------------------------------------------------------------------------------------------------------------------
Chlamydomo : TVIARSGKSGDGAAASDFKDADYDERAAKLRALDPTRFNDHIFLSASSGHTLSYFANGTATADAGAHRWHTGYVAGLKHFGGGMSLRLRKAGGGHDLDAVPKDAVYESMLNTLLELDLALECSGFVGSIDSHWVRLVDEMR
Ixodes_sca : ---------ASGSTSGGSSGRAADDFSQ------------------------------------------------------------------------------------------------------------------
Xenopus_(S : ---------NEASSDSSGSEEKQKKQKE------------------------------------------------------------------------------------------------------------------
Pan_troglo : ---------SRGSTK--TQGPQQQPGSE------------------------------------------------------------------------------------------------------------------
Homo_sapie : ---------SRGSTK--AQGPQQQPGSE------------------------------------------------------------------------------------------------------------------
Bos_taurus : ---------SRAGTKAQTQGPQQQRSSE------------------------------------------------------------------------------------------------------------------
Mus_muscul : ----------------------------------------------------------------------------------------------------------------------------------------------
Nematostel : ----------------------------------------------------------------------------------------------------------------------------------------------
Strongyloc : ---------KQGTNITGTCQPQLDEHGK------------------------------------------------------------------------------------------------------------------
```

```
                        *       440         *       460         *       480         *       500         *       520         *       540         *       560
Saccharomy : -------------------------------------------------------TDIKREKYANWFYIFSLSALTGTA-IYMARDWEPQESEELKKDI----DNGYTLSLMYKRFKAR-----------------------
Cyanidiosc : -------------------------------------------------------SRWRALFRPGRLLGFV---ALGSA-ATVLTFWVVDPEGTVARLE---------------RLRHE-----------------------
Esil_TIM50 : -------------------------------------------------------GGAKGQGKWGWGTKLV-AGTSVGL-AALGIAWQLKP-DEMRKLL-----DDSPIDHFFI----------------------------
Arabidopsi : -------------------------------------------------------GLKYLGYALLWALTGA----TAAT-GYASFAYTIDEVNEKTKAFRESATKTPVIKSSGIDVIDKYQTKLYSAAMTGSARAIDKYLEL
Zea_mays_g : -------------------------------------------------------NPWGALKFAAFAAVSA---AVGGT-GYVSYAYSLEEVDQMTRELRKKSKQPIPEDASGFERFQA---MAYSAAMKVPVAAIEVYLDV
Phaeodacty : ----------------------------------------------------------------------------------------------------------------------------------------------
Phytophtho : ------------------------------------------------------TSSLPARISFALLAGSI-----SGS-IVWNFVLDDGIKSRVKETL-----GATFLGDIYAIIAKK-----------------------
Phytophtho : -------------------------------------------------------SSLPARISFALLAGSI-----SGS-ILWHFVLDDGIKTRITETL-----GATVLGDVYAFLAKK-----------------------
Magnaporth : -------------------------------------------------------KVAQWVFYGFVAGGLF------GV-VFLGRNWEDEEERAKHADV----PDGWTPVLWWKRAMAR-----------------------
Aspergillu : -------------------------------------------------------RMAK-LMYALFLLGSV-----GGM-AYLGRNWDTVEEENAHPDV----PSGWSFGLWYNRIKAR-----------------------
Penicilliu : -------------------------------------------------------RLAN-VMYGILLGGIL-----GGT-VYMGRNWDSEEEARLHKDA----PSGWGIRLFYDRIKAR-----------------------
Talaromyce : -------------------------------------------------------RVAN-VMYGILLGGLI-----GGT-VYMGRNWDSEEEAQAHKDA----PSGWGLGLFYNRIKAR-----------------------
Ostreococc : -------------------------------------------------------EHVPKALRETPLGGVY-----GGA-IERLLALRE-----------------------------------------------------
Micromonas : -------------------------------------------------------TVVDTVSTMGTAVGCL----VGAT-IGVSYYTHSTKQLEEALEKGEHVPGALKDTPLKQALDYA----------------FGHLLEF
Chlamydomo : SVVRCSADAPYIDVGHDDPRQMNLHCVLDEVAQARLKRIKSQLEATGELPVEPPSGGVWGAIRMLWRGALLGATGLGATAAYYTYAYDTKELVSIVKQTRAELKAEEKPNPLRKLWCDG----------------MQRYLVF
Ixodes_sca : -------------------------------------------------------RA---MKYTFVAFGAI---FTGVA-GYLVVSWGAPSVDETGKEM-------------------------------------------
Xenopus_(S : -------------------------------------------------------NAANAKRFLLRAAGLL---GVAGS-GSLVYIFGSNSVDEQGNKI----PDEFDSDPPVVQQIRR----------------------T
Pan_troglo : -------------------------------------------------------GPSYAKKVALWLAGLL---GAGGT-VSVVYIFGNNPVDENGAKM-------------------------------------------
Homo_sapie : -------------------------------------------------------GPSYAKKVALWLAGLL---GAGGT-VSVVYIFGNNPVDENGAKI----PDEFDNDPILVQQLRR----------------------T
Bos_taurus : -------------------------------------------------------GPSYAKKVALWLAGLL---GAGGT-VSVIYIFGNNAVDENGAKI----PDEFDNDPILVQQLRR----------------------T
Mus_muscul : ----------------------------------------------------------------------------------------------------------------------------------------------
Nematostel : ----------------------------------------------------------------------------------------------------------------------------------------------
Strongyloc : -------------------------------------------------------VIVDEFEDDFIVFGYL---------------------KRAYREMNTYRTVSFKQKIIRIRHF-------------------------

                      *       580         *       600         *       620         *       640         *       660         *       680         *       700         *
Saccharomy : ---FNSMFTYFQEPPFPDLLPPPP-PPPY-----QRPLTLVITLEDFLVHSE---------WSQKHGWRTAKRPGADYFLGYLS-QYYEIVLFSSNYMMYSDKIAEKLDPIHAF-VSYNLFKEHCVYKDGVHIKDLSKLNRD
Cyanidiosc : ---LESRIRYYVEPSREKLLPDPVAPFPGS----LPPRTLVLDLDETLVHSD---------WTRSTGWRTSKRPGVDAFLAYMA-QFYEIVVFTSALPGYADPILDRMDP-NGY-ITHRLYRHETKYRDGLHMKDLAKLNRD
Esil_TIM50 : --WFMGKWALYSSPVKDKLLLDCP-LPPGA----LPPPTLVLDLEGTLLGTI---------YTRKKGWRVAKRPGLDAFLKEMS-QLYEIVVFTDSMGGLADEWITQMDP-QGT-ISQRVYRDGTRYIDGKYVKDLSALNRP
Arabidopsi : REIVEEQVKGFTEPLSEKLLPD---LHPAE----QHVFTLVLDLNETLLYTD---------WKRERGWRTFKRPGVDAFLEHLG-KFYEIVVYSDQMEMYVLPVCEKLDP-NGY-IRYKLARGATKYENGKHYRDLSKLNRD
Zea_mays_g : RSQIEDQIRGFSEPVSDKLLPD---LAPQE----QHVLTLVLDLNETLVYSD---------WKRERGWRTFKRPGVDAFLEHLG-RFYEIVVYSDQLSMYVDPVVDRLDP-KGN-IRHRLSRVATKYENGKHYRDLSKLNRN
Phaeodacty : ---------PLAKPAHEKLLPDWS-QLPNVPQDIPVPHTLVLDLENTLVSST---------WDRRYGWRHAKRPGVDKFLRELA-QYYEIVLYSPSIDGIADPVVTSLDK-DGC-IMHRLYREATYYTGGVHVKDLNRLNRP
Phytophtho : ---VEETVKPFTDPSRQKLLPDW--PIPQVPADTPPVPVLVLDLEDTLVHSE---------WSRKHGWRHAKRPGVDEFLETLC-QYYEIVIFSQNYG--AEEIVQKLDP-KQC-ALHILSRDATRYLNGAHVKDLSNLNRD
Phytophtho : ---VEETVKPFTDPSRQKLLPDW--PIPQVPADTPPVPVLVLDLEDTLVHSESLMVYRFIILQRKHGWRHAKRPGVDEFLETLC-QYYEIVIFSQNYG--AEEIVQKLDP-KQC-ALHVLSRDATRYLNGAHVKDLSNLNRD
Magnaporth : ---MGDTVSYYQEPAFEKLLPD---PDPVN----SPPYTLCISLEDLLVHSE---------WTRDHGWRVAKRPGVDYFIRYLS-QYYELVLFTSVPYGIAEPLWRKMDP-FRF-VQWPLFREATKYVDGKIVKDLSYLNRD
Aspergillu : ---MGDFTSYYKDPAFPKLLPD---EDPNL----RQPYTLVLSLEDLLVHSE---------WSREHGWRVAKRPGVDYFLRYLN-QYYELVLFTSVPSMMADQVLRKLDP-YRI-IRWPLFREATRYKDGEYIKDLSYLNRD
Penicilliu : ---MDSFTSYYRDPAFDKLLPE---EDPSL----RQPYTLVLSLEDLLVHSE---------WTREHGWRVAKRPGVDYFLRYLN-QYYELVLFTSVPSMMADQVLRKLDP-FRI-IRWPLFREATKYKDGEYVKDLSYLNRD
Talaromyce : ---LDNLTSYYRDPAFEKLLPD---EDPSL----RQPYTLVLSLEDLLVHSE---------WTREHGWRVAKRPGVDYFLRYLN-QYYELVLFTSVPSMMADQVLRKLDP-FRI-IRWPLFREATKYKDGEYVKDLSYLNRD
Ostreococc : --WMDDQSHNYLDPISDKLLPDHP-PQAEY----I-PHTLVLDLDDTLINSN---------WKRERGWRVFKRPGVDGFLAHMA-QFYEMVIFTDQLMTYGDPIIERLDP-TRY-VTHRLYRESAQYKNGEYIRDLSKLNRD
Micromonas : RQWADGLRHQYLDPVSDKLLPDHP-PNAVY----I-PHTLVLDLDECLIKSD---------WRRERGWRTFKRPGAGDFIKHMA-QFYEVIVFSDQTSMYVDPILERLDP-QRF-LAGRLAREANQYVDGEYLRDLTKLNRD
Chlamydomo : RHDMESRIKDFTDPATDKLLPD---QHPAM----KGAITLVLDLDEVLVWKE---------WTRQRGWSIYKRPGVQEFLLEMA-QYFEIVVYTDEPYTYADPILNKFDP-QRVAVMHRLYRTDTQYLDGKHVRDLSKINRD
Ixodes_sca : ----------IQEPSRDKLLPDPL-TEPYF----QPPYTLVLEMTGVLVHPD---------WTYQTGWRFKKRPGVNLFLQQVGPPLFEVVVYTSEQGFTAYPILDSLDP-QGF-IMYRLFRDATRYTKGHHVKDLSCLNRD
Xenopus_(S : YKYFIDYRQMIIEPTSPKLLPDPL-KEPYY----QPPYTLVLELTDVLLHPE---------WSLSTGWRFKKRAGIDNLFQQLA-PLYEIVIFTSETGLTAFPLIDNVDP-HGF-VSYRLFRDATRYTDGHHVKDISCLNRD
Pan_troglo : ----------IIEPTSPCLLPDPL-QEPYY----QPPYTLVLELTGVLLHPE---------WSLATGWRFKKRPGIETLFQQLA-PLYEIVIFTSETGMTAFPLIDSVDP-HGF-ISYRLFRDATRYMDGHHVKDISCLNRD
Homo_sapie : YKYFKDYRQMIIEPTSPCLLPDPL-QEPYY----QPPYTLVLELTGVLLHPE---------WSLATGWRFKKRPGIETLFQQLA-PLYEIVIFTSETGMTAFPLIDSVDP-HGF-ISYRLFRDATRYMDGHHVKDISCLNRD
Bos_taurus : YKYFKDYRQMIIEPTSPCLLPDPL-REPYY----QPPYTLVLELTGVLLHPE---------WSLATGWRFKKRPGIETLFQQLA-PLYEIVIFTSETGMTAFPLIDSVDP-HGF-ISYRLFRDATRYMDGHHVKDISCLNRD
Mus_muscul : ---------MIIEPTSPCLLPDPL-REPYY----QPPYTLVLELTGVLLHPE---------WSLATGWRFKKRPGIETLFQQLA-PLYEIVIFTSETGMTAFPLIDSVDP-HGF-ISYRLFRDATRYMEGHHVKDISCLNRD
Nematostel : ---------MFVEPSSTKLLPDPL-PEPYI----QPPYTLILEMTDVLVHPE---------YDRKSGWRFRKRPGVEFFLNQLA-PLFEIVVFTHEVGFSASPVIDGIDP-HQM-IMYRLFRDATKYIKGTHVKDLSGINRD
Strongyloc : ---ISHFVQMIAEPSAEKLLPDPL-VEPYY----QPPYTLVLEMKDILVHPE---------WTYANGWRFKKRPGVEYFLQQAGPPLFEIVIYTSEQGFTAFPILDSLDP-KGC-IMYRLFRDATKYVNGHHVKDLSSLNRD
```

```
                    720         *       740         *       760         *       780         *       800         *       820         *       840         *
Saccharomy : LSKVIIIDTDPNSYKLQPENAIPMEPW---NG-EAD---------DK-LVRLIPFLEYLATQQTKDVRPILNSFEDK-----KNLAEEFDHRVKKL--KDKFYGDHKSGGNWAMTAL-------------------------
Cyanidiosc : LRRTIIIDNDPRVFALQSENGIEIAPW---NGTDPD---------DKELLRLTAFLEWVVRNDVADVRPIIATVRNCD----RTTSRSFAERFEAC--RQQVESVLASQKLAAVAGA-------------------------
Esil_TIM50 : LEQTLIIDDNADCISMQPENAIKVKAFSLEDGSDPTA--------DTALYDLAPFLRALATQGVADFRDVLRPHVGEDS---NAVVADFRSKVNAVRQKEDAEKSKGLGGLVRQIAPVVGAGGPAAGMGGMLTSKDIVGDAP
Arabidopsi : PKKILFVSANAFESTLQPENSVPIKPY---KL-EAD---------DTALVDLIPFLEYVARNSPADIRPVLASFER------KDIAKEFIDRSIEY--QKRKQGQLGQGRFWRR----------------------------
Zea_mays_g : PAQVLYISAHAKESSLQPENSVEIKPW---KL-END---------DTQLLDLIPFLEYVAMARPSDIRAVLASYQS------GDIAAEFIER------SKEHQRRMQEQKK-------------------------------
Phaeodacty : LNRMVVIDDDPSEVQFNPENLIRVKPY---AD-PTDRT-------DNTLERILPFLVEIAREGYNDVPGLLRQYEGMDA---DQIADEQDRRIHELRTHRERRSQQGLGALARG----------------------------
Phytophtho : LRQVVILDDDPAAYQLQPENAIPVTPFT--NGRDRD---------DHELKDLIPFLKALASERVPDFRQVIGEFRDEDGVV-RDLATKYGARVHQLEMQKEQKKKKGFGGFVRGRLS-------------------------
Phytophtho : LRQVVILDDDPAAYQLQPENAIPVKPFT--NGRDRD---------DHELKDLIPFLKALASERVPDFRQVIGEFRDDDGVV-RDLATKYGARVHQLEMEKEQKKKKGFGGFVRGRMS-------------------------
Magnaporth : LSKVIIIDTNPEHVSAQPENAIILPKW---TG-DAQ---------DKDLVALIPFLEYIHTMQYPDVRKVLKSFEG------KNISEEFARREAIA--RKKFQEQLEQNRKKHPNKPGAGVFGALGNAFGLKPSKMSMMVPV
Aspergillu : LSKVILIDTKEEHARLQPENAIILDKW---NG-NPK---------DKTLVALIPFLEYLAGMGVDDVRTVLKSFEG------QSIPIEFAKREKAM--RERFEKELAEEQKKRPRSG----MGSLASALGLKSSART----L
Penicilliu : LSKVILIDTVPGHAREQPENAIILPKW---KG-DTK---------DKSLVALIPFLEYVAGMNVEDVRPVIKSFEG------TYIPAEFAAREKVM--REKFQKQLEEEQKSRPRRG----VGSLAALLGLKPQS-S----I
Talaromyce : LSKVILIDTVPAHAREQPENAIILPKW---KG-DPK---------DKSLVALIPFLEYVAGMNVEDVRPVIKSFEG------TYIPAEFAVREKVM--REKFQKQMEEEQKSRPRRG----VGNLAALLGLKPQS-S----I
Ostreococc : MGQILYISSKPRSAELHPANVIPIKPWTYEDG--SK---------DTALLDLMPFLESIVRLNVQDVRVVLDSYKKEMSATGKDIPTIFRERQLAL--QRRRRDKLANQTKMSR----------------------------
Micromonas : VGMVLYITARPKT-SMQQENVVQISPY---IV-DSEGRTAGGDGPDTTLLDLMPFLESIVRLNVKDVREVLASYKQEQEATGKSVPEIFRSRQYRF--QKQQRQKTVTKPA-------------------------------
Chlamydomo : LNHVLMISAKPEAWALQPENTLKLKPW---KG-QPG---------DTGLIDLIPFLQFLAMRRVKD----------------------------------------------------------------------------
Ixodes_sca : LSKVILVDWSEEACSLQPRNALKLRKW---DG-GDE---------DRTLLDLAQFLRTIGTSEVEDVRTVLDYYRQF-----EDPLVAFKENQKRL----QEEKQLASIEAVPSQ---------------------------
Xenopus_(S : PSHVVIVDCKREAFKLQPYNGLAIKKW---DG-SSE---------DRALYDLTAFLKTIAVSGVSDVRSVLENYALE-----EDPLEAFKRRQTQL--EQEEQQRLADMSQLSKRQ--------------------------
Pan_troglo : PARVVVVDCKKEAFRLQPYNGVALRPW---DG-NSD---------DRVLLDLSAFLKTIALNGVEDVRTVLEHYALE-----DDPLAAFKQRQSRL--EQEEQQRLAELSKSNKQ---------------------------
Homo_sapie : PARVVVVDCKKEAFRLQPYNGVALRPW---DG-NSD---------DRVLLDLSAFLKTIALNGVEDVRTVLEHYALE-----DDPLAAFKQRQSRL--EQEEQQRLAELSKSNKQ---------------------------
Bos_taurus : PARVVVVDCKKEAFRLQPYNGVALRPW---DG-NSD---------DRVLLDLSAFLKTIALNGVEDVRTVLEHYALE-----EDPLEAFKQRQSRL--EQEEQQRLAELSKSSKQ---------------------------
Mus_muscul : PARVVVVDCKKEAFRLQPFNGVALRPW---DG-NSD---------DRVLLDLSAFLKTIALNQVEDVRTVLEHYALE-----DDPLEAFKQRQSRL--EQEEQQRLAELSKSNRQ---------------------------
Nematostel : LKKVIVIDCNKAATELNERNAIILKKW---EG-NPA---------DTTLVDLLPLLQTIATSGVDDVRAVLDFYRQE-----DDIVAAFKRNQARL--REAEQARLQKLEQQNKQ---------------------------
Strongyloc : LSKVIIVDCNAKSYQLQPKNALGLKKW---EG-NDD---------DRTLFELAAFLRTVAASGVEDVRTVLEFYQQF-----EDPMEAFRINQARLQVRSEQEEEAKRIAEAKEKGG-------------------------

                  860         *       880         *       900         *       920         *       940         *       960
Saccharomy : ---GLGNSLGGSTKFPLDLIHEEGQKNYLMFMKMIEEEKEKI---------RIQQEQMGGQTFTLKDYVEGNL---PSPEEQMKIQLEKQKEVDALFEEEKKKKKIAESK
Cyanidiosc : -----------------------------ATASSSSADGHLSGNGVRAPVRGLGIFAIPRRDPAEQSATTTSE---ASSPAAPAQPVPDQSIWTRLRR------------
Esil_TIM50 : ETLSPGMAAAAAATAGGKGGGKGGGGSKPLAEKQKGGLWKSLQEGNKEREEDFMRRNEAFQRVMEKRMAREKA---KRDEQAQQQ-------------------------
Arabidopsi : --------------------------------------------------------------------------------------------------------------
Zea_mays_g : --------------------------------------------------------------------SGRIW---RR--------------------------------
Phaeodacty : --------------------------------------------------------------------------------------------------------------
Phytophtho : -----------------------------------------------------HHPASPTGGVAASGFSSGPHV------------------------------------
Phytophtho : -----------------------------------------------------HHPAPPNGGVAASGFGGGH--------------------------------------
Magnaporth : EGEESVADALAQGKMLQDIARERGQRQYERIDKMVRENGEKWLKEEAEMMEKMQAEAMANMKTGFMGWFGSSEQKDGAKDEPKKA-------------------------
Aspergillu : DGEQLPSAGLQEGKMLWDQIRERGQKNYELIEKEIRENGEKWLAEMAAEEEKLRQEQMESMKGSLTGFFGG-----GKKE------------------------------
Penicilliu : DGQ----GA-SEEKMLWDQIRERGQKNYEMMEQEIRENGETWLKMMAEEEEKARQEQMKNMKGSLTGFFGASG--EGEKK------------------------------
Talaromyce : DGQ----AAGSEEKMLWDQIRERGQKNYEMMEQEIRENGETWLKMMAEEEEKARQEQMKNMKGSFTSFFGATG---GEKK------------------------------
Ostreococc : -------------------------------------------------------------GYDVGKRAQREA---RAAA------------------------------
Micromonas : --------------------------------------------------------------------FSRGW-------------------------------------
Chlamydomo : --------------------------------------------------------------------------------------------------------------
Ixodes_sca : -----------------------------------------------------------------RSWTKGLW---KRN-------------------------------
Xenopus_(S : -------------------------------------------------------------TLSLGSFTGRLW---PRSKQQ----------------------------
Pan_troglo : -------------------------------------------------------------NLFLGSLTSRLW---PRSKQP----------------------------
Homo_sapie : -------------------------------------------------------------NLFLGSLTSRLW---PRSKQP----------------------------
Bos_taurus : -------------------------------------------------------------NLFFSSLTSRLW---PRSKQP----------------------------
Mus_muscul : -------------------------------------------------------------GLSFGSLASRLW---PRSKQP----------------------------
Nematostel : -------------------------------------------------------------GRSWGGMFGR---------------------------------------
Strongyloc : -------------------------------------------------------------SSLVGGWTRGLI---GRR-------------------------------
```

(d) Sam50 amino-acid alignment (p. 9-16)

```
                      *        20         *        40         *        60         *        80         *       100         *       120         *       140
Neorickett : ----------------------MRKKLFLVWLLFLSRFCFADEVVEIRISGNKRVAETTIHGLLHVEVGQDVTSDELNAVFKRLTASRLFSSVELDLTAG-ILEVKLQENPILRNVVVKGNKLLSRAAIDKILVYKKDAIFD
Rhizobium_ : --MKAGSRFLNAVSAFALSASMVATGTGAAL-VASTSVAQAAVISRVEVRGATRVSPETVRANITIVPGKSFSNADIDASVKRLYATGYFSDVSISISGG-TLVVNVSENQLVNQVVFNGNRKIKDDKLQGVVRTQPLGPYS
Rickettsia : ----------------MKIISISKLTILLLTIFYYHISFADYVIRKITIEGNHRVERSTIESYLKLNVGETYNNSKEDEAIKRLYATSLFRNINMYITNDGNLIVNVTETPFISSVVFSGNSKIKTNILAKEIYTMSGESLS
Rickettsia : ----------------MKIISISKLTILLLTVFYYHISFADDVIRKITIKGNHRIERSTIESYLKFNVGETYNNSKEDEAIKRLYATSLFKNINMYITNDGNLIVNVTETPFISSVVFSGNSKIKTNILAKEIYTMSGESLS
Magnetospi : MSRFQGNGFTNLRGAWMRFVLWTAVLLGILVGVMPASAQEGGRIRSISILGTQRIEVETVKSYMTVAEGDLYDTDRVNRSLKALFNTGLFQDVAIRREGD-QLLVRVVENPIINRIAYEGNNRIKEEQLNTEVQLRPRTVYT
Gluconacet : MPTKRSTLLASVCLIPLFFAGAAEARQGVATRGPVAHTPTGGVIESIDISGNDRIETNTVLSYMVVQPGDPFNQDQLDRSLKTLYATGLFRDVTLHRAGN-VLQVHLVENPIVNRIVFEGNHAAKDEDLRKVIALRPRAVFS
Chlamydomo : ----------------------------------------------MTSSEAEPAPATAASASPAAADASGSSAG----ASGAAAT---------------------PAAGGPHGVVD--------------YE--------
Phaeodacty : --------------------------------------------------MNQAQLQQKQERLRQLQEEERQLRLQLQVQEHAATY--------------------------------------------------------
Thalassios : ----------------------------------------------MNNNHQQRRQGGDASSASSAISHQQRLQRHRQQQQQQMME---------------------KLNQLKAQYVSDYSLPLNYPANVTSLL--------
Saccharomy : --------------------------------------------------MTSSSGVDNEISLDSPMPIFNESST-------------------------------------------------------------------
Ectocarpus : ----------------------------------------------------------MVIPLD------------------------------------------------------------------------------
Phytophtho : ---------------------------------------------------MASTNSEAPEPTAE-PPHPW-----------------------------------------------------------------------
Phytophtho : ---------------------------------------------------MASTRSEAPKAAAE-PPHPW-----------------------------------------------------------------------
Phytophtho : --------------------------------------------------MASTNSSETPKPAAEDPPHPW-----------------------------------------------------------------------
Physcomitr : ---------------------------------------------------MNIMSEKLV----------------------------------------------------------------------------------
Zea_mays_A : -----------------------------------------------MAIAADSAADQDPNPIPNEAAGESVELPAYTDHGEPNAD---------------LADVDYEDEEELDGVTAEAAARDK-------VE--------
Sorghum_bi : -------------------------------------------MAAAADSAAGQNPKDDERHVLTADSDARFSEEEYEEDGEEEYE-------------------EEEEEEELDGPAAEAAERERVQSVFRRLS--------
Oryza_sati : ---------------------------------------------------MATAADQNPNDAEHREAAAGANAAAEEYEEEDEEE---------------------EEVELDGPAAVAAEREK--------VQ--------
Arabidopsi : --------------------------------------------------MENPAEKPDPNPSKPKIESEDEREELGDINGDEEEE---------------------EEYEEEDDGKPRTREDAIADRI---KA--------
Vitis_vini : --------------------------------------------MALKENQEETPISENPNGDEEGGEESQADGVGDFEDEDEDEA---------------------EEEDEEEEARSEKSRESRLAEDGSKLE--------
Ricinus_co : --------------------------------------------------------MEKPDPIAENPDNGEEEEEDDLDDDEDDLE---------------------PEPEPLNPRARESQARVEREKVENLIR--------
Populus_tr : ------------------------------------------------------MAEPESDISDPVVQNPNNEDEEDLYEQSDDVE---------------------DEDEDEDEDLESQKAKLRNR-FQNFFR--------
Ostreococc : ------------------------------------------------MATTTTTDARDDDASFTARGERRDATSDAADAAESSAS---------------------AESSASADAGATTSTPPVNEF--------------
Micromonas : --------------------------------------------------MADQAGQKKPYDYQERYRHVR-----------------------------------------------------------------------
Micromonas : -----------------------------------MTIENDVATSDATPSSSSSATEEPPPPPPPPPPPPPPQQQGGPPPGYDHQA--------------------------------------------------------
Pichia_sti : -----------------------------------------------MSLDHDDMLDRLASSPTGGHQDLSKSEK----ELQSLMQ---------------------EKQELMMKQSKDF------------ME--------
Hydra_magn : ---------------------------------------------------MGGSFVKPIYAADASQFVIQAEETDIDENDIPL----------------------------------------------------------
Ciona_inte : -----------------------------------------------------------------------------------------------------------MAGSSKEIV----------------LD--------
Trichoplax : -------------------------------MVTMVNLKLKLNLHIKYKNAFDFFLGKMPNQVLVSYPSPVYKNLQYFYLVPLSTRLKNTHVDTIVDTLQETTGKGSSMQGAENEKRGATRLTKLTDCIMYFLS--------
Salmo_sala : ---------------------------------------------------MGTVHARSMDPLPMHGRDMGVHPD----DMIEVQE---------------------AEQETKQEV----------------LE--------
Danio_reri : ---------------------------------------------------MGTVHARSLDPLPMQGPELGVQAD----DM-DLGE---------------------PEREEKQEV----------------LE--------
Xenopus_la : ---------------------------------------------------MGTVHARSLDPLPMNGPDFGS-PD----DA-DLVE---------------------VEPEKKQEI----------------LE--------
Monodelphi : ---------------------------------------------------MGTVHARSLEPLPVSGPDFGGLGE----EA-EFVE---------------------AEPEIKQEV----------------LE--------
Bos_taurus : ---------------------------------------------------MGTVHARSLEPLPASGPDFGALGE----EA-EFVE---------------------VEPEAKQEI----------------LE--------
Mus_muscul : ---------------------------------------------------MGTVHARSLEPLPSSGTDFGALGE----EA-EFVE---------------------VEPEAKQEI----------------LE--------
Homo_sapie : ---------------------------------------------------MGTVHARSLEPLPSSGPDFGGLGE----EA-EFVE---------------------VEPEAKQEI----------------LE--------
Macaca_mul : ---------------------------------------------------MGTVHARSLEPLPSSGPDFGGLGE----EA-EFVE---------------------VEPEAKQEI----------------LE--------
Pediculus_ : -------------------------------------------------------------------------------MKLEKK-----------------------------------------------LI--------
Apis_melli : ---------------------------------------------------MGTVYAKEKMNL------------------FEKRK---------------------EEPID--------------------LH--------
Tribolium_ : ---------------------------------------------------MGTVHAKSEENAPI-------LPT----IPQEHIK---------------------SEPAQHQDQREIF------------LD--------
Laccaria_b : -------------------------------------------MDGEFELSATPLRPPLQNSSAPRDKEPDDLQKLIKWQEERLAR---------------------RLRGEYESAVLH-------------LS--------
Coprinopsi : ---------------------------------------------MSEELSHPPLKPPLQNSSAPRDKEPKEIEKLLKWQQQRIER---------------------RLRGEYESAVMH-------------LQ--------
Schizosacc : ---------------------------------------------------MSVSPTDTMENTPA-----GIDTEQLKLSMEDLAQ--------------------------------------------------------
Neurospora : ---------------------------------------------------MASSPSAPGNPVEAATAKIDKAAAIVHQKAAAVV----------------------TEPKEPQPYQ---------------VP--------
Nectria_ha : -----------------------------------------------MAAIPSSSGGLSGDPLNKLHTDTGSLLHEAESKPEFQRE---------------------QEEAKQRRM--------------------------
Phaeosphae : -------------------------------------------MAAP---HEDDVFERLKN----KT-DQAEWQR----KQDELNT---------------------RLYAQQHKSQER-------------LG--------
Aspergillu : -----------------------------------------MASFSPPGASRGQPKRTVRGLLAIAAAEMSSPLSAEDEDIFERLQ-------------------QRADPKVLEEQQQAINERVHAIYQKAQMR--------
Ajellomyce : ------------------------------------------------MVSPLESDENIFERLKQKSTDPKVLEEQQQAVNERINA---------------------IYQKAQSRL--------------------------
Coccidioid : ------------------------------------------------MASPLQDDDGIFERLRKQNEDPKVLEERQQAVNERIHA---------------------IYQKAQTRL--------------------------
```

```
                    *      160         *      180         *      200         *      220         *      240         *      260         *      280
Neorickett : VHEFENSITALKTYYRDSVVEKTAISYRVVPIDENNVNVEVTVKEAKPTV--IRAI--EFEGNI----RYSDRVLKHVIRSREKSILRLFGTAHYYSREKLEFDKDLLADFYQGKGYFDYSLEGLEERENEDGVVL--VFKL
Rhizobium_ : EATVETDIQAIKDAYAAIGRSDVTVTTQVVPIAEGRVNLAFVINEGERTK--ITQI--NFVGNE----AYSDGRLQSVIATKESGIFSFLTRKDVYNPDKLRADEELLRQFYYNRGYADFNIISSEATLNEATNEYTVTITV
Rickettsia : QAKIELDVKKILEIYKRSGRFSTKVTPKIKSLENNRVKVIFDIAEGPKTV--IKSI--YFSGNE----HYSDSELKSIVLTKESRWFRFLESNDTYDPDRVEYDKELLREFYQSVGFADFRVISASVALNDTKEYFTITYSI
Rickettsia : QAKIELDVKKILEIYKRSGRFSTKVIPKIKNLENNRVKVIFDIAEGPKTG--IKSI--YFSGNE----HYSDSELKSIVLTKESRWFRFLENNDTYDPDRVEYDKELLREFYQSVGFADFRVISASVELNDTKEYFTLTYSI
Magnetospi : RSKVQADVKRILELYRRSGRFAATVEPKIIQLEQNRVDLVYEISEGQPTY--VRRI--AFVGNK----RYDQDKLREVLQTKEERWYRFLTTDDTYDPDRVTYDRELLRRYYLKQGFADFRVSSAIAELTPSREGFFITYTI
Gluconacet : TQTTAADRQKILGVYAEKARYAATVTPQIIRLSHNRVDVVFQINEATQTL--IKKI--SFVGNR----AFSEARLAQVVSSKETAWYRFFASSDEYNPERLRYDGELLRRFYLRNGFVDFQVKNATGELSPDRKSFYVTFTV
Chlamydomo : -------------------------------------A-LYEQIKDKPCH--VVQINQRKDGFNGGTFRTRASLIERELE-P--------------------------------------------------------IYKA
Phaeodacty : ---------------------------------------------QVELAWPARIV--DSNGNAP---RTNNELIQHRLLESGV------------------------------------------------------LYEV
Thalassios : ---------------------------------------DASDDNTSDAS--------KSKQNEPSLLRTDQDFINARLLEA--------------------------------------------------------GVPL
Saccharomy : ---------------------------------------------LKPIR--VAGV--VTTGTD----HIDPSVLQAYLDDT--------------------------------------------------------IMKS
Ectocarpus : ---------------------------------------------ELPLR--VRTV--KIVGNN----RTKPYVVEDQLQ-D--------------------------------------------------------AYEA
Phytophtho : ---------------------------------------------GPQIH--IGKV--FLKGNE----RTKPEVFENELQ-E--------------------------------------------------------AYSA
Phytophtho : ---------------------------------------------GPHMR--IGKV--FLKGND----RTKPEVFENELQ-E--------------------------------------------------------AYGA
Phytophtho : ---------------------------------------------GPRMR--IGKV--FLKGND----RTKPEVFENELQ-A--------------------------------------------------------AYSA
Physcomitr : ---------------------------------------------KEPVKVRVHDI--EIRGNV----KTKDSVIEAQLK-E--------------------------------------------------------LREV
Zea_mays_A : -------------------------------------A-VFQRLSEAPVGIRVHDI--IIKGNT----KTRDALIEAEVVGL--------------------------------------------------------IRSA
Sorghum_bi : ---------------------------------------------SNPVGIRVHDV--LIKGNT----KTRDELIEAEVA-D--------------------------------------------------------LLRA
Oryza_sati : -------------------------------------A-VFKRLSSDPVGIRVHDV--IIKGNA----KTKEELIEAEVAEL--------------------------------------------------------LRAA
Arabidopsi : -------------------------------------ESLFRRMRATPVAVRVHDV--IVKGNE----KTKDHVIEAEVD-V--------------------------------------------------------VRQA
Vitis_vini : -------------------------------------S-MFRRLASEKVKLRVHDV--LIKGNT----KTKDSLIEAELE-A--------------------------------------------------------IKNA
Ricinus_co : ----------------------------------------RMQTETVPLR--VHDV--IIKGNV----KTKDSILESETA-L--------------------------------------------------------LKDV
Populus_tr : ----------------------------------------RIQHESVPIR--VHDV--VIKGNT----KTKDTLIEAETA-S--------------------------------------------------------LKDA
Ostreococc : -------------------------------------RARYDRVRDDACA--VSSI--EIRGNE----RTRASVIERELA-R--------------------------------------------------------AYEA
Micromonas : ---------------------------------------------QKPLS--VYSV--EVKGDE----RTRPGLFARVLD-P--------------------------------------------------------VYRA
Micromonas : ---------------------------------------RYQALKDKPIA--VESV--EIQGIE----RTRRGILEAALE-P--------------------------------------------------------LYRA
Pichia_sti : -------------------------------------E-LFKQNSTQPIK--VKNV--QITNGH----SFRDNFLQAQFS-P--------------------------------------------------------LLNS
Hydra_magn : ---------------------------------------------KVPAV--IKNI--FIDGLC----KTKNVLIANQLS-D--------------------------------------------------------LFKA
Ciona_inte : ---------------------------------------------NKDIE--IVKV--HISGLV----KTKSDYVLDDIK-P--------------------------------------------------------VFGS
Trichoplax : -----------------------SISSALLYFGVIIIKSAEQQLKNKKIK--VHNI--NIDGLR----VTRTDLVQPKIH-N--------------------------------------------------------VFEA
Salmo_sala : ---------------------------------------------NKDVV--VQHV--NIEGLG----RTKEDLLGYEIS-E--------------------------------------------------------VFHA
Danio_reri : ---------------------------------------------NKDVV--VQHV--HIDGLG----RTKEDILTYEIA-D--------------------------------------------------------VFHA
Xenopus_la : ---------------------------------------------NKDVV--VQRV--HFEGLG----RTKDDLIAHEIG-Q--------------------------------------------------------VFKA
Monodelphi : ---------------------------------------------NKDVV--VQHV--HFEGLG----RTKDDIIMYEIG-D--------------------------------------------------------VFKA
Bos_taurus : ---------------------------------------------NKDVV--VQHV--HFDGLG----RTKDDIIMYEIR-D--------------------------------------------------------VFKA
Mus_muscul : ---------------------------------------------NKDVV--VQHV--HFDGLG----RTKDDIIICEIG-E--------------------------------------------------------VFKA
Homo_sapie : ---------------------------------------------NKDVV--VQHV--HFDGLG----RTKDDIIICEIG-D--------------------------------------------------------VFKA
Macaca_mul : ---------------------------------------------NKDVV--VQHV--HFDGLG----RTKDDIIICEIG-D--------------------------------------------------------VFKA
Pediculus_ : ---------------------------------------------YLESE--VDKI--NIEGLQ----RTKDDVVVETVQ-D--------------------------------------------------------LFKA
Apis_melli : ---------------------------------------------SVKAR--VDRI--HVDGLI----RTKDDIIKAQVI-E--------------------------------------------------------LFKA
Tribolium_ : ---------------------------------------------GVKAR--VDKI--HVDGLA----RTKDDIIEDCIR-D--------------------------------------------------------LFKA
Laccaria_b : -------------------------------------E-VVSCNRQTPVT--ISSV--RVEGAI----HTRKSFLGFIID-P--------------------------------------------------------ALKA
Coprinopsi : -------------------------------------E-TVNNNLKTPVN--IASV--RVEGAH----GTRTSFLGSIID-P--------------------------------------------------------ILAA
Schizosacc : ---------------------------------------ILNDNSTLPLG--IASI--RVVGAT----RTRPSFLEKAVSYK--------------------------------------------------------LAQK
Neurospora : -------------------------------------NPIIEDHLLTPAT--VNSI--EIHGAN----NTRRGLLDHVFK-P--------------------------------------------------------VVEE
Nectria_ha : -------------------------------------AQIVADQNLAPMT--VNEI--RVHGAV----NTRTDFLDPLFQ-P--------------------------------------------------------LVRD
Phaeosphae : -------------------------------------E-LVGEQLGTEEL--VLMS--NRSKTT----PRFPSFLERVVN-P--------------------------------------------------------ILSA
Aspergillu : ------------------------------------LGELIDQNSTLPCA--ISSV--QVLNAT----HTRRSFLQSIFD-P--------------------------------------------------------LLSV
Ajellomyce : -------------------------------------AELIDQNSTLPCT--ISSV--QVLNAN----NTRRGFLEKVLS-P--------------------------------------------------------LLSA
Coccidioid : -------------------------------------AELIDQNSTLPCT--ISSV--QVLNAS----HTRKDFLEKVLS-P--------------------------------------------------------LLSS
```

```
                      *        300         *        320         *        340         *        360         *        380         *        400         *        420
Neoricktt : KEGARYSFGRVNVVSEKSEIEISDLKEKVRIREGAVFNIGAVRENALTLLSVLNERGHMFVNVVPQYQPD-ADGRVDVT--YYVVSTKKYRIRKINISGNIRTRDTVIRREMLLSENDLYQPSKVADSRRRILNLGFFDEVY
Rhizobium_ : EEGPRYDFGAINVESTVEGVNAEELKGLVQSREGSVYKAKDIQDTMSEISKRVASAGYPFARVTPRGNRDLANHTIAVD--YLVDQGERAYVERIEIRGNTRTRDYVIRREFDVGEGDAFNQEMIARAKRRLEALGYFSSVN
Rickettsia : EEGEKYRFGNVTIDNKLTNINIKQLNKIVNIKQGKIFNMKTVDDIAEKIGEYFTANGYPAVNVYPDIKKN-DNHTADIK--FIIEKADKVYINKINIINNLKTEDHVIRRAFKIEEGDVMNRSYIEKGERNLRNLDYFE---
Rickettsia : EEGEKYRFGNVTIDNKLTNINMKYLNKIVNIKQGKIFNMKKVDDIAEKIGEYFTANGYSAVNVYPEIKKN-DNHTADIK--FIIEKGDKVYINKINIINNLKTEDHVIRRAFKIEEGDVINRSYIEKGERNLRNLDYFEKVS
Magnetospi : DEGERYKFGSSVINAELRDLKPEDLQPLLISETGGWYNADQVEDIVQRLTDAVGTKGYAFVDVKPQVQRNRETHTIDIT--YEIKEGPRVFVERIDISGNVRTLDQVIRREFRLVEGDAFNSAKLRRSRQRLKDLNFFEKAE
Gluconacet : DEGLRYRLGRVNIRSSLKHVPAASLRKYIELFANQWYDGSAVQHNATDMEEILQGQGHPFAMVRPEIARNPEKRTVDLL--FDVSEGPRVYVERIDINGNTITEDKVIRRNLPMAEGDPYTPSERKYAKAMLQDLGYFSSVS
Chlamydomo : -------------ETLAEVHEEMEAAGKRLRQLGVFT--GV--------SMLAHE-------EPLDDPT----ACTVE--LAVEE-SNWF-------------------------------------------------
Phaeodacty : -------------PVRVGATVESTNAFIQDLEKTGCFN--SV--------RVEVGQ-------GVSDEDASTRVQKQLK--ITLDE-KRWY-------------------------------------------------
Thalassios : GGGVPDLTPTSQSKMTITDHNASIARFIHDLEGTGCYD--AV--------QVYLGNNNEVSTSGNQDDGATSTDSFDVT--VRLKE-KKWY-------------------------------------------------
Saccharomy : -------------ITLGQLVKNADVLNKRLCQHHIAL--NAKQSFHFQGNTYISDE----KETHDVVPL-----MEVVSQLDILP-PKTF-------------------------------------------------
Ectocarpus : -------------TTVGDVYGGLVEGAQRLDGLGLFE--SV--------QVSMDAV------DDGSLDQ-----TDVT--VTVKE-KNWY-------------------------------------------------
Phytophtho : -------------KRIGRLVRNLEEATEELKALDIFE--SI--------DIKLDKA------SSGERDE-----TDIT--ITVKE-KGWR-------------------------------------------------
Phytophtho : -------------ERIGHLVRKLEEATEEFKALDIFE--SI--------NIELDKA------SSGKHDE-----TDVT--ITVKE-KGWR-------------------------------------------------
Phytophtho : -------------ERIGQLVHKLEEATEEFRALDIFE--SI--------NIELDKA------SSGQLDE-----TDLT--ITVKE-KGWR-------------------------------------------------
Physcomitr : -------------DTMQELLQECVRANSRLRALGIFD--KC--------VITLDA-------GPQELPG-T---ANVI--LEVEEVKRLF-------------------------------------------------
Zea_mays_A : -------------VTVQDLVRTANIAAVRLRLLDVFD--SV--------HVLLDA-------GPPELPG-T---TNVI--IQVVEAANPI-------------------------------------------------
Sorghum_bi : -------------APTVQDLLRAASVATARLHSLDVFD--TV--------KITLDA-------GPPELPG-T---TNVV--IEVVEAANPL-------------------------------------------------
Oryza_sati : -------------PTVQDLLRNASIASARLRQLDVFD--SV--------NITLDA-------GPPELPG-T---TNVV--VEVVEAANPI-------------------------------------------------
Arabidopsi : -------------TTLQELLKASKVANFNLQALDIFD--SV--------KITLDS-------GPPELPG-T---TNVV--IDVVESKSPI-------------------------------------------------
Vitis_vini : -------------TTMQELLKAAGIANHRFHSFGIFD--SV--------GITLDC-------GPPELPG-T---VNVI--VDVVETKNPL-------------------------------------------------
Ricinus_co : -------------SSMQELLEASKVVNFRLQALEVFD--SV--------RITLDS-------GPSELAG-T---ANVV--VEVVESKSPV-------------------------------------------------
Populus_tr : -------------STFKELFAASSDVNFRLQALEIFD--SV--------KITIDS-------GPPELPG-T---ANVI--VDVVETKSPL-------------------------------------------------
Ostreococc : -------------RTLDGIKDALFAANARLYEYGIFK--DV--------AIVIDADHEGGRLDGGDVPG-----AKVV--VSVEE-KDAF-------------------------------------------------
Micromonas : -------------STLDELRVRCVEANAVLNSYDIFD--RV--------DIEMDA-------GPREHPD-S---AKVT--VEVSE-KKKL-------------------------------------------------
Micromonas : -------------SSLDDLRDKCVDANGALNAYDVFE--SI--------DIVCDA-------GASSRPD-T---ASVI--VRVEE-KKRL-------------------------------------------------
Pichia_sti : -----KQP------VSLQEFLKQLSVVENLM--VGIHP--VV--------GTITTQS---IF-GRRTVPD-NGACISVVPIFNTLP-VKKF-------------------------------------------------
Hydra_magn : -------------KTFSDIMTEAHLGKLKLERLGIFS--TI--------DVLVDV-------SEDSHGD----AFDVH--YIVKE-KRRF-------------------------------------------------
Ciona_inte : -------------KTFLDAYQQSLFCREKLMSRGIFK--DV--------DVVIDTT----D-GYDNAPDNG---VQVV--FNVKE-RNGV-------------------------------------------------
Trichoplax : -------------ATFEELVKEMYLSKNYLEDLDIFH--PV--------NVFIDAA------SDKKADPIE---LDVT--FRVRE-LSRF-------------------------------------------------
Salmo_sala : -------------KNLIDVMKKSHIARQKLLRLGIFK--EV--------EVVIDTS----D-GADALPN-G---LDVT--FEVTE-MKRL-------------------------------------------------
Danio_reri : -------------KNLIDVMKKSHEARQKLLRLGIFR--HV--------EVVIDTA----E-GADALPN-G---LDVT--FEVRE-LRRM-------------------------------------------------
Xenopus_la : -------------KNLIEVMRKSHEAREKLLRLGVFR--NV--------EVLIDTS----E-GEDAVPN-G---LDVT--FEVTE-LRRL-------------------------------------------------
Monodelphi : -------------KNLIDVMRKSHETREKLLRLGIFR--QV--------DVLIDTC----Q-GDDALPN-G---LDVT--FEVTE-LRRL-------------------------------------------------
Bos_taurus : -------------KNLIEVMRKSHEAREKLLRLGIFR--QV--------DVLIDTC----Q-GDDALPN-G---LDVT--FEVTE-LRRL-------------------------------------------------
Mus_muscul : -------------KNLIEVMRRSHEAREKLLRLGIFR--QV--------DVLIDTC----H-GEDALPN-G---LDVT--FEVTE-LRRL-------------------------------------------------
Homo_sapie : -------------KNLIEVMRKSHEAREKLLRLGIFR--QV--------DVLIDTC----Q-GDDALPN-G---LDVT--FEVTE-LRRL-------------------------------------------------
Macaca_mul : -------------KNLIEVMRKSHEAREKLLRLGIFR--QV--------DVLIDTC----Q-GDDALPN-G---LDVT--FEVTE-LRRL-------------------------------------------------
Pediculus_ : -------------KDFQEVIINAHKVRGKLEKLGCFQ--NI--------GIFIDTS----T-GAEATLD-G---LEVT--FFVKE-LKRM-------------------------------------------------
Apis_melli : -------------QDFYDVVLRAYKVREKLQGLGCFG--NI--------GIYIDTS----Q-GPRATPE-G---VEVT--FMVRE-IRRL-------------------------------------------------
Tribolium_ : -------------ADFQDVLLRAHRARLKLDELGCFK--NI--------AVFIDTS----K-GQGASPD-G---LEVT--FNVTE-HKRV-------------------------------------------------
Laccaria_b : -----STVSPLDSQTNLESVLHTTRRIYHMLNKSDLFA--TI--------EAKVERA----R-DPLASSE-G---VDLV--FKTRE-RGRY-------------------------------------------------
Coprinopsi : -----SNKDSPEGQSDLESVLHTTRKIGAVLTKSDLFS--TV--------EAKIERA----K-NSSAANT-D---VDII--FKTKE-RGRW-------------------------------------------------
Schizosacc : ---------GNIKSIPLGESLETAQQIAERLMEFGIYE--DA--------KLLIDRAS-----GPIAGEN-D---IDIT--ILVKE-KSRL-------------------------------------------------
Neurospora : ---------TASPTTTLGEALARISTATQKLTRFGIFKEDGF--------GVFISDARQQQQ-EQFQSPT-DRTELDVS--IRVKE-QSRL-------------------------------------------------
Nectria_ha : ---------DQNATSTVGEVINKLRIASNKLDALQIFQPQP---------ELFLTSA---QQTDPSTSPT-D---VNVD--IRVKE-LSRF-------------------------------------------------
Phaeosphae : -----NRAEP----YTLEEALKEVGEATEKLNRFGIFK-SPI--------SVYLDRP--NQS-IASSSPS-D---VDVY--ITAYE-RGNY-------------------------------------------------
Aspergillu : ---------NQSRPYTLSEALREISSRADKLGKFDIFQ-QPI--------SVYIDQSQDAK--SQNGIPN-----IDVF--FSVKE-KSRV-------------------------------------------------
Ajellomyce : ---------NNDRPYTLSEALSEISICTDKLSRFDIFE-HPI--------SVFLDKP---SQTDPSTTPT-D---LEVY--LSVKE-KSRI-------------------------------------------------
Coccidioid : ---------NKDHPYTLAEALQEVGACTDKLHKFDIFQ-QPI--------SVYLDKP---SQTDPSTTPT-D---LDVY--LSVKE-RSRF-------------------------------------------------
```

```
                   *       440         *       460         *       480         *       500         *       520         *       540         *       560
Neorickett : IEER---KVDDQNLILEVRVKERPTGTLNLSGGYG--------SDVGFFGNFSFVENNLFGTSDRLVVELQRASLG---------SNYSVEFQRKRIFDTF-ITAGAS-------------------------------VFY
Rhizobium_ : ITTAPG-SAADRVIV-VVDVQDQSTGSFGIGAGYAAGSGGGFLVEASI------EEKNFLGRGQYIRLAAGKGEDS---------QTYNVSFTEPYFLGYR-LAAGFD-------------------------------LFK
Rickettsia : -------KVSISLAQTKAKDKYDVNVEVDEKSTSSIGFDLGYNTAGGLFGRFSFLERNLVGTGKLLNAGVQVSKNST--------SYYG-CITDPHFLDRD-LSLSVN-------------------------------AFR
Rickettsia : ISLA---KTKAKDKY-DVNVEIDEKSTSSIGFDLGYNTAGGLFGRFSF------LERNLVGTGKLLNAGVQVSKNS---------TSYYGCITDPHFLDRD-LSLSVN-------------------------------AFR
Magnetospi : VTNIPSDTAPDRTII-KVDVQEKSTGELMFGVGWA--SSAGPIIEASL------RERNLLGRGQDLRLGAGLGTKR---------SSLDLSFTEPYFMDRE-VAAGFD-------------------------------AFV
Gluconacet : IDQSPG-SAPDKINV-AANLVEKPTGEFSLGGGYS--TDVGILGNIGL------KQHNLLGSGVDAGISGTMAYYQ---------RQADISVSDPYFLNRN-MVAGVD-------------------------------IFA
Chlamydomo : -------KLRAATYV-QGGES-----T----------------FELGA------GLTNASGRAEALSANVEYGMENS--------HTASVAFKQPRVGGLP-ATLELR-------------------------------GSQ
Phaeodacty : -------RVNAGAGV------------------------------------------------------------------------KTDGWLRPETAVND--------------------------------------GF-
Thalassios : -------KLYIGGGV-NSEDLSSFGGTSGMGNTLGGPGGAALGILPKLQFETSASLLNLTGFADVSTALYSVDQTGA--------SSFKFTHDRPLCSWLP-KHSSIREWLMPPDPRLIDPEERNASNGQSADSVDENQVFV
Saccharomy : -------TAKTGTNF--GNDNDAEAYL----------------QFEKL------IDKKYLKLPTRVNLEILRGTKIH--------SSFLFNSYSSLPQSI----LNLK-------------------------------VFS
Ectocarpus : -------LLQSGATT-TGTAKGNLDAS----------------EFSNLRYSVAGALRNPLGHGEMLDVGYNSPIKGQEGHTVSAKLHLPHLFRTPVSGTLE-AIMDTV-------------------------------VFE
Phytophtho : -------SLHVGATT-DGSDE-----A----------------GESTL------TLSNALGEAEKITLSATYARSGS--------NTQRATFKKPRFLGLP-LYLSAI-------------------------------GTN
Phytophtho : -------SLHVGATT-DGNDE-----A----------------GESSL------TLSNALGEAEKITLSATYARSGS--------NTQRATFKKPRFLGMP-LYLSAV-------------------------------GTN
Phytophtho : -------SLHVGATT-DGNDE-----A----------------GESSL------TLSNALGEAEKITLSATYARSGS--------NTQRATFKKPRFFGLP-LYLSAV-------------------------------GTN
Physcomitr : -------SGDLGMFSKPETKA----WT----------------LEGTV------KYKNLAGLAETLDATGCYGLDTT--------SELSAGVTYPRFKGLP-ASLATR-------------------------------LTL
Zea_mays_A : -------DGSVGCFSKPEARS----WT----------------VEGSV------RWRNIFGYGDIWDASGAYGWDQS--------SEISIGVSLPRFRSIP-TPSTAR-------------------------------ASL
Sorghum_bi : -------TGTAGVYSKPEARS----WS----------------LEGSL------KLKNLFGYGDIWDASGSFGWDQT--------TEVGVGVYLPRFKSIP-TPLMAR-------------------------------ASL
Oryza_sati : -------TGSAGVYSKPEARS----WS----------------LEGSV------KLKNLFGYGDIWDASGAYSWDQT--------SEVGIGVSLPRFKSIS-TPLMAR-------------------------------ASL
Arabidopsi : -------TGQIGTFTKAEARS----SS----------------LEGSL------KYKNIFGYGDIWDGSLAYGCDHS--------AEVGLGMYLPRFRGRP-TPFTSR-------------------------------VYL
Vitis_vini : -------TGDLGIFTKPEART----WS----------------LEGSL------KLKNLFGYGDLWDGSLVYGWDQS--------SEISAGVSLPRFKGMV-TPMLAR-------------------------------VSL
Ricinus_co : -------SCEVGAYTKGEARS----ST----------------VEGTL------KYKNLFGYGDLWDSSVAYGGDHM--------TEVSAGVYVPRFKGRV-TPLTAR-------------------------------LFL
Populus_tr : -------LGEIGVFNKGEASS----ST----------------LEGTL------KYKNIFGYGDLWDGSLAYDCDHK--------AEVSAGVFLPRFKGLV-NPVTAR-------------------------------LFL
Ostreococc : -------HFKVGTYV-SKRGE----GE----------------AEATF------GLRNPLGYGEKFEVELIKGQNSS--------STYSAAWEQPKLYGSD-VNLDAR-------------------------------AFQ
Micromonas : -------NLKGGAYV-SQQGE----GS----------------MEVSV------GLNNALGYAEKLDVEFIKGHERS--------NSCTLAWNQPRVGNVD-VDVVTR-------------------------------AFQ
Micromonas : -------NLKGGAFV-SQARS----------------------MELSA------GVNNALGYAEKVDVEIARGHERS--------STYTLSWHQPRVRNED-VDVVTR-------------------------------AFQ
Pichia_sti : -------YAKTGTNI--GNGE----GD----------------GYIQF------QLKNIFGGAENISFDAVTGTKTS--------SSYLLNYNQPVFNNAD--YISEN-------------------------------SFS
Hydra_magn : -------SMNIGTSI--GANE----GG----------------MTMNA------NINNIRGYGETLKSGLSFGTRVS--------SAYEFAYIKPFSNDSD-KKFTIR-------------------------------LIK
Ciona_inte : -------VGSISTEM--SNTE---RPR----------------WVARL------LSPNLFGRGETLSTDIAHQLNSTTGNHLYQPTDFSATFMKPMRG----SSVKLR-------------------------------LLQ
Trichoplax : -------SAQFKTEV--GNQD----SR----------------LVIGG------KLRNLFGRAEELMGSIAYGAKKS-------------------------TTLQII----------------------------------
Salmo_sala : -------TGSYNTMV--GNNE----GS----------------MVLGV------KLPNVFGRAEKLTFQFSYGTKET--------S-YGLSFFKPQPGNFE-RNLTLN-------------------------------LYK
Danio_reri : -------TGSYNTMV--GNNE----GS----------------MVLGI------KLPNVLGRAEKLTFQFSYGTKET--------S-YGLSFFKPQPGHFE-RNFSLN-------------------------------LYK
Xenopus_la : -------TGSYNTMV--GNNE----GS----------------MVLGL------KFPNLFGRAEKMTFQFSYGTKET--------S-YGLSFFKPQVGNFE-RNFSVN-------------------------------LYK
Monodelphi : -------TGSYNTMV--GNNE----GS----------------MVLGL------KLPNIFGRAEKVTFQFSYGTKET--------S-YGLSFFKPQPGNFD-RNFSVN-------------------------------LYK
Bos_taurus : -------TGSYNTMV--GNNE----GS----------------MVLGL------KLPNLLGRAEKVTFQFSYGTKET--------S-YGLSFFKPQPGNFD-RNFSVN-------------------------------LYK
Mus_muscul : -------TGSYNTMV--GNNE----GS----------------MVLGL------KLPNLLGRAEKVTFQFSYGTKET--------S-YGLSFFKPQPGNFE-RNFSVN-------------------------------LYK
Homo_sapie : -------TGSYNTMV--GNNE----GS----------------MVLGL------KLPNLLGRAEKVTFQFSYGTKET--------S-YGLSFFKPRPGNFE-RNFSVN-------------------------------LYK
Macaca_mul : -------TGSYNTMV--GNNE----GS----------------MVLGL------KLPNLLGRAEKVTFQFSYGTKET--------S-YGLSFFKPRPGNFE-RNFSVN-------------------------------LYK
Pediculus_ : -------IGGANTMV--GNNE----GS----------------LVVSL------RAPNMFGRGEKLQAEYSYGSRRT--------NNLSLSFVKPLRTKYD-AMFTSS-------------------------------IFQ
Apis_melli : -------TGGISTMV--GNNE----GS----------------VIVHA------KAPNLFGRGERLQMEYSYGSKSS--------TNISISAVKPFIDNWRHTVLTGS-------------------------------VFN
Tribolium_ : -------TGGVTTQV--GNNE----GA----------------LLIGL------RAPNLFGRGERVQLEYSHGSKRT--------TNFNVAFIKPMRGKYR-PMFTTS-------------------------------VFQ
Laccaria_b : -------YVSSSTEL--GNNE----GS----------------ASASA------RVRNVFGGAETFTANLSLGTKTK--------RAFRASLVTPLTSDLD-TFGEVN-------------------------------VYG
Coprinopsi : -------YLSSATEL--GNNE----GS----------------ASASA------RVRNVFGGAETFTANLSLGTKTR--------RSFTASLSAPLTPDLN-TFGEFS-------------------------------VYG
Schizosacc : -------LAKTGTDI--GNVE----GN----------------VYANI------LARNAFGGAEFIEGNLSYGTRNR--------MLASLTFDTPINADPY-THFRVN-------------------------------AIN
Neurospora : -------VFKAGTDF--GNAE----GS----------------AYTNA------VLRNIFGGAETLSVNAAAGTRTR--------SAYNAVFSTPVNGNPD-IRLALE-------------------------------ALR
Nectria_ha : -------KLQTGTDV--GNGE----GS----------------AYGSL------LWRNMFGGAEMLTLNAKAGTRTR--------SAYSANLSMPVASNPD-LRISLE-------------------------------GVA
Phaeosphae : -------TIKTGTEA--GASE----AD----------------AYVHA------ELRNLLGGAETLNAHGSLGTRTR--------SAYSLAFDSPILSNPD-LKFQVN-------------------------------GFA
Aspergillu : -------LLKTGTDL--GNTE----GS----------------AYGNL------LWRNVFGGAETLNLNAAMGTRTR--------SAYQAAFETPILSDPD-FRFELG-------------------------------GIA
Ajellomyce : -------LLKTGTDL--GNAE----GS----------------AYANF------MWRNIFGGAESLNVNASLGTRTR--------SAYQAAFDTPILSNPD-FRCEIG-------------------------------GIG
Coccidioid : -------LLKTGTDL--GNAE----GS----------------AYANA------LWRNIFGGAETLNLNASLGTRTR--------SAYQATFETPILSDPD-FRFEIG-------------------------------ALA
```

```
              *       580         *       600         *       620         *       640         *       660         *       680         *       700         *
Neorickett : KNRNE------KANGLYKFSSVGGDGSV---------SYSLRDD-------------------------------------LRLHLGYSLSFDRI------FDVDGDAPE-----SVK-RSAGTKILSAVSYSLFLNK--LD
Rhizobium_ : NEHDF-------DDDNYSYNDQGFSLRVTAPI-----TENLSTT-------------------------------------LRYNYT---ELEYF------GDQDELSSP-----YDRVVEDSPWTRSSVSQSVTYNT--LD
Rickettsia : NYTGRGASVLNTTDQSYKLHSIGVKISL---------GYDMKED-------------------------------------LSHEIDYLIKRDIL------SAPSPSNSI-----FLN-EQMGKLITSAIGHTITYDQ--TD
Rickettsia : NYTGRGASVLNTTDQSYKLHSIGVKISLGYDM-----KEDLSHE-------------------------------------IDYLI----KRDIL------SAPSPSNSI-----FLN-EQMGKLITSAIGHTITYDQ--TD
Magnetospi : IDRKL------QKESSYDASSIGGDLRAGYRL-----SESLRQD-------------------------------------VIYTL----KQDT-------VKGISSTSP-----YVL-EQIGSKVSSVIGQSLMYDR--RD
Gluconacet : IQNNY------QTYQNYSEGRYGISFRMGYSY-----NNHLSQS-------------------------------------FSYSLT---DRDI-------DNAYSDASW-----YVL-KQTGWSLLSQLSTTLTYDT--RD
Chlamydomo : LFRNN------Q-------------------------NMDGSQG-------------------------------------LEYELG---WRRL-------LDPSRSASR-----AVMGQGVGPT-----DLMFQLPH--PA
Phaeodacty : -----------LPTAEIDHSLASPEY-----------TPDLYYG-------------------------------------VEWSLL---SRDLVPRRQSKMPYAMDASP-----EVV-SQAGPSLKHSILAEFRSNGELLD
Thalassios : ADDAQ------YSLGGGSHTSLGIHAMVHDLDFE---STRSSKEFVRSIGVRLANHCRGAAGGSNKPGVSPPEAMAGPYLFLDWNAS---LRDTMPRRNAEFPFALDCSK-----EVA-SQAGTVFKHSLTGGMYLNGCFTD
Saccharomy : QFYNW------NTNKGLDIGQRGARLSLRYEPLFLHKLLHNPHS------------------------------NESPTLFHEWFLETC-WRST------KICSQGTSAPYMYSGTML-SQAGDQLRTILGHTFVLDK--RD
Ectocarpus : DENTR-----QEHWTLTVERHASS-------------DVETRQA----------------------------------------------KRIT-------AIRTALSTA-----SCY-DQTLLLLLLLLLLLLLLLL--LL
Phytophtho : ELHNQ------EWLSSYSEKIRAGSISIS--------DYEGVHD-------------------------------------LSLNVG---WRDLLPRRDAKVPTAYRASP-----SIL-AEAMPSTKTSVKYVFTDDN--RN
Phytophtho : ELHNQ------EWLSSYNEKIRAGSISIS--------DYEGVHD-------------------------------------LSLNVG---WRDLLPRRDPKIPTAYRASP-----SIL-AEAMPSTKTSVKYIFTDDN--RN
Phytophtho : ELHNQ------EWLSSYNEKIRAGSISIS--------DYEGVHD-------------------------------------LSLNVG---WRDLLPRRDSKIPTAYRASP-----SIL-AEAMPSTKTSVKYVFTDDK--RN
Physcomitr : LTQDW------QRYSSYRERLTGFSVGL---------VGDNQHD-------------------------------------ISYNLT---WRDL-------KDPSRSASK-----SVR-RQLGHSLLSAVKYTYRRDT--RD
Zea_mays_A : LSHDW------LKFSSYKERLLGLSFGL---------LSDMHHD-------------------------------------LSYNLT---WRTL-------TDPSQAASK-----SIT-RQLGHNLLSALRYTYTIDQ--RD
Sorghum_bi : SSQDW------LKFSSYKERLLGLSFGL---------LSTRNHD-------------------------------------LSYNLT---WRTL-------TDPSHVSSK-----AIR-RQLGHNLLSALKYIYKIDK--RD
Oryza_sati : SSQDW------LKFSSYKERLLGLSFGL---------ISTMQHD-------------------------------------LSYNLT---WRTL-------TDPSQVSSK-----SIR-RQLGHNLLSALKYTYKIDQ--RN
Arabidopsi : STQDW------LKFSSYKERALGLSLGL---------IASKYHE-------------------------------------LAYNIA---WRNL-------IDPSQMASR-----SIR-RQLGHNLVSALKYTFKFDQ--RN
Vitis_vini : LSQDW------LKFSSYKERSLGLNLGL---------ISTKRHD-------------------------------------LTYNLL---WRTL-------TDPSQMSSR-----AVR-RQLGHGLLSSLKYTFRIDN--RN
Ricinus_co : LSQDW------LRFSSYKERSLGLSLGL---------VSSRNHD-------------------------------------LVYNLA---WRTL-------TDPSQMASR-----SIR-RQLGHGLLSSLKYTFKVDQ--RN
Populus_tr : QSQDC------LKFSSFKERSLGLSLGL---------FSTRNHD-------------------------------------VVYNLA---WRTL-------TDPSRTASS-----SVR-GQLGHGLLSSLKYTFKIDR--RN
Ostreococc : SVECF------KKLSSFDVTSRGIRVGV---------AGDGPGT-------------------------------------VDYEIA---WRDV-------QDPTRMASR-----EVR-RQLGHSLKSAVTHTYVSDR--LD
Micromonas : QVSCS------KRLSSFDETARGISVTA---------VGGGPAT-------------------------------------VDYSLV---WREI-------ADPTRLASK-----SIR-QQLGHSLKSSVSYTYQVDE--RD
Micromonas : SVTCE------KRLSSFDETDRGVSVQV---------LGGGPAT-------------------------------------LEYALV---LREI-------ADPTRLASR-----AVR-NQLGHSLKSSLSYSIGGDA--RD
Pichia_sti : TNTRK------WDWIQSNVKTFGFSNKIY---TQF--DGPVNHE-------------------------------------FTVENY---WKVL-------ENLN-SKSI-----DVY-TQSGSHFKNSLIYNVSYDT--RD
Hydra_magn : SMEDK-------SQSLYNENTKCAGFDFQ---IP---SALGVHV-------------------------------------FGLDFM---CRE--------NLINLEAPF-----SVR-ENAGHSLKSSIKHTLTSDG--RD
Ciona_inte : E-KQD------CYWSSYGMTIRGGEVGVE---GS---VKGNTHR-------------------------------------LSWVGH---WREL-------LCLNRNTAF-----DVR-EQMGHSLKSSIHSMTMDR--VD
Trichoplax : -------------------------------------TEFGAHS-------------------------------------LHFDSV---WREI-------QCLSDRSSW-----AVR-EESGHSLKCSIKHAFMRDT--RD
Salmo_sala : V-TGQ------FPWSSLKETDRGVSTELN---FP---LWRTNHT-------------------------------------LKWEGV---WREL-------GCLARSASF-----AVR-EESGHTLKSALSHTMSIDT--RN
Danio_reri : V-TGQ------FPWSSLRETDRGVSAEIS---FP---IWRTSHT-------------------------------------LKWEGV---WREL-------GCLARTASF-----AVR-EESGHSLKSSLAHAMVIDT--RN
Xenopus_la : V-TGQ------FPWSSLRETDRGVSAEIN---FP---IWKTSHT-------------------------------------LKWEGV---WREL-------GCLARTASF-----AIR-EESGHTLKSSLSHTMVIDS--RN
Monodelphi : V-TGQ------FPWSSLRETDRGVSAEYS---FP---IWKTSHT-------------------------------------VKWEGV---WREL-------GCLARTASF-----AVR-KESGHSLKSSLSHAMVIDS--RN
Bos_taurus : V-TGQ------FPWSSLRETDRGVSAEYS---FP---TWKTSHT-------------------------------------VKWEGV---WREL-------GCLSRVASF-----AVR-KESGHSLKSSLSHSMVIDS--RN
Mus_muscul : V-TGQ------FPWSSLRETDRGVSAEYS---FP---LWKTSHT-------------------------------------VKWEGV---WREL-------GCLSRTASF-----AVR-KESGHSLKSSLSHAMVIDS--RN
Homo_sapie : V-TGQ------FPWSSLRETDRGMSAEYS---FP---IWKTSHT-------------------------------------VKWEGV---WREL-------GCLSRTASF-----AVR-KESGHSLKSSLSHAMVIDS--RN
Macaca_mul : V-TGQ------FPWSSLRETDRGMSAEYS---FP---IWKTSHT-------------------------------------VKWEGV---WREL-------GCLSRTASF-----AVR-KESGHSLKSSLSHAMVIDS--RN
Pediculus_ : H-GSD------WPWSGYRVIERGVLFDLV---IK---ILQLRHN-------------------------------------IQYEGA---WREM-------GTSSRSSAF-----DVR-QESGHKIKSALRHILTFDK--RD
Apis_melli : T-SNR------FPWSGFSQCDKGFLLDIG---LNPDGAGTLKHN-------------------------------------LQYEAT---YREI--------ISSKQVSF-----RVR-EQCGPNLKSALRHICSIDK--RD
Tribolium_ : S-NAE------WPVSGYKQLDRGLLFDFG---FHS--TALIKHN-------------------------------------IQWEAA---IRDL-------AVLSRNTSF-----EVR-EQAGLSLKSALRHIISIDL--RD
Laccaria_b : LERDQ------SSYASCSEGLRGVKAVVRNGT-----PETGTHE-------------------------------------LGYEAV---VRHI-------SDLAPTASI-----SMR-EAAGESLKSSISHTYLFDT--RD
Coprinopsi : LERDQ------TSYASCFEGLRGLKAVVKGGT-----LEKGLHE-------------------------------------MGYEAV---HRHV-------RDLLPTASI-----SMR-QAAGESIKSSIFHSYTLDT--RN
Schizosacc : VLRDN------KLIASHDILTRDVNASITHKD-----AWNGFHE-------------------------------------LKQSLT---WRQV-------TNLVDTASP-----SIR-LAAGDSLKQAFSYSYTLDK--RD
Neurospora : -SSTH------KPWASHDEHLTGGNLRLAWST-----DNGDDHA-------------------------------------LTYSGV---WRQL-------TGLSASASP-----TVR-ADAGDSLKSSITHTFTRDR--RD
Nectria_ha : SAAEK-------PWASHEEVLKGGTVRFSWLN-----SERDTQS-------------------------------------VEYSHV---WRQI-------TGLGEGASP-----TVR-ADAGDSIKASIKHSFYRER--RD
Phaeosphae : S-STL------KSWASHEEVLRGGTSKLLWRS-----KTGHQHE-------------------------------------LGYSGV---WRQV-------TSLAENASP-----TVR-ADAGDSFKSSITHAWTNDK--RD
Aspergillu : SSTQK-------SWANHEEVLKGGWSKLRWLS-----QSGHRHE-------------------------------------IGYNGF---WRQV-------TGLAENASP-----TVR-ADAGDSVKSSIFHSWVADR--RD
Ajellomyce : SSTEK-------SWASHEEVVKGAWTKLRWLS-----PSGHRHE-------------------------------------LGYNGF---WRQV-------TGLSTDASP-----TVR-GDAGDSVKSSISHTWVNDR--RD
Coccidioid : SDTQK-------SWASHEEVVKGAWSRLRWMS-----AAGHRHE-------------------------------------LGLNGF---WRQV-------TGLAGSASP-----SVR-ENAGDSVKTSISHTWTKDQ--RD
```

```
                      720         *       740         *       760         *       780         *       800         *       820         *       840         *
Neorickett : NYFVPRY--GYGVRFGNKFAGIG-----GDVKFLRSDFKAGGFVSIFD-------------------------QSAVLSLVVRAGNIFGYSGQ---------GVDVA-NRFFL-------------NEMRGFDSLGIGPRDV
Rhizobium_ : DAQLPHE--GILASVTQEFAGLG-----GTSDFYKLTGKAKWYYTVND------------------------EADIIASLAGSAGHLFKTSGS----------MEVF-DQFQLGS-----------NDIRGFERNGIGPRVN
Rickettsia : NKIVPKN--GYLVSGTQEFAGVG-----GDNKYIKHEIECKFYKSFIN-------------------------NKVTLKLSAAGGDMAGLGGK---------MVRIS-DRFNLGD-----------YSLRGFASGGVGPREK
Rickettsia : NKIVPKN--GYLVSGTQEFAGIG-----GDNKYIKHEIECKYYKSFIH-------------------------NKVTLKLSAAGGDMAGLGGK---------IVRIS-DRFNLGD-----------YSLRGFASGGVGPREK
Magnetospi : SRLDPTE--GYFIKLGTDGAGLG-----GDIRYLRGTLTGGQYFTL-T-------------------------DKVILGVSSSVGYIYGLDQR----------VRIT-DRFFVGG-----------DNLRGFANGGVSPRDK
Gluconacet : NRMNPHS--GYVVRLAGDFAGIG-----GNERYLRGKLDAAYYIPLDDLMGN---------------------HDWTVALTAGAGDIVNWGGG---------RSDII-DNFYLGG-----------STLRGFMDGGAGPRTM
Chlamydomo : LKLLRLH--GHAFVNGGNVIQLA-----GTGRSPSELLSE---------------------------------FGSSWRWSCGTGLVLPTPFGRFEANYCVLLSSQEHDRNSL------------VLSLLGLALI-------
Phaeodacty : HPYSPTG--GVEMHGSAEVAVPP-----GSVGFVRCNGGFGIHMPL-L-------------------------QSLSVHSIFNAGYLKALSFG-----GLCRPPTLS-DRYYVGG----------PLRFRGFVPAGIGPRTK
Thalassios : DRYDPTM--GYDAHVLGEVAG--P---PGDVGFWKLKGGGSWHLPL-ELIGMMAFGVNEEEYTSKEEDESPVVIGATLHSSFNAGLIRPLSFSGLCNSGSIGGVPSS-DKFYVGG----------PGQLRGFLPSGIGPRSA
Saccharomy : HIMCPTK--GSMLKWSNEL---------SPGKHLKTQLELNSVKSW-MND-----------------------DFITFSTTIKTGYLKNLSSQ------QSLPVHIC-DKFQSGG----------PSDIRGFQTFGLGPRDL
Ectocarpus : LLLLPSPPVGDTHTFGMRI-GLM-----GDTSFAKGMLEMQRHIPL-FGVELGPET----------------YSPVTLSLCASGGAIRPFGPS--------KRTFFS-DRFNLGG----------PMTLRGFPFYGAGPRSP
Phytophtho : NVVYPTA--GGLFKYTTEIAGLV-----GDVKFVKAEVEGQKHVAV-GPNVFG-------------------FPILNFSLSCHLGTVKSYGNE------QHRPARIS-DRFFLGG----------PMNVRGFNHKGIGPRAS
Phytophtho : NAVYPTA--GGLFKYTTEIAGLV-----GDVKFVKAEVEGQKHVAL-GPVVFG-------------------FPILNFSLSYHVGTVKSYGSE------QHRPARIS-DRFFLGG----------PMNVRGFNHKGIGPRAS
Phytophtho : NVVYPTA--GGLFKYTTEIAGLV-----GDVKFVKAEVEGQKHVAL-GPVLNG-------------------FPILNFSLSYHLGTVKSYGSE------QNRPARIS-DRFFLGG----------PMNVRGFNHKGIGPRAS
Physcomitr : SVFRPRR--GIAFISTTQIAGLGP--DAKLLRFARQEVELRLAIPLGL-------------------------ANAALNLGVAGGIILPWGPG-----YKTKATPIG-DRFYVGGHSSLLGELKGPSALLGFRTRGVGPNEL
Zea_mays_A : SHLRPTK--GYAFVSTSQVSGLWG---SKGLRFFRQEFDIRGAVPLGF-------------------------YNAALNAGISAGVILPLGRG-----FMESPSSVP-DRFFLGGHSSPVISLCSLTSLLGFKTRGVGPTEL
Sorghum_bi : SHLRPTK--GYAFQSTSQVGGLW---NSKGLRYFRQEFDVRGAVPLGF-------------------------YNAALNVGVGAGVILPLGGG-----FMNSTSPVP-ERFYLGGNSSPVCSLGGLSSLLGFRLRGVGPTEA
Oryza_sati : SHLRPTK--GYAFLSTSQVGGLW---DSKGLRFFRQEFDVRGAVPLGF-------------------------YNSALNVGLGVGAILPLGRG-----FMNLSSSVP-DRFYLGGHSSPVCSLSGLSSLLGFRTRGIGPTEP
Arabidopsi : SSLRPTR--GYSFISTSQIGGLAP--DSRTLRFLRQEIDLRYAVPLGF-------------------------YRAALNFGIAGGITFPWGSG-----YKSRASCVP-ERFFLGGNISPVCSLGGPSALWGFKTRGLGPNEP
Vitis_vini : SVLRPTQ--GYAFISTSQIGGLVP--DYRSLRFLRQEIDLRCAIPLGF-------------------------YRTALNLGISGGVIFPWGNG-----ALSMPSSLP-ERFFLGGNSSPVCTLGGPTTLLGFKSRGLGPTER
Ricinus_co : SPLRPTH--GYAFVSTTQVGGLAP--DSRSLRFLRQELDLRCAIPLGF-------------------------LRSALNLGISVGVVFPWGTG-----FSNMPSPLP-ERFFVGGNLSPVCTLGGPMAFYGFRTRGLGPTEP
Populus_tr : SPLRPTR--GFAFVSTTQIGGLAP--DSRSLRFLRQELDLRCAIPLGF-------------------------YHSALNLGISAGVVFPWGSG-----FSSMPSPLP-ERFFLGGNLSPVCTLGGPTSLWGFRTKGLGPTEP
Ostreococc : RQVRPTS--GYLWKIRSELAGIGFMDNALAAKFVKSEVSAHVVETL-DAS-----------------------RGITASASGRFGVLFPWGKS---GNDGDSRTCIA-DRFFLGG----------VGCLRQFENNGVGPSDA
Micromonas : RPVRPGA--GYLARVRSELAGVGW--DPQMTRFLKHEAEVQAAHTP-A-------------------------EGVTFFASAKLGAMMPLGQN---AKDPEKGTCIA-DRFFLGG----------VGSLRGFEPHGAGPSDE
Micromonas : RAVRPTT--GALWRLRSELAGIGF--DRNATRFFKQEAEVKAFKRL-A-------------------------EGVTWHLGGKLGALVPLGAA---AREDQRGTCIA-DRFFLGG----------VGSLRGFEPRGVGPSDV
Pichia_sti : NKHLPTQ--GKFLRFGVEYNGLF---KFTTSPFIKSVFESQFVYAF-PKN-----------------------WYTSVILTGKSGFLFPLNKT----------TSVL-DRFYIGG----------PNDVRSFTLNGLGPKDY
Hydra_magn : DWIFPSS--GHLVKHSVEYSGIG-----GNVKSIKSDLELQINKEI-I-------------------------PNVVVSCSFQGGVVRSLTSD---------LLLIN-DRLFLGG----------PVSIRGFAMRGIGRHDG
Ciona_inte : DVILPMR--GGLVKMEEEVAGVG-----GDVSFIKETMEAKVAATI-F-------------------------DKITFVASMHCGVMVGW-----------RGVSVC-DKFFIGG----------PLTLRGFELNSVGARSG
Trichoplax : HSVSPTT--GHFIRFEQEHAGLG---SLGDTKFHRFFLECQYNKTL-P-------------------------YNIVFSSSLNLGLLASDGQS-----------KIS-DRFFLGG----------PLSIRGFQMRGIGPHSE
Salmo_sala : SAIFPKK--GALLRINQELAGYTG----GDASFLKEDFELQVNRRL-V-------------------------WDSVLSASLWGGCLLPIGGR---------PSSIA-DRFYLGG----------PTSVRGFGMYSIGPQSE
Danio_reri : STILPRK--GALLKINQELAGYTG----GDVSFLKEDFEIQLNKTL-F-------------------------WDSVLSTSLWGGMLLPLGEK---------ASSIA-DRFYLGG----------PTSVRGFSMYSIGPQSE
Xenopus_la : ASILPKR--GALLKINQELAGYTG----GDVSFLKEDFELQLNKQL-A-------------------------WDSVLSTSLWGGMLVPVGDR---------PSSIA-DRFYLGG----------PTSVRGFSMYSIGPQSE
Monodelphi : SSILPRK--GTLLKVNQELAGYTG----GDVSFLKEDFELQLNKQL-F-------------------------FDTVLSGSLWGGMLVPIGEK---------PSCIA-DRFYLGG----------PTSVRGFSMYSIGPQSE
Bos_taurus : SSILPKR--GALLKVNQELAGYTG----GDVSFLKEDFELQLNKQL-I-------------------------LDTVFSASLWGGMLVPMGDK---------PSSIA-DRFYLGG----------PTSVRGFSMHSVGPQSE
Mus_muscul : SSILPRR--GALFKVNQELAGYTG----GDVSFIKEDFELQLNKPL-A-------------------------LDSVFSTSLWGGMLVPIGDK---------PSSIA-DRFYLGG----------PTSVRGFSMHSIGPQSE
Homo_sapie : SSILPRR--GALLKVNQELAGYTG----GDVSFIKEDFELQLNKQL-I-------------------------FDSVFSASFWGGMLVPIGDK---------PSSIA-DRFYLGG----------PTSIRGFSMHSIGPQSE
Macaca_mul : SSILPRR--GALLKVNQELAGYTG----GDVSFIKEDFELQLNKQL-I-------------------------FDSVFSASLWGGMLVPIGDK---------PSSIA-DRFYLGG----------PTSVRGFSMHSIGPQSE
Pediculus_ : AQIFPSS--GTLFRFTTELAGLG-----GDIKFLKNDFFAQYNFPL-I-------------------------ADMVLQATLQAGYLQPLGDG--------EAVGLP-DKFYVGG----------PMSVRGFETRGVGPRSD
Apis_melli : SLIFPTM--GSLVQFSTEVAGLG-----GDIGFVKNELTMQTNWSP-Y-------------------------ECLTFQLGFQSGLLRGISND--------MKINIA-DHFFLGG----------PLNLRGFDMRGCGPRHD
Tribolium_ : DLIFPTS--GSLLQLTSEVAGLG-----GDVGFLKNEFFIQGNYSL-V-------------------------EDFVLQGTLSGGFMRGLSND--------MKIGMS-DMYYLGG----------PLTLRGFQMRGVGPHSE
Laccaria_b : DRMMAKK--GVYAKLYHEFAGLG-----GDASFYKAEAEGQVSRPV-S-------------------------NGVSISLAARAGLLSSLKGS----------TLFS-DRFQLGG----------PTSIRSFKANSMGPRDG
Coprinopsi : DRIAATR--GGYVKFYHEFSGLG-----GDASFYKTEAEGQVSRPL-I-------------------------PGLSFSLAGRTGLL--WGLN--------KPLLFS-DRFQLGG----------PTSVRSFKANGLGPHDG
Schizosacc : NPALPTK--GGLLKYMLELAGMGP--IIGDAKFVKNEIWSQLSIPL-NVS-----------------------HSSCATVGLRAGAIHDLDNE---------TIGIC-DRFYLGG----------PTSLRGFTEGRIGPKDG
Neurospora : NPMLPQA--GYLVRTAAELAGWGP--LKGDVSFAKSEVELSAAQALPL-------------------------PGVSVGAGFRAGLLYPLPMGYSLSSTSVAPSRIN-DRFQLGG----------PTDVRGFSMGGLGPHDG
Nectria_ha : NPQLPQD--GYMVRTGLEVAGFGP--LVGDVAFSKGEVEVGGAIPV-PLPGIKD------------------RTGISIGGGLRLGLLCPLPLG-YDFGGKLGLSRIN-DRFQLGG----------PTDVRGFKLGGLGPRD-
Phaeosphae : YPLLPSS--GYLMKTVSELAGFGG--LSGDVAFFKSEAESQVALPFGN-------------------------TGITLTAGLRGGLIYPLALP---GQNQPQGSRIN-DRFILGG----------PNNVRGFRLAGLGPHDG
Aspergillu : NPLLPSR--GYYAKAFNELAGWGP--LKGDVSFWKSEVEAQGAIPI-PIPGIKG------------------DSGISFTTGFRAGLLYPLGLD---SDSRPQLSRIN-DRFVLGG----------PTDVRGFRLCGLGPHDG
Ajellomyce : NPLLPSR--GFYTRTFNEVAGLGP--LKGDVSFWKSELEAQGAVPI-PIPGVKG------------------DSGISLTTGFRAGILYPLGLD---ATQKPQLSRIN-DRFQLGG----------PTDVRGFRLAGIGPREG
Coccidioid : NPILPTK--GFYAKTLNELAGWGP--LKGDVSFWKSEIETQAAVPV-PIPGLKG------------------DSGISFTAGFRAGLIYPLGMD---SNSKPQLSRIN-DRFQLGG----------PTDVRGFRISGLGPRE-
```

```
                  860         *       880         *       900         *       920         *       940         *       960         *       980         *
Neorickett : DT----------------------------GDALGGNSFILGTAEVQVPMR---L--PVELD-------LKAAFFYEVGTLAGVDV-----------------------TAEKVYD----SHA-LRSSIGAGLVWNSP-FGV
Rhizobium_ : N-----------------------------GDALGGTTYFTASAEASFPLP---G---IPRDSG-----FRGALFVDAGTLYGNDV-----------------------ALTGAGEFAEGTDASLRVSVGVSLIWASP-FGP
Rickettsia : NT----------------------------NEGLGGERYYTFSTELNFPTP---V----PEEFN-----FTGAVFIDLGSV--WGV--GLN------------------KKQYKTPNGFYNDQSLRASVGFGFIWVTR-FAP
Rickettsia : NT----------------------------NEGLGGEKYYTFSTELNFPTP---T----PEEFN-----LTGAVFIDLGSV--WGV--GLN------------------KKQYKTPNGFYNDKSLRASVGFGFIWVTR-FAP
Magnetospi : NT----------------------------GDALGGIWQAVGSAQVKFPLG---L----PEEFG-----VSGQAFTDIGAVGETDT-----------------------ADTAAVQQ---SSS-VRVSPGVGISWKSP-MGP
Gluconacet : GIPAYTWQGVTYPMHSQ-------------EDFLGGRFMYTGSATVNFPMP---L----AADMG-----IKGRYFVDMGGLDGLRI--PRR------------YTAYPQDMPYYTPVYSDTIK-PRVSTGVGFSWKSP-FGL
Chlamydomo : ------------------------------SSYSLDIDTICNFLDLDLDPA---I--RIARSNH-----VDSSSEEAVEDLVLWRK-----------------------PKEAGAIF---GGA--TAAYLADIMHTRSAAPG
Phaeodacty : HG------------GSSTPG----------GDAVGGDFFYTATAMASI----------------------------------------------------------------------------------------TP-TSG
Thalassios : KG------------GSSVPG----------GDSLGGDVYYTTTLAGSIPFP---SYFATLRQNG-----ARVFGFTNVGTCVSTGG--SVL------------------GMPAFSQI---LQS-SRVAVGGGVSVGSP-MGR
Saccharomy : ------------------------------YDAVGGDAFVSYGLSVFSRLP---W--KKVEKSN-----FRLHWFFNGGKLVNHDN---------------------TSLGNCIGQL---SKE-HSTSTGIGLVLRHP-MAR
Ectocarpus : KE------------EGGCEG----------GDALGGDIRYTASASLGFPFP---V--PAMATAG-----WRGYLFTNLGNLTTWDT--------------------------PLKQY---GRD-TRVSVGVAAAWNFLGVGR
Phytophtho : PL------------DGGVAQ----------GDALGGDVSYNGTASVGFPVP---L--PLLAALG-----LRGQLFANAGNLTTWDR--LLD------------------EKKWMKNL---ADD-TRVSIGLGLVWGTR-IGR
Phytophtho : PL------------DGGVAQ----------GDALGGDVSYNGTAALGFPVP---L--PLFAALG-----LRGQLFANAGNLTTWDR--LLD------------------EKKWMKNL---ADD-TRVSVGLGLVWGTR-IGR
Phytophtho : PL------------DGGVAQ----------GDALGGDVSYNGTASLGFPVP---L--PLFVALG-----LRGQFFANAGNLTTWDR--LLD------------------EKKWLKNL---TDD-TRVSVGMGLVWGTR-IGR
Physcomitr : RRTTASQTDKVDSEVETSLK----------RDTLGGDLAVSGFADISFDLP---M--QFLKSYG-----IHAHTFACAGNLVPLTG--NNT------------------TQWSLRNF---LSG-FRVSSGAGIVIPTK-LFR
Zea_mays_A : RRIVPSESVTNE--STASPG----------WDYLGGDFAVSAFADLSFDLP---L--KLFQDAG-----IHGHAFLSAGNLAKLSE--GEF------------------RNFSFSKF---GRT-FRSSAGVGIILPTK-LLR
Sorghum_bi : RRHVLDKPES----GSGDPG----------RDYLGGDLAVSAFADLSFDLP---L--KIFRDAG-----IHGHAFLTAGNLAKLSE--GEY------------------KNFTASEF---QRT-FRSSAGVGIILPTK-LFR
Oryza_sati : RRLVPSESEDG---SAASPG----------RDYLGGDLAVSAFADLSFDLP---L--KIFRDAG-----IHGHAFLTAGNLAKLSE--GEY------------------KKFSLSEF---GRT-FRSSAGVGIILPTK-LFR
Arabidopsi : RREVQDDE------SGDTYE----------RDFVGGDVAVTAFADLSFDFP---L--KWFRDRG-----IHGHVFACAGNMAELSE--NKY------------------RNFTAPKL---LET-FRSSVGAGIVVPTS-LFR
Vitis_vini : RRLIRDKSNGE---NSETSG----------RDVIGGDLAVTAFADLSFDLP---L--KLFRDAG-----IHGHVFACAGNLTKLTE--NEF------------------RKFSFQKF---LDS-FRSSAGFGIIVPTK-LFR
Ricinus_co : RRQLQSNSTD----DSADPG----------RDYLGGDLAVTAFADLSFDFP---S--KWCQAKG-----IHGHVFASAGNVDKLTE--NAY------------------RNFSLQKF---VES-SRSTVGVGIVVPTN-LFR
Populus_tr : RRQLQNNPADENADSG--------------RDYLGGDLAVAAFADFSFDFP---S--KWLSAKG-----IHGHLFASAGNVDKLTE--NSY------------------RNFSLQKF---TQS-FRSSVGVGVVIPTN-LFR
Ostreococc : RRGTKKTAAAEESPGAKTPSSAEDGEPSLTRDALGGDFVWSATAALQMDIPGEKF--EAMREAG-----IHFHAFATAGTLLPLAALSGA-------------------PKDAIKVV---RDA-TRASLGVGIVWPLP-IGQ
Micromonas : RRPPPTKAAAS---GENLIT----------RDALGGDVLAQLFASYQLEPE---W--SKLRDIG-----VYGHFFVNAGTLMPWIT--GER------------------KRVSGREL---ADS-FRASVGVGLVFPLP-VGN
Micromonas : RRPPKDPAAAAAAGMSS-------------RDALGGDLLAQAFTAVQATPT---W--SKLSDIG-----VYAHAFANVGTLARVNTPGVAA-----AAAAAAGEGGDGDRPSSAREF---LEG-FRGSLGVGLVFPLP-VGN
Pichia_sti : ------------------------------NSSIGGDMFINGGLSFISKIP---R---VSSESN-----FKLHNFVNFGKLVPMDK--SAG------------------FVRNFKSV---VGD-FSLSYGFGILYNHP-MAR
Hydra_magn : ------------------------------QASLGGEKYWASGLHLYTPLP---F-RPGQGGFG---DLFRLHLFFNAGSLSDFNT--------------------------CFRDF---ISK-PRYSYGLGVMIMLGAVAR
Ciona_inte : ------------------------------GDYLGSGAFWLSGLHIYTPLP---F-YWSRFGGGSWLDNFRTHAFVNGGNSFDVNI--TES------------------AGANFNAA---TNN-VRLSCGIGLVYKFMNSAR
Trichoplax : ------------------------------GDSIGGNALWASGLHLISPLP---F-KPLQGGFG---ERLRTHLFVNAGNLGHIRS--SA-------------------IRDSTEEL---IRG-YRWSYGIGLLVKLG-VAQ
Salmo_sala : ------------------------------GDYLGGEAYWAGGLHLYTPLP---F-RPGRGGFG---DLFRTHFFLNAGNLCNLNY--GEG------------------PRAHLQKL---AEC-IRWSYGAGIVLRLANIAR
Danio_reri : ------------------------------GDYLGGEAYWAGGVHLYTPLP---F-RPGRGGFG---DLFRTHFFLNAGNLCNLNY--GEG------------------PRAHLSKL---AEC-IRWSYGAGIVLRLGNIAR
Xenopus_la : ------------------------------GDYLGGEAYWAGGVHLYTPLP---F-RPGRGGFG---DLFRTHFFLNAGNLCNLNY--GEG------------------PRAHLQRL---AEC-IRWSYGAGLVLRLGNIAR
Monodelphi : ------------------------------GDYLGGEAYWAGGLHLYTPLP---F-RPGQGGFG---DLFRTHFFLNAGNLCNLNY--GEG------------------PRAHLRKL---AEC-IRWSYGAGIVLRLGNIAR
Bos_taurus : ------------------------------GDYLGGEAYWAGGLHLYTPLP---F-RPGQGGFG---ELFRTHFFLNAGNLCNLNY--GEG------------------PKAHIRKL---AEC-IRWSYGAGIVLRLGNIAR
Mus_muscul : ------------------------------GDYLGGEAYWAGGLHLYTPLP---F-RPGQGGFG---ELFRTHFFLNAGNLCNLNY--GEG------------------PKAHIRKL---AEC-IRWSYGAGVVLRLGNIAR
Homo_sapie : ------------------------------GDYLGGEAYWAGGLHLYTPLP---F-RPGQGGFG---ELFRTHFFLNAGNLCNLNY--GEG------------------PKAHIRKL---AEC-IRWSYGAGIVLRLGNIAR
Macaca_mul : ------------------------------GDYLGGEAYWAGGLHLYTPLP---F-RPGQGGFG---ELFRTHFFLNAGNLCNLNY--GEG------------------PKAHIRKL---AEC-IRWSYGAGIVLRLGNIAR
Pediculus_ : ------------------------------GNATGSNVALAGGLHLYTPLP---F-RPGKGGFG---DLFRVHFFLNMGQCKEYEF--GNG------------------VTEDVKNI---LDG-YRLSYGCGLALRLGQLAR
Apis_melli : ------------------------------GNSIGGDAYWALALHLYTPLP---F-RPGRNGFG---DLFKLHGFINGGNVSNFTFKFAND------------------YKENMKIF---KEN-VRCAVGGGIAMKLGNIAR
Tribolium_ : ------------------------------GDALGSMAYWASGLHLFSPLP---F-RPGRGGFG---DLFKTHLFITAGNVGDFSL----S------------------DKNLIDSL---TAS-VRISYGVGIALRLGNMAR
Laccaria_b : ------------------------------PDYIGGDIYYSAGASLISNIP-------KKPHWP-----VKAHLWVNAGRLDALDK-----------------------SRPLLESIQNTLSR-PSISAGVGLIYRFD-PIR
Coprinopsi : ------------------------------QDSIGGDIYYSVGASLIGDIP---N----KAHWP-----LKGHLWVNAGRLEQVDR--ARP------------------LTDNVKDM---LAR-PSISAGLGLIYRFD-PVR
Schizosacc : ------------------------------KDCLGGTAYLAASASLTSPVP---G---VNPSKP-----FRMQFFINAGGLRMLDQ---RQ------------------PLRTFRDI---IQK-PCVTSGIGLIYATG-VAR
Neurospora : ------------------------------ADSVGGDVFAAGSVNMLLPLP------RAGPTSP-----LRFQLFANGGRLVALQGKKTAE-----GSVSLDSGAVASGMKSAVAEL---ANGLPSIAAGFGLVYAHP-VAR
Nectria_ha : -------------------G----------ADSVGGDVFAAGSVNMLLPVP---Y---KGPDSG-----LRFQLFANGGRLVALKN--PNKSSSGAATQTLRPSAVRDGMLNAVGEL---FNGVPSLAAGVGLVYAHP-VAR
Phaeosphae : ------------------------------SDAVGGDVYAAGGASLLLPVP------RVGKDTP-----LRLQAFINGGRLLSLKGQKGQQ----------------TSLSQTFASL---TDELPSVAAGIGLVYAHP-IAR
Aspergillu : ------------------------------ADAVGGDVYAAGSANLFLPLP------RVGADKP-----LRLQAFVNGGRLLPLRTLQKEA--------PTNSTEVKDAMTATISEL---GNGLPSVAAGIGLVYAHP-VAR
Ajellomyce : ------------------------------PDALGGDVFAAGSANLLFPLP------RVGAEKP-----LRLQAFVNAGRLLPLKTQDRKL--------PSTQGEVRDSMVATVSEL---GNGLPSMAAGLGLVYAHP-AAR
Coccidioid : -------------------G----------PDALGGDVYAAGSANLLCPLP---R---VGADKP-----FRLQAFVNAGRLLALKT--PQGAS------PTSPQEVRESVASTLSEL---ANGLPSTAAGVGLVFAHP-VAR
```

```
                     1000         *      1020
Neorickett : LRVDVAKALIEGKGDKVK--TVKF---GIVSPF--
Rhizobium_ : LRVDYAVPVAKEDFDEIQ-N-FKF---GINSSF--
Rickettsia : IRMDWGFPIKKKQYDDTQ--NFHL---RFSTHL--
Rickettsia : IRMDWGFPIKKKQYDDTQ--NFHL---RFSTHL--
Magnetospi : VSVDLGYPLVKEKFD-KK-EFFRF---NFGTKF--
Gluconacet : INIDLGIPVLKEKHDRTR-L-FRF---GFGQQF--
Chlamydomo : TPASIHTELAQLPGPTVS-PRLDLFHPSLGPTHS-
Phaeodacty : IQSVDSLRLRVVLG---------------------
Thalassios : FEATYAVPMRYGPRDATK--AVQF---GFGFSFG-
Saccharomy : FELNFTLPITAHENDLIR-KGFQF---GLGLAFL-
Ectocarpus : LEINYAHVLRRSPRDLHRERPLQF---GFGVSFE-
Phytophtho : LEANYSWILKAHDHDNIK-R-AQL---GLGMTFC-
Phytophtho : LEANYSWILKAHDHDNIK-R-AQL---GLGMTFS-
Phytophtho : LEANYSWILKAHAHDNIK-R-AQL---GLGMTFC-
Physcomitr : LEVNYCYLLRYQENDRIK-RGVQI---SLNSPQ--
Zea_mays_A : AEINYCYILKQFEHDRGK-TGIQF---SFSSPM--
Sorghum_bi : VEVNYCHILKQAEHDSSK-TGIQF---SFSSPM--
Oryza_sati : VEVNYCYILKQAEHDSGR-TGIQF---SFSSPL--
Arabidopsi : MELNYCHILKKQEHDRAK-SGFFM---TFSTSS--
Vitis_vini : MEVNYCYILKQFEHDHGR-TGVQF---SFSAT---
Ricinus_co : LELNYCFILKKFEYERGK-SGFRV---SFSTPS--
Populus_tr : MELNYCNILKKFGEDRGK-SGFRV---SFSSPS--
Ostreococc : IEMNYGKVVRAGTNDRVS-DGFQV---GIAAHVSM
Micromonas : IEVNYCHTLRSRPGDRVK-NGLQI---GLATALA-
Micromonas : IEVNYVKTVRAGANDRVK-DGLQV---GLATQMSI
Pichia_sti : FELNFVLPLVVHDRDYVR-KGLQY---GIGVSFL-
Hydra_magn : LEINYCIPKNAQAGDVIN-NGLQV---GVGMSFL-
Ciona_inte : IELNFCVPLRNVPTDKVV-NGLQF---GIGVTSV-
Trichoplax : LELNYCVPCRAHATDRIS-PGFQF---GVGLSFL-
Salmo_sala : LELNYCIPMGVQSGDRIC-DGVQF---GAGIRFL-
Danio_reri : LELNYCIPMGVQSGDRIC-DGVQF---GAGIRFL-
Xenopus_la : LELNYCIPMGVQSGDRIC-DGVQF---GAGIRFL-
Monodelphi : LELNYCIPMGVQRGDRIC-DGVQF---GAGIRFL-
Bos_taurus : LELNYCVPMGVQRGDRIC-DGVQF---GAGIRFL-
Mus_muscul : LELNYCIPMGVQGGDRIC-DGVQF---GAGIRFL-
Homo_sapie : LELNYCVPMGVQTGDRIC-DGVQF---GAGIRFL-
Macaca_mul : LELNYCIPMGVQTGDRIC-DGVQF---GAGIRFL-
Pediculus_ : VEFNYVIPLKAQDADVTA-EGFQF---GVGLQFL-
Apis_melli : VELNLVMPLLFVRSDVLQ-Q-FQF---GIGLQYL-
Tribolium_ : VEVNYCFPHTFEKGDQIH-PGIQF---GIGVQFV-
Laccaria_b : VEVNFGVPLVASKSDGSR-RGIQV---GMGLEFL-
Coprinopsi : VEVNLGVPLVASKSDGTR-KGIQV---GVGLEFL-
Schizosacc : FELNFTLPLMTTVKDLGR-RGFQL---GAGLEFL-
Neurospora : FELNFSLPLVVRRGEEAR-KGLQV---GVGINFL-
Nectria_ha : FELNFSLPLALRRGEVST-KGLQV---GVGINFL-
Phaeosphae : FEINFSLPLVVRAREESR-KGLSF---GVGIEFL-
Aspergillu : FELNFSLPLVLRKGEEGR-KGLQL---GIGINFL-
Ajellomyce : FELNFSLPLILRRGEEGR-KGLQF---GIGINFL-
Coccidioid : FELNFSLPLVLRKGEEGR-KGLQL---GIGISFL-
```

(e) YidC, Oxa1, Cox18/Oxa2, Alb3 amino-acid alignment (p. 17-22)

```
                     *        20         *        40         *        60         *        80         *       100         *       120         *       140
Cme_Alb3   : ----------------------------------------------------------------------------------------------------------------------------------------------
Ptr_Alb32  : ---------------------------------------------------------------------------------------------------------------------------------------------M
Tps_Alb32  : ----------------------------------------------------------------------------------------------------------------------------------------------
Ota_Alb3_1 : ----------------------------------------------------------------------------------------------------------------------------------------------
Olu_Alb3_1 : ----------------------------------------------------------------------------------------------------------------------------------------------
Ptr_Alb31  : ----------------------------------------------------------------------------------------------------------------------------------------------
Tps_Alb31  : ----------------------------------------------------------------------------------------------------------------------------------------------
Cme_Oxa1   : ----------------------------------------------------------------------------------------------------------------------------------------MGVDPA
CreAlb3_1  : --------------------------------------------------------------------------------------------------------------------------------------MSPSDLGD
CreAlb3_2  : --------------------------------------------------------------------------------------------------------------------------------------MAVSRRPA
OtaAlb3_2  : ----------------------------------------------------------------------------------------------------------------------------------------MFNAHA
OluAlb3_2  : ----------------------------------------------------------------------------------------------------------------------------------------------
OsaAlb3_a  : --------------------------------------------------------------------------------------------------------------------------------------MAGLDLHA
AthAlb3_a  : --------------------------------------------------------------------------------------------------------------------------------------MASVDIGA
PAlb3_a1   : --------------------------------------------------------------------------------------------------------------------------------------MASVDFNT
PAlb3_a2   : --------------------------------------------------------------------------------------------------------------------------------------MASIDFNS
OsaAlb3_b  : --------------------------------------------------------------------------------------------------------------------------------------MDFAEVGE
AthAlb3_b  : ---------------------------------------------------------------------------------------------------------------------------------------MSPDSFD
PAlb3_b2   : ----------------------------------------------------------------------------------------------------------------------------------------------
PAlb3_b1   : ----------------------------------------------------------------------------------------------------------------------------------------------
Syn_YidC   : ----------------------------------------------------------------------------------------------------------------------------------------------
Pma1_YidC  : ----------------------------------------------------------------------------------------------------------------------------------------------
Pma2_YidC  : ----------------------------------------------------------------------------------------------------------------------------------------------
Tps_Oxa2   : ----------------------------------------------------------------------------------------------------------------------------------------------
Ptr_Oxa2   : ----------------------------------------------------------------------------------------------------------------------------------------------
Esi0025_01 : MLTGARLSRQSRRLVGRVHGNGASLTAPAIAENRLTHHDGSDNSKRTCRGYLRPPQHLLQAQTLHVPALQQRGHRSSSVTRLGGDGSAIGAAALGGGRGLDLSAAWRGAAAAGGTVGARRSFWGRSGGDGSQAGSSGSGSDS
Mbr_Oxa2   : -------------------------------------------------------------------------------------------------------------------------------------------MAA
Ptr_Oxa1   : ----------------------------------------------------------------------------------------------------------------------------------------------
Tps_Oxa1   : ----------------------------------------------------------------------------------------------------------------------------------------------
Yli_Oxa1   : ----------------------------------------------------------------------------------------------------------------------------------------------
Cal_Oxa1   : ----------------------------------------------------------------------------------------------------------------------------------------------
Sce_Oxa1   : ----------------------------------------------------------------------------------------------------------------------------------------------
Mbr_Oxa1   : ----------------------------------------------------------------------------------------------------------------------------------------------
Hsa_Oxa1   : ----------------------------------------------------------------------------------------------------------------------------------------------
Cfa_Oxa1   : ----------------------------------------------------------------------------------------------------------------------------------------------
Mmu_Oxa1   : ----------------------------------------------------------------------------------------------------------------------------------------------
Aga_Oxa1   : ----------------------------------------------------------------------------------------------------------------------------------------------
Dme_Oxa1   : ----------------------------------------------------------------------------------------------------------------------------------------------
Pin_Oxa1   : ----------------------------------------------------------------------------------------------------------------------------------------------
Pra_Oxa1   : ----------------------------------------------------------------------------------------------------------------------------------------------
Pso_Oxa1   : ----------------------------------------------------------------------------------------------------------------------------------------------
Ota_Oxa1   : ----------------------------------------------------------------------------------------------------------------------------------------------
Olu_Oxa1   : ----------------------------------------------------------------------------------------------------------------------------------------------
Osa_Oxa1   : ---------------------------------------------------------------------------------------------------------------------------------------MALALSH
AthOxa12   : ----------------------------------------------------------------------------------------------------------------------------------------------
AthOxa11   : ----------------------------------------------------------------------------------------------------------------------------------------------
POxa11     : ----------------------------------------------------------------------------------------------------------------------------------------------
POxa12     : ----------------------------------------------------------------------------------------------------------------------------------------------
Cme_Oxa2   : ----------------------------------------------------------------------------------------------------------------------------------------------
Esi0028_00 : ----------------------------------------------------------------------------------------------------------------MSTAAGTGHSGDVATTADLGPDAMAGGADA
Mmu_Oxa2   : ----------------------------------------------------------------------------------------------------------------------------------------------
Hsa_Oxa2   : ----------------------------------------------------------------------------------------------------------------------------------------------
Cfa_Oxa2   : ----------------------------------------------------------------------------------------------------------------------------------------------
Aga_Oxa2   : ----------------------------------------------------------------------------------------------------------------------------------------------
Dme_Oxa2   : ----------------------------------------------------------------------------------------------------------------------------------------------
Yli_Oxa2   : ----------------------------------------------------------------------------------------------------------------------------------------------
Cal_Oxa2   : ----------------------------------------------------------------------------------------------------------------------------------------------
Sce_Oxa2   : ----------------------------------------------------------------------------------------------------------------------------------------------
Olu_Oxa2   : ----------------------------------------------------------------------------------------------------------------------------------------------
Ota_Oxa2   : ----------------------------------------------------------------------------------------------------------------------------------------------
Esi0170_00 : ----------------------------------------------------------------------------------------------------------------------------------------------
Pra_Oxa2   : ----------------------------------------------------------------------------------------------------------------------------------------------
Pin_Oxa2   : ----------------------------------------------------------------------------------------------------------------------------------------------
Pso_Oxa2   : ----------------------------------------------------------------------------------------------------------------------------------------------
Osa_Oxa2   : ----------------------------------------------------------------------------------------------------------------------------------------------
AthOxa21   : ----------------------------------------------------------------------------------------------------------------------------------------------
AthOxa22   : ----------------------------------------------------------------------------------------------------------------------------------------------
POxa2      : ----------------------------------------------------------------------------------------------------------------------------------------------
```

```
                    *       160         *       180         *       200         *       220         *       240         *       260         *       280
Cme_Alb3   : ----------------------------MTAG------------------------------------------------------------------------HEQTKR-SGGL-----TLW--LTGSAIALRLIT-LPLE
Ptr_Alb32  : ASVDGLVQQSAAATAPSDDGGWWGAYIQLFKT-------------------------------------------------------------------TLNAVHSTIQGPLQNVGI-EQSWGVSIAIFTTIVRTLL-VPLS
Tps_Alb32  : ------MMQHQVTLDAKDENSWWESYIQIYKN-------------------------------------------------------------------GLAFVHDNVDEPLRKLGF-DQTWGVSIFLFTAGVRALL-VPFS
Ota_Alb3_1 : --------------QPQRMEGWLA----PVSN-------------------------------------------------------------------ALEDLLFTIKGQLLDLGV-PYPTGNAIIIVTILVKMVT-YPLT
Olu_Alb3_1 : --------------APQRMEGWLA----PVSD-------------------------------------------------------------------ALEDLLFAIQGQLQGLGV-PYSTGNAIIIVTILVKFVT-YPLT
Ptr_Alb31  : --MRDLPQQSLLQDVAESDSGWFG----FLTI-------------------------------------------------------------------PIKLLLQAIHSLLLTVGLNTNSWGVAIVLLTILIKVVT-FPLT
Tps_Alb31  : --------------APNAQNGWFG----FLTL-------------------------------------------------------------------PIEGLLKLIHGGLDSMGMSSNAWGISIIAMTVVIKALT-FPLT
Cme_Oxa1   : ALQDSWHTTFALASAGAQAGLWNG----FVHL-------------------------------------------------------------------IE-TIITGTGDTLAALGV-PGSYGFAIIFLTIIVKAIT-FPLN
CreAlb3_1  : ALHRL-GAIYVLADAPQRAGGWVA----PVAD-------------------------------------------------------------------ALEQVLYALQEGLDKLHV-PYSYGYSIILLTLIVKLLT-YPLT
CreAlb3_2  : VVVKASLLDAASAALTLAEGGPID----VLAQ-------------------------------------------------------------------FFEFVLQTLDEGLESAKI-PYSYGFAIIALTVLVKVAT-FPLT
OtaAlb3_2  : ALVSSPLFDLAARLAGIQKGGWLG----PITD-------------------------------------------------------------------ALEGALRGIDGVLDGK--VPYSYGYSILLLTVLVKLAT-FPLS
OluAlb3_2  : --------------AGLQKGGWLG----PITD-------------------------------------------------------------------GLESALEGIDSVLDGR--VPYSYGFSIIVLTVLVKLAT-FPLS
OsaAlb3_a  : ALERAEAALYTLADSAQKNGGWFG----FISE-------------------------------------------------------------------ALEVVLKVLKDGLSAVHV-PYSYGFAIILLTVIVKAAT-LPLT
AthAlb3_a  : IFTRAESLLYTI-ADVQKSGGWFG----FISD-------------------------------------------------------------------AMELVLKILKDGLSAVHV-PYAYGFAIILLTIIVKAAT-YPLT
PAlb3_a1   : IISRAEGFLYTL-ADAQKNGGWFG----FISD-------------------------------------------------------------------GMEFVLKVLKDGLSSVHV-PYAYGFAIILLTIAVKVAT-LPLT
PAlb3_a2   : IISRAEGFLYTL-ADAQKSGGWFG----FISD-------------------------------------------------------------------GMEFVLKVLKDGLSAVHV-PYAYGFAIILLTVFVKVAT-LPLT
OsaAlb3_b  : LFGRVEAFLYTV-ADKEAAGDWLS----GITN-------------------------------------------------------------------SMETVLKVLKDGLSALHV-PYPYGFAIILLTVLVKAAT-FPLT
AthAlb3_b  : FIKDHAENLLYTIADTTQSNDWFS----GIAN-------------------------------------------------------------------YMETILKVLKDGLSTVHV-PYSYGFAIILLTVLVKAAT-FPLT
PAlb3_b2   : -LFGRAESIVYTIADTTQNSDWLS----GITY-------------------------------------------------------------------GLESTLKVLKDGLSAVHL-PYAYGFAIILLTVLVKAAT-FPLS
PAlb3_b1   : LFGRAESIIYTI-ADTKQNSDWLS----GITS-------------------------------------------------------------------CLESTLKVLKDGLSALHV-PYAYGFAIILLTVLVKAAT-FPLS
Syn_YidC   : -------------------MDF------GVGF-------------------------------------------------------------------LSNNVMLPILDFFYGI---VPSYGLAIIFLTLVIRFAL-YPLN
Pma1_YidC  : ----------------------------MIGY-------------------------------------------------------------------ISDNILLPILDFFYGL---VPSYGLAIVALTVVIRLAL-FPLS
Pma2_YidC  : ----------------------------MIGY-------------------------------------------------------------------ISDNILLPILDFFYGL---VPSYGLAIVALTVVIRLAL-FPLS
Tps_Oxa2   : -----------------------------------------------------------------------------------------------------------------------------MSLTNICIRTSL-LPIA
Ptr_Oxa2   : ----------MWPEVLQNVSVW------GGSG----------------------------------------------------------------------YLLKAIHA-DGVV-----PYWACFAAINVMVRIGL-FPLV
Esi0025_01 : GSGSEDSAGFPETGGAADADGWSA----PPDAWAPDEVPPEIPQDASVPGFDVGEGFSVDSAAAALDAVAGAAPDASAVGTAMTATGLSAADLGMYPHHLFMHVIEYVQA-TAGV-----PYWEAIVMVSVAARIAV-LPAV
Mbr_Oxa2   : AAVQPALSVGGTRLSLEALTLT-G----IVDG-------------------------------------------------------------------TE-AALVGLQA-QTQA-----PWPVAVLLTAGLVRTVVGAPAA
Ptr_Oxa1   : --------------MQPWDPTWYN----LADQ-------------------------------------------------------------------AI-LAVKSLHE-FSGL-----EYGWSIVGVTVILRLCL-FPVM
Tps_Oxa1   : -------------MSPFEPTWW------PSDQ-------------------------------------------------------------------LL-LLLNHVHDVLPAY-----PYAVTIGVTTLAARLLL-FPIY
Yli_Oxa1   : ------------MDTGSLWSLW------PSDI-------------------------------------------------------------------YL-NLLEHVHV-YTGL-----PWWAAIASTTVIVRVLL-FPLF
Cal_Oxa1   : -------------MQIGLAQGW-G----PTSL-------------------------------------------------------------------IE-RLLEVTHV-YTGL-----PWWGTIVVATIAVRLVL-FPLY
Sce_Oxa1   : --------------MFGLAQTWYW----PSDI-------------------------------------------------------------------IQ-HVLEAVHV-YSGL-----PWWGTIAATTILIRCLM-FPLY
Mbr_Oxa1   : -----------------GLGSFAT----PVGL-------------------------------------------------------------------IQ-NLVEFNTV-MLDA-----PWAVGICATTILMRTLM-LPLV
Hsa_Oxa1   : -------------MPELGLGSY-T----PVGL-------------------------------------------------------------------IQ-NLLEFMHV-DLGL-----PWWGAIAACTVFARCLI-FPLI
Cfa_Oxa1   : --------------PELGLGSY-T----PVGL-------------------------------------------------------------------IQ-NLLEFMHV-NLGL-----PWWGAIAACTVLARCLV-FPLI
Mmu_Oxa1   : --------------SELGLGSY-T----PVGL-------------------------------------------------------------------IQ-NLLEYIHV-DLGL-----PWWGAIATCTVLARCLV-FPLI
Aga_Oxa1   : ---------------TLGLGGW-T----PVGI-------------------------------------------------------------------VQ-NCMEFLHI-GLDL-----PWWGCIAIGTVCVRTLL-FPLV
Dme_Oxa1   : --------------MSIGLGGW-S----PVGM-------------------------------------------------------------------VQ-NCLEFLHC-TWDI-----PWWGTIAIGTLAVRTII-FPLV
Pin_Oxa1   : ---------------SVSDLGY------SLSD-------------------------------------------------------------------MAIRSLDVIHS-TTGL-----PWWATIIATTVAVRTVF-FPVT
Pra_Oxa1   : -------------MVSVSDLGY------SLSD-------------------------------------------------------------------IAIRSLDVIHA-TTGL-----PWWATIIATTVAVRTAF-FPIT
Pso_Oxa1   : ---------------SVSDLGF------SLSD-------------------------------------------------------------------IAIRSLDVIHA-TTGL-----PWWATIIATTVAVRTVF-FPVT
Ota_Oxa1   : -------------MDPVAVQSW------PTTA-------------------------------------------------------------------ALMYAMEYFHV-AHGL-----EWWLAIVGATVFMRTVT-FPLV
Olu_Oxa1   : ---------------PVASQSW------PTTA-------------------------------------------------------------------ALMYAMEYFHV-AHGL-----EWWLAIVGATVFMRTIT-FPLI
Osa_Oxa1   : PATAPAAAAEAVAPVAAAADSF-F----PVAA-------------------------------------------------------------------LQ-HVIDYIHT-FTGL-----NWWACIALATVLIRSAT-VPLL
AthOxa12   : --MARGIDCRHMAAIAAADSAF------PVAA-------------------------------------------------------------------LQ-HLIDAVHS-FTGL-----NWWASIALTTVLIRGVT-IPIL
AthOxa11   : ---MAQTLSYRYMQTLAAADSFF-----PIAA-------------------------------------------------------------------LQ-QCIDMVHT-FTGF-----EWWASIVVATILIRSST-VPLL
POxa11     : --------------AIAAADSF-L----PVAV-------------------------------------------------------------------LQ-HAIDAVHN-FTGF-----NWWASIVVTTLLIRSFT-LPLL
POxa12     : ---MARSLDCRDMQAIAAADSY-F----PVAA-------------------------------------------------------------------LQ-HVIDAVHS-FTGF-----NWWASIIVTTLLIRGAT-VPLL
Cme_Oxa2   : ------------MTAALSAERNWYD----PVV----------------------------------------------------------------------QGIIMFQE-YTDL-----PWWATVAAVTVLARILV-LPLT
Esi0028_00 : AMAAAGTADQAQAALEVAPETFKMALYTPPQV-------------------------------------------------------------------AM-MAVDYVHA-TTGM-----PYWMTIVAITVGIRTAI-LPIG
Mmu_Oxa2   : -------------MGYEALAAS-A----PVRT-------------------------------------------------------------------AE-EVLLGAQE-ATGL-----PWWSNIILSTVALRGAVTLPLA
Hsa_Oxa2   : -------------MGYEALAAS-S----PVRV-------------------------------------------------------------------AE-EVLLGVHA-ATGL-----PWWGSILLSTVALRGAVTLPLA
Cfa_Oxa2   : -------------MGYEALAAS-A----PVRG-------------------------------------------------------------------AE-EMLLGLHA-AAGL-----PWWACIGLGTVALRGAVTLPLA
Aga_Oxa2   : --------------FWQTLSQS-A----PVAY-------------------------------------------------------------------VQ-QGMINLHD-LTGL-----PWWATVILTTVGLRTLVTLPLA
Dme_Oxa2   : -------------MYWQTLSNS-T----PVAY-------------------------------------------------------------------MQ-DVLIKIHD-YSGL-----PWWASIVLSTFLFRSVVTLPLT
Yli_Oxa2   : -------------MTVRQLSTV-D----IVRP-------------------------------------------------------------------VE-TALNAIHD-FSGL-----PWWAVIPLVTLTLRSTVTLPIA
Cal_Oxa2   : -------------MRNFSIDHN-A----IINT-------------------------------------------------------------------MT-SSFQTVHE-FSGL-----PWWALIPLTTFTLRSVWTLPLA
Sce_Oxa2   : -------------MTPSTKRSF-S----LFQS-------------------------------------------------------------------VA-DTFLTVHE-ASHI-----PWIVLVPLTTMTLRTLVTLPFS
Olu_Oxa2   : --------------------------------------------------------------------------------------------------MS-DSMTHLHA-ATGA-----PWFVALAASAALVRAAT-APIS
Ota_Oxa2   : --------------------------------------------------------------------------------------------------------MSTLHH-ASGL-----PWCATLAVSALCARLVT-APVA
Esi0170_00 : ---------------------M-H----VMGG-------------------------------------------------------------------VQ-SGVQAVHH-TTGL-----PWWATIAVATIGVKISL-LPVV
Pra_Oxa2   : -------------MSTTGHEPW-A----IVQG-------------------------------------------------------------------VQ-SVLETVHT-TTGL-----PWWATLLLSGVTLRAAV-FPFY
Pin_Oxa2   : -------------MNGTGNEPW-A----IVQG-------------------------------------------------------------------VQ-SVLETVHT-TTGL-----PWWATLLLSGVTVRAVI-FPFY
Pso_Oxa2   : -------------MSGTGGEPW-A----IVQG-------------------------------------------------------------------VQ-SVLEAVHT-TTGL-----PWWATLMLSGVTVRAAI-FPFY
Osa_Oxa2   : ------------MADGGSASGF------AVSS-------------------------------------------------------------------LI-DILDGFHN-LTGI-----PWWITISLSTVAMRLLI-LPVL
AthOxa21   : -------------MEIPTDDSS-L----PVLA-------------------------------------------------------------------VV-DFLEGFHE-FTGL-----PWWMIIASSTVAVRLAL-LPLL
AthOxa22   : --------------MLQDLSNYDY----LTQP-------------------------------------------------------------------VI-SLLDSYHD-ITGL-----PWWVVIATSTVAFRTAL-LPIL
POxa2      : --------------MTDGVVNGIL----PVDS-------------------------------------------------------------------MI-WLLDSYHD-LTGL-----PWWIIIASSTLAMRLTL-FPLH
```

```
                    *       300         *       320         *       340         *       360         *       380         *       400         *       420
Cme_Alb3   : AYQARQWLRLREAQR----LASAAYQLLTRSSQVQLASNDDLL--------SSLTCLQRKALQRY--GTSRWRALVWGRVVGLPAFVFAALRVRQL----------------------------------------------
Ptr_Alb32  : IEQSKSAEYIKSLKP----YVADIKAKYKNNQ-------------------EAQNRATAKLYEDA--QQNPLAGCFV-ALIQLPVFLGLYRGVRLL----------------------------------------------
Tps_Alb32  : IQQSKSSEYMKALKP----YQQKIKEKYTDK--------------------NMQNRAISKLFEDA--QANPLAGCVT-SFAQIPIFLGLYRSVTRL----------------------------------------------
Ota_Alb3_1 : KDQVVSSLNMKNLQP----QIAAIREKYEDDQ-------------------ERMNKEINRVYEEN--GVNPLAGCGP-ALLSFPVLAGLYRAFNNA----------------------------------------------
Olu_Alb3_1 : RDQVVSSLNMKNLQP----QIAAIREKYEDDQ-------------------ERMNKEINRVYEEN--GVNPLAGCGP-ALLTFPVLAGLYRAFNNA----------------------------------------------
Ptr_Alb31  : KTQLESTNKMQAMQP----AIKELQAKYQSNP-------------------EVMNQKIAEFYQTN--EINPLAGCLP-SIVQIPVFIGLYRAVLEL----------------------------------------------
Tps_Alb31  : KSQLESTNKMQALQP----TIKSLQAKYQSNP-------------------EVMNQKIAEVYQTN--EVNPLAGCIP-SLVQIPVFIGLYRAVLNL----------------------------------------------
Cme_Oxa1   : YKQMKSTMAMQALAP----KVRELQARYRDNP-------------------QLLNLETARLYQEA--KVNPLTGCLP-VFVQLPVWIALYRALMNL----------------------------------------------
CreAlb3_1  : KQQVESAMAVQALKP----RIDLIKDRFGEDK-------------------DKIQKETSVLYEQA--GVNPLAGCLP-TLATIPIFIGLFSSLTNV----------------------------------------------
CreAlb3_2  : QKQVESTLSLQALQP----RVKELQAKYADDP-------------------ENLQLETARLYKEA--GVNPLAGCFP-TLATIPVFIGLYNALSNA----------------------------------------------
OtaAlb3_2  : KQQVESSIQMQAMQP----RIKELQAMYANDP-------------------ERLQLEQARLYREA--GFNPLAGCLP-LFATLPVFIGLYRALSNA----------------------------------------------
OluAlb3_2  : KKQVESSMQMQAMQP----RIKELQAMYANDP-------------------ERLQMEQARLYKEA--GFNPLAGCLP-VFATLPVFIGLYRALSNA----------------------------------------------
OsaAlb3_a  : KQQVESTLAMQNLQP----QIKAIQQRYAGNQ-------------------ERIQLETARLYKQA--GVNPLAGCFP-TLATIPVWIGLYQALSNV----------------------------------------------
AthAlb3_a  : KQQVESTLAMQNLQP----KIKAIQQRYAGNQ-------------------ERIQLETSRLYKQA--GVNPLAGCLP-TLATIPVWIGLYQALSNV----------------------------------------------
PAlb3_a1   : KKQVESTLAMQNLQP----KIKAIQQRYAGNQ-------------------ERIQLETSRLYRQA--GVNPLAGCFP-TLATIPVWIGLYQALSNV----------------------------------------------
PAlb3_a2   : KKQVESTLAMQNLQP----KIKAIQQRYAGNQ-------------------ERIQLETSRLYRQA--GVNPLAGCFP-TLATIPVWIGLYQALSNV----------------------------------------------
OsaAlb3_b  : KKQVESAIAMRSLQP----QVKAIQERYAGDQ-------------------ERIQLETARLYKLS--DVDPLAGCLP-TLVTIPVWIGLYRALSNV----------------------------------------------
AthAlb3_b  : KKQVESAMAMKSLTP----QIKAIQERYAGDQ-------------------EKIQLETARLYKLA--GINPLAGCLP-TLATIPVWIGLYRALSNV----------------------------------------------
PAlb3_b2   : KKQVESAMAMRSLQP----QIKAIQQRYAGDQ-------------------ERIQLETARLYKLA--GINPLAGCLP-TLATIPVWIGLYRALSNV----------------------------------------------
PAlb3_b1   : KKQVESAMAMRSLQP----QIKAVQQLYAGDQ-------------------ERIQLETARLYKLA--GINPLAGCLP-TLATIPVWIGLYRALSNV----------------------------------------------
Syn_YidC   : VGSIRNMRRMKVINPLMQRRMREIQEKYRDDP-------------------QKLREAQAKLYSEL--GVNPLGGCLP-LLIQMPVLFALFATLRGSPFAATYDLPAEVAAEVVPTPYVSPSKNIFVTDSVHKPVVLVAPKGT
Pma1_YidC  : AGSIRSARRMRIAQPAMKKRQDEIKSRYAKDP-------------------QKQQEELGKVMKEF--G-NPLSGCLP-LLVQMPILFALFATLRGSPFADPYLLPSDQIAAIEPKPFTSSKHSIFISETKHFPVIASLPSGT
Pma2_YidC  : AGSIRSARRMRIAQPAMKKRQDEIKSRYAKDP-------------------QKQQEELGKVMKEF--G-NPLSGCLP-LLVQMPILFALFATLRGSPFADPYLLPSDQIAAIEPKPFTSSKHSIFISETKHFPVIASLQSGT
Tps_Oxa2   : IQGAKTSVSFGKVAP----EVQYLISSFQRDFRLLREGMEAAQAQMSLL--KTTVATLRGIFRLH--KVNLLDIFKS-PLMQIPVFWYFAIDIRKI----------------------------------------------
Ptr_Oxa2   : LYGAQTSTRFAKVVP----EVQFLLSLFQADWQRLRQNPLRERLMLM----RTNLGTLGGIYKLH--GIHPMAVFLS-PLLQVPLFWYVSVDLRKI----------------------------------------------
Esi0025_01 : ATFLGMSKRLNMIKP----EMAVHQGKMQDIKNRMEANPEIKEAAMAEM--MLVSQEMGNLLKQH--RIHFPKMMLS-MFAQFPVFISLFLATRDM----------------------------------------------
Mbr_Oxa2   : WYQAHWSSRVELQRR----RLMQWADAIGHQVMRRKANTDAEAENLIQ---EQVRAQQKVLMDEI--GWRRWKLVLP-ALVQVPIFITVSLALRRL----------------------------------------------
Ptr_Oxa1   : VASQQTSSRMAHLQP----ELQQIKARYEALGTPSRQDQ------------LQFSGQMKAIFAKY--KVKPFRAFAG-PVIQMPLFMGMFFGLRKM----------------------------------------------
Tps_Oxa1   : AAGQRNSSRMAHMQP----EMKKIMDATPKQPDQATQ--------------QKVMAQTRALWRKY--DCNPMKGLVV-PLASFPFFMGMFFGLKKA----------------------------------------------
Yli_Oxa1   : VQAANEQGKMSEVKP----ELNVIDEKLKSAANMTEM--------------QMVAHEKKKILKKY--GISQMKLFYP--MAMFPLTIGIFLGIRRM----------------------------------------------
Cal_Oxa1   : VRASSNATKMSKIKP----QIDELLQQIKTGDTVDQ---------------MRAMEKRRLIMKEN--GVSTLATLFP--AVQLPLAYGFFQALRKM----------------------------------------------
Sce_Oxa1   : VKSSDTVARNSHIKP----ELDALNNKLMSTTDLQQG--------------QLVAMQRKKLLSSH--GIKN-RWLAA-PMLQIPIALGFFNALRHM----------------------------------------------
Mbr_Oxa1   : FGSMRNNTILMNIQP----QLQLHSQRIRECQTRNDHDGA-----------AQAAANLNGLFKEH--GVHPLKGLLP-LFVQAPVFISFFMALRQM----------------------------------------------
Hsa_Oxa1   : VTGQREAARIHNHLP----EIQKFSSRIREAKLAGDHIEY-----------YKASSEMALYQKKH--GIKLYKPLIL-PVTQAPIFISFFIALREM----------------------------------------------
Cfa_Oxa1   : VKGQREAAKIHNHLP----EIQKFSTRIREAKLAGDQAEF-----------YKASSEMTFYQKKH--DVKLFRPLIL-PLTQAPIFISFFIALREM----------------------------------------------
Mmu_Oxa1   : VKGQREAAKIHNHMP----EMQKFSARIREAKLAGDQAEF-----------YKATIEMTRYQKKH--DIKLLRPLIL-PLTQAPVFISFFIALREM----------------------------------------------
Aga_Oxa1   : IASQRNAAKMNNYMP----QLQVLQMKMTEARQAGNAIDS-----------ARYGQEMVLFMKEK--NLNPLKNMLV-PLAQAPIFISFFMGLREM----------------------------------------------
Dme_Oxa1   : ILAQRNSAKMNNNMP----QMQMLQLKMTEARQSGNAIES-----------ARYAQEMMLFMREK--GVNPLKNMVV-PLAQAPLFISFFMGLRQM----------------------------------------------
Pin_Oxa1   : VISMRNAAKMKLFQP----DMEKLKAQMDANPTQSPEST------------KEFQTKYKALMKKH--DVNPFKSVLT-PLSQIPVFLGFFWGLQDI----------------------------------------------
Pra_Oxa1   : VVSMRNAAKMKLFQP----DMEKLRDEMDANPTRDEKST------------REFQTKYKALMKKH--GVNPFKSMLT-PLAQIPVFLGFFWGLQDI----------------------------------------------
Pso_Oxa1   : VISMRNAAKMKLFQP----DMEKLRDEMDSNPTQTPETA------------QEFQKKYKALMKKH--DVNPFKSMLT-PMAQIPVFLGFFWGLQDI----------------------------------------------
Ota_Oxa1   : VMQMRNTARMQLCKP----ELEALQAKMKSNPSQDPELA------------TAYYAEMQKVWKKY--DVNPFKSFAP-VLINAPVFISFYFAISKM----------------------------------------------
Olu_Oxa1   : VMQMRNTAKMQLCKP----ELEALQAKMKSNPQQDPELA------------NAYYKEMQKVWKKY--DVNPVKSFAP-ILINAPVFISFFFAISKM----------------------------------------------
Osa_Oxa1   : VNQLKATQKLNAIRP----EMEAIKEEMNAMDPKSA---------------KEGKAKMTALFQKH--GVSPFTPLKG-LLIQGPIFMSFFFAIRNM----------------------------------------------
AthOxa12   : LNQLKATYKLNVLRP----QLEELRQEMSTKAQDPEAM-------------AEGQRRMQLLFKEH--GVTPFTPLKG-LIIQGPIFISFFFAIRNM----------------------------------------------
AthOxa11   : IKQMKDTTKLALMRP----RLESIREEMQNKGMDSVTM-------------AEGQKKMKNLFKEY--GVTPFTPMKG-MFIQGPLFICFFLAIRNM----------------------------------------------
POxa11     : INQLKATSKLSIVRP----HLEEVKQRVERQGMDPTAV-------------SEGQKEMKKLFKEH--GVSPFTPLKG-LFIQAPVFISFFLAITNM----------------------------------------------
POxa12     : INQLKATTKLTLMRP----HLEEIRQQMSDKAMDPMAL-------------AEGQKQMKKLFKEY--GVSPLTPLKG-LFIQGPIFVSFFLAISNM----------------------------------------------
Cme_Oxa2   : LNTFRNAARMQSIKP----DVDAIKERMQAAMHSGDQQRI-----------RALQQQVFRLLREN--QISPLRSLVN-PLVQMPLFISFFLGLRKI----------------------------------------------
Esi0028_00 : LLAARNGARTAAMKP----EMDELQAAIKGDQQSSQPRKA-----------DRYRQETKALFQKH--KASLVMNAAL-PIVQLPLFIGFFLGLRRM----------------------------------------------
Mmu_Oxa2   : AYQHYILAKVENLQP----EIKDIAKRLNQEVAVCARGWSKRVARLTYL--KNMRRLVSELYVRD--NCHPFKATVL-VWVQLPMWVFISVALRNL----------------------------------------------
Hsa_Oxa2   : AYQHYILAKVENLQP----EIKTIARHLNQEVAVRANGWSKRDARLTYL--KNMRRLISELYVRD--NCHPFKATVL-VWIQLPMWIFMSFALRNL----------------------------------------------
Cfa_Oxa2   : AYQHYILAKVENLQP----EIKNIARHLNQEVAVRANKWSKRVARLTYL--KNMRRLVSELYVRD--NCHPFKATIL-VWIQFPMWIFMSVALRNF----------------------------------------------
Aga_Oxa2   : VYQNKILARLEQISL----EMPELIKELKAETAYAKNWTEKEARIMYN---HSLKKQWNNLIVRE--NCHPAKTMVL-LWGQIPLWIVQSVAIRNL----------------------------------------------
Dme_Oxa2   : IYQHKITARIEKIAL----EMPAIVEELKKEAAM-AHKWSEKQTQIVYR--RSIKKQWQNLIVRD--NCHPMKTMIV-LWGQIPLWIFQSVALRNL----------------------------------------------
Yli_Oxa2   : ISTRLRAQKQHELRP----LISALGPILRAKLAFNANAPQIEMLAM-----KERRKRRVKLYKEH--GCEMWKSLFIGPLVQLPIWITMSLAVRAM----------------------------------------------
Cal_Oxa2   : ILQRKRIQKQSQLRP----LVSAMNPILKLNLARRQSTIQSKYEQILLLSAKEARKRQKELFAKN--GVQLWKNFIL-PAFQVPLWIMMSITMRDL----------------------------------------------
Sce_Oxa2   : IWQRRRILKQQELRK----LVQPITPIIKLRLAAVNSSNPLQPEQITLLAVKETRKRQKKLFKKY--NVPLWKNALL-PMVQIPLWVTVSMGIRTL----------------------------------------------
Olu_Oxa2   : ARTIKASATVSAASA-----LAKSRKRGNSEHVGVRDV-------------LDAIGELRGKAPGT--GAHPAWLVAG-PLAQIPFFLCAVMAVRRL----------------------------------------------
Ota_Oxa2   : ARTTKASATVSAASA----LAKATKQGDAERVSI-----------------KDVLEAMKELRERSGVGAHPAWLVAG-PLAQIPLFACAMMAVRRL----------------------------------------------
Esi0170_00 : VYQAGHMDRMRAAWP----EIQILRGYLATSLEEIPQERVLERWRKY----KVFFSGARGVLGLH--GTH-LRGMFATPLVNLPVFITFVWSIRGM----------------------------------------------
Pra_Oxa2   : VFQIQAMQRLMHARP----DFSKLYSAYKYARTFTPGSDHKGHLDAV----LLGRQGMKAVMKKY--NTRPVQTIMG-SLAYIPIFVLMAYSARDM----------------------------------------------
Pin_Oxa2   : VLQIQAMQRLIQARP----DFSKLHSAYKYARTFTPGSDHKGHLDAI----LLGRQGLKAVMKKY--NTRPIQTIMG-SVAYIPIFVLMAYSARDM----------------------------------------------
Pso_Oxa2   : VFQIQAMQRLVQAKP----DFSKLYSAYKYARTFTPGSDHKGHLDAI----LLGRKGVKAVTKKY--NTRPVQTVMG-SVAYIPIFVLMAYSARDM----------------------------------------------
Osa_Oxa2   : ITQIKKAAKIGKLLP----ELPPPFPPPLSGRSFRDQF-------------SLYQKKRREL------GCPSFLW----NWAYFSIQIAFHGTHTKH----------------------------------------------
AthOxa21   : ILQLKKLKTISELLP----KLPMPIPETPTLKGSIDQF-------------SHFLKESRAI------GCPSFLWFFPYLSVQLPCFFLLMASIRKM----------------------------------------------
AthOxa22   : ILQRKQTKRISQFLP----KLPHFWPPQGSGRSVLDQL-------------KLFRKERKDI------GCPSFLWVPAYFSIQISCFFLWITSIRRM----------------------------------------------
POxa2      : VLQMHKIKKISRSFS----KLPPLFPPPLSGRSYIEQI-------------SLFRNERRAI------GCPSYLWFLAFLSVQIPCFLLWMTSIRRM----------------------------------------------
```

```
                 *        440       *        460       *        480       *        500       *        520       *        540       *        560
Cme_Alb3    : --------------------------------------------------------------ALAGDLGGTLPG---LWLKDLS--------EPDPF--YLL---------------------P---LCNMVLMLRNLEYSF
Ptr_Alb32   : ------------------------------------------------------------------AMDGVLEE-PFLWIPSLEGPVAP---PNFQGLDWLVQGWVNGAPALGWETTLAFLIMP---VILVVLQSVTMQVLQ
Tps_Alb32   : ------------------------------------------------------------------AQEGRLDE-PFLWIPSLQGPVTAETNYRGTE--WLTQGWVDGVPPLSWETTLAFCVMP---VLLVLGQSFTMNVLQ
Ota_Alb3_1  : ------------------------------------------------------------------GIDGAFDE-PWFFLPSLAGP------TDARDLSWLLPLDADLAPPIGWDDASLYLLFP---IMTTLSQFVSMEVLK
Olu_Alb3_1  : ------------------------------------------------------------------GIDGAFDE-AWFFLPSLAGP------TDARDLSWLLPLDSDYAPPIGWEEASLYLIFP---VLTTISQFVSMEVLK
Ptr_Alb31   : ------------------------------------------------------------------AQANKLDE-SFLFLPNLEGPTYGA--EPGSAADWILKGWTDGVPSLGWPDTIAFLILP---VFLVISQYLSMELMQ
Tps_Alb31   : ------------------------------------------------------------------AKENALDE-PFLFLPNLEGPTYGA--DPAHGSDWLFKNWVDGVPGLGWEDTIAFLSIP---VFLTISQVISMNMMQ
Cme_Oxa1    : ------------------------------------------------------------------AADNRLDQ-GFFWLPSLEGPV-----RQGQGLSWLFPF-QNGAPPIGWHDAIAYLVLP---CLLVVSQSISQKILQ
CreAlb3_1   : ------------------------------------------------------------------ANDGLLDQ-GFYFVPSLAGPTTMAMRQSGLGTSWLWPLGPDGAPPIGWEDAAAYLTLP---LLLVAVQYASSSVTS
CreAlb3_2   : ------------------------------------------------------------------AKEGLLTE-GFFWIPSLGGPT-----TIGGGLEWLVPF-ENGAPPVGWANAAAYLVMP---VLLVASQYASQKIIS
OtaAlb3_2   : ------------------------------------------------------------------AAEHLLDD-GFYWIPSLGGPTSIAARNDGNGFAWLWPF-VDGHPPLGWYETGCYLVLP---VLLVVSQFVSQTIIS
OluAlb3_2   : ------------------------------------------------------------------ASEGLLTD-GFYWIPSLGGPTSIASRNAGSGFAWLWPF-VDGHPPLGWHDTTAYLVLP---VLLVASQYVSQQIVS
OsaAlb3_a   : ------------------------------------------------------------------ANEGLLTE-GFFWIPSLGGPTTIAARQSGAGISWLLPF-VDGHPPLGWHDTICYLVLP---VLLVASQFVSMEIMK
AthAlb3_a   : ------------------------------------------------------------------ANEGLFTE-GFFWIPSLGGPTSIAARQSGSGISWLFPF-VDGHPPLGWYDTVAYLVLP---VLLIASQYVSMEIMK
PAlb3_a1    : ------------------------------------------------------------------ANEGVLTE-GFFWIPSLGGPTTIAARQSGSGISWLFPF-VDGHPPLGWHDTAAYLVLP---VLLIASQYVSMEIMK
PAlb3_a2    : ------------------------------------------------------------------ANEGVLTE-GFFWIPSLGGPTTIAARQSGSGISWLFPF-VDGHPPLGWNDTAAYLVLP---VLLVVSQYVSMEIMK
OsaAlb3_b   : ------------------------------------------------------------------ANEGLLTE-GFFWIPSLAGPTTIAARQSGQGISWLFPF-TDGHPPLGWSDTLAYLVLP---VLLVISQYVSSQVMQ
AthAlb3_b   : ------------------------------------------------------------------ADEGLLTE-GFFWIPSLAGPTTVAARQNGSGISWLFPF-IEGHPPLGWPDTLAYLVLP---LLLVFSQYLSIQIMQ
PAlb3_b2    : ------------------------------------------------------------------ANEGLLTE-GFFWIPSLAGPTAIASRQNGSGISWLFPF-VDGHPPLGWSDTVAYLVLP---VMLVVSQYISVQIMQ
PAlb3_b1    : ------------------------------------------------------------------ANEGLLTE-GFFWIPSLAGPTTIAERQNGSGISWLFPF-VDGQPPLGWSDTVAYLVLP---AMLVVLQYMSVQIMQ
Syn_YidC    : KIAVGEQVQFRLQGPGGKPFEQLVAEAGGDPTLRPTWKITKGEERAQIQPDGTLLALQPGEVTVEVAIPGLASDTGFLFIDKLGRVG-----AFDPTIHWDI---------------------IGMIVLFGVSIYLNQSLTN
Pma1_YidC   : KLGVGDKAQIKLQTLTGESFTSRLSGVEGGTKFTPTWSVTKGDDLVKVSADGTVQALAEGDATVQGKIPGLAAQSGFLFIKALGQVGF----YVDGEINWDI---------------------A---ILVGGFGLTLLVSQI
Pma2_YidC   : KLGVGDKAQIKLQTLTGESFTSRLSGVEGGTKFTPTWSVTKGDDLVKVSADGTIQALSEGDATVQGKIPGLAAQSGFLFIKALGQVGF----YVDGEINWDI---------------------A---ILVGGFGLTLLVSQI
Tps_Oxa2    : -------------------------------------------------------------INGSDLAQQLVDS-SFLWITDLT--------EPDPW--YGL---------------------P---IITGLLLYWNVETAV
Ptr_Oxa2    : -------------------------------------------------------------VNGLDLAQQLVES-SVAWVPDLT--------EADPW--FGL---------------------P---VLAGLVMYANVEVAI
Esi0025_01  : ----------------------------------------------------------------GTYFPGYMTG-GLDWMMNLN--------APDPT--WTL---------------------P---ILTSGSMILLMELGS
Mbr_Oxa2    : -------------------------------------------------------------QDSDLYQADLASQ-GPLWTASLT--------EHDI---LLL---------------------P---LVLGITNWFTLELST
Ptr_Oxa1    : ---------------------------------------------------------------PSIFPEELSTG-GMYWFTDLT--------ASDPL--YIL---------------------P---FTSALSFLALIELGK
Tps_Oxa1    : ----------------------------------------------------------------PDYFPDLLSNGGLFWFTDLT--------QADPL--MIL---------------------P---VLSAGTFLVMTELTK
Yli_Oxa1    : --------------------------------------------------------------CEIGGVQGLSTE-GVLWFQNLA--------APDPY--LGL---------------------Q---VITAAMYMASIRLGS
Cal_Oxa1    : ---------------------------------------------------------------ANHNVEGFSDQ-GYAWFQNLI--------EVDPY--LGL---------------------Q---AISAAAIIAVVRVGG
Sce_Oxa1    : -------------------------------------------------------------ANY--PVDGFANQ-GVAWFTDLT--------QADPY--LGL---------------------Q---VITAAVFISFTRLGG
Mbr_Oxa1    : ---------------------------------------------------------------ANLPIESMKTG-GLLWFTDLT--------AADPY--YVL---------------------P---VIASATMLATIEFGS
Hsa_Oxa1    : ---------------------------------------------------------------ANLPVPSLQTG-GLWWFQDLT--------VSDPI--YIL---------------------P---LAVTATMWAVLELGA
Cfa_Oxa1    : ---------------------------------------------------------------ANLPVPSLQTG-GLLWFQDLT--------LSDPT--YIL---------------------P---LVVTATMWGVLELGA
Mmu_Oxa1    : ---------------------------------------------------------------ANLPVPSLQTG-GLWWFQDLT--------VSDPI--YVL---------------------P---LVVTATMWCVLELGA
Aga_Oxa1    : ---------------------------------------------------------------ANTPVESMRDG-GLFWFTDLT--------ICDQF--YAL---------------------P---IITSLTLFATIELGT
Dme_Oxa1    : -------------------------------------------------------------ANA--PVESMRDG-GLFWFTDLT--------MADPF--YLL---------------------P---LITSATLYLTIEIGT
Pin_Oxa1    : ----------------------------------------------------------------SKYFPEYAHE-GIGWVPDLS--------VADPT--LAL---------------------P---VISSALMAASVELGG
Pra_Oxa1    : ----------------------------------------------------------------SKYFPEYAHE-GIGWVTDLS--------ASDPT--MAL---------------------P---IISSALMVASVELGG
Pso_Oxa1    : ----------------------------------------------------------------SKYFPEYAHE-GIGWVTDLS--------AADPT--MAL---------------------P---CISSALMVASVELGG
Ota_Oxa1    : ----------------------------------------------------------------AAGVPSFETG-GPSMYPDLS--------IADPT--YSL---------------------P---ILSSLTFLASVELGT
Olu_Oxa1    : ----------------------------------------------------------------AQGVPSFESG-GPSMYPDLS--------MADPT--YSL---------------------P---ILSSLTFLASVELGA
Osa_Oxa1    : ----------------------------------------------------------------IDKVPSMKGG-GSLWFTDLT--------TPDPL--YIL---------------------P---VLTALIFLVTVELNL
AthOxa12    : ----------------------------------------------------------------AEKVPSFKTG-GTLWFTDLT--------TTDTT--YIL---------------------P---LLTAVTFLIMVESNM
AthOxa11    : ----------------------------------------------------------------AEKVPSFQTG-GALWFTDLT--------TPDSL--YIL---------------------P---VITGLTFLITVECNA
POxa11      : ----------------------------------------------------------------AEKVPSFKSG-GAFWFVDLT--------TADDL--YIF---------------------P---VLTALTFLITVECNT
POxa12      : ----------------------------------------------------------------TEKVPSFKSG-GAYWFLDLT--------TPDSL--YIL---------------------P---ILTGLTFWITVECNM
Cme_Oxa2    : ---------------------------------------------------------------AKIYPDELRNG-GLGWFRDLS--------LPDPF--YGL---------------------P---ALTSATMLFMIQMGT
Esi0028_00  : ----------------------------------------------------------------PDVVPEFATG-GVLWFQDLG--------APDPY--MIF---------------------P---VMTGVMMMAMAELGG
Mmu_Oxa2    : ------------------------------------------------------------STGATHVQEQLAAG-GTLWFPDLT--------AVDST--WIL---------------------P---VSVGVVNLLIVEIFA
Hsa_Oxa2    : ------------------------------------------------------------STGAAHVQEQLATG-GILWFPDLT--------APDST--WIL---------------------P---ISVGVINLLIVEICA
Cfa_Oxa2    : ------------------------------------------------------------STGAAHVQEQLATD-GILWFPDLT--------ALDST--WIL---------------------P---ISVGVINLLIVEIFA
Aga_Oxa2    : -----------------------------------------------------------VSMLDEAAYTELTLG-GFGWIPNLT--------ELDHS--LIF---------------------P---VALGVINLSIIEIQA
Dme_Oxa2    : ----------------------------------------------------------VYMLDQ-AVTTEMTIG-GFGWIPNLT--------VVDNS--YIL---------------------P---VALGLINLAIIEVQA
Yli_Oxa2    : -------------------------------------------------------------CGWTVVVKSMGTE-GALWFPDLL--------MMDHS--GVL---------------------P---AAVGIITLLNVELTT
Cal_Oxa2    : ------------------------------------------------------------SGWSWNLDPSLYEE-GILWFQDLS--------IADPM--HVF---------------------P---VILGITALCNIEWTL
Sce_Oxa2    : ------------------------------------------------------------------TETQLIESFYPSWFSALG--------LSSPLVPLLA---------------------P---ILVGTLAVLNVELNG
Olu_Oxa2    : --------------------------------------------------------------AADGANNGLVTG-GALWFQDLTVPAVDVAMTAPMGPGGIL---------------------P---VLTAAALFANVNANF
Ota_Oxa2    : --------------------------------------------------------------ASEGGSNGLISG-GVFWFSDLTLPAMDIATSAPMGPGAVL---------------------P---IVTAGALFANVNANF
Esi0170_00  : --------------------------------------------------------------LRDGTVPGLDTG------------------EADTVHSLML---------------------P---IIGTLCTYTSLEIVK
Pra_Oxa2    : --------------------------------------------------------------VRSGNFSGFDTG-GLLFWKNLV--------ETDST--FIL---------------------P---ILAATSTYGSLELSM
Pin_Oxa2    : --------------------------------------------------------------VRSGNFAGFDSG-GLLFWKNLM--------ETDST--YVL---------------------P---ILAATSTYGNLELSV
Pso_Oxa2    : --------------------------------------------------------------VRSGNFAGFDSG-GLLFWKNLM--------ETDST--YIL---------------------P---IIAAASTYGNLELSI
Osa_Oxa2    : -------------------------------------------------------------------------------YPGIF---------------------------------------G---VLAKYYR--------
AthOxa21    : ---------------------------------------------------------------SLDGHPGFDSG-GVLWFQNLS--------DLPGGSFPVF---------------------P---ILIATFHYINIQISF
AthOxa22    : ---------------------------------------------------------------SLDHHPGFDSG-GALWFQNLT--------EIPNGLYPLF---------------------P---FLIAGLHYTNTQVCT
POxa2       : ---------------------------------------------------------------CLDNHPGFDCG-GALWFQNLT--------ELPHGVLPIF---------------------P---FLIAGLHGVNVHY--
```

```
                *       580         *       600         *       620         *       640         *       660         *       680         *       700         *
Cme_Alb3   : PLMPFPYWLQSQSLK--VLLQGAVLVI--TPGVVNLPSAVLLFWLTNSCIQTALTSAWWRQRWHRWI-QQT-----------------ALQREL------------------------------------------------
Ptr_Alb32  : PPVDEEERETLERSQ--TILKFLPLLI--GFFALQVPAGLTIYWFTSNIFTLTQSLAVRAYFSANPP-QIE-----------------LPDYWD------------------------------------------------
Tps_Alb32  : QPDDDETKKQMESTK--TILKFLPLMI--GFFSLQVPAGLTIYWFTSNLFTVSQSLIIRGYYAA----NPP-----------------EVKLPD------------------------------------------------
Ota_Alb3_1 : PEEDEKTKEMQN-QS--VLLKLLPFFI--GYISLTVPAGLALYWFWNNVFTTGIQVYLRNGGA-------------------------------------------------------------------------------
Olu_Alb3_1 : PEETEKTDEMKN-QS--VLLKLLPLFI--GYISLTVPAGLALYWFWNNVFTTGIQVFLRNGGAK----ATV-----------------E-----------------------------------------------------
Ptr_Alb31  : PKTDDPAQQQSN-----AILKVLPIMI--GWFSLSVPAALSVYWVINNIITTGTSLIIRNSMSVETV---------------------TPSGTA------------------------------------------------
Tps_Alb31  : PKTDDPQQQQAN-----VILKVLPFMI--GWFALNVPAALGVYWVVNNIVTTATTLYVR-----------------------------------------------------------------------------------
Cme_Oxa1   : PPVQDPQQQQAN-----AILRFLPFMV--GWFSLNVPSGLGLYWVTNNIVSTIQTIGIKRYLASKQP---------------------ERVGAP------------------------------------------------
CreAlb3_1  : PPIDPKDENANT-QR--ALLVFLPLMV--GWFSLNVPAGLSLYYLANTVLSSAIQIYLKKLGGANVV-MNE-----------------LGPVTK------------------------------------------------
CreAlb3_2  : SQNNQDPSQQQ--AQ--AILKFLPLMI--GWFSLNVPSGLTLYWFVNNLLSTGQQLYLK---ATVKV-NIP-----------------EAIKAP------------------------------------------------
OtaAlb3_2  : PQPKTDDPAQQQ-SQ--AILKFLPFMI--GFFSLNVPAGLTLYWFFNNIITTAQTVVLR--KITKPI-EVP-----------------TGAGAG------------------------------------------------
OluAlb3_2  : PQPKTDDPAQQQ-SQ--AILKFLPLMI--GFFSLNVPAGLTLYWFANNIITTAQTLILRK----------------------------TTTAPE------------------------------------------------
OsaAlb3_a  : PPQTDDPSQKNTL----LVLKFLPFMI--GWFSLSVPSGLSIYWFTNNILSTAQQVWLRKLGGAKPV-VNQ-----------------GGSGII------------------------------------------------
AthAlb3_a  : PPQTDDPAQKNTL----LVFKFLPLMI--GYFALSVPSGLSIYWLTNNVLSTAQQVYLRKLGGAKPN-MDE-----------------NASKII------------------------------------------------
PAlb3_a1   : PPQTDDPTQKNTL----LVFKFLPIMI--GYFSLSVPSGLSIYWFTNNVLSTAQQVWLRKLGGAKPV-VNE-----------------NASGII------------------------------------------------
PAlb3_a2   : PPQTDDPTQKNTL----LVFKFLPLMI--GYFSLSVPSGLSIYWFTNNVLSTAQQVWLRQLGGAKPV-VNE-----------------NASGII------------------------------------------------
OsaAlb3_b  : PPQNNDPSQQGAQ----AVVKFLPLLI--GYFALSVPSGLSLYWLTNNILSTAQQVWLQKLGGAKNP-VKE-----------------YIDKLA------------------------------------------------
AthAlb3_b  : SSQSNDPAMKSSQ----AVTKLLPLMI--GYFALSVPSGLSLYWLTNNILSTAQQVWLQKYGGAKNPVEKF-----------------TNLVTK------------------------------------------------
PAlb3_b2   : SSQSDDPNVKNSQ----AITKFLPLMI--GYFSLSVPSGLSLYWLTNNILSTAQQVWLQKSGGAKNP-MMK-----------------SSDDIV------------------------------------------------
PAlb3_b1   : SSQSDDPNVKNSQ----AIMKFLPLMI--GYFSLSVPSGLSLYWLTNNILSTTQQVWLQKLGGAK-----------------------------------------------------------------------------
Syn_YidC   : AGQEDPSQS--------SMARITPVLFSAMFLFFPLPAGVLLYILVSNIFQTVQTFLLSREPLPENLQQLV-----------------EEERRR------------------------------------------------
Pma1_YidC  : LSGRGPANPQQS-----TANKITPVMITGMFLFFPLPAGVLLYMVIANIFQAGQTFLLSREALPENLQKIL-----------------NDQLSK------------------------------------------------
Pma2_YidC  : LSGRGPANPQQS-----TANKITPVMITGMFLFFPLPAGVLLYMVIANIFQAGQTFLLSREALPENLQKIL-----------------NEQQSK------------------------------------------------
Tps_Oxa2   : GKKALSQSRMALFLK--DAFQSLAVFM--PCFMAQQPSGVQIYLTTSMLFSLLQSMAMRDDAVREYI-GLP-----------------ALNAKP------------------------------------------------
Ptr_Oxa2   : GRRSLSKADTGVLLK--DVFQSLAVFM--PCFTSQMPAGVQIYLVTSFLFTMGQSAALRTEAFRAAV-GLP-----------------SLATAP------------------------------------------------
Esi0025_01 : DMPAAHGPDKPN-FNPKVMFRVMSVVF--VPVAFSMPAGVLVYWTTTNVFGMLQRGLFEMRPVQQAL-GWPLPEDMPAP---------AAPADK------------------------------------------------
Mbr_Oxa2   : IKRPVSPESREDRLG--TALRYLAIAM--IPVAAAVPSGLSLYWASSSVLTLTQNLMLRHPSVRQRV-GLP-----------------AVPSEL------------------------------------------------
Ptr_Oxa1   : EQMVAQNAQSGHLMV--NFFRVMSIGM--VPVCVNFEAAMLCYWTSNNFMTLTQTAILKAPAARSYF-GIW-----------------DAPKPV------------------------------------------------
Tps_Oxa1   : DQMMSSDPVRGRNMV--NAMRALAIVM--VPLTAYFNSAVLCYWVTNNSFTMGQSLFFKLEPVKKMF-GIW-----------------DPPKPV------------------------------------------------
Yli_Oxa1   : ETGTNNLSPGMK-----KILQWAPWIS--VPFLMKMPAALLLHFFVNGILMLIQGVALRNPFFRKKL-GIH-----------------EIVPLP------------------------------------------------
Cal_Oxa1   : ETGQHAMAAGMK-----KVMTVVPIAS--IFITKGFASAIILYFAVNSIFSLIQSSLFKSSWFRKIA-GMP-----------------PKLSLA------------------------------------------------
Sce_Oxa1   : ETGAQQFSSPMK-----RLFTILPIIS--IPATMNLSSAVVLYFAFNGAFSVLQTMILRNKWVRSKL-KIT-----------------EVAKPR------------------------------------------------
Mbr_Oxa1   : EGVKQNNVMMK------NVFRGLSIVL--LPVTINLPTAIFVYWCTANMFSLSQMLMLKIPGLKKSL-GIP-----------------EQIQH-------------------------------------------------
Hsa_Oxa1   : ETGVQSSDLQW--MR--NVIRMMPLIT--LPITMHFPTAVFMYWLSSNLFSLVQVSCLRIPAVRTVL-KIP-----------------QRVVHD------------------------------------------------
Cfa_Oxa1   : ETGMQSSDLQW--MR--NLIRVMPLAV--LPITIHFPTAVFMYWLSSNMFSLGQVACLRIPAVRTIL-KIP-----------------QRVVHD------------------------------------------------
Mmu_Oxa1   : ETGVQSNDLQF--MR--NIIRVMPLVV--LPVTIHFPSAVFMYWLSSNVFSLCQVACLRIPAVRTVL-KIP-----------------QRVVHD------------------------------------------------
Aga_Oxa1   : DSARMSAANMQT-AK--YILRALPLFI--FPFTINFPGAILCYWACSNFFSLVQVGFLRIPKVRDFF-KID-----------------RIVTHK------------------------------------------------
Dme_Oxa1   : DSARLSAANMNT-MK--YVLRALPIVI--FPFTMNFPAAILTYWACSNFISLGQVAVLRIPSVREYF-KIE-----------------KMLTHA------------------------------------------------
Pin_Oxa1   : DAMGGDMQRNLK-----FGMRCFALMM--VPLTMNFQSGIFVYWVTSNMFTLTQTALMRLNVVKRAL-NIP-----------------VTEVQR------------------------------------------------
Pra_Oxa1   : EAMGDEMKGKMK-----FGMRCFALMM--IPLTMNFQSGIFVYWVSSNMYTLVQTALMRLNFVKRAL-KIP-----------------VSEVQR------------------------------------------------
Pso_Oxa1   : EAMTGEMKDKMK-----FGMRCFALMM--VPLTMNFQSGIFVYWVTSNMYTLTQTALLRVNFIKRAL-KIP-----------------VTEVQR------------------------------------------------
Ota_Oxa1   : VEGMQTSQSAQ--MK--WFLRALAVAM--VPLTASFPQGVFVYWITSNMFSGVQTSITRTKGFKAAM-GIP-----------------DVSAVA------------------------------------------------
Olu_Oxa1   : VEGMQTSQSAQ--MK--WFLRALAVAM--VPLTASFPQGVFVYWITSNIFSGFQTSITRTKAFKSTM-GIP-----------------DVSAVA------------------------------------------------
Osa_Oxa1   : QEGMEGNPMARK-MK--NFSRGMAVLT--VPFTMSFAKGIFCYWITSNLFTLTYGFVIRRPAVRKFC-NLP-----------------ALEAQS------------------------------------------------
AthOxa12   : QEGLEGNPVAGT-MK--KFSRIIAFLS--IPVLIGIEKALFCYWLTSNLFTLVYGLTLRRPDVRKLL-NLP-----------------DVVNSS------------------------------------------------
AthOxa11   : QEGMEGNPMAGT-VK--TVCRVFALLT--VPMTMSFPQAIFCYWITSNLFSLMYGLVIKRPQVKKML-RIP-----------------DLPPPP------------------------------------------------
POxa11     : QEGMEGNPAAGT-MK--NVSRALAAAS--VPLTMSFPKAIFCYWITSNLFSLTYGLVLKAPGVKEFL-GVP-----------------KVPVAP------------------------------------------------
POxa12     : QEGLEGNPIAAT-MK--KVSRVFAVAS--VPLTMGFPNAIFCYWVTSNLFSLFYGLTLKAPGVKKFL-GLP-----------------EIPVAP------------------------------------------------
Cme_Oxa2   : ETGGAQLPPFAL-----NLMRIFALIL--LPLTAQFPAILFCYWLPNNLFSMLQAFALRHQRVRRWL-GCP-----------------PLGARS------------------------------------------------
Esi0028_00 : EGGALAGSSVK--MK--AGMRGMALLV--TPLTMYVSTGVFVYWTTSNFYSILQTLAFKSSGIKKFF-DFP-----------------DLPPNKLKSNTAEKEIGWLDVLGGDHPIKEYRKMQDQREVQAWFVEGEGGWTRK
Mmu_Oxa2   : LQKIGTSRFQMH-VT--NFVRAVSVLM--IPVAATVPSALVLYWLCSSLMGLAQNLLLRSPGFRQLC-RIP-----------------PSKSDS------------------------------------------------
Hsa_Oxa2   : LQKIGMSRFQTY-IT--YFVRAMSVLM--IPIAATVPSSIVLYWLCSSFVGLSQNLLLRSPGFRQLC-RIP-----------------STKSDS------------------------------------------------
Cfa_Oxa2   : LQKIGMSRFQTY-IT--YFVRAVSVLM--IPVAATVPSSIVLYWLCSSLMGLSQNLLLRSPRFRQLC-RIP-----------------LTKSDS------------------------------------------------
Aga_Oxa2   : ASRTKLPSKLQTIFT--NLFRGLSILM--VPIAASVPSCLCLYWVTSSAYGLGQNLLLLSPRVRRTV-GIP-----------------AVPSEL------------------------------------------------
Dme_Oxa2   : MSRTRPSTRLQNIAN--NVFRGLSVVM--VPVACTVPSALCVYWVASSSFGLAQNLLILSPEVRRSV-GIP-----------------KTQTEL------------------------------------------------
Yli_Oxa2   : KAQAQAGPKLPKMMA--NFARVGAIAL--FSIAAQTPTAVCLYWISSSGFSLIQNVLLNKYMPLR---EEP-----------------PMFTAA------------------------------------------------
Cal_Oxa2   : KTLELRPTLTDA-FG--NLTKMSIVFM--MAISLHAPAALTIYWISSQLYSLLQNVMMDL--------MLP---------------------IS------------------------------------------------
Sce_Oxa2   : RLMFSNSTRVQEAMSILNVSRLGCVVM--LAMSSQAPFLLSLYWISSQLFSLVQNIILNWIYPYQR----------------------------------------------------------------------------
Olu_Oxa2   : ASAAAQSRGMTI-VK--LVLEWMTLPM--LVIGMQLPQAVHCYWLTSSAWALAQNRALSTTFAREAL-GIN-----------------ALAKVT------------------------------------------------
Ota_Oxa2   : AAAAQQSRGMTI-VK--LCLEWMTLPM--LLIGLQLPQAVHCYWITSSAYAYAQNRALSTAYAREAL-GLN-----------------ELAKTT------------------------------------------------
Esi0170_00 : MKGATGWMKFFQ-----DGMQTFIILM--LPWVSTFPQGVFMYWIPSAVFQMGQTYTMKNNKVRELL-GLKPLGLPPREAAGATPSLAPAPAPA------------------------------------------------
Pra_Oxa2   : RTKSGLWTDLL------QIGQYGSLLA--IPWLATLPQGIFFYWLGASWSSMAQIAAMDNNNFRRRI-GLK-----------------PRITQT------------------------------------------------
Pin_Oxa2   : RNKSGFWTTLL------QGGQYITIFA--VPVLVNLPQGVFFYWLGASCSSMAQTIAMNNNNFRRRI-GLK-----------------PRITET------------------------------------------------
Pso_Oxa2   : RKKSSFWTQVL------QFGQYGTIFA--IPLMANLPQGVFFYWLGASWSSMAQTIAMDNNNFRRRI-GLK-----------------PRIAHT------------------------------------------------
Osa_Oxa2   : -----------------VYLEILTIPL--FLIGYVIPQGSLVYWTTNGLITVAQQLSLKNDAVKKVL-GLP-----------------DTRAHQ------------------------------------------------
AthOxa21   : DTSTITGLLMRY-YK--LYLEILSVPL--FFVGYAIPQGSLVYWVTNSSVNIFQQLSLKHPTVGAKL-GLL-----------------SQGATP------------------------------------------------
AthOxa22   : -----------------------------VWFFVGS----------------------------------------------------------------------------------------------------------
POxa2      : -------------YR--KYLNFMMLPL--FFIGYCIPQGSLVYWVTNSSLTAIQQVSLKLPVVRAKL-GLL-----------------DKDFPK------------------------------------------------
```

```
                    720         *       740         *       760         *       780
Cme_Alb3   : -------DKFDQALDQASKH-------------------------------------------------------
Ptr_Alb32  : -------TALKNQAVSA----------------------------------------------------------
Tps_Alb32  : -------YWGASKT-------------------------------------------------------------
Ota_Alb3_1 : ---------------------------------------------------------------------------
Olu_Alb3_1 : ---------------------------------------------------------------------------
Ptr_Alb31  : -------TASAPPRAN-----------------------------------------------------------
Tps_Alb31  : ---------------------------------------------------------------------------
Cme_Oxa1   : -------RTLDGAVDDR----------------------------------------------------------
CreAlb3_1  : -------PGSGRRNGAAGGA-------------------------------------------------------
CreAlb3_2  : -------ATAGSSTPIKKA--------------------------------------------------------
OtaAlb3_2  : -------SSPASAEPISSG--------------------------------------------------------
OluAlb3_2  : -------PPVAKSGAAG----------------------------------------------------------
OsaAlb3_a  : -------TAGRAKRTAS----------------------------------------------------------
AthAlb3_a  : -------SAGRAKRSAE----------------------------------------------------------
PAlb3_a1   : -------TAGRAKRSSG----------------------------------------------------------
PAlb3_a2   : -------TAGRAKRSAG----------------------------------------------------------
OsaAlb3_b  : -------KEESTNLGPCNK--------------------------------------------------------
AthAlb3_b  : -------EDKTQQISSTTR--------------------------------------------------------
PAlb3_b2   : ---------------------------------------------------------------------------
PAlb3_b1   : ---------------------------------------------------------------------------
Syn_YidC   : -------AAQASTGEAKEVFEP-----------------------------------------------------
Pma1_YidC  : -------PALATEAIGGSDRSK-----------------------------------------------------
Pma2_YidC  : -------PALATEAIGGSDRK------------------------------------------------------
Tps_Oxa2   : -------VDMGDSVQEFPKK-------------------------------------------------------
Ptr_Oxa2   : -------PPEAKYQFIAKKG-------------------------------------------------------
Esi0025_01 : -------KEPAEADFASKEDLERWGGGGEGASGEASRAAAEAAERAREAVAKASKVRHEELKRQHGERSGEDGPK
Mbr_Oxa2   : -------PRPLTSLRRHGNP-------------------------------------------------------
Ptr_Oxa1   : -------PGQEPETKAA----------------------------------------------------------
Tps_Oxa1   : -------PGQETGLEK-----------------------------------------------------------
Yli_Oxa1   : -------AAAPGAGVRK----------------------------------------------------------
Cal_Oxa1   : ---EMQANNPKANQSIQRH--------------------------------------------------------
Sce_Oxa1   : -------TPIAGATENM----------------------------------------------------------
Mbr_Oxa1   : ---------------------------------------------------------------------------
Hsa_Oxa1   : -------LDKLPPGFLEPLG-------------------------------------------------------
Cfa_Oxa1   : -------SNKLLPGFLKPLG-------------------------------------------------------
Mmu_Oxa1   : -------PDKLPPGFLKPLG-------------------------------------------------------
Aga_Oxa1   : -------PETLPKGFTD----------------------------------------------------------
Dme_Oxa1   : -------PSALPPGFVGEQG-------------------------------------------------------
Pin_Oxa1   : -------LEASTITTSP----------------------------------------------------------
Pra_Oxa1   : -------LEASSITQTP----------------------------------------------------------
Pso_Oxa1   : -------LEAASITTSP----------------------------------------------------------
Ota_Oxa1   : -------DAPADAALSR----------------------------------------------------------
Olu_Oxa1   : -------EAPAEAALGR----------------------------------------------------------
Osa_Oxa1   : -------ASAKKQMFNLMRP-------------------------------------------------------
AthOxa12   : ------TRQPSPPLKKK----------------------------------------------------------
AthOxa11   : -------PGQQPSFDLSKK--------------------------------------------------------
POxa11     : -------PTTAAKSFDLK---------------------------------------------------------
POxa12     : -------ASTTPSFDLNKR--------------------------------------------------------
Cme_Oxa2   : -------AAATAGHTNR----------------------------------------------------------
Esi0028_00 : AHNAEQGEGGNVAREGAPFHAPPSMAGPVSGKAKTPSFASSAAAGTPKVVLRSVRPKPAGDRGKTAARGPRKNRG
Mmu_Oxa2   : -------ETPYRDLSAA----------------------------------------------------------
Hsa_Oxa2   : -------ETPYKDIFAA----------------------------------------------------------
Cfa_Oxa2   : -------DSPYKDLFAA----------------------------------------------------------
Aga_Oxa2   : -------THPYQHIRS-----------------------------------------------------------
Dme_Oxa2   : -------SEPYDLLWLA----------------------------------------------------------
Yli_Oxa2   : -------NEALPKEH------------------------------------------------------------
Cal_Oxa2   : -------FTPKKRININ----------------------------------------------------------
Sce_Oxa2   : ---------------------------------------------------------------------------
Olu_Oxa2   : -------REIAASLGGE----------------------------------------------------------
Ota_Oxa2   : -------REIVRELHSD----------------------------------------------------------
Esi0170_00 : -------PIPAPKAIGG----------------------------------------------------------
Pra_Oxa2   : -------ESPAAAAAEM----------------------------------------------------------
Pin_Oxa2   : -------KPPAAAVAEM----------------------------------------------------------
Pso_Oxa2   : -------ESPAAAAAEM----------------------------------------------------------
Osa_Oxa2   : -------KFPRVGMMQEKEH-------------------------------------------------------
AthOxa21   : -------GGHSMEISESSHF-------------------------------------------------------
AthOxa22   : ---------------------------------------------------------------------------
POxa2      : -------APALSALSSS----------------------------------------------------------
```
