# Supplementary material for: In Silico Survey of the Mitochondrial Protein Uptake and Maturation Systems in the Brown Alga Ectocarpus siliculosus
Source: PLoS One. 2011 May 18;6(5):e19540. doi: 10.1371/journal.pone.0019540 (PMC3097184; doi:10.1371/journal.pone.0019540)
Supplement: Figure S3 — Alignment of Tom70 proteins showing the presence of a single transmembrane region and multi-TPR domains. TPR regions have been superimposed on the Saccharomyces cerevisiae model and these domains were validated by bioinformatic analysis using the HHrep ID and TPRpred tools. Transmembrane regions were predicted by DAS and TMpred. Residues shaded in green (opisthokonts) or blue (stramenopiles) represent amino acids implicated in substrate binding and red-coloured (opisthokonts) or yellow-coloured (stramenopiles) positions correspond to residues involved in dimerisation according to [34], [49]. Esi0007_0019 and Esi0232_0002 are the Ectocarpus siliculosus proteins and the sequences from other organisms correspond to Blastocystis sp. (Blasp), Phaeodactylum tricornutum (Phatr), Phytophthora infestans (Phyin), Pichia pastoris (Picpa), Saccharomyces cerevisiae (Sacce), Naumovia castellii (Nauca), Neurospora crassa (Neucr), Aspergillus nidulans (Aspni), Trichoplax adhaerens (Triad), Nematostella vectensis (Nemve), Danio rerio (Danre), Homo sapiens (Homsa) and Xenopus laevis (Xenla). (PDF) [file pone.0019540.s003.pdf]

(a)

```
                            TRANSMEMBRANE REGION
BlaspTom70      1 ----------MSSNNQAK------VVTAVAAVGVVAAVAVYFF----------KSRKSQKKSEAPKEEEKPKQEFPGKVEGKVEFPGKVEGDKKEFPGKVEGKVEFPGKVEGKKEFPGKVE
Esi0007_0019  131 ---------------------TADLATIGSSLL--GAGAVYTPGPQ-------QAGRRGGRGGGAPLC---DGTPRQPSRAGHAGRCSSSPAGDGDSSCAGENNKCSTAGRAEGGGGGGGG
Esi0232_0002   32 GPRREGRGRWRMDQSKLAVLGTVGLVATAAVCCAIVFSGRFSF----------AGSSTGKPFASSPGTTADAQGVAASNKVIDDEDDKDKLTGGVSTEKGTGKDSGGGDGSRAAPGSGG-
PhatrTom70      1 --------MALGTRSN-Q-----TLVVGLAAAATASLLLYYVV----------QARTRPTS----------------DSSPRDTTDRKSRS---------VEFTTPSPKKTSGPLTKAVG-
PhyinTom70      1 ----------MEQHAILQ------VAVLASLATISAFVAFSLY----------HKRQQQ-------------------------------------------FEIAKQPEVVVSTPEAQP-
PicpaTom70      4 QSFWNSTVKFVSSHKA------VITLTTIAGA---STLAYLYY----------ATNATSKP--------------------------------DSSATGSSKNKKKKSKKQTAPETSNAD-
SacceTom70      1 ------MKSFITRNKT------AILATVAATGT--AIGAYYYY----------NQLQQQQ--------------------------RGKKNTINKDEKKDTKDSQKETEGAKKSTAPSNP-
NaucaTom70      8 ------VGGFLTRNRT------AIVATVAAGSA--AIGAFYYY----------HHLQQTHT----------------SLEGEEDGNSTGRNPVNDEAHLSKNQKKKQRKKHKKKATPDQTT
NeucrTom70     24 SSIWDRVSNWVSEHKA------VVYTIAGVSVVITTAGVVYYL----------RKGSEQKE----------------SGPKLSKKERRKRK---QAEKGEAEKASTSKTEEAAPTQPKAA-
AspniTom70     27 ASVWDRISKWVSENKA------LVYTVAGVAVVVTSAGVVYYL----------SDSSRSGK--------------PSPTTPAPTEKKKKKSGSQRRKEKKAEEERKSKAASVQDEKTEKK-
TriadTom70     13 GGSLANLSKW----Q-------VALLLGVGV---GVGAAYYYY-------------------------------------------------------------RRGQSSIAARQPQQAD-
NemveTom70      8 ----SGLEKW----Q-------IALLIGAPVAVVCVAGAVWYW----------RNSQ------------------------------------------------EGEEEGEREDEEKGN-
DanreTom70     13 ----SGLPRW----Q-------LALLVGTPIVL--GVGAVYLW----------NRSRAKEK--------------------------------------------QGKRNGERKTPEGSA-
HomsaTom70     30 GPGTGGLPRW----Q-------LALAVGAPLLL--GAGAIYLW----------SRQQRRRE------------------------------------ARGRGDASGLKRNSERKTPEGRA-
XenlaTom70     13 ----PGIPHW----K-------LALAVGAPLLV--GAGAVYLW----------RRHK------------------------------------------------AAKSDKQRRTPEGSA-
```

```
                                                            TPR1                                                   TPR2
BlaspTom70    329 APAKKPAE FVTFYNEAKRQF-----------------------------------NNQEYEAALSNFNKAIDAIPKN NAQ-------------LKT LIYSRGSCLKKLNRQEEA
Esi0007_0019  353 ACSES--- PTAADGDGDTSLCERPAACLEFLLTSQAVRVRADESMERGLDKEAAASLKEANILMEAFLEEGQRLPRP -----------SRH--LAE LRAGLAKCEMRGGDLEAA
Esi0232_0002  164 DGEALIVR FNRANSKAKKLF-----------------------------------TGQRYALAAEQYGIALELCDEL PNHD----------NKRTA LHNNRGAAYEKDGQYALA
PhatrTom70     94 EKEVN-AK IELLDKKGKDLF-----------------------------------KNKQYLDAAETFTEALTLIETS DTSGSVQNTSSSLHRQLIT LLNNRSAMYEKGNQPELA
PhyinTom70     74 KPTPAANI GEQEATLATKKY-----------------------------------KAGDYDGAVELYSVAISKCEQQ QPLDA---------RNLKV MYSNRAAAYEKLEDYENV
PicpaTom70     97 EKNTW--- AQSLKETGNRAF-----------------------------------KSDDYETALEYYNLALLCKKDP ------------------A FYSNSSACWACLGDNQKV
SacceTom70     93 EKDKY--- ALALKDKGNQFF-----------------------------------RNKKYDDAIKYYNWALELKEDP ------------------V FYSNLSACYVSVGDLKKV
NaucaTom70    117 EQKKI--- SMQLKDKGNKYF-----------------------------------KAKDYTNAIKYYNLALDLNKDP ------------------I FYSNISACYVSLGQLDKV
NeucrTom70    131 ERKAY--- AAKLKELGNKAY-----------------------------------GSKDFNKAIDLYSKAIICKPDP ------------------V YYSNRAACHNALAQWEQV
AspniTom70    134 TRKAY--- AAKLKAAGNKAY-----------------------------------GAKDYPRAIELYGKAILCKPDP ------------------V FYSNRAACYNVQSEWEKV
TriadTom70     79 ER------ ASAVKGKGNKFF-----------------------------------KGGKYEQAIRCYTEAIELCPSS ESDI------------RSV LYQNRAAAYEQLKEFDKV
NemveTom70     77 EQ------ AQVAKLKGNKYF-----------------------------------KGCKYEQAIKCYTEAIELCPPE NKQD------------LST FYQNRAAAYEQMNQFENV
DanreTom70     82 DR------ AQSAKNKGNKYF-----------------------------------KAGKYDHAIKCYTEAIGLCPKE KKGD------------LST FYQNRAAAYEQQMKWTEV
HomsaTom70    111 DR------ AQAAKNKGNKYF-----------------------------------KAGKYEQAIQCYTEAISLCPTE KNVD------------LST FYQNRAAAFEQLQKWKEV
XenlaTom70     79 EK------ AQAAKNKGNKYF-----------------------------------KASKYEQAIQCYTEAISLCPAH NKSD------------LST FYQNRAAAHEQSQNWKEV

                                          TPR3
BlaspTom70        IADYTQCITLDDKY S RAYKARAGVYKSLDRLEEAIEDFSYCYLLDTV SSG-----------DMQMTPEGMDEVIQALAKRDAKAELARRH--------------AD-KEYKRHLPSKQ-
Esi0007_0019      IAHAEAALAEHPAC G EAFLIRGQCRRELGDNGGALRDLVNAFVLQGN AL---------NNAGDGSEAQAIEDVSRESCRARAGEEFSQRA--------------APNA-----LPADW-
Esi0232_0002      LADCSMCLSREVGH K FARVRKSRVLEAMGKHEEALSEVCAHLLLERD RVQAKAALNPSEPLTPPAPPANLEGLLQKVASKRADAILLEREQTAEKQEAAAAGGAGTGTE-K-LKPLVKQ
PhatrTom70        LEDCTQILDQDVHH A KARTRKLRVLESLGRWHDALVEVCAVQLLFMR KHRDSMRLGLKVP-PPPVPESKMQEILTNVVPLEMEPYIQALN--------------EK---TTRPLPSGY-
PhyinTom70        VADCTKALKLDKRH P KSYLRRAKARANAGDLRGSLVDYVCLLVISEE --------------KQEQVDENLAQEISRIHSDITAKEIEDAQ--------------QNKQNPTRYLPDQF-
PicpaTom70        IEFSTKALELKPDY A KCLMRRAAAYEKIEEYEKALYDLTVLTIYEST I-------------ADKSIQSMLERILTKQANYVMEKQLKDFV--------------PQ-------LPSAS-
SacceTom70        VEMSTKALELKPDY S KVLLRRASANEGLGKFADAMFDLSVLSLNGDF --------------NDASIEPMLERNLNKQAMSKLKEKFGDIDTATATPTELSTQPAKERKDKQENLPSVT-
NaucaTom70        IESSTKALELNPNY S KALLRRASANESLENYSDALFDLSVLSLNGDY --------------SGASIEPILERNLNKQAIKVLNERMNKMT--------------GS-NKEQQLLPSTT-
NeucrTom70        VADTTAALKLDPHY V KALNRRANAYDQLSRYSDALLDFTASCIIDGF --------------RNEQSAQAVERLLKKFAENKAKEILETKP--------------PK-------LPSST-
AspniTom70        VEDTSAALAMDSEY V KALNRRAIAYEHLEKYSEALLDFTASCIIDGF --------------SNEVSRVALERLLKKVAERKGKEILEAKG--------------KK-------LPSPT-
TriadTom70        VEDCNSALELNKHY V KAINRRSRAYEELKEYRKCLEDLTAQCILESF --------------QNAATIMSADRVLKIVGKIEAKQRFAERQ--------------YF-------LPSTA-
NemveTom70        VEEATKALELNSKY T KALMRRARALEKLERKQECLQDLTAVCILEGF --------------SNPSWMMHADRVLKDIGRQKAKEHFKNRK--------------PT-------IPSPT-
DanreTom70        IQDCSQAVELNPRY V KALFRRAKALEKLDNKKECLEDVTAVCILEVF --------------QNQQSMLLADKVLKQLGKEKAKEKYKNRE--------------PL-------MPSPQ-
HomsaTom70        AQDCTKAVELNPKY V KALFRRAKAHEKLDNKKECLEDVTAVCILEGF --------------QNQQSMLLADKVLKLLGKEKAKEKYKNRE--------------PL-------MPSPQ-
XenlaTom70        VEDCTKAVELNPRY V KALFRRAKAHEKLDNKKECLEDVTAVCILEGF --------------QNQQSMFLADKVLKLLGKEKAKDKYKNRE--------------PL-------MPSPQ—

                                                                                         TPR4
BlaspTom70        FVAFYFSTI------------------ASEKAAEYNPSKFTEQYLAELIA------ APQGDAT---VGDYLLMRGGLRKA-EQRYEEAMADFLEA A-------------KEESHCHAQ---- ADA
Esi0007_0019      VVRSFLTSYDTKGLYEE----------HDAVFRLHG----------EDPVAAAAAA EGDEGGQAAAVTEFWQGLSLVREG---KYAESIAKFSSS VSAFSSSAPVAVLGEGTGAGDTTR-IQ SLA
Esi0232_0002      VVMELLRSFGSFAQLERRYKGMEETAITRELKDAEKAGKEGDGSASATTTSASRVS SLLDRGL---LRMVKRNYDGAREDIFEAAELLSTLTEAD P-------------SEAGADEVPPHVK ASV
PhatrTom70        TILQLLRSFTSYNSWMA----------QAAKDGNVANIDKELVEGVDAASKAQRVH VLLKRGR---RHVYDRAFENASDDFEQAYALAETNEVQL L-------------LEGDDY------- ARV
PhyinTom70        FVTSYYSSFH-----------------PSDDENDVVAEKSSEEYTTELHALEAGDD TRQQRGW---LLTKRGLALKKEKDYDMAAKDLDAACKLV E-------------PEDEAY------- YTA
PicpaTom70        SIASLLGSFT-----------------EDSVSDVFGTEIPEDGSG----------D KFLYEAL---QDFKTATADSYEHA-DVALNQAVSKYADV T-------------VSSDETTKRRA-- AMA
SacceTom70        SMASFFGIFK-----------------PELTFANYD--------------ESHEAD KELMNGL---SNLYKRSPESYDKA-DESFTKAARLFEEQ L-------------DKNNEDEKLKEKL AIS
NaucaTom70        SLVSFFGIFD-----------------PELTFQNYN--------------ENDQAD VELLTGL---TNLFKRTDQGYLDA-DKNFVKASSLFMEE L-------------AKNPKDKHLLEKT AIA
NeucrTom70        FVGNYLQSFR-----------------SKPRPEGLE----------------DSVE LSEETGL---GQLQLGLKHLESKT-GTGYEEGSAAFKKA L-------------DLGELGPHE---- ALA
AspniTom70        FVSNYLQSFR-----------------PKSLPEGLD----------------ESAD IPEESGK---GQLRKGLLAMAKKT-GDGYEEAAAAFVKA L-------------ELGDLGEFE---- GLA
TriadTom70        FIQTYLESFA-----------------NGKKNNDIILFNF---------------- TTAFEED---DYF---LIARYNLS-QCNYENIIDYCTKE I-------------D-KDFSPYR---- AEA
NemveTom70        YIKAYLESFS-----------------QDEIFSDDLQGQD---------------- --DVPND---SPF---LAALEEIK-EKRFDKVIDLCDDE I-------------N-RGPSPCY---- ARA
DanreTom70        FIKSYFSSFT-----------------DDIISQPLQKGEKKDEDKDK---EGE--- ASEVKGS---SGY---LKAKQYME-EENYDKIISECTKE I-------------ESGGRYS------ AEA
HomsaTom70        FIKSYFSSFT-----------------DDIISQPMLKGEKSDEDKDK---EGE--- ALEVKEN---SGY---LKAKQYME-EENYDKIISECSKE I-------------DAEGKYM------ AEA
XenlaTom70        FIKSYFSSFT-----------------DDIISQPLQKGEKSDEDKDK---EGE--- AAVVKEK---SGY---LKAKKYME-EENYDKIISECTKE V-------------ESEGKYM------ AEA
```

```
                  TPR5                                   TPR6                               TPR7
BlaspTom70     CLEAATLLSLSGDTIEALKYYERVYADKAQ------RSV  NLLVKYASCLVEQEDERYR-AIFDEACASFPGE  CDGFFHRGQVMYI---------ENKTEEAIRDLTKVTELNKNH A
Esi0007_0019   LEYCGSFLYLMGDMNTALEHLRLA-GEVDE------TNA  KSWVKRGSVLSDLGRREEAFECFDAAAAIAPRD  SDLFLHRGQGHLL---------ANDFRKATADLRRSVELCPTM P
Esi0232_0002   WEWQGTFLQLSGKLDEAMEAYRRC-GEEMEAEGEE-YPA  DVLIKMAWVCMDKEDMDAAKDLFARAGEAHPEY  GSSFAHRARLDSE---------KDGAEQVRSFLRKAIELNSED A
PhatrTom70     LEWTGMVKHWRYKLDEASACYEKC-ADLEP------TNA  LVLVKNAGVKMDGSHQDEAMKLFDTALGLDPKN  ADALLHRANLRLL---------QTKPDEAKEDLEACIAVRPDH I
PhyinTom70     QIENGTYHHLRGEFELARKSFEKS-LNAKP------RSI  FAKIRMGGLCFDQKDLKKALEWFDKALAEKSEC  STAYFHRGQLHSIDVSLDGTSNESSMGAALNDLEKCISIAPDF A
PicpaTom70     YEYLGQFYFLKNVTAVAQEHLKRA-IELSP-------RP  RSHVILALTYIDKDLLNEAEVEFQKAMAINAND  PDIYYQRGQLSYL---------RGDLANASENFETCKRLNPKN V
SacceTom70     LEHTGIFKFLKNDPLGAHEDIKKA-IELFP-------RV  NSYIYMALIMADRNDSTEYYNHFDKALKLDSNN  SSVYYHRGQMNFI---------LQNYDQAGKDFDKAKELDPEN I
NaucaTom70     LEYNGIFRFLKNDLLGAQTDIEKA-IGFFP-------RV  NSYLYLALISADKSTSQEYSKYFDKALELNPDS  LSVYYHRGQLYFI---------TQDYPKAKVEFEKAKELDETN I
NeucrTom70     YNLRGTFHCLMGKHEEALADLSKS-IELDP------AMT  QSYIKRASMNLELGHPDKAEEDFNKAIEQNAED  PDIYYHRAQLHFI---------KGEFAEAAKDYQKSIDLDSDF I
AspniTom70     LNQRATFTYLQGNAHNALADLNKS-VELDP------SLV  QSYIKRASLHLELGNKDAAQDDFELAITHNKDD  PDIYYHRAQLHFI---------LGEFAEAAKDYQKSIDLDRTF I
TriadTom70     YALRGTLRLLMTDSDSALEDFDKV-LEFDD--GSPRVKV  NALIKRASYKLQQEKTNEAIADFSSALELDPEN  CDIYYHRGQAYFL---------LERLSDAMLDFQKSYELNENF S
NemveTom70     MALRGTMHTLMSQVKEAIDDLTQV-IDMDDDKASTKLKI  NCLIKRGSLHIQETREAEADQDFKKAILLDPNN  SDIYHHRAQIHFL---------TEKIAEAKEDFEKSIKLNPDF I
DanreTom70     LLLRATFYLLIGNATAAQPDLDRV-INMND--ASVKLRA  NALIKRGSMYMQQQQPQLSTQDFNMAAEIDHRN  ADVYHHRGQLKIL---------LDQVEEAVGDFDECIKLRPDS A
HomsaTom70     LLLRATFYLLIGNANAAKPDLDKV-ISLKE--ANVKLRA  NALIKRGSMYMQQQQPLLSTQDFNMAADIDPQN  ADVYHHRGQLKIL---------LDQVEEAVADFDECIRLRPES A
XenlaTom70     LLLRATFYLLIGNAAAAKPDLDQV-ISMEE--CNVKLRA  NALIKRGSMYMQQQQPVLSTQDFNMAADIDPQN  ADVYHHRGQLKIL---------LDQVEEAVEDFDECIRLRPDS A
```

```
                     TPR8                                  TPR9                                             TPR10
BlaspTom70     MAYVQLAFCYMNQN---------QSQQAMESMKRAVACGKNR  AQVFN----HFGELCMALG-------LFSDADMNFKKAEEADKD-  -----W  AYSYVNEATYYLQTT----------QDYL-KASDLLK
Esi0007_0019   ISRAAWGVALFKLATAAELPSPSSLSKCVQVLEESRELFPEN  PEVLF----FFAEVLISMG-------DFKKGLEFLQTAASLDPEC  ------  PVPYVNAARAYLGMNDTKAARRQVVAVVQ-QGYDFFL
Esi0232_0002   FAWEQLCRIHVQAG---------DIPKATSTIEEGLEFVPNS  DALLT----LKAELKYSMAMKAGDASSCAAILEVFDAAIRANPS-  -----S  PVLYLNKASCLLQMM----------SDVG-GAMELLE
PhatrTom70     MARLRLASILAATN---------EAAKAKKHLDAAEKVEPKS  SEVQS----YRGELHFTQG-------EFDQARAQFEKAIALDPT-  -----N  PTPYVNAAMAILQTPPPPGQM----PDAQ-EVIRLLE
PhyinTom70     MAYIQLGVTHARTG---------NFQGAVEYLTTAARITPDV  PEIYNYLGETYMQMLQAPGS----TVDLKTVEEMFEEAIELDPS-  -----Y  PMAYINQGNLLVQKG----------TEYGHQALALFE
PicpaTom70     YAYIQLACISYREG---------KIEEAVERFVQAKRTFPTS  PEVPN----YYGEILVDKN-------DTEDAIKQFDIAIKLQRSL  STVSIG  ALGILNKATILARR-----------NEFP-QAIELLE
SacceTom70     FPYIQLACLAYREN---------KFDDCETLFSEAKRKFPEA  PEVPN----FFAEILTDKN-------DFDKALKQYDLAIELENKL  DGIYVG  IAPLVGKATLLTRNP--------TVENFI-EATNLLE
NaucaTom70     FPYIQLACLAYREN---------DFDRCQKLFDETRQKFPTY  PEVPT----FYAEILADKG-------DFALAIKQYDIATRLEKAQ  KNIHVG  IAPLVGKATVLARQP--------NLLNLK-EATQLME
NeucrTom70     FSHIQLGVTQYKMG---------SIASSMATFRRCMKNFDQT  PDVYN----YYGELLLDQN-------KFQEAIEKFDTAIALEKET  KPMCMN  VLPLINKALALFQWK----------QDYA-EAEQLCE
AspniTom70     YSHIQLGVTQYKMG---------SVASAMATFRRSVKNFEDV  PDVYN----YYGELLLDQQ------NFSEAIEKFDKAVEMEKQS  KPMSIN  VLPLINKALALFQWK----------HDFA-EAEQLCQ
TriadTom70     QAYVHLGYARYKSAVTQQSPS--LVEKSIKTFEDALEKYPNS  ADAVS----LYAQILQDQQ-------QLQKADELFDKAISIRPN-  -----F  PSYYVHKGVLQVQLK----------QDIE-AGIKLIE
NemveTom70     PARIQLAYCIYKTAVFQQNAV--LARGALELFEEISTNNPDN  PDALS----LHAQVLQDQG-------QFEEACEKFDTAIKLQPE-  -----N  PVHKVYKGLLMVQWK----------QDFG-KSVELVN
DanreTom70     LAQAQKCFALYRQAYTGNNPS--QVQKAMDGFEDVIRRFPKC  AEGYA----LYAQALTDQQ-------QFGKADEMYDKCIELEPD-  -----N  ATTYVHKGLLQLQWK----------QDLE-MGLELIS
HomsaTom70     LAQAQKCFALYRQAYTGNNSS--QIQAAMKGFEEVIKKFPRC  AEGYA----LYAQALTDQQ-------QFGKADEMYDKCIDLEPD-  -----N  ATTYVHKGLLQLQWK----------QDLD-RGLELIS
XenlaTom70     LAQAQKCFALYRKAYTGSNPI--QIKEAMQGFEDVIIKFPKC  AEGYA----LYAQALTDQQ-------QFGKADEMYDKCIDLEPD-  -----N  ATTYVHKGLLQLQWK----------QDLE-KGLDLIS
```

```
                                               TPR11
BlaspTom70     KA-IKIDPSCI  NAYLQLAQLHMLL----------------QEWDDANSILDSALKEAVSY  SDITSVYTSKESSTYQLKTLQKYQTSLKAAK------------------
Esi0007_0019   DG-HAVCPATG  CCASENLSVIGRRMRFILRSMLMPMCVLLGTVKSLFLFHSFVGICSRFS  LAATTWCMVQLFWDIRPIVRGRLCLWSNRLLRF----------------
Esi0232_0002   KG-VSVDPTSV  NALVQLANLKI----MVAREM--------AEAEAATALLDKAVALCTTK  EELMETLSVRVATEGRIKGALLLGRTTLG-------------------
PhatrTom70     EA-IRVDPSFT  LAYTHLGNVKL----GTATEL--------SSAREVVTLYDQALANCRSE  EEIKELCSMRILAVAQVEAASMLKMESFNMQ-----------------
PhyinTom70     KA-VEMCPRSK  FAYCHLAQVYMAM----------------QEYSRAIEQIDKAVAFAFSK  DELNELFAIRVTAETHQQASVLLQ-----------------------
PicpaTom70     EA-IQVDPKSE  LAAVQLAQIKLQL----------------NQAEEAIVLFEKTAKLARSP  EEKQHAISFAEAAKIQARVKKD-PALSKRVEEIIAQAQGHQNEATAASA
SacceTom70     KA-SKLDPRSE  QAKIGLAQMKLQQ----------------EDIDEAITLFEESADLARTM  EEKLQAITFAEAAKVQQRIRSD-PVLAKKIQETLAKLREQGLM------
NaucaTom70     KA-CEIDPRSE  QAIVGLAQLKLQN----------------EEVDLAIELFEKAADLSRTM  DEKIQAMTFAEAAKIQKRIRAD-PVISAKVDEALASYRSQGVI------
NeucrTom70     KA-LIIDPECD  IAVATMAQLLLQQ----------------GKVVEALKFFERAAELARTE  GELVNALSYAEATRTQIQVQENYPELASKLQGMSGGPGMR---------
AspniTom70     KALIIVDPECD  IAVGTMAQLLLQQ----------------GKVSQALKYFERAAELARTE  PEIVNAISYAEATRTQLEVQEKYPQLAARLQSMGAGFGGPPPM------
TriadTom70     KA-IELDNKCD  FAYETLATVEVQR----------------GNIDRGIELFEKALNLARAE  SELSHTLSLLIAAQTQIKVGKNLGVSPMMPF-----------------
NemveTom70     EA-ISIDSKCD  FAYETLATLEVQR----------------GNLEKSVELFDKAIDLVRTE  GEMAQTFSLREAIAQHHVTKKMGIVPNFMG-----------------
DanreTom70     KA-IEIDNKCD  FAYETMGTIEVQR----------------GNLDKAIDMFNKAINLAKSE  MEMAHLYSLCDAAYAQTEVAKKYGLKPPTL------------------
HomsaTom70     KA-IEIDNKCD  FAYETMGTIEVQR----------------GNMEKAIDMFNKAINLAKSE  MEMAHLYSLCDAAHAQTEVAKKYGLKPPTL------------------
XenlaTom70     KA-IEIDNKCD  FAYETMGTIEVQR----------------GNLDKAIEMFNKAINLAKSE  MEMAHLYSLCDAAYAQTEVARKYGLKPPTL------------------
```

(b)

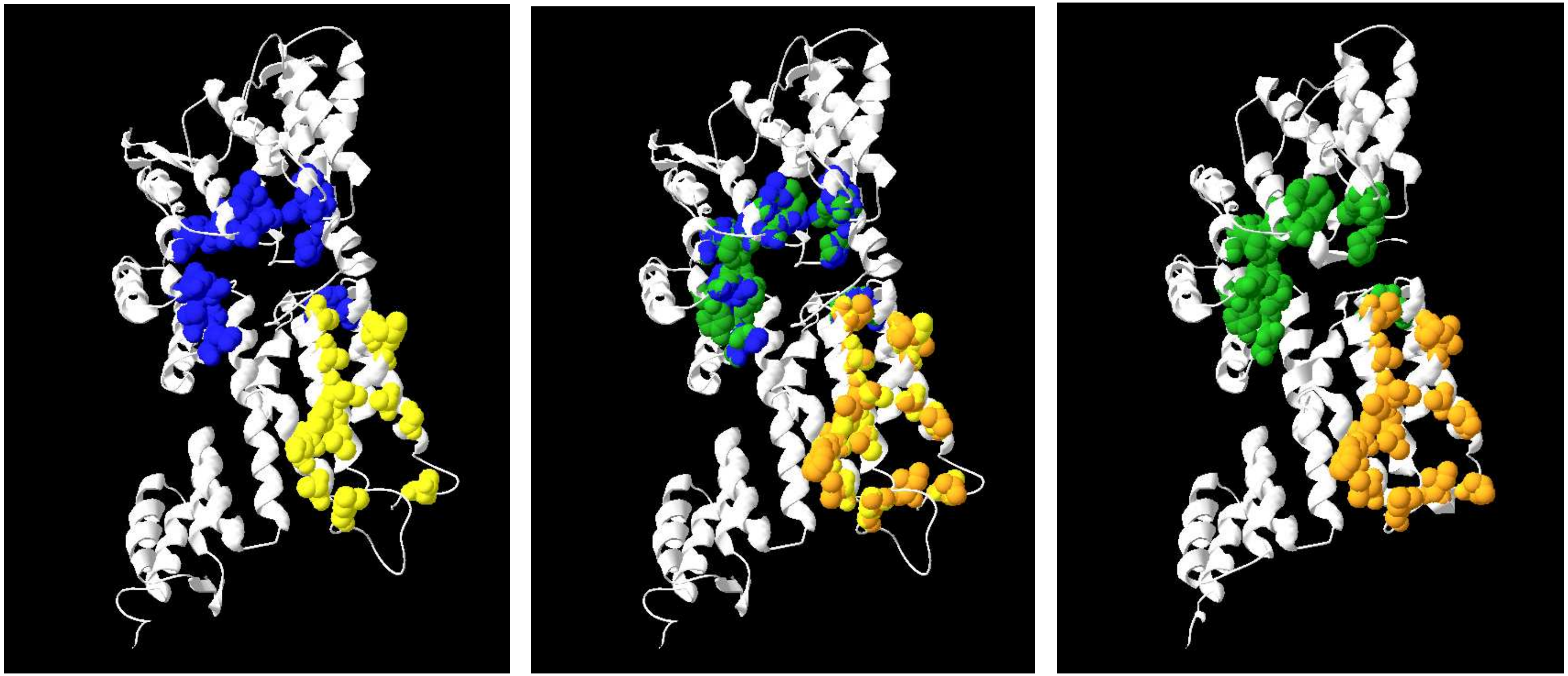

Ectocarpus Esi0232_0002 | Superimposition of the two models | Yeast Tom70
