# Supplementary figures and images for: In Silico Survey of the Mitochondrial Protein Uptake and Maturation Systems in the Brown Alga Ectocarpus siliculosus
Source: PLoS One. 2011 May 18;6(5):e19540. doi: 10.1371/journal.pone.0019540 (PMC3097184; doi:10.1371/journal.pone.0019540)

(a)

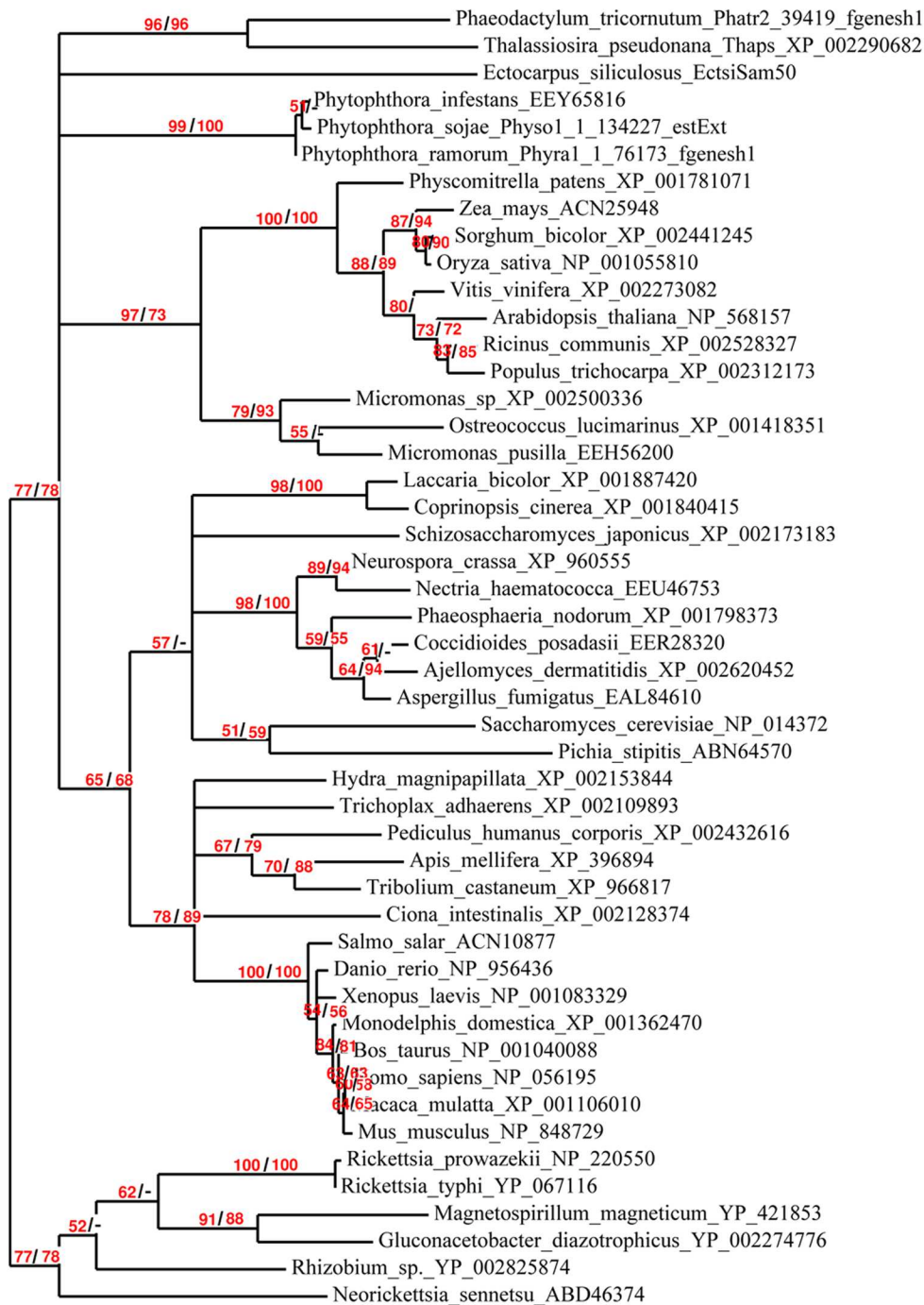

Stramenopiles

Plantae

Opisthokonts

Alpha-proteobacteria

(b)

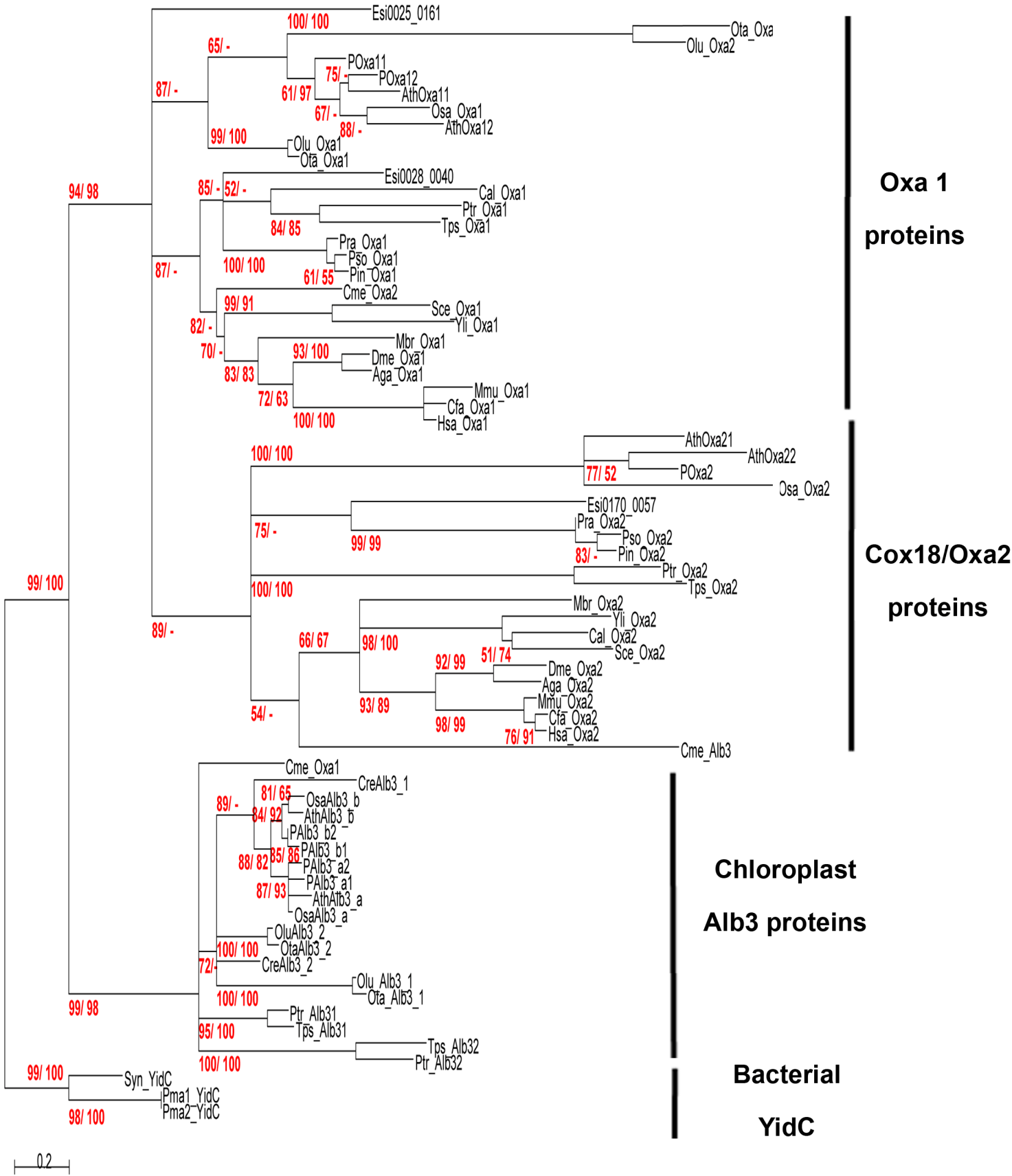

Supplement: Figure S4 — Maximum likelihood trees based on alignments of (A) Sam50 and (B) Oxa protein families. PHYML trees were constructed as described in the materials and methods section, with 113 and 88 amino acid sites for Sam50 and Oxa trees, respectively. Bootstrap values (100 replicates) above 50 are indicated for Maximum likelihood (first value) and Neighbour-Joining (second value) analyses. (PDF) [file pone.0019540.s004.pdf]
