# Supplementary material for: In Silico Survey of the Mitochondrial Protein Uptake and Maturation Systems in the Brown Alga Ectocarpus siliculosus
Source: PLoS One. 2011 May 18;6(5):e19540. doi: 10.1371/journal.pone.0019540 (PMC3097184; doi:10.1371/journal.pone.0019540)

(a)

Alignment of MPP and core subunits from diverse eukaryotes.

beta-MPP motif

```
SacceCore2       GVAHLLNRFNFQNTNT-RSALKLVR--ESELLGGTFKSTLDREYITLKATFLKDDLPYYVNALADVLYKTA---FKPHELTESVLPAARYDYAVA
NeucrCore2       GLTVGLEEFAFKNTNK-RTALRITR--ESELLGGQLQAYHTREAVVLQASFLREDLPYFTELLAEVISETK---YTTHEFHEL-VENCIHEKQAK
ChlreCore2       GASKVLEVAAFKATAN-RSTFRLTR--ELEKIGATSFARAGRDHVAFGVDATRLNQLEALEILADAVVNAR---YTYWEVRDS-LDAVKEQLAAQ
Esi0111_0016     GASLHLEGMAYKLTEA-RSSIRLMRQADVENVGGNLAASRGREKMVYVSECPPDSAGTVLSALAESVVSPK---IVPWEISDA-SAKLSEILQR
HomsaCore2       GTTHLLRLTSSLTTKG-ASSFKITR--GIEAVGGKLSVTATRENMAYTVECLRGDVDILMEFLLNVTTAPE---FRRWEVADL-QPQLKIDKAVA
ChlreAlphaMPP    GCSALLECLGFKATQH-RNTLRIMK--EVEKFGNTIVANASREQMSYTIDCLKTGFPAALELLLDCVLNPA---FEEGEVEDQ-KARLAALLGGK
Esi0268_0010     GTCHLLELMAFKSTAT-RSHQQVVS--EFEEMGGTTSTHGSRDQMLYCVDVLRDNLERAVELLADTLINPR---VTPEEVEEQ-KAVIGFQLEDT
HomsaAlphaMPP    GIAHFLEKLAFSSTARFDSKDEILL--TLEKHGGICDCQTSRDTTMYAVSADSKGLDTVVALLADVVLQPR---LTDEEVEMT-RMAVQFELEDL
NeucrAlphaMPP    GASHIMDRLAFKSTST-RTADEMLE--TVEKLGGNIQCASSRESMMYQAATFNKAIPTAVELMAETIRDPK---LTDEELEGQ-IMTAQYEVNEI
SacceAlphaMPP    GCTHILDRLAFKSTEH-VEGRAMAE--TLELLGGNYQCTSSRENLMYQASVFNQDVGKMLQLMSETVRFPK---ITEQELQEQ-KLSAEYEIDEV
Esi0011_0084     GVCSVIGASAFTGSEP-------------AIAAMGGHFTQTVDREVMTYSATVAEADVPKAMAVLADAVKATS---LSAESLQAS-KGAVLDDIEAA
SacceCore1       GVSNLWKNI-FLSKEN-SAVAAKE--------GLALSSNISRDFQSYIVSSLPGSTDKSLDFLNQSFIQQKANLLSSSNFEAT-KKSVLKQVQDF
HomsaCore1       GAGYFLEHLAFKGTKN-RPGSALEK--EVESMGAHLNAYSTREHTAYYIKALSKDLPKAVELLGDIVQNCS---LEDSQIEKE-RDVILREMQEN
Esi0098_0070     GVAHFLEHVAFKGTRK-RTQTQLET--EIEDMGAHLNAYTSREQTVYYAKVFKEDLGRGLEILSDILMNSL---IDEGAVHRE-RDVILREMEEV
ChlreBetaMPP     GVAHFLEHILFKGTKN-RSVKELEV--EVENMGGQLNAYTGREQTCYYAKVMGKDVGKAVNILSDILLNSN---LDARAIDKE-RDVILREMEEV
SoltuAlphaMPP    GVAHFLEHILFKGTKN-RSVKELEV--EVENMGGQLNAYTGREQTCYYAKVMGKDVGKAVNILSDILLNSN---LDARAIDKE-RDVILREMEEV
SoltuBetaMPP     GVAHFLEHMIFKGTEK-RPIRALEE--EIENMGGHLNAYTSREQTTYFAKVLGCDVPKAVDILGDILQNSL---LEEDKIIRE-RSVILREMEEV
HomsaBetaMPP     GTAHFLEHMAFKGTKK-RSQLDLEL--EIENMGAHLNAYTSREQTVYYAKAFSKDLPRAVEILADIIQNST---LGEAEIERE-RGVILREMQEV
NeucrBetaMPP     GTAHFLEHLAFKGTTK-RTQQQLEL--EIENMGAHLNAYTSRENTVYFAKALNEDVPKCVDILQDILQNSK---LEESAIERE-RDVILRESEEV
SacceBetaMPP     GTAHFLEHLAFKGTQN-RSQQGIEL--EIENIGSHLNAYTSRENTVYYAKSLQEDIPKAVDILSDILTKSV---LDNSAIERE-RDVIRESEEV
```

alphaMPP motif

```
SacceCore2       Q-YEVLANYLTSALSE-LSGLISSAKLDK--------------------------FTDGG
NeucrCore2       V-PHPETSVLVGLL---GGVSNIKWSPGFSLLAKATAANPGAEAFAHNYA-----YSDAG
ChlreCore2       DIKSSAAASVVKALLD-------EARPTMP-------YQRKEHEVFTSVNPFAFAYKGTG
Esi0111_0016     D-ASAPAYEVLGALLG---ARAAAAAPSAS--------------------GFSVSYTDAG
HomsaCore2       S-AEANAFSVLQHVLG--AGPHVKRGSNTT--SHLHQAVAKATQQPFDVSAFNASYSDSG
ChlreAlphaMPP    DVHGAVVMTVLNYLLG-GGNSFSSGGPGKGMHSRLYTRVLNKYAWVHSCASFNTTFNESG
Esi0268_0010     D-DLLVPTCVLQVLLG-GGDSFSAGGPGKGMYSRLYREVLNRFYWAEAAEAFSMIHDETG
HomsaAlphaMPP    E-EDFIPFAVLNMMMG-GGGSFSAGGPGKGMFSRLYLNVLNRHHWMYNATSYHHSYEDTG
NeucrAlphaMPP    D-DDIYALATLQTLLG-GGGSFSAGGPGKGMYSRLYTNVLNQHGWVESCVAFNHSYTDSG
SacceAlphaMPP    H-PDIYALATLQTLLG-GGGSFSAGGPGKGMYSRLYTHVLNQYYFVENCVAFNHSYSDSG
Esi0011_0084     S-KHSISLMMVQALLGFEYNERTVLGVNAA--SKWAQEIAEL-NLAAVATPFYKGYKDAG
SacceCore1       S-PNYFVAKLAAQIFG-SYNAFEPASRLQG--IKLLDNIQEY-QLCDNFNHFSLSYKDSG
```

(b)

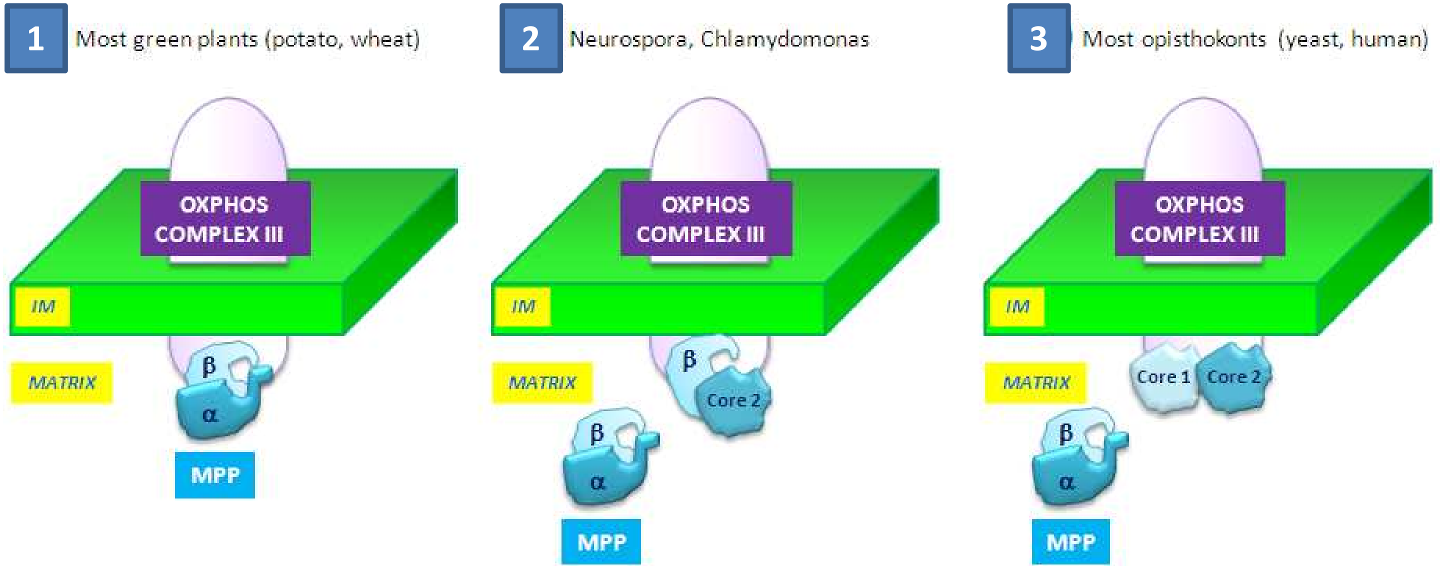

Supplement: Figure S6 — MPP proteins. (A) Alignment of MPP and core subunit motifs from diverse eukaryotes. Esi0111_0016, Esi0268_0010, Esi0011_0084 and Esi0098_0070 are the Ectocarpus sequences. The sequences from other organisms are from Homo sapiens (Homsa), Saccharomyces cerevisiae (Sacce), Neurospora crassa (Neucr), Chlamydomonas reinhardtii (Chlre), Solanum tuberosum (Soltu). alphaMPP and betaMPP indicate functional MPP subunits (shown in negative type). Core2 and core1 represent the corresponding non-functional MPP homologues inserted into the respiratory chain. The highlighted residues correspond to the key amino acids of alphaMPP and betaMPP activities. (B) Schematic representations of mitochondrial MPP complexes from diverse eukaryotes. Panels 1, 2 and 3 reflect the evolution of MPP proteins as suggested by [94]. See text for details. (PDF) [file pone.0019540.s006.pdf]
