# Supplementary material for: In Silico Survey of the Mitochondrial Protein Uptake and Maturation Systems in the Brown Alga Ectocarpus siliculosus
Source: PLoS One. 2011 May 18;6(5):e19540. doi: 10.1371/journal.pone.0019540 (PMC3097184; doi:10.1371/journal.pone.0019540)
Supplement: Table S1 — Comparative analysis of mitochondrial protein import systems across the eukaryotic tree. The table is based on searches of public databases of protein sequences. ALL, predictions obtained using all the subcellular localisation predictors listed in Materials and Methods. NI, not identified. (PDF) [file pone.0019540.s007.pdf]

Name	Alternative names	OPISTHOKONTS		PLANTAE			STRAMENOPILES							*ECTOCARPUS* DATA		
		Fungi	Animals	Land plants	Green algae	Red algae	Oomycetes		Pelagophytes	Blastocystis	Diatoms		Brown algae	Locus ID	PREDICTIONS	
		S. cerevisiae	*H. sapiens*	*A. thaliana*	*O.lucimarinus*	*C. merolae*	*P. infestans*	*A.laibachii*	*A.anophagefferens*	*B.hominis*	*P. tricornutum*	*T.pseudonana*	*E.siliculosus*	(protein size)	HECTAR	ALL
**OUTER MEMBRANE**																
**mtOM64**		0	0	1	1	0	0	0	0	0	0	0	0	NI		
**TOM complex**																
**Tom70**	Mas70, Mom72, Omp1	1	1	0	0	0	2	2	0	1	1	1	2	Esi0007_0019 (952)<br>Esi0232_0002 (739)	NO<br>NO	0/9<br>6/9
**Tom40**	Isp42, Mom38	1	1	2	1	1	1	1	1	NI	1	1	1	Esi0055_0058 (442)	YES	7/9
**Tom22**	Mas17, Mas22, Mom22	1	1	2	1	1	1	1	0	0	1	1	1	Esi0246_0018 (112)	NO	2/9
**Tom20**	Mas20, Mom19	1	1	4	1	0	0	0	0	0	0	0	0	NI		
**Tom7**	Mom7, Yok22	1	1	2	1	NI	NI	1	NI	NI	1	1	1	Esi0179_0016 (57)	NO	6/9
**Tom6**	Isp6, Mom8B, OM10	1	1	1	NI	NI	NI	NI	NI	NI	NI	NI	0	NI		
**Tom5**	Mom8A, OM7.5, OM5	1	1	1	NI	NI	NI	NI	NI	NI	NI	NI	0	NI		
**SAM/TOB complex**																
**Mim1**	Tom13	1	0	0	0	0	0	0	0	0	0	0	0	NI		
**Sam50**	Omp85, Tob55, Tom50	1	1	1	1	1	1	1	1	1	1	1	1	Esi0503_0006 (451)	NO	1/9
**Sam35**	Fmp20, Tob38, Tom38	1	0	1	1	0	0	0	0	0	0	0	0	NI		
**Sam37**	Mas37, Pet3027, Tom37	1	0	0	0	0	1	0	0	0	0	0	0	NI	NO	2/9
**Metaxin**		0	2	1	1	0	1	1	0	0	0	0	1	Esi0338_0018 (407)		
**INTERMEMBRANE SPACE**																
**MIA/ERV complex**																
**Mia40**	Fmp15, Tim40	1	1	1	1	1	1	1	0	1	1	1	0	NI		
**Erv1**		1	1	1	1	1	1	1	1	1	1	1	1	Esi0202_0015 (194)	NO	1/9
**Hot13**		1	1	1	NI	1	1	1	0	0	1	1	1	Esi0046_0129 (101)	NO	0/9
**TIM8/13 Complex**																
**Tim8**		1	2	1	1	1	2	1	NI	1	1	1	1	Esi0109_0044 (125)	NO	0/9
**Tim13**		1	1	1	1	1	1	1	NI	1	1	1	1	Esi0243_0012 (82)	NO	0/9
**TIM9/10 Complex**																
**Tim10**	Mrs11	1	2	1	1	1	1	1	NI	1	1	1	1	Esi0041_0146 (107)	NO	0/9
**Tim9**		1	1	1	1	1	1	1	1	NI	1	1	1	Esi0075_0052 (88)	NO	0/9
**INNER MEMBRANE**																
**TIM23 complex**																
**Tim50**		1	1	1	1	1	1	1	1	0	1	1	1	Esi0000_0471 (547)	YES	8/9
**Tim44**	Isp45, Mim44, Mpi1	1	1	2	1	1	1	1	1	0	1	1	1	Esi0086_0051 (602)	YES	8/9
**Tim23**	Mim23, Mpi3, Mas6	1	1	3	1	1	1	1	NI	1	1	1	1	Esi0047_0026 (206)	NO	1/9
**Tim21**		1	1	1	1	0	1	1	0	0	1	1	1	Esi0103_0070 (205)	NO	0/9
**Tim17**	Mim17, Mpi2, Sms1	1	2	3	1	1	1	1	1	1	1	1	1	Esi0117_0080 (264)	NO	0/9
**Pam17**	Fmp18	1	0	0	0	0	0	0	0	0	0	0	0	NI		
**Tim16**	Pam16, Mia1	1	1	1	1	1	1	1	0	1	1	1	1	Esi0237_0006 (155)	NO	6/9
**Tim14**	Pam18	1	1	2	1	1	1	1	1	1	1	1	1	Esi0159_0032 (209)	YES	8/9
**mtHsp70**	Ssc1, Ens1	1	1	2	1	1	1	1	1	1	1	1	1	Esi0010_0066 (688)	NO	1/9
**Mge1**	Yge1, hTid	1	1	2	1	1	1	1	1	1	1	1	1	Esi0000_0394 (281)	NO	2/9
**TIM22 complex**																
**Tim54**		1	0	0	0	0	0	0	0	0	0	0	0	NI		
**Tim22**		1	1	3	1	1	1	1	1	1	1	1	1	Esi0063_0018 (192)	NO	2/9
**Tim18**		1	0	0	0	0	0	0	0	0	0	0	0	NI		
**Tim12**	Mrs5	1	0	0	0	0	0	0	0	0	0	0	0	NI		
**OXA1, polytopic membrane protein insertion and folding**																
**Oxa1**		1	1	1	1	1	1	1	NI	1	1	1	1	Esi0028_0040 (411)<br>Esi0025_0161 (552)	NO<br>YES	1/9<br>8/9
**TAT system**																
**TatA/B**		0	0	1	1	NI	NI	NI	NI	NI	1	1	1	Esi0067_0034 (170)	NO	2/9
**TatC**	Orfx, MttB	0	0	1	0/1	1	1	NI	NI	NI	1	1	1	mt genome (254)		
