# Supplementary material for: In Silico Survey of the Mitochondrial Protein Uptake and Maturation Systems in the Brown Alga Ectocarpus siliculosus
Source: PLoS One. 2011 May 18;6(5):e19540. doi: 10.1371/journal.pone.0019540 (PMC3097184; doi:10.1371/journal.pone.0019540)
Supplement: Table S2 — Summary of the results of manual searches for components of mitochondrial protein import systems. Relevant hits are in bold characters. (PDF) [file pone.0019540.s008.pdf]

Name	Accession (aa length)	Protein sequence	Domains (Interpro, HMMPanther, Prosite, Pfam)	KOG database	TM Predictions	2D predictions	3D predictions
**OUTER MEMBRANE**							
**TOM complex**							
**Tom70a**	Esi0007_0019 (952)	MCSVFFEVVEPLDHATFLSVCDEVLRDSLLVLAPFGLPRHLREALGSEKEFIRLYGPRFRFILEAVSDVTYKERGVTESQVAVTLHKLTEEEQNPEAMAYLARMGDSLREIYSPARILELPTRAPTSTADLATIGSSLLGAGAVYTPGPQQAGRRGGRGGGAPLCDGTPRQPSRAGHAGRCSSSPAGDGDSSCAGENNKCSTAGRAEGGGGGGGGGDSRADKGETDHDKVQRERVASCLSALRGCREELEVFSDELEQAHIQALTEAFAGRYSGAAPPGDSAGGSSSSSQELKSRRSDVAALDEFAAVPFLDHELFMLVRAIEEERLGEEKGAGKEKESASGGGSSSSSATGACSESPTAADGDGDTSLCERPAACLEFLLTSQAVRVRADESMERGLDKEAAASLKEANILMEAFLEEGQRLPRPSRHLAELRAGLAKCEMRGGDLEAAIAHAEAALAEHPACGEAFLIRGQCRRELGDNGGALRDLVNAFVLQGNALNNAGDGSEAQAIEDVSRESCRARAGEEFSQRAAPNALPADWVVRSFLTSYDTKGLYEEHDAVFRLHGEDPVAAAAAAEGDEGGQAAAVTEFWQGLSLVREGKYAESIAKFSSSVSAFSSSAPVAVLGEGTGAGDTTRIQSLALEYCGSFLYLMGDMNTALEHLRLAGEVDETNAKSWVKRGSVLSDLGRREEAFECFDAAAAIAPRDSDLFLHRGQGHLLANDFRKATADLRRSVELCPTMPISRAAWGVALFKLATAAELPSPSSLSKCVQVLEESRELFPENPEVLFFFAEVLISMGDFKKGLEFLQTAASLDPECPVPYVNAARAYLGMNDTKAARRQVVAVVQQGYDFFLDGHAVCPATGCCASENLSVIGRRMRFILRSMLMPMCVLLGTVKSLFLFHSFVGICSRFSLAATTWCMVQLFWDIRPIVRGRLCLWSNRLLRFRDFVCDR	IPR011990 (Interpro), Tetratricopeptide-like helical IPR013026 (Interpro), Tetratricopeptide region PTHR23083 (HMMPanther), TETRATRICOPEPTIDE REPEAT PROTEIN, TPR PF07719 (Pfam), Tetratricopeptide repeat, 0.0011	**KOG0547, Translocase of outer mitochondrial membrane complex, subunit TOM70/TOM72**	DAS: 4 Transmembrane segments, cutoff 2.2 TMpred: 4 Transmembrane segments	PHYRE: multi α-helical structure	PHYRE: 100% Tetratricopeptide repeat (TPR), 2e-40 **100% crystal structure of the yeast tom70, 1.2e-37**
**Tom70b**	Esi0232_0002 (739)	MGQGIKVCSSQGPSIPLLPARRVQKHHLRTPGPRREGRGRWRMDQSKLAVLGTVGLVATAAVCCAIVFSGRFSFAGSSTGKPFASSPGTTADAQGVAASNKVIDDEDDKDKLTGGVSTEKGTGKGDSGGGDGSRAAPGSGGASVPAAEAAAARGVAVGQGKAEDGEALIVRFNRANSKAKKLFTGQRYALAAEQYGIALELCDELPNHDNKRTALHNNRGAAYEKDGQYALALADCSMCLSREVGHKFARVRKSRVLEAMGKHEEALSEVCAHLLLERDRVQAKAALNPSEPLTPPAPPANLEGLLQKVASKRADAILLEREQTAEKQEAAAAGGAGTGTEKLKPLVKQVVMELLRSFGSFAQLERRYKGMEETAITRELKDAEKAGKEGDGSASATTTSASRVSSLLDRGLLRMVKRNYDGAREDIFEAAELLSTLTEADPSEAGADEVPPHVKASVWEWQGTFLQLSGKLDEAMEAYRRCGEEMEAEGEEYPADVLIKMAWVCMDKEDMDAAKDLFARAGEAHPEYGSSFAHRARLDSEKDGAEQVRSFLRKAIELNSEDAFAWEQLCRIHVQAGDIPKATSTIEEGLEFVPNSDALLTLKAELKYSMAMKAGDASSCAAILEVFDAAIRANPSSPVLYLNKASCLLQMMSDVGGAMELLEKGVSVDPTSVNALVQLANLKIMVAREMAEAEAATALLDKAVALCTTKEELMETLSVRVATEGRIKGALLLGRTTLG	IPR011990 (Interpro), Tetratricopeptide-like helical IPR013026 (Interpro), Tetratricopeptide region PTHR23083 (HMMPanther), TETRATRICOPEPTIDE REPEAT PROTEIN, TPR PF00515 (Pfam), Tetratricopeptide repeat, 0.00022	KOG4234, TPR repeat-containing protein	**DAS: 51-68, cutoff 2.2 TMpred: 50-69 (in-out) score=2175 48-69 (out-in) score=2252**	PHYRE: multi α-helical structure	**PHYRE: 100% crystal structure of the yeast tom70**
**Tom40**	Esi0055_0058 (442)	MATRLPRCFAAAAAAASATSSASSGSNSSRATPPAAASSKHSQARGDGGKEPWRREAASTPADAAGCTKLASGGCRRGAVGGTAAAAGAASVRPSIEALEASLQGLLGSVPLVRIAHADGAAAVAVEAAGAEGEGDAAAAVSELEDGDKPEYIQRADEQAAQMRQQLRRPAQFADQFLEVSQLTQVHSFEGFRLDMSKAVTPTFFTSHNVFFGNPQFPSGHYQGTLTVVGESETMVRASMDADCNVSMDAHAPLGMPGLAGKLTVHQGKNDVIQGTALYHGDTCSGQISLGTGPTEISYNQAVTPHLSMGGQGQFSSAQQAVGLLYGFKYNTPSWAVLGRLIGGGANVVTAQYLRRVVPGRVTLGAEYQAQLGAGSQMMVGAEFQLKQSKMSASVDSNGKVDSTLELKCGTFPTLPVTLTISSSLDHSEDKQTFGLALTCGQ	IPR001925 (Interpro), Porin, eukaryotic type **PTHR10802 (HMMPanther), MITOCHONDRIAL IMPORT RECEPTOR SUBUNIT TOM40** PF01459 (Pfam), Eukaryotic porin, e-value=2.4e-25	**KOG3296, Translocase of outer mitochondrial membrane complex, subunit TOM40**	**DAS: 11-17, cutoff 1.7 TMpred: 5-23 (in-out) score=778, 5-23 (out-in) score=565** TMHMM: 0	**PHYRE: N-terminal α-helix following by at least 16 β-strands**	**PHYRE: 25% Transmembrane beta-barrels, porins superfamily**
**Tom22**	Esi0246_0018 (112)	MAKKLTFNDEKRDNGPGVLQTLVVTGRSLGVAGFGWAQWAAKKTGRTGWVLLTTAVVTLVPLVFEITREAQLIEQEKIHINALLAEGKTRQEIAQMGLYSALDPNVMGPEAS	**PF04281 (Pfam), Mitochondrial import receptor subunit Tom22, 1.3e-05**	NI	**DAS: 50-63, cutoff 2.2 TMpred: 47-64 (in-out) score=1332, 48-64 (out-in) score=1678** TMHMM: 0	PHYRE: 3 or 4 a-helices	PHYRE: 0% TraM-like
**Tom7**	Esi0179_0016 (57)	MGEKIMRRRPRRKSAPLLPKVLAVAKPIMRWGIMPAVLLMGMRSEPNPTLLEVLYPL	**PF08038 (Pfam), TOM7 family, 1.2e-06**	NI	**DAS: 32-40, cutoff 1.7 TMpred: 23-42 (in-out) score=329, 24-44 (out-in) score=156** TMHMM: 0	**PHYRE: Protein with a single central α-helix**	PHYRE: 15% doa4-independent degradation protein 4
**Tom6**	NI						
**Tom5**	NI						
**SAM/TOB complex**							
**Mim1**	NI						
**Sam50**	Esi0503_0006 (451)	MVIPLDELPLRVRTVKIVGNNRTKPYVVEDQLQDAYEATTVGDVYGGLVEGAQRLDGLGLFESVQVSMDAVDDGSLDQTDVTVTVKEKNWYLLQSGATTTGTAKGNLDASEFSNLRYSVAGALRNPLGHGEMLDVGYNSPIKGQEGHTVSAKLHLPHLFRTPVSGTLEAIMDTVVFEDENTRQEHWTLTVERHASSDVETRQAKRITAIRTALSTASCYDQTXXXXXXXXXXXXXXXXXXXXXXXXPSPPVGDTHTFGMRIGLMGDTSFAKGMLEMQRHIPLFGVELGPETYSPVTLSLCASGGAIRPFGPSKRTFFSDRFNLGGPMTLRGFPFYGAGPRSPKEEGGCEGGDALGGDIRYTASASLGFPFPVPAMATAGWRGYLFTNLGNLTTWDTPLKQYGRDTRVSVGVAAAWNFLGVGRLEINYAHVLRRSPRDLHRERPLQFGFGVSFE	IPR000184 (Interpro), Bacterial surface antigen (D15) IPR010827 (Interpro), Surface antigen variable number PF01103 (Pfam), Bac_surface_Ag, 3.3e-24	KOG2602, Predicted cell surface protein homologous to bacterial outer membrane proteins	**DAS: 0 Transmembrane segments** TMpred: 2 Transmembrane segments	**PHYRE: N-terminal α-helix following by at least 16 β-strands**	**PHYRE: 100% structure of the membrane protein fhac: a member of the2 omp85/tpsb transporter family**

**Sam37/ Metaxin1**	Esi0338_0018 (407)	MASEEHGDAVPVRTSWNPLRALRSWGRIEEQPDLEQPGPVITHGSALDVMVVTQFRPAWEIQAHLRFVRLPYRVENSSYMGSAATGLYPALTDGQFVLRSEDAAGHIASRRSDVDVGLTEAEKVEAQALAMLVREGLQPLLRVMRYMGDEGEVRQTVHPPMKKALSWPLSWWSPAAEGRRSKRESAVRGLDRLSKAELIGRAKEMYAALDLRLGNSKEAFFFGSRPTSVDAVVFGHLAEAWTIAVLLDLLPAFDNLSRLFRHVCDNYFRPGSFPPPSSGGEGVAESKKSGSEDRLRDAMLRADYVNSHNAFNQLAGCALCSEVPYIEDPYPPRPIANAGIPPPVLAGDLPGVEGAQGTAAAGSTTAAAAAAEAAAGKATAANESLPVKYTVISIAAFMVLSNILSRG	**PTHR12289 (HMMPanther), METAXIN RELATED** PF00043 (Pfam), Glutathione S-transferase, C-terminal domain, 0.022	KOG3027, Mitochondrial outer membrane protein Metaxin 2, Metaxin 1-binding protein **KOG3028, Translocase of outer mitochondrial membrane complex, subunit TOM37/Metaxin 1**	DAS: 2 Transmembrane segments TMpred: 2 Transmembrane segments	PHYRE: α-helix structure	PHYRE: 100% structure of glutathione s-transferase iii in apo form
**Sam35/ Metaxin2**	NI						
**MDM complex**							
**Mdm10**	NI						
**Mdm12**	NI						
**Mmm1**	NI						
**INTERMEMBRANE SPACE**							
**MIA/ERV complex**							
**Mia40**	NI						
**Erv1**	Esi0202_0015 (194)	MPSSRRRSKDPADCEDPACADMADLLRKGRALAAKNKQKAASNTATDGKAAGSQAQPSSSAAETDEHAASSSRNDGCPLDKGELGAATWGLIHTTAAHYPEKPSKETQDQARALVTGLAGLYPCTYCRKDFREEVRKLPPDVSSRVALSLWACQQHNLVNEKIGKPTFGCTLPALDERWKKGKPSCWEGGAEGV	**PTHR12645 (HMMPanther), ALR/ERV IPR017905 (Interpro), ERV/ALR sulphydryl oxidase IPR006863 (Interpro), Erv1/Alr PF04777 (Pfam) Erv1 / Alr family, 7e-25**	**KOG3355, Mitochondrial sulfhydryl oxidase involved in the biogenesis of cytosolic Fe/S proteins**	DAS: 1 Transmembrane segment, cutoff 2.2 **TMpred: 0 Transmembrane segments**	**PHYRE: Four-helical up-and-down bundle**	**PHYRE: 100% FAD-dependent thiol oxidase**
**Hot13**	Esi0046_0129 (101)	MLVPGQVSIQFPCCKRWYECPECHDERENHPQRISTKNVAFACKTCRKIFVKDLSIYGTEDSACPHCETVFVLPAVTPEAKVAQLALSILAAEVSAEVSKF	IPR008913 (Interpro), Zinc finger, CHY-type PF05495 (Pfam), CHY zinc finger, 8.1e-13	KOG1940, Zn-finger protein	DAS: 1 Transmembrane segment, cutoff 2.2 **TMpred: 0 Transmembrane segments**	PHYRE: 2 or 3 α-helix structure	PHYRE: 95% ring finger and chy zinc finger domain
**TIM8/13 Complex**							
**Tim8**	Esi0109_0044 (125)	MSWFRGKKEPEPESTPEPTFSSDTSAFEGSTNFASGPPSRGGGGATSAGMGDLQAAMQIEQQKAQMQAIVSRLTDLAFTKCIQKPSSSLSSSEQSCINATVLKYFDTSEFVLGRLMKSQQGGGDI	IPR004217 (Interpro), Mitochondrial inner membrane translocase complex, Tim8/9/10/13-zinc finger-like **PTHR21535 (HMMPanther), MAGNESIUM AND COBALT TRANSPORT PROTEIN/MITOCHONDRIAL IMPORT INNER MEMBRANE TRANSLOCASE SUBUNIT TIM8** PF02953 (Pfam), Tim10/DDP family zinc finger, 6.4e-15	**KOG3489, Mitochondrial import inner membrane translocase, subunit TIM8** KOG1733, Mitochondrial import inner membrane translocase, subunit TIM13	**DAS: 0 Transmembrane segments, cutoff 1.7 TMpred: 0 Transmembrane segments**	PHYRE: 2 α-helix structure	**PHYRE: 100% tim8-tim13 complex**
**Tim13**	Esi0243_0012 (82)	MDGQASAEQQQAIIQQLQEQVQAKALQELMTQMTDQCFNRCAKTSSGDRINSSEQGCLAMCMDRYMDTMGLVNKAMVAKANR	IPR004217 (Interpro), Mitochondrial inner membrane translocase complex, Tim8/9/10/13-zinc finger-like **PTHR19338 (HMMPanther), TRANSLOCASE OF INNER MITOCHONDRIAL MEMBRANE 13 HOMOLOG** PF02953 (Pfam), Tim10/DDP family zinc finger, 1.6e-16	**KOG1733, Mitochondrial import inner membrane translocase, subunit TIM13** KOG3489, Mitochondrial import inner membrane translocase, subunit TIM8	**DAS: 0 Transmembrane segments, cutoff 1.7 TMpred: 0 Transmembrane segments**	PHYRE: 2 α-helix structure	PHYRE: 100% Tim10-like, **100% tim8-tim13 complex**
**TIM9/10 Complex**							
**Tim10**	Esi0041_0146 (107)	MSWFGNSEPEPPSGPSPMDLAKQEVDMYSDLFTKMSGLCFKKCVVKMHGESDLNVGEMSCVDRCVSKYMEAQEKVGVILKRAEESLAAQQGGVGGPAAGGVPGVPGR	IPR004217 (Interpro), Mitochondrial inner membrane translocase complex, Tim8/9/10/13-zinc finger-like **PTHR11038 (HMMPanther), MITOCHONDRIAL IMPORT INNER MEMBRANE TRANSLOCASE SUBUNIT TIM10** PF02953 (Pfam), Tim10/DDP family zinc finger, 2.8e-16	**KOG3480, Mitochondrial import inner membrane translocase, subunits TIM10/TIM12**	**DAS: 0 Transmembrane segments, cutoff 1.7 TMpred: 0 Transmembrane segments**	PHYRE: 2 α-helix structure	**PHYRE: 100% crystal structure of the tim9 tim10 hexameric complex**
**Tim9**	Esi0075_0052 (88)	MTPVQQQDFLQHLESQQRKDSLAMYNNLVFRCFDECSKSFRSKRLDDGETKCINVCAEKFIKLTSRVALRFQDIQQQKAKDEAAGVQR	IPR004217 (Interpro), Mitochondrial inner membrane translocase complex, Tim8/9/10/13-zinc finger-like **PTHR10898 (HMMPanther), MITOCHONDRIAL IMPORT INNER MEMBRANE TRANSLOCASE SUBUNIT TIM9** PF02953 (Pfam), Tim10/DDP family zinc finger, 1.9e-14	**KOG3479, Mitochondrial import inner membrane translocase, subunit TIM9**	**DAS: 0 Transmembrane segments, cutoff 1.7 TMpred: 0 Transmembrane segments**	PHYRE: 2 α-helix structure	**PHYRE:** 100% Tim10-like, **100% crystal structure of the tim9 tim10 hexameric complex**
**INNER MEMBRANE**							
**TIM23 complex**							
**Tim50**	Esi0000_0471 (547)	MLLGRVGTLLPSCSGRVATRRFLAKKAAGGGGGGKKGSGTPKKGPAAIKARGAMPVSKAGKQGSKTGKTGGAAAREAAATTTSTAKAAAAEAAASVKPPPVGGGGGFTKSEMLAARKARKASVTAKTAAAAATAKAAAPAAGAGAGRTGGAGAAASAAGAGGAKGQGKWGWGTKLVAGTSVGLAALGIAWQLKPDEMRKLLDDSPIDHFFIWFMGKWALYSSPVKDKLLLDCPLPPGALPPPTLVLDLEGTLLGTIYTRKKGWRVAKRPGLDAFLKEMSQLYEIVVFTDSMGGLADEWITQMDPQGTISQRVYRDGTRYIDGKYVKDLSALNRPLEQTLIIDDNADCISMQPENAIKVKAFSLEDGSDPTADTALYDLAPFLRALATQGVADFRDVLRPHVGEDSNAVVADFRSKVNAVRQKEDAEKSKGLGGLVRQIAPVVGAGGPAAGMGGMLTSKDIVGDAPETLSPGMAAAAAATAGGKGGGKGGGGSKPLAEKQKGGLWKSLQEGNKEREEDFMRRNEAFQRVMEKRMAREKAKRDEQAQQQ	PTHR12210 (HMMPanther), NUCLEAR LIM INTERACTOR-INTERACTING FACTOR-RELATED IPR004274 (Interpro), NLI interacting factor PF03031 (Pfam), NLI interacting factor-like phosphatase, 4e-37	KOG2832, TFIIF-interacting CTD phosphatase, including NLI-interacting factor (involved in RNA polymerase II regulation)	**DAS: 1 Transmembrane segments, cutoff 2.2** TMpred: 3-4 Transmembrane segments	PHYRE: multi α-helical structure	PHYRE: 100% HAD-like, NLI interacting factor-like phosphatase

**Tim44**	Esi0086_0051 (602)	MLRLGRHGSTLKACSLRQPAAAYARATRPSSIWRRDPIVPSGPHDNIVRGITTNTFTNTTTNTEQQRSVALSRWIQADRRRGPHVGPAPAASVTQLQQWRGLASGSSGKGKGGEEEGGMFDRLKKTFTEEIDKNPSLKESLSKLKDAEQKLRENPSVKENLAKLKEAEKKFRENPSVQENLSKLKDAEKKWREDAKSKQGEFAEQAEKAREKAQQGGGAFQEKFQELKQTLEEKLGKVGGESGDGKDGGEGKDGAENEFVRASKEKFSGAQKALDENSLFSKLRVKTQAFREAVAGASSELFPSEGQKAESAVVKKLKQPKKEKKKKKKKKDGKEKAPVEGEDDEEEEEEEEEKGGMGSLMIVKTAGEAWERLQERLKESPIIQDLLGASRVVASGSLGQGAKSAKDTVKDKVEDVQEAWETSQHPLVYKLSSAWDSLTAESDEGIGVRELRRLDPSFSVEDWKRDIQELFLPEFMSAFLRGDVKLLKQWTGEACYNKLASEAKQRKADGMVLDPHVLDIRQGEVLAIKADAGKANPTIALQFMCQQINCVRNKKGEILEGAEDDIRATYYILAFQREFNDDEAELRWRVVDMMVVGAFPWY	**IPR007379 (Interpro), Mitochondrial inner membrane translocase complex, subunit Tim44-related PTHR10721 (HMMPanther), MITOCHONDRIAL INNER MEMBRANE TRANSLOCASE SUBUNIT TIM44 PF04280 (Pfam), Tim44-like domain, 6.2e-28**	**KOG2580, Mitochondrial import inner membrane translocase, subunit TIM44**	**DAS: 0 Transmembrane segments TMpred: 0 Transmembrane segments**	PHYRE: multi α-helical structure	**PHYRE: 100% TIM44-like**
**Tim23**	Esi0047_0026 (206)	MASEPTEESGGVQDPYSDASAKYGPLKLPELQNVDLSKMYGGAAQGKEPEYLDYNIKGRGFWERMPYNAGALYITGILGGGAAGVREGFAKAPNRRSRVLLNSIMNHAGKKGSFYGNTFAVLATYYTCAETLLDHFEVDQMGPVQQAGLGEIINPLLAGASTGLLYKSSAGPRLALMASVAGLGAVGVAYAVDKTSASVLGQGIIF	**PTHR15371 (HMMPanther), TIM23** IPR003397 (Interpro), Mitochondrial inner membrane translocase complex, subunit Tim17/22 PF02466 (Pfam), Tim17/Tim22/Tim23 family, 3.4e-13	**KOG3324, Mitochondrial import inner membrane translocase, subunit TIM23**	**DAS: 4 Transmembrane segments, cutoff 1.7** TMpred: 3 Transmembrane segments	**PHYRE: 4 α-helices structure**	PHYRE: 25% Mitochondrial carrier
**Tim21**	Esi0103_0070 (205)	MMSKASRKGDDSKGEQQQQQKQQGEEQQYEDADYEPEPEEKISIVKTVAGVCVLGLVVAALSATITELWPSHMAPQSIMSHAHDVFQADPDTANHFGTPLKGYGRDNNRHREGRRNFVEHVDHEDPIDKSKRTRVRFNVEGPHGHGMAYAEVSNKMESGEWVYLCVQDLQTGHVITLHDNRALLMAQAQAGSDEEKNAFRKMLGQ	PTHR13032 (HMMPanther) FAMILY NOT NAMED **IPR013261 (Interpro), Mitochondrial inner membrane translocase complex, subunit Tim21 PF08294 (Pfam), TIM21, 9.8e-12**	KOG4836, Uncharacterized conserved protein	**DAS: 44-67, cutoff 1.7 TMpred: 51-69 (in-out) score=1978, 47-63 (out-in) score=1876**	**PHYRE: 2 N-t α-helices followed by 4 β-sheets**	**PHYRE: 100% structure of the ims domain of the mitochondrial import2 protein tim21 from s. cerevisiae**
**Tim17**	Esi0117_0080 (264)	MGDRDPCPHRIVGDVGGAFAFGLAGGGIWHSVKGFRNSPKGQGTQGALKAVMYRAPVLGGNFAVWGALFSVCDCSLVAVRHKEDAWNPILSGAATGGILALRAGPRTAAKNAVVGGALLAVIEGMGILLSRYMAQVEPPPMEAGGDAGGAGGGLGAPQAPRPLGLAPPLPPVGAPAGYRPMGDGGEVDSSATRSHDVVTGSGFETGSRFEDTLATQDPFASNTGDRYLEGGAAPRGAGDAAAAAAEGAESRGWFGRMIGRGGGK	**PTHR10485 (HMMPanther), MITOCHONDRIAL IMPORT INNER MEMBRANE TRANSLOCASE SUBUNIT TIM17** IPR003397 (Interpro), Mitochondrial inner membrane translocase complex, subunit Tim17/22 PF02466 (Pfam), Tim17/Tim22/Tim23 family, 4.1e-34	**KOG1652, Mitochondrial import inner membrane translocase, subunit TIM17**	**DAS: 4 Transmembrane segments, cutoff 1.7 TMpred: 4 Transmembrane segments**	**PHYRE: 4 α-helices structure**	PHYRE: 25% ammonium transporter amt-1 from a. fulgidus (ma)
**Pam17**	NI						
**Tim16/Pam16**	Esi0237_0006 (155)	MANPFARIIAQLAVASAGIVSRAFVSAYSQAVHNARTGTLESAKAMSRTSKLSTLEAMQILNLQKGEMKPDLIKQRYDQYFGINDPDKGGSFYLQSKVFRAKEALDEQLALQAKQASEEAALKAKAAAGGKGGAGGAKARPAAGGARRPSANRRR	**IPR005341 (InterPro), Protein Transporter, Pam16** IPR001623 (InterPro), Heat shock protein DnaJ, N-terminal PF03656 (Pfam), Pam16, 2.7e-26	KOG3442, Uncharacterized conserved protein	DAS: 1 Transmembrane segment, cutoff 2.2 TMpred: 1 Transmembrane segment	PHYRE: multi α-helical structure	**PHYRE: 100% structure of the tim14-tim16 complex of the mitochondrial2 protein import motor, 1.5e-09**
**Tim14/Pam18**	Esi0159_0032 (209)	MAARPLVRIAGLKPSVSHRCPLPLLGRLGATSSSNRESGANDRHARAQALVSQARDIHVSRRNESSVLITGAGIAVSAMVARYGLMEYQKYQAAHPEITEEADSGAAGDQTAAGETGKASPQGAAKGAGASAGGFFGAFGKRHYDGGFEEKMTRKEAALILGVRESATAQRIKDSHRRILMINHPDKGGSKYMAAKINEAKEILLKGRK	PTHR12763 (HMMPanther), UNCHARACTERIZED IPR001623 (InterPro), Heat shock protein DnaJ, N-terminal PF00226 (Pfam), DnaJ domain, 6.5e-09	KOG0723, Molecular chaperone (DnaJ superfamily)	**DAS: 1 Transmembrane segment, cutoff 2.2** TMpred: 1 -2 Transmembrane segments	PHYRE: multi α-helical structure	PHYRE: 100% j-domain of dnj-12 from caenorhabditis elegans, 6.9e-12
**mtHsp70**	Esi0010_0066 (689)	MLGARLGRSVAKRQLAHIRGGPSSAPSPLPLCAAASRRALSSKGPAGDIIGIDLGTTNSCVAIMEGRSARVIENTEGARTTPSVVAFQPDGTRLVGLVAKRQSVTNPESTLYATKRLIGRRFKDKEVQGVQKLVPYKIVESQDGAAWVEVQGNKMSPSQVGSMVLTKMKETSESFLGRPVGKAVVTVPAYFNDSQRQATKDAGRIAGLEVMRIINEPTAAALAYGLDKADGKLIAVFDLGGGTFDISILEISGGVFEVKSTNGDTMLGGEDFDEVLLKHLLSEFRKESGIDLSGDTLAMQRLREAAEKAKRELDGMPQTDVSLPFITADSSGPKHMNVKVTRAEFENLVDGLIERAIKPCKDCMRDAGLETDEVHEVLLVGGMTRMPKVQSKVEAFFGKPPSRGVNPDEVVAMGAAIQGGVLRGSVKDILLLDVTPLSLGIETLGGVMTKLINRNTTIPTKKVQTFSTAADNQSQVQIKALQGEREMATDNKMLGQFDLVGIPPAQRGVPQIEVSFDIDADGILHVGAKDKQTGKEQSIVIQSSGGLGEADIEKMVRDAEEHAEADKKRRDLIEAKNSADSAVYGTEKNLLEHKDKIPEDVKADVQKAIDELKAVMESEDAEQIKEKVQALQTAALKIGEAIYGNKGAAGDDAEGAPEGGDAESDKENIQDAEFEEKKEEGEGEKKSQ	**IPR018181 (InterPro), Heat shock protein 70, conserved site PTHR19375 (HMMPanther), HEAT SHOCK PROTEIN 70 (HSP70) PF00012 (Pfam) Hsp70 protein IPR001023 (InterPro), Heat shock protein Hsp70 IPR013126 (InterPro), Heat shock protein 70** IPR012725 (InterPro), Chaperone DnaK	KOG0102, Molecular chaperones mortalin/PBP74/GRP75, HSP70 superfamily	**DAS: 0 Transmembrane segments, cutoff 2.2** TMpred: 0-1 Transmembrane segment	PHYRE: α/β structure	PHYRE: 100% crystal structure analysis of sse1, a yeast hsp110, 1.4e-45 **100% crystal structure of the 70-kda heat shock cognate protein2 from rattus norvegicus in post-atp hydrolysis state, 1.8e-41**
**Mge1**	Esi0000_0394 (281)	MVVASLSTAADVGKEQSSLAARAGGQGVWHPHAFAGRGQRGLCPLGGVRSLSTNAKEDEEDKAKEGGGAGGEAEQSNSKGEGEDEDEDQEGLAEKLAKTEERLMDTKEKALYLAAEMENVRSIAKKDAESARLYAVQKFAKQLLDVADNLERAIASAKEAEGEGGGDSSHDVLLQGVEMTSNELTKVFRSQGLEKYGEVKDKFDPHLHDAMFEFVNPAQEPGTLGQVLKCGYTLHGRVIRAAQVGTVKAA	IPR000740 (InterPro), GrpE nucleotide exchange factor IPR013805 (InterPro), GrpE nucleotide exchange factor, coiled-coil IPR009012 (InterPro), GrpE nucleotide exchange factor, head **PTHR21237 (HMMPanther), GRPE PROTEIN, MITOCHONDRIAL** PF01025 (Pfam), GrpE, 8.4e-52	KOG3003, Molecular chaperone of the GrpE family	**DAS: 0 Transmembrane segments, cutoff 1.7 TMpred: 0 Transmembrane segments**	PHYRE: multi α-helical structure + one β-strand	PHYRE: 100% crystal structure of the nucleotide exchange factor grpe2 bound to the atpase domain of the molecular chaperone dnak
**TIM22 complex**							
**Tim54**	NI						
**Tim22**	Esi0063_0018 (192)	MPADDDRRHGECCNQQGPEGLRSLHPQLGSRLPFFLPWVPRPAAGAADAMPTPPVLTESCGFKLIMGTVGGAGMGFVFGLFLGAMGDMQPLQMINGREVPQAPFREQARLAYKQTADRSLSMGRNFASFSAIFMGSECVIEKMRGKTDMMNSVYAGCATGAAFGMKQGPQAACFGCVGMATFSALMDKLMGH	**PTHR14110 (HMMPanther), MITOCHONDRIAL IMPORT INNER MEMBRANE TRANSLOCASE SUBUNIT TIM22** IPR003397 (InterPro), Mitochondrial inner membrane translocase complex, subunit Tim17/22	**KOG3225, Mitochondrial import inner membrane translocase, subunit TIM22**	DAS: 3 Transmembrane segments, cutoff 1.7 TMpred: 3 Transmembrane segments	**PHYRE: 4 α-helices structure**	PHYRE: 70% structure of the multidrug transporter emrd from2 escherichia coli, 1.3
**Tim18**	NI						
**Tim12**	NI						
**Tim17/22-like proteins**							

**Tim17/22-A (Tim22-like)**	Esi0019_0065 (217)	MAYCRLLLQGLLLSVALGCNALPGWSRNSVGAAVQPHHGLAWTSRTPTTRTLRRQFPVAAAVAS<br>TSHLTTGRTASGVMENCMEACLTTMQSFIGGTIMGCVIGGVMGGSFGRVPDVGWLKSVQTKAVE<br>MGGNWGQLSAAFTGFTSISTVIRGRNDKWDQVLGACGAGAFLNRAKGPQGMAQGAATYGLFSL<br>IFAFPSGQDDELDVVDIPVDAPKTR	PS51257 (Prosite), PROKAR_LIPOPROTEIN IPR003397 (Interpro), Mitochondrial inner membrane translocase complex, subunit Tim17/22 PF02466 (Pfam), Tim17/Tim22/Tim23 family, 5.4e-17	**KOG1652, Mitochondrial import inner membrane translocase, subunit TIM17**	DAS: 4 Transmembrane segments, cutoff 1.7<br>TMpred: 4 Transmembrane segments	**PHYRE: 4 α-helices structure**	PHYRE: 5% ammonium transporter amt-1 from a. fulgidus
**Tim17/22-B**	Esi0046_0104 (318)	MGWFGLGRRKPADESSPSSPTLSTDGINMSTGCGAQAASESTGRSCTPMAEPVGAPKTTLNAPAA<br>AMRADACDKASPVEEQQEQQQGEGVADSSPGYAHRLKAAFLDTVATAKRAPETMEEAMAVIKG<br>ERAVKPDYGRAVGTVSAAFAGGCAHGWWHGAALAAKYPPHQRSIVRVRAGARRGLRFATFALI<br>FEGSSAVMEALRRKKDFVGGTVGGALAGMAYGVSGGVAAARSGLFYGVWLGGVFGLFRNHVA<br>SLREQARLAEEDERLAQEERDAIGETALRRSIAGIDAQVRTWPTAAADSEDETDTTELASSQRTP	IPR003397 (InterPro), Mitochondrial inner membrane translocase complex, subunit Tim17/22 PF02466 (Pfam), Tim17/Tim22/Tim23 family, 5.8e-07	NI	DAS: 4 Transmembrane segments, cutoff 1.7<br>TMpred: 3 Transmembrane segments	PHYRE: multi α-helical structure	PHYRE: 65% Phase 1 flagellin, 1.4
**OXA complex**							
**Oxa1a**	Esi0028_0040 (412)	MSTAAGTGHSGDVATTADLGPDAMAGGADAAMAAAGTADQAQAALEVAPETFKMALYTPPQV<br>AMMAVDYVHATTGMPYWMTIVAITVGIRTAILPIGLLAARNGARTAAMKPEMDELQAAIKGDQQ<br>SSQPRKADRYRQETKALFQKHKASLVMNAALPIVQLPLFIGFFLGLRRMPDVVPEFATGGVLWFQ<br>DLGAPDPYMIFPVMTGVMMMAMAELGGEGGALAGSSVKMKAGMRGMALLVTPLTMYVSTGV<br>FVYWTTSNFYSILQTLAFKSSGIKKFFDFPDLPPNKLKSNTAEKEIGWLDVLGGDHPIKEYRKMQD<br>QREVQAWFVEGEGGWTRKAHNAEQGEGGNVAREGAPFHAPPSMAGPVSGKAKTPSFASSAAAG<br>TPKVVLRSVRPKPAGDRGKTAARGPRKNRG	**IPR001708 (InterPro), Membrane insertion protein, OxaA/YidC PTHR12428 (HMMPanther), Oxa1, 3.3e-58** PF02096 (Pfam), 60Kd inner membrane protein, 3.9e-40	**KOG1239, Inner membrane protein translocase involved in respiratory chain assembly**	DAS: 4 Transmembrane segments, cutoff 2.2<br>TMpred: 4 Transmembrane segments	PHYRE: multi α-helical structure	PHYRE: 95% membrane protein / hydrolase
**Oxa1b**	Esi0025_0161 (553)	MLTGARLSRQSRRLVGRVHGNGASLTAPAIAENRLTHHDGSDNSKRTCRGYLRPPQHLLQAQTL<br>HVPALQQRGHRSSSVTRLGGDGSAIGAAALGGGRGLDLSAAWRGAAAAGGTVGARRSFWGRSG<br>GDGSQAGSSGSGSDSGSGSEDSAGFPETGGAADADGWSAPPDAWAPDEVPPEIPQDASVPGFDVG<br>EGFSVDSAAAALDAVAGAAPDASAVGTAMTATGLSAADLGMYPHHLFMHVIEYVQATAGVPYW<br>EAIVMVSVAARIAVLPAVATFLGMSKRLNMIKPEMAVHQGKMQDIKNRMEANPEIKEAAMAEM<br>MLVSQEMGNLLKQHRIHFPKMMLSMFAQFPVFISLFLATRDMGTYFPGYMTGGLDWMMNLNAP<br>DPTWTLPILTSGSMILLMELGSDMPAAHGPDKPNFNPKVMFRVMSVVFVPVAFSMPAGVLVYWT<br>TTNVFGMLQRGLFEMRPVQQALGWPLPEDMPAPAAPADKKEPAEADFASKEDLERWGGGGEGA<br>SGEASRAAAEAAERAREAVAKASKVRHEELKRQHGERSGEDGPK	**IPR001708 (InterPro), Membrane insertion protein, OxaA/YidC PTHR12428 (HMMPanther), Oxa1, 5.3e-41** PF02096 (Pfam), 60Kd inner membrane protein, 4.1e-34	**KOG1239, Inner membrane protein translocase involved in respiratory chain assembly**	DAS: 4 Transmembrane segments, cutoff 2.2<br>TMpred: 5-6 Transmembrane segments	PHYRE: multi α-helical structure	PHYRE: 95% membrane protein, transport protein
**Oxa1c**	Esi0170_0057 (261)	MHVMGGVQSGVQAVHHTTGLPWWATIAVATIGVKISLLPVVVVYQAGHMDRMRAAWPEIQILRG<br>YLATSLEEIPQERVLERWRKYKVFFSGARGVLGLHGTHLRGMFATPLVNLPVFITFVWSIRGMLR<br>DGTVPGLDTGEADTVHSLMLPIIGTLCTYTSLEIVKMKGATGWMKFFQDGMQTFIILMLPWVSTF<br>PQGVFMYWIPSAVFQMGQTYTMKNNKVRELLGLKPLGLPPREAAGATPSLAPAPAPAPIPAPKAI<br>GG	**IPR001708 (InterPro), Membrane insertion protein, OxaA/YidC PTHR12428 (HMMPanther), Oxa1, 3.8e-32** PF02096 (Pfam), 60Kd inner membrane protein, 2.1e-19	**KOG1239, Inner membrane protein translocase involved in respiratory chain assembly**	DAS: 4 Transmembrane segments, cutoff 2.2<br>TMpred: 4 Transmembrane segments	PHYRE: multi α-helical structure	PHYRE: 95% Voltage-gated potassium channels
**TAT system**							
**TatA/B**	Esi0067_0034 (171)	MKLACCVLVWACALAARVAEAFVPALAGRAGIMSAAGRQASMSPAAAGRQNAFSHTSYGSPAQ<br>AQSSRWLSGRPSSGASTTTMGLFGLGAPEIAVCIAVAALILGPDKMAGFAKDMGKMAGELKDVP<br>KEFQAGVEEGEAKTQKTIREIESNGLAVEDAEPATKKEEEEKA	**PF02416 (Pfam), mttA/Hcf106 family**	NI	DAS: 2 Transmembrane segments, cutoff 2.2<br>TMpred: 2 Transmembrane segments	PHYRE: multi α-helical structure	PHYRE: 25% crystal structure of the c-terminal tropomyosin fragment2 with n- and c-terminal extensions of the leucine zipper
**TatC**	mt genome (254)	MKRLQLSIYYIQELNYRLAYAALGTTLIFFTTYTYKQGLIFLLLPKGLSHFVSAGLTEIFFTYIQLCII<br>LSISFGIFLIASQSYVFLRPGMYTYESSTFLKLIISAFCFYTYIYILVFPALIKVSWELFLTYSQNFTPIN<br>LTFEPRLNNYLDHIQQLNKILIFSFPCLLTLNLFQKYTNKQLWVKHRGIAYIIAFFIAAFITPPDIISQI<br>LVGAPLIFFFEIQIILWAFYKEYKKQLLVRQPIKSHKNTLRNKE	**PF00902 (Pfam), Sec-independent protein translocase protein (TatC)**	NI	DAS: 7 Transmembrane segments, cutoff 2.2<br>**TMpred: 6 Transmembrane segments**	PHYRE: multi α-helical structure	PHYRE: 85% Rhodopsin-like, 0.014
