# Supplementary material for: In Silico Survey of the Mitochondrial Protein Uptake and Maturation Systems in the Brown Alga Ectocarpus siliculosus
Source: PLoS One. 2011 May 18;6(5):e19540. doi: 10.1371/journal.pone.0019540 (PMC3097184; doi:10.1371/journal.pone.0019540)
Supplement: Figure S5 — Alignment of the C-terminal regions of Oxa1 family proteins. C-terminal domains characteristic of Oxa1 and Oxa2 are boxed. (PDF) [file pone.0019540.s005.pdf]

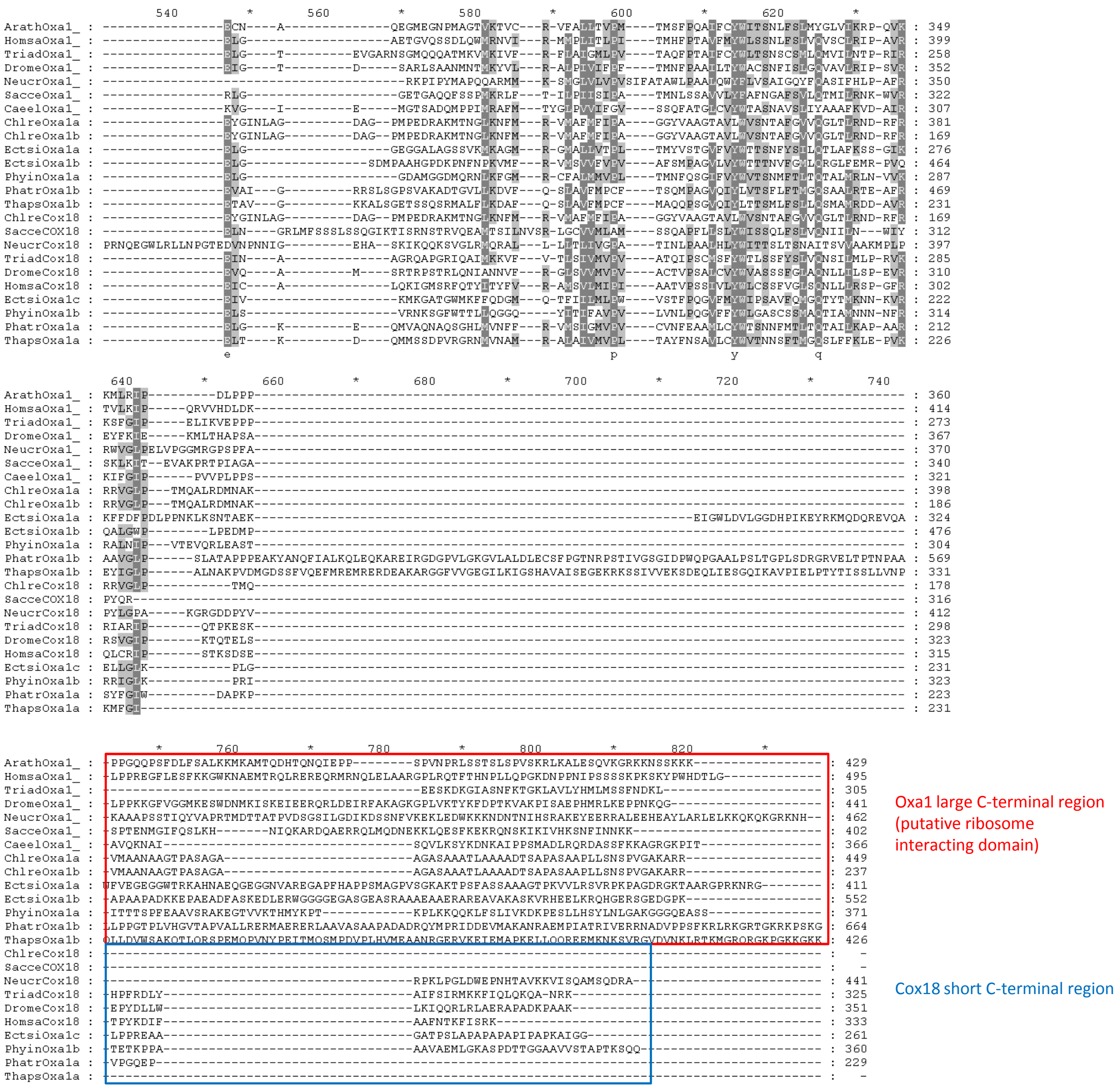
Oxa1 large C-terminal region
(putative ribosome
interacting domain)
Cox18 short C-terminal region
