# Supplementary material for: In Silico Survey of the Mitochondrial Protein Uptake and Maturation Systems in the Brown Alga Ectocarpus siliculosus
Source: PLoS One. 2011 May 18;6(5):e19540. doi: 10.1371/journal.pone.0019540 (PMC3097184; doi:10.1371/journal.pone.0019540)
Supplement: Table S3 — Mitochondrial protein import components across the eukaryotic tree. These accessions have been used in amino acid alignments and phylogenetic analyses. Note that the table includes both proteins identified in previous published studies and proteins found in this study by manual searches. (PDF) [file pone.0019540.s009.pdf]

| PROTEIN | STRAMENOPILES | | | | ALVEOLATES | | | PLANTAE | | | | | | | AMOEBOZOA | OPISTHOKONTS | | | | | | EXCAVATA | | | | |
|---|---|---|---|---|---|---|---|---|---|---|---|---|---|---|---|---|---|---|---|---|---|---|---|---|---|---|
| | BROWN ALGAE | DIATOMS | | OOMYCETES | CILIATES | APICOMPLEXANS | | RED ALGAE | GREEN ALGAE | | | MOSSES | LAND PLANTS | | | ANIMALS | | | | FUNGI | | JAKOBIDS | TRYPANOSOMATIDS | HETEROLOBOSEANS | DIPLOMONADS | PARABASALIDS |
| | *E. siliculosus* | *P. tricornutum* | *T. pseudonana* | *P. infestans* | *P. tetraurelia* | *P. falciparum* | *T. gondii* | *C. merolae* (red algae) | *O. luminarinus* | *M. pusilla* | *C. reinhardtii* | *P. patens* | *P. sitchensis* | *A. thaliana* | *D. discoideum* | *H. sapiens* | *D. melanogaster* | *C. elegans* | *T. adhaerens* | *S.cerevisiae* | *N. crassa* | *R. americana* | *T. brucei* | *N. gruberi* | *G. intestinalis* | *T. vaginalis* |
| Name | Accession (aa length) | Accession (aa length) | Accession (aa length) | Accession (aa length) | Accession (aa length) | Accession (aa length) | Accession (aa length) | Accession (aa length) | Accession (aa length) | Accession (aa length) | Accession (aa length) | Accession (aa length) | Accession (aa length) | Accession (aa length) | Accession (aa length) | Accession (aa length) | Accession (aa length) | Accession (aa length) | Accession (aa length) | Accession (aa length) | Accession (aa length) | Accession (aa length) | Accession (aa length) | Accession (aa length) | Accession (aa length) | Accession (aa length) |
| **OUTER MEMBRANE** | | | | | | | | | | | | | | | | | | | | | | | | | | |
| **mtOM64** | NI | NI | NI | NI | NI | NI | NI | ? | XP_001420347 (576) | EEH56501 (432) | XP_001698766 (507) | | | NP_196504 | NI | NI | NI | NI | NI | NI | NI | NI | NI | NI | NI | NI |
| **TOM complex** | | | | | | | | | | | | | | | | | | | | | | | | | | |
| **Tom70a** | Esi0007_0019 (952) | NI | NI | EEY66046 | **NI** | **NI** | **NI** | **NI** | **NI** | **NI** | **NI** | **NI** | **?** | **NI** | **NI** | NP_055635 (608) | NP_609536 | **NI** | XP_002112180 (567) | EDN62356 (639) | AAD21979 (624) | **NI** | **NI** | **NI** | **NI** | **NI** |
| **Tom70b** | Esi0232_0002 (739) | XP_002181625 (640) | XP_002291422 (219*) | EEY69545 (595) | XP_001429606 | XP_001348498 | EEE29632 (608) | NI | NI | NI | NI | XP_001764673 | ? | AAM61607 (277) | NI | NP_055635 (608) | NP_609536 | NI | XP_002112180 (567) | EDN62356 (639) | AAD21979 (624) | NI | NI | NI | NI | NI |
| **Tom40** | Esi0055_0058 (442) | XP_002182279 | XP_002293745 | EEY60531 | NI | CAG24986 | EEE23864 | AP006488 (*) | XP_001417025 | EEH60685 | EDP06354 (339) | XP_001783489 | ? | NP_188634 (309) NP_175457 (310) | EAL68763 | O96008 | NP_648417 | XP_002680549 | XP_002111548 (305) XP_002107842 (325) | NP_013930 (387) | XP_961545 | NI | XP_828689 | XP_002680549 | NI | NI |
| **Tom22** | Esi0246_0018 (112) | XP_002179609 | NI | EEY62448 | NI | XP_001351803 | NI | NI | XP_001416904 | EEH59409 | Scaffold 38 DNA (*) | XP_001783851 XP_001755556 | ADE77091 (115) | NP_563699 (94) Q9FNC9 (99) | NI | BAB16408 | NP_523910 | NP_493741 | XP_002109797 (135) | NP_014268 (152) | XP_956695 | NI | NI | NI | NI | NI |
| **Tom20-opisthokont** | NI | NI | NI | NI | NI | NI | NI | NI | NI | NI | NI | NI | ? | NI | | AAH66335 | NP_649169 | Q19766 | | NP_011596 | P35848 | NI | NI | NI | NI | NI |
| **Tom20-plant** | NI | NI | NI | NI | NI | NI | NI | NI | XP_001420673 (138) | EEH60139 (208) | XP_001692995 | XP_001780292 XP_001754924 | ABK23348 (205) ABK23751 (203) | NP_189343 NP_174059 NP_189344 NP_198909 | NI | NI | NI | NI | NI | NI | NI | NI | NI | NI | NI | NI |
| **Tom7** | Esi0179_0016 (57) | sequence in Macasev et al., 2004 | CP001160 (*) | NI | NI | NI | NI | NI | NI | EEH52506 (49) | EDP05834 (61) | XP_001764044 (56) XP_001758755 (56) | ABK21382 (72) | NP_568593 (75) Q3EC17 (117) | sequence in Macasev et al., 2004 | NP_061932 | NP_610433 (54) | NP_498706 (58) | NI | NP_014329 (60) | AAK18812 | NI | NI | NI | NI | NI |
| **Tom6** | NI | NI | NI | NI | NI | NI | NI | NI | NI | NI | EDO98875 (329) | ? | ? | NP_014688 (61) | NI | NP_001127965 | NI | NI | NI | NP_014688 (61) | ? | NI | NI | NI | NI | NI |
| **Tom5** | NI | NI | NI | NI | NI | NI | NI | NI | NI | NI | EDP08420 (54) | ? | ? | NP_015459 (50) | NI | NP_001001790 NP_001127957 NP_001127956 | NI | NI | NI | NP_015459 (50) | ? | NI | NI | NI | NI | NI |
| **SAM/TOB complex** | | | | | | | | | | | | | | | | | | | | | | | | | | |
| **Mim1** | NI | NI | NI | NI | NI | NI | NI | NI | NI | NI | NI | NI | NI | NI | NI | NI | NI | NI | NI | NP_014616 (113) | | ? | ? | ? | ? | ? |
| **Sam50** | Esi0503_0006 (451) | XP_002183360 (368) | XP_002290682 (672) | EEY65816 (435) | NI | NI | XP_002367788 | AP006497 (*) | XP_001418351 | EEH56200 | XP_001691355 | XP_001781071 | ADE77075 | NP_568157 | ? | NP_056195 | AAC72375 (428) | P46576 | XP_002109893 | NP_014372 (484) | XP_960555 (521) | ? | ? | ? | ? | ? |
| **Sam35/Metaxin2** | NI | NI | NI | NI | NI | NI | NI | NI | NI | EEH60938 | EDP05944 | XP_001779589 XP_001777829 | ? | NP_565446 | NI | NP_006545 | NP_649161 | NP_498689 | XP_002116555 | NP_011951 (329) | NI | NI | NI | NI | NI | NI |
| **Sam37/Metaxin1** | Esi0338_0018 (407) | XP_002179017 | XP_002292973 | EEY57486 | NI | NI | ? | ? | ? | NI | NI | NI | ? | | NI | NP_002446 | NP_649908 | NP_493569 | XP_002112282 | NP_013776 (327) | NI | ? | ? | ? | ? | ? |
| **INTERMEMBRANE SPACE** | | | | | | | | | | | | | | | | | | | | | | | | | | |
| **MIA/ERV complex** | | | | | | | | | | | | | | | | | | | | | | | | | | |
| **Mia40** | NI | XP_002182915 | XP_002292670 | EEY62204 (1468) | NI | NI | NI | AP006492 (168) | XP_001419132 | EEH56979 | NI | XP_001765902 | ? | NP_680211 (162) | XP_643501 (97) | 2K3J (142) | NP_733343 (158) | NP_500803 (125) | XP_002108194 (114) | NP_012726 (403) | XP_959393 | NI | NI | NI | NI | NI |
| **Erv1** | Esi0202_0015 (194) | XP_002185840 | XP_002291504 | EEY69279 | XP_001448726 | XP_001351032 | EEE22130 | AP006502 (*) | XP_001417101 | EEH60024 | EDP03768 (140) | XP_001751213 | ADE75626 (214) | NP_564557 (191) | XP_645302 | CAB87993 | NP_608353 (261) | NP_490690 | XP_002110094 | NP_011543 (189) | XP_959716 (199) | NI | XP_001022007 | NI | NI | NI |
| **Hot13** | Esi0046_0129 (101) | EEC47891 | NI | EEY65160 | XP_001445542 | NI | NI | AP006495 (*) | ? | ? | XP_001703786 | XP_001703786 | ? | NP_197366 | EAL72440 | ? | ? | ? | ? | NP_012839 (116) | EAA28094 | ? | ? | ? | ? | ? |
| **TIM8/13 Complex** | | | | | | | | | | | | | | | | | | | | | | | | | | |
| **Tim13** | Esi0243_0012 (82) | XP_002183931 | NI | EEY56845 EEY53075 | ? | Q8ILN5 | ? | AP006491 (*) | ABO97719 (77) | EEH57037 (76) | EDO99447 (79) | XP_001754051 | ABK27025 (77) | NP_564780 (87) | NI | O60220 Q9Y5J9 | Q9Y1A3 | AAD39993 (83) | XP_002112736 | NP_011697 (105) | Q9Y8C0 | NI | NI | ? | ? | NI |
| **Tim8** | Esi0109_0044 (125) | XP_002182231 | XP_002294681 ? | EEY55518 | ? | Q8I500 | ? | AP006484 (*) | ABO93935 (81) | EEH60245 (83) | EDP04523 (85) | XP_001773834 (89) | ABK21550 (87) | NP_199894 (77) | NI | AAF15101 AAF15102 | Q9VTN3 | AAD39955 (108) | XP_002112460 | NP_058168 (87) | Q7SBR3 | NI | EAN79502 | ? | ? | NI |
| **TIM9/10 Complex** | | | | | | | | | | | | | | | | | | | | | | | | | | |
| **Tim10** | Esi0041_0146 (107) | XP_002177509 (84) | XP_002294517 (66) | EEY57510 (103) | ? | Q8I5W2 | ? | AP006489 (*) | XP_001416863 (79) | EEH53236 (76) | EDO96752 (83) | XP_001757494 | ABK22209 (82) | NP_565682 (83) | EAL64919 | P62072 | Q9W2D6 | Q9Y0V6 (86) | XP_002107711 (90) | NP_011869 (93) | Q9C0N3 | NI | AAX80231 | ? | ? | NI |
| **Tim9** | Esi0075_0052 (88) | XP_002182231 (92) | XP_002295276 (84) XP_002295313 (81) | EEY66230 (85) | ? | Q8ID24 | XP_002368151 | AP006500 (*) | XP_001422729 (95) | NI | EDP09353 (103) | XP_001774597 (90) XP_001753519 (90) | ABR16754 (93) | NP_190240 (93) | EAL71103 | Q9Y5J7 | Q9VYD7 | Q61TH2 (78) Q17754 (90) | XP_002113530 (69) | NP_010894 (87) | Q8J1Z1 | NI | AAX69615 | ? | ? | NI |
| **INNER MEMBRANE** | | | | | | | | | | | | | | | | | | | | | | | | | | |
| **TIM23 complex** | | | | | | | | | | | | | | | | | | | | | | | | | | |
| **Tim50** | Esi0000_0471 (547) | XP_00217713 (220*) | XP_002289544 (116*) | EEY55063 (385) Q5S7T7 (409) | XP_001450869 | NI | NI | AP006496 (*) | XP_00141704 (237) | EEH60713 (450) | EDP05270 (729) | XP_001775518 (122*) | NI | NP_175986 (376) | XP_635771 (567) | NP_001001563 (456) | NP_570027 (428) | NP_505722 (452) | XP_002113553 (246) | NP_015262 | AAO32939 | NI | NI | XP_002683376 (223) | NI | NI |
| **Tim44** | Esi0086_0051 (602) | XP_002178476 | XP_002289749 | EEY68783 (467) | XP_001435479 (405) | XP_001347936 (470) | XP_002366406 (598) | AP00649 (*) | XP_001420450 (286) | EEH54071 (289) | XP_001697863 | XP_001762763 | NI | NP_565473 NP_181151 | NI | NP_006342 | NP_732403 | NP_491780 | XP_002110490 (473) | NP_012242 | XP_965329 | NI | NI | XP_002674337 (520) | NI | XP_001311960 (326) |
| **Tim23** | Esi0047_0026 (206) | XP_002180317 (202) | XP_002294983 (107*) | EEY55562 (206) | XP_001436200 (159) | XP_001350273 (167) | XP_002370748 (188) | AP006497 (*) | XP_001416908 | EEH59889 (263) | ? | XP_001784087 (174*) | ABK23235 | AAK31587 (187) NP_177419 (188) NP_187131 | NI | NP_006318 (209) | NP_001015387 (206) | NP_492953 | XP_002116263 (198) | NP_014414 | XP_322457 | NI | NI | NI | NI | NI |
| **Tim21** | Esi0103_0070 (205) | Phatr2_42559 | Thaps3_4318 | EEY67838 (229) | NI | NI | NI | NI | XP_001416554 (203) | EEH59720 (171*) | EDP00063 (250) | XP_001783295 (355) | NI | NP_001031562 (269) | NI | AAF29118 (248) | NP_608929 (236) | NP_499991 (213) | XP_002112064 (317) | P53220 | XP_961997 | NI | NI | NI | NI | NI |
| **Tim17** | Esi0117_0080 (264) | XP_002176261 (125*) | XP_002297575 (140*) | EEY55736 | XP_001446003 (203) | XP_001348502 (162) | XP_002364444 (213) | AP006501 (*) | XP_001415788 (136) | EEH59563 (206) | XP_001698342 | XP_001781469 (238) | NI | NP_173460 (218) NP_181277 (243) NP_196730 (133) | XP_637129 (183) | NP_006326 (171) NP_005825 (172) | NP_001015401 (173) NP_524746 (176) | NP_500627 (181) | XP_002117827 (147) | NP_012392 (158) | XP_325478 | NI | NI | XP_002676796 (130) | NI | NI |
| **Pam17** | NI | NI | NI | NI | NI | NI | NI | NI | NI | NI | NI | NI | NI | NI | | NI | NI | NI | XP_002112266 (188) | NP_012991 (197) | XP_964390 (267) | NI | NI | NI | NI | NI |
| **Tim16/Pam16** | Esi0237_0006 (155) | XP_002178349 | XP_002293579 | EEY66595 | XP_001451517 | XP_001351692 | XP_002367323 | AP006493 (*) | XP_001419952 | EEH57687 (181) | EDP00908 | XP_001761102 (115) XP_001757252 (117) | ABK21068 (115) | NP_851243 | XP_640279 | NP_057153 | NP_524370 (141) | NP_499775 (136) | XP_002112778 | NP_012431 (149) | XP_960477 | NI | NI | XP_002683317 (150) | NI | XP_001309776 (111) |
| **Tim14/Pam18** | Esi0159_0032 (209) | XP_002180499 (67*) | XP_002290857 (95*) | EEY64820 (152) | XP_001460806 (107) | XP_001349146 (115) | XP_002367564 (140) | AP006498 (*) | XP_001417918 (111) | EEH55216 (110) | EDP02285 (114) | XP_001772964 (112) | ABK25685 | NP_565824 | XP_638992 (114) | NP_660304 (116) | NP_648475 (118) | NP_491662 (112) | XP_002109813 (131) | NP_013108 | XP_322161 (105) | NI | XP_847461 (120) | XP_002675014 (132) | XP_002364144 (105) | XP_001581820 (117) |
| **mtHsp70** | Esi0010_0066 (689) | XP_002177297 (673) | XP_002295809 (670) | EEY64540 (663) | XP_001461379 (665) | XP_001348022 (663) | XP_002367417 (728) | AP006494 (*) | XP_001417572 (674) | EEH58878 (732) | EDP02463 (607) | XP_001763211 (676) XP_001774874 (680) | NI | NP_195504 NP_196521 (682) | XP_629204 (658) | NP_004125 (679) | NP_523741 (686) | NP_504291 (657) | XP_002117735 | NP_012579 (654) | XP_329817 (516) | NI | XP_845492 (657) | XP_002681196 (649) | BAB69031 | XP_001580703 |
| **Mge1** | Esi0000_0394 (281) | XP_002182951 (157*) | XP_002289839 (178*) | EEY58699 (218) | XP_001346838 (273) | XP_001347929 (301) | EEE31125 (347) | APO006494 (*) | XP_001417453 (240) | EEH58010 (150) | XP_001701310 (264) | XP_001773453 (302) XP_001775604 (132) | ABK25023 (322) | NP_200331 (302) NP_567757 (327) | NP_079472 (217) | NP_610886 (213) | NP_497713 (237) | XP_002109822 (191) | XP_638912 (213) | NP_014875 (228) | XP_327955 (238) | NI | XP_845338 (222) | XP_002680804 (283) | NI | XP_001329309 (191) |
| **TIM22 complex** | | | | | | | | | | | | | | | | | | | | | | | | | | |
| **Tim54** | NI | NI | NI | NI | NI | NI | NI | NI | NI | NI | NI | NI | NI | NI | NI | NI | NI | NI | NI | NP_012481 (478) | XP_327581 (425) | NI | NI | NI | NI | NI |
| **Tim22** | Esi0063_0018 (192) | XP_002176835 (136*) | XP_002288595 (114) | EEY60403 (116) EEY66293 (163) | XP_001458532 | XP_966257 (193) | XP_002366234 | APO006501 (*) APO006502 (*) | XP_001417996 (200) | EEH58567 (143) | XP_001689511 (192) | XP_001761114 (144*) XP_001771365 (132*) XP_001755119 (123*) | ABK23272 (208) ABK25689 (143) | NP_566368 (173) NP_567754 (210) NP_200362 (214) | XP_638851 (179) | NP_037469 (194) | NP_732364 (195) | NP_496412 (213) | NI | NP_010064 (207) | XP_323099 (194) | NI | XP_829386 (152) | XP_002678961 (134*) | NI | NI |
| **Tim18** | NI | NI | NI | NI | NI | NI | NI | NI | NI | NI | NI | NI | NI | NI | NI | NI | NI | NI | NI | NP_014940 (192) | NI | NI | NI | N | NI | NI |
| **Tim12** | NI | NI | NI | NI | NI | NI | NI | NI | NI | NI | NI | NI | NI | NI | NI | NI | NI | NI | NI | NP_009649 (109) | NI | NI | NI | NI | NI | NI |
| **OXA complex** | | | | | | | | | | | | | | | | | | | | | | | | | | |
| **Oxa1a** | Esi0028_0040 (411) | XP_002177276 (664) | XP_002292903 (426) | EEY61447 (371) | XP_001452857 (358) XP_001442355 (361) | XP_00134924 (589) | NI | AP006485 (*) AP006487 (*) | XP_001418188 (304) XP_00141692 (271*) | EEH56601 (260) EEH54616 (582) | ACM07437 (449) ACL98473 (237) | XP_001782477 (419) XP_001774924 (252*) | NI | NP_201011 (429) NP_182170 (431) | XP_639906 (396) | NP_005006 (495) CAD62623 (262) | NP_648417 (441) | NP_493356 (366) | | NP_011081 | AAL30813 (462) | NI | XP_828689 (418) | XP_002680549 (226*) XP_002674062 (424) | NI | NI |
| **Oxa1b** | Esi0025_0161 (552) | XP_002177276 (664) | XP_002292903 (426) | EEY61447 (371) | NI | NI | NI | AP006485 (*) AP006487 (*) | XP_001418188 (304) | EEH56601 (260) | EDO98825 (178) | XP_001782477 (419) | NI | NP_182170 (431) NP_201011 (429) | XP_639906 (396) XP_640859 (418) | NP_776188 (333) | NP_648286 (351) | NP_493356 (366) | XP_640859 (418) | CAA97064 | XP_963619 (441) | NI | XP_828689 (418) XP_827302 (424) | XP_002680549 (226*) XP_002674062 (424) | NI | NI |
| **Oxa1c (Cox18)** | Esi0170_0057 (260) | XP_002177364 (229*) | XP_002290599 (231*) | EEY60615 (360) | NI | NI | NI | NI | NI | NI | NI | NI | NI | NI | NI | NI | NI | NI | NI | NI | NI | NI | NI | NI | NI | NI |
| **TAT system** | | | | | | | | | | | | | | | | | | | | | | | | | | |
| **TatA/B** | Esi0067_0034 (170) | XP_002181489 XP_002181490 | XP_002287992 XP_002294855 | NI | NI | NI | NI | ? | ? | EEH59514 | XP_001690564 | XP_001774935 | ABK21127 | NP 198227 | NI | NI | NI | NI | NI | NI | NI | NI | NI | NI | NI | NI |
| **TatC** | mt genome | ? | OK | OK (248) | ? | ? | ? | OK (267) | OK | ? | ? | OK | ? | Ath2 (277) Ath3 (277) | NI | NI | NI | NI | NI | NI | NI | OK (260) | ? | OK | ? | ? |

* incomplete sequence
